# Supplementary material for: SHP-1 agonist SC-43 limits methicillin-resistant Staphylococcus aureus infection through inhibition of heme biosynthesis
Source: EMBO Mol Med. 2026 Apr 10;18(5):1990–2005. doi: 10.1038/s44321-026-00418-4 (PMC13179323; doi:10.1038/s44321-026-00418-4)
Supplement: Supplementary file 1 — Appendix [file 44321_2026_418_MOESM1_ESM.pdf]

Table of contents

Contents	Page numbers
Appendix Figure S1	2
Appendix Figure S2	3
Appendix Figure S3	4
Appendix Table S1	5-26
Appendix Table S2	27
Appendix Table S3	28

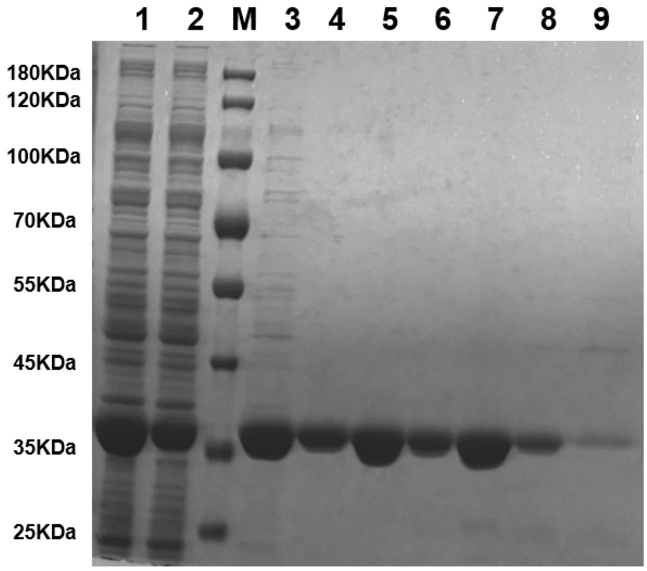

Appendix Figure S1. Protein purification of SA_{CpfC}.
Lane M: protein ladder, lane 1: membrane fraction before column, lane 2: Flow through, lane 3: washed with 10mM imidazole, lane 4: washed with 20mM imidazole, lane 5-6: washed with 50mM imidazole, lane 7-8: washed with 100mM imidazole, lane 9: washed with 500mM imidazole.

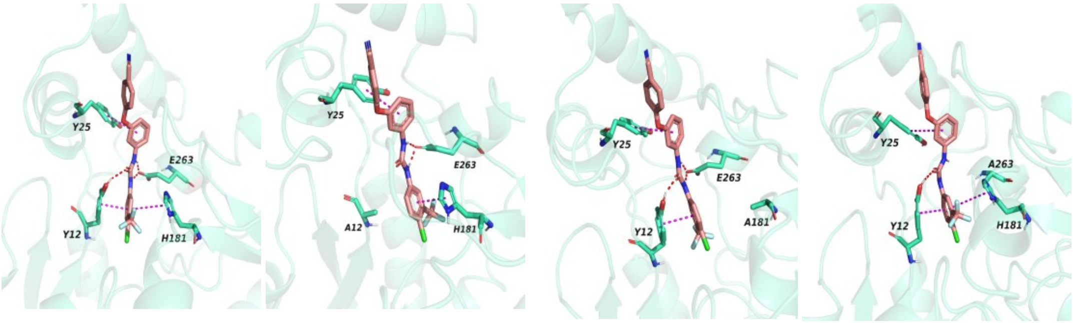

**Wild-type**	**Y12A mutant**	**H181A mutant**	**E263A mutant**
Glide GScore: -5.505	Glide GScore: -3.627	Glide GScore: -3.762	Glide GScore: -2.698

Appendix Figure S2. Molecular docking of SC-43 with the wild-type SACpfC protein and its Y12A, H181A, and E263A mutants.

The binding poses and corresponding Glide GScores are shown. Carbon atoms of the ligand (SC-43) and the protein are colored in wheat and cyan, respectively. Hydrogen bonds and π-π interactions are represented by red and magenta dashed lines, respectively. In the Y12A mutant complex, the mutation to alanine abolishes both a critical hydrogen bond and a π-π stacking interaction that were observed with the tyrosine residue in the wild-type protein. Similarly, the binding poses for the H181A and E263A mutants show the loss of key interactions present in the wild-type complex.

```
1     ............................................................

1     MVRWFHRDLSGLDAETLLKGRGVHGSFLARPSRKNQGDFSLSVRVGDQVTHIRIQNSGDF

1     ..........................MTKKMGLLVMAYGTPYKESDIEPYYTDIRHGKRP
                                 .:. .::.: |.  ..:.. |.:| : . | .
61    YDLYGGEKFATLTELVEYYTQQQGVLQDRDGTIIHLKYPLNCSDPTSERWYHGHMSGGQ.

35    SEEELQDLKDRYEFIG.......GLSPLAGTTDDQADALVSALNKAYADVEFK...LYLG
      .|. ||.  :.:.|:.       |  .|.. .|:. .: .|:|. .  .|  .   . :|
120   AETLLQAKGEPWTFLVRESLSQPGDFVLSVLSDQPKAGPGSPLRVTHIKVMCEGGRYTVG

85    LKHISPFIEDAVEQMHNDGITEA...ITVVLAPHYSS.FSVGSYDKRADEEAAKYGIQLT
        .. . :.| ||:....||.||   :. : .| |.. ...:..:.|. | . | : : |
180   GLETFDSLTDLVEHFKKTGIEEASGAFVYLRQPYYATRVNAADIENRVLELNKKQESEDT

141   HVKHYYEQPKFIEYWTNK..VNETLAQIPEEEHKDTVLVVSAHSLPKGLIEKNNDPYPQE
          ::|: . ::  . |  ..   :| ||:. |:    : : . .:.::: .:. .|..
240   AKAGFWEEFESLQKQEVKNLHQRLEGQRPENKGKNRYKNILPFDHSRVILQGRDSNIPGS

199   LEHTALLIK.....EQSNIEHIAIGWQSEGNTGTPWLGPDVQDLTRDLYEKHQYKNFIYT
         .| .||     .:.| . .  : .. :.|...::. . |: .| :  . .  :   .
300   DYINANYIKNQLLGPDENAKTYIASQGCLEATVNDFWQMAWQENSRVIVMTTREVEKGRN

254   PVGFVCEHLEVLYDNDYECKVVCDDIGANYYRPKMPNTHPLFIGAIIDEIKSIF......
      ...   ..:::  . :  . . |:: :.. |: :  .. ||  |.:| ||   .
360   KCVPYWPEVGMQRAYGPYSVTNCGEHDTTEYKLRTLQVSPLDNGDLIREIWHYQYLSWPD

308   ............................................................

420   HGVPSEPGGVLSFLDQINQRQESLPHAGPIIVHCSAGIGRTGTIIVIDMLMENISTKGLD

308   ............................................................

480   CDIDIQKTIQMVRAQRSGMVQTEAQYKFIYVAIAQFIETTKKKLEVLQSQKGQESEYGNI

308   ........................................................

540   TYPPAMKNAHAKASRTSSKHKEDVYENLHTKNKREEKVKKQRSADKEKSKGSLKRK
```

Appendix Figure S3. Alignment of SA-CpfC.seq (upper line) and PTN6.seq (lower line).

Identity = 16.34% (50/306), similar residues = 49.35% (151/306), gap = 48.66% (290/596).

Appendix Table S1. The antibacterial activity screening of a phase 1-3 clinical compound library against *S. aureus* ATCC 29213.

Compounds	MIC (μg/mL)
Atropine sulfate monohydrate	>10
Sodium Nitroprusside	>10
Physostigmine Salicylate	>10
Quetiapine	>10
Clopidogrel	>10
Tamsulosin hydrochloride	>10
Digitoxin	>10
Naproxen sodium	>10
Losartan	>10
Dasatinib monohydrate	>10
Amiloride hydrochloride dihydrate	>10
Lapatinib ditosylate	>10
Oxybutynin	>10
Pioglitazone hydrochloride	>10
Ziprasidone hydrochloride	>10
Capsaicin	>10
Perphenazine	>10
Promethazine hydrochloride	>10
β-Cyclodextrin	>10
Levamlodipine	>10
Prednisolone	>10
Erlotinib hydrochloride	>10
Pemetrexed disodium	>10
S-(+)-Ketoprofen	>10
Pergolide mesylate	>10
CPI0610	>10
Icotinib Hydrochloride	>10
Entecavir	>10
AS601245	>10
Scopolamine hydrobromide	>10
Lycopene	>10
Idazoxan hydrochloride	>10
Fosaprepitant dimeglumine	>10
Atazanavir	>10
Sorafenib	>10
CAY10603	>10
Tiagabine	>10
Dacinostat	>10
Vortioxetine hydrobromide	>10
Hematoporphyrin dihydrochloride	>10

WAY-181187	>10
Polydatin	>10
Aprotinin	>10
Vitamin D3	>10
KW-2478	>10
MK-1064	>10
Pralatrexate	>10
Carbidopa	>10
Fondaparinux sodium	>10
Liraglutide	>10
Angiotensin II human	>10
Octreotide Acetate	>10
Visomitin	5
PHA-767491	>10
GLP-1(7-36), amide	>10
Nirogacestat	>10
Resminostat hydrochloride	>10
Cyclosporine	>10
LTX-315	>10
Tauroursodeoxycholate sodium	>10
BMS-911543	>10
Spermidine trihydrochloride	>10
Oxytocin	>10
JTC-801	>10
RAF265	>10
Dabigatran Etexilate Mesylate	>10
Eicosapentaenoic Acid	>10
Docosahexaenoic Acid	>10
Rolapitant	>10
S 38093 HCl	>10
Atosiban acetate	>10
Desmopressin acetate (16679-58-6 free base)	>10
Oxytocin acetate	>10
Almonertinib	>10
DAPTA	>10
Aprocitentan	>10
Iberdomide	>10
Emodepside	>10
OSI-027	>10
GSK2982772	>10
Antineoplaston A10	>10
Diclofenac Potassium	>10
Larotrectinib	>10
zanubrutinib	>10

Elagolix sodium	>10
tafamidis meglumine	>10
Bivalirudin	>10
Pentadecanoic acid	>10
Remdesivir	>10
Pinaverium bromide	>10
Phenprocoumon	>10
Bremelanotide Acetate	>10
Indinavir sulfate	>10
Propafenone hydrochloride	>10
Flumatinib mesylate	>10
Zuranolone	>10
PT-2385	>10
Licarbazepine	>10
RGX-104	>10
AZD-5069	>10
Verucerfont	>10
Milnacipran ((1S-cis) hydrochloride)	>10
Mosapride	>10
Fingolimod	>10
Tianeptine	>10
Amsacrine hydrochloride	>10
Cholic acid sodium	>10
Imipramine hydrochloride	>10
Tiagabine hydrochloride	>10
L-Cysteine hydrochloride	>10
Sodium benzoate	>10
Quinidine sulfate dihydrate	>10
Iron sucrose	>10
Isonicotinic acid	>10
Rimeporide hydrochloride	>10
2-hydroxy Flutamide	>10
CBL0137	>10
FK962	>10
Quinidine	>10
Trametinib (DMSO solvate)	>10
Saquinavir	>10
Eniluracil	>10
Aminopterin	>10
Isoflavone	>10
Nelfinavir	>10
Cannabidivarin	>10
9-amino-CPT	>10
Ethyl pyruvate	>10

Dobutamine hydrochloride	>10
Nicotinamide riboside chloride	>10
Valrubicin	>10
Seletalisib	>10
AMG319	>10
Galanthamine	>10
Noscapine	>10
Lasmiditan hydrochloride	>10
Afuresertib hydrochloride	>10
Nidufexor	>10
E7046	>10
Emoxypine Succinate	>10
Transcrocetinate disodium	>10
Pyroxamide	>10
Enoximone	>10
PLX51107	>10
MAK683	>10
Belinostat	>10
Prexasertib	>10
Mobocertinib	>10
ODM-203	>10
TAS0728	>10
PF-06873600	>10
Cariprazine hydrochloride	>10
Trifluoperazine	>10
Ixazomib citrate	>10
Prednisone acetate	>10
Alectinib hydrochloride	>10
Ropivacaine hydrochloride monohydrate	>10
Crizotinib hydrochloride	>10
Mitiglinide Calcium	>10
Ulipristal	>10
Isosorbide Mononitrate	>10
Prucalopride Succinate	>10
Lasofoxifene Tartrate	>10
Selpercatinib	>10
Prasugrel Hydrochloride	>10
Lasmiditan succinate	>10
Trihexyphenidyl hydrochloride	>10
Desogestrel	>10
Metoclopramide hydrochloride hydrate	>10
Nomegestrol acetate	>10
Cyclopentolate Hydrochloride	>10
Isosorbide	>10

Cyproheptadine hydrochloride	>10
S-Adenosyl-L-methionine disulfate tosylate	>10
Ondansetron hydrochloride	>10
Dantrolene	>10
MRX-2843	>10
Deucravacitinib	>10
Vorolanib	>10
ZSET1446	>10
ASP4132	>10
GANAXOLONE	>10
VTP-27999 TFA	>10
Avacopan	>10
Paricalcitol	>10
LML134	>10
APX-115 free base	>10
AZD7325	>10
Blu-782	>10
Lersivirine	>10
Amiselimod hydrochloride	>10
GSK2018682	>10
DS-1001b	>10
Adagrasib	>10
Gaboxadol hydrochloride	>10
VLX600	>10
Derenofylline	>10
Obefazimod	>10
BNC210	>10
ONO-7475	>10
T-1101 tosylate	>10
Pralsetinib	>10
Empesertib	>10
Encequidar	>10
SX-682	>10
AB928	>10
Centhaquin	>10
GSK3145095	>10
Dapansutrile	>10
Vonafexor	>10
Alobresib	>10
lumateperone Tosylate	>10
Cebranopadol	>10
Osoresnontrine	>10
TNO155	>10
Sodium 2-oxopropanoate	>10

Triazavirin	>10
Esomeprazole Magnesium trihydrate	>10
Bosentan (hydrate)	>10
Afatinib	>10
Ceritinib dihydrochloride	>10
Masitinib mesylate	>10
Bendamustine	>10
Anagliptin	>10
Ripretinib	>10
Phentolamine	>10
Terazosin hydrochloride dihydrate	>10
Hydroxyprogesterone caproate	>10
Tofogliflozin (hydrate)	>10
Nitroprusside disodium dihydrate	>10
Albiglutide TFA (782500-75-8 free base)	>10
Pemigatinib	>10
BAY1082439	>10
Coenzyme A	>10
Edicotinib	>10
Emixustat hydrochloride	>10
Omaveloxolone	>10
Bradykinin	>10
Savolitinib	>10
Unesbulin	5
Substance P	>10
Tetracosactide	>10
Cibinetide	>10
Quisinostat dihydrochloride	>10
Tyroserleutide	>10
Carcinoembryonic Antigen CEA	>10
AZD3839 free base	>10
Argireline	>10
Dabrafenib Mesylate	>10
Ningetinib	>10
Derazantinib	>10
AV-412	>10
Silmitasertib sodium salt	>10
Ripasudil	>10
Lenvatinib mesylate	>10
Ribociclib succinate	>10
Metformin	>10
Alvespimycin hydrochloride	>10
Fluphenazine decanoate	>10
Naftopidil dihydrochloride	>10

Venlafaxine	>10
Ralfinamide mesylate	>10
Flecainide acetate	>10
SC-43	< 0.625
Glasdegib	>10
Udenafil	>10
Perhexiline maleate	>10
Delcasertib	>10
Telotristat ethyl	>10
Exatecan Mesylate	>10
Sparsentan	>10
Naratriptan	>10
Pentamidine	>10
Voxilaprevir	>10
Nilotinib monohydrochloride monohydrate	>10
cangrelor tetrasodium	>10
Buserelin Acetate (57982-77-1 free base)	>10
Tegobuvir	>10
Rilzabrutinib	>10
SEP-363856 hydrochloride	>10
Orelabrutinib	>10
USL311	>10
Verubulin hydrochloride	>10
AZD-8529 mesylate	>10
Inarigivir soproxil	>10
Temoporfin	>10
9-ING-41	>10
Farudodstat	>10
JNJ-40411813	>10
Pimitespib	>10
Enarodustat	>10
PRN1371	>10
Olutasidenib	>10
Lixisenatide acetate (320367-13-3 free base)	>10
Brepocitinib P-Tosylate	>10
CB-103	>10
Sotorasib	>10
Velneperit	>10
S-Nitroso-N-acetyl-DL-penicillamine	>10
Basimglurant	>10
Dopexamine hydrochloride	>10
Evocalcet	>10
ATN-161 trifluoroacetate salt	>10
Harringtonine	>10

β-Elemene	>10
Bradykinin (acetate)	>10
Hydroxyzine	>10
Tafluprost	>10
Vazegepant hydrochloride	>10
Milciclib	>10
Taletrectinib	>10
RBN-2397	>10
Calcimycin	>10
KO-947	>10
Niclosamide olamine	>10
Denifanstat	>10
Nicardipine	>10
Dovitinib lactate hydrate	>10
PF04929113	>10
PF-543 hydrochloride	>10
Lurasidone	>10
L-Cysteine hydrochloride hydrate	>10
Regorafenib monohydrate	>10
Beclometasone	>10
Elimusertib	>10
Vatalanib free base	>10
Poziotinib hydrochloride	>10
Capmatinib 2HCl.H2O	>10
Ro 5126766	>10
APTO-253	>10
Tolimidone	>10
Tanzisertib	>10
Lerociclib dihydrochloride	>10
Inobrodib	>10
TK216	>10
Evacetrapib	>10
ASTX660	>10
Ipamorelin 2acetate(170851-70-4(free base))	>10
TAK-243	>10
Dimethylcurcumin	>10
Sonidegib diphosphate	>10
Presatovir	>10
TAK-071	>10
Velsecorat	>10
Hydroxocobalamin acetate	>10
BAY-2402234	>10
TAK-700	>10
GSK2838232	>10

ARQ 531	>10
Thymalfasin	>10
Harmaline hydrochloride	>10
Oleoylethanolamide	>10
Miglustat	>10
Fibrin	>10
Olsalazine	>10
Cinobufotalin	>10
Secoisolariciresinol	>10
Vericiguat	>10
Lipoic acid	>10
Hexaminolevulinate hydrochloride	>10
Sodium stibogluconate	>10
Tenalisib	>10
Treosulfan	>10
Perflubron	>10
Rigosertib	>10
Peretinoin	>10
Remetinostat	>10
Etifoxine hydrochloride	>10
Linaclotide	>10
Fosravuconazole L-lysine ethanolate	>10
Glycerol phenylbutyrate	>10
Opicapone	>10
Indibulin	>10
L-Thyroxine sodium	>10
Fulacimstat	>10
Finerenone	>10
K-604 dihydrochloride	>10
AMG131	>10
4-MMPB	>10
Glucagon (1-29), bovine, human, porcine hydrochloride	>10
GW-870086	>10
Fluzoparib	>10
Semaglutide	>10
ONC206	>10
MK-0557	>10
Copanlisib dihydrochloride	>10
Teriparatide acetate	>10
E7449	>10
Capromorelin Tartrate	>10
Navoximod	>10
Aviptadil Acetate	>10
Icatibant Acetate	>10

Pinealon Acetate	>10
Terlipressin Acetate	>10
Arimoclomol maleate	>10
Lipopolysaccharides	>10
Arhalofenate	>10
Sotorasib racemate	>10
Isoflurane	>10
LY-2584702 free base	>10
Amcenestrant	>10
Elexacaftor	>10
AG-120 (racemic)	>10
AMG 925	>10
Retigabine dihydrochloride	>10
PF-06882961	>10
Carboxyamidotriazole Orotate	>10
Gemcitabine elaidate hydrochloride	>10
Lanraplenib	>10
Deferitrin	>10
Remibrutinib	>10
Triptorelin acetate(57773-63-4 free base)	>10
Metyrosine	>10
Almotriptan	>10
PF-06882961 Tris	>10
Solabegron	>10
Ilginatinib	>10
Rucaparib monocamsylate	>10
Metarrestin	>10
TAS6417	>10
PRX-08066	>10
Pamiparib	>10
HPPH	>10
Relacorilant	>10
CD73-IN-3	>10
Cenicriviroc	>10
Sulforaphane	>10
Gefarnate	>10
Nevanimibe hydrochloride	>10
Rosiptor	>10
Vodobatinib	>10
Seralutinib	>10
Rotigotine Hydrochloride	>10
(R)-Elagolix	>10
GSK256073	>10
Samuraciclib hydrochloride	>10

Trientine-2HCl	>10
Salcaprozate sodium	>10
AER-271	>10
Nafarelin acetate(76932-56-4 free base)	>10
Tegoprazan	>10
Salmeterol	>10
Soticlestat	>10
Polmacoxib	>10
PF-3758309	>10
Eleclazine hydrochloride	>10
Lisinopril	>10
XL228	>10
Pyropheophorbide-a	>10
Phenylephrine	>10
Cabergoline	>10
HALOFUGINONE LACTATE	>10
Benin	>10
LY900009	>10
Mal-amido-PEG2-C2-amido-Ph-C2-CO-AZD	>10
BGC-20-1531 hydrochloride(1186532-61-5 free base)	>10
ARQ 621	>10
Dopamine	>10
Valemetostat	>10
JNJ-63576253 free base	>10
NVR 3-778	>10
Brincidofovir	>10
ACT-389949	>10
BOS-172722	>10
CA-4948	>10
Dexpramipexole dihydrochloride	>10
Lucitanib	>10
Liarozole	>10
Dorzagliatin	>10
Etrasimod	>10
Ramosetron hydrochloride	>10
PF-04995274	>10
Traxoprodil	>10
Ilginatinib maleate	>10
Fenebrutinib	>10
AB-423	>10
Etalocib	5
Fezolinetant	>10
Zimlovisertib	>10

AZD9977	>10
Arundic Acid	>10
Danicopan	>10
Atuliflapon	>10
Cintirorgon	>10
Uzansertib phosphate	>10
Golidocitinib	>10
SNDX-5613	>10
Tuxobertinib	>10
Delafloxacin meglumine	>10
Etomidate hydrochloride	>10
Capadenoson	>10
Upamostat	>10
RX-3117	>10
Ritanserin	>10
Iobenguane sulfate	>10
Larazotide acetate	>10
Indotecan	>10
Pictilisib dimethanesulfonate	>10
Eltoprazine hydrochloride	>10
Tenapanor	>10
Lemborexant	>10
Argatroban Monohydrate	>10
Eicosapentaenoic acid ethyl ester	>10
Mivacurium dichloride	>10
Tipepidine hydrochloride	>10
Acenocoumarol	>10
Laninamivir octanoate	>10
Oteseconazole	>10
Olinciguat	>10
2-Iminobiotin hydrobromide	>10
Centanafadine hydrochloride	>10
MK-7622	>10
Elenbecestat	>10
Avoralstat	>10
Tolebrutinib	>10
CC-90003	>10
SB-649868	>10
Seltorexant	>10
GSK163090	>10
Vabicaserin hydrochloride	>10
Tepilamide fumarate	>10
ITI-214	>10
Lorediplon	>10

Temanogrel	>10
S 3304	>10
LUT014	>10
Grapiprant	>10
Dexrazoxane	>10
DT2216	>10
Batabulin	>10
BIIB068	>10
Tirabrutinib hydrochloride	>10
Ningetinib Tosylate	>10
BNC105	>10
NVP-CGM097	>10
TAK-593	>10
Eliglustat hemitartrate	>10
XL092	>10
Bavdegalutamide	>10
OTX008	>10
PF-06291874	>10
Voreloxin hydrochloride	>10
Emeramide	>10
Aticaprant	>10
Mirodenafil dihydrochloride	>10
ALZ-801	>10
AG-636	>10
NGP555	>10
NP-G2-044	>10
Treprostinil	>10
Aluminum Hydroxide	>10
SHR0302	>10
Roluperidone	>10
Vamorolone	>10
Evenamide	>10
BTRX-335140	>10
LY518674	>10
Dilmapimod	>10
Siremadlin	>10
DPA-714	>10
Giredestrant	>10
RG7800	>10
DBPR108	>10
ATN-224	>10
Quarfloxin	>10
Olafertinib	>10
GSK256066	>10

Zatolmilast	>10
LY2940094	>10
KL1333	>10
IACS-13909	>10
AZD5423	>10
Tecadenoson	>10
Brensocatib	>10
AT-007	>10
BMS-986242	>10
Balovaptan	>10
AZD5305	>10
BAY-85-8501	>10
Simufilam dihydrochloride	>10
Telotristat	>10
HM-30181 mesylate monohydrate	>10
Iptacopan hydrochloride	>10
Retagliptin Phosphate	>10
Zamicastat	>10
Sorbinil	>10
Ralmitaront	>10
IAXO-102	>10
S55746	>10
JNJ-39758979	>10
Navtemadlin	>10
DSP-2230	>10
BI-671800	>10
PF-05221304	>10
Zegocractin	>10
Deslorelin acetate(57773-65-6 free base)	>10
Plecanatide	>10
Abarelix	>10
PMX 53 acetate(219639-75-5 free base)	>10
Sermorelin acetate	>10
Ensartinib hydrochloride	>10
Atrasentan	>10
JPH203	>10
T807	>10
LM11A-31 dihydrochloride	>10
LY2880070	>10
Elimusertib hydrochloride(1876467-74-1 free base)	>10
BGT226	>10
Parsaclisib	>10
Bozitinib	>10
JNJ-38877618	>10

AB-680	>10
LY2510924 acetate(1088715-84-7 free base)	>10
Inavolisib	>10
(2-Hydroxypropyl)-β-cyclodextrin	>10
Sisunatovir hydrochloride	>10
Morphothiadin	>10
VCH-916 free acid(1200133-34-1 free base)	>10
Mericitabine	>10
LHF-535	>10
Vebicorvir	>10
Elsulfavirine	>10
Bemnifosbuvir hemisulfate	>10
TMC647055 Choline Hydroxide Salt	>10
Pyrotinib dimaleate	>10
Estramustine	>10
1-[2,4-Dihydroxy-6-methoxy-3-(3-methyl-2-buten-1-yl)phenyl]-3-(4-hydroxyphenyl)-2-propen-1-one	>10
Castanospermine	>10
Hycanthone	>10
IDH-305	>10
Zolpidem tartrate	>10
Tirbanibulin Mesylate	>10
Hydroxychloroquine	>10
Dotinurad	>10
Dalbavancin	>10
Pipamperone	>10
Prazosin	>10
Rutin hydrate	>10
Ceftobiprole	>10
Guanabenz hydrochloride	>10
Ifenprodil	>10
Dexamethasone Phosphate disodium	>10
Solifenacin	>10
Azosemide	>10
Tacrine	>10
Midodrine	>10
Amitriptyline	>10
Testosterone decanoate	>10
Brilliant blue G-250	>10
Calcifediol monohydrate	>10
Betaxolol	>10
Nandrolone propionate	>10
Haloperidol decanoate	>10
JNJ-63576253	>10

Etripamil	>10
MMV390048	>10
JNJ-18038683	>10
Proxalutamide	>10
Pimonidazole	>10
EHP-101	>10
Ervogastat	>10
Xaliproden hydrochloride	>10
Aficamten	>10
(S)-Flurbiprofen	>10
Lotilaner	>10
AQ-13 dihydrochloride	>10
Ataciguat	>10
Danicamtiv	>10
AG-270	>10
NV-5138	>10
Bemnifosbuvir	>10
Trofinetide	>10
Icerguastat	>10
PQ912	>10
Nuclomedone	>10
GSK-626616	>10
PAP-1	>10
Tesmilifene fumarate	>10
KA2507	>10
Endovion	>10
Taminadenant	>10
CNDAC hydrochloride	>10
Incyclinide	2.5
GET73	>10
AZD9056 hydrochloride	>10
Reparixin L-lysine salt	>10
TQS	>10
DM4	>10
PCO371	>10
Clomethiazole	>10
Vercirnon	>10
P110δ-IN-1	>10
(-)-Carvone	>10
Darexaban	>10
Fenobam	>10
Talinolol	>10
Nirmatrelvir	>10
Dimebolin	>10

Tavapadon	>10
Ibutamoren	>10
Adenosine A1 receptor activator T62	>10
Praliciguat	>10
Anecortave Acetate	>10
Telratolimod	>10
GDC0575 monohydrochloride	>10
Nitroaspirin	>10
EF-5	>10
ALK inhibitor 1	>10
Elacestrant	>10
CPDA	>10
TAS4464 hydrochloride	>10
Fezagepras	>10
PF-5190457	>10
Filorexant	>10
Supinoxin	>10
KB-0742 dihydrochloride	>10
GFT505	>10
TAK-041	>10
Carotegrast methyl	>10
Razuprotafib	>10
Fosifloxuridine nafalbenamide	>10
Islatravir	>10
PF-00835231	>10
Fosciclopirox	>10
8-Chloroadenosine	>10
HM43239	>10
AZD-4818	>10
SGN-2FF	>10
Fadraciclib	>10
4SC-203	>10
MK-8033	>10
Talampanel	>10
Icenticaftor	>10
SAR407899 hydrochloride	>10
Ximelagatran	>10
LOXO-195	>10
Benzolamide	>10
MAP4343	>10
Mardepodect	>10
Linzagolix	>10
Firibastat	>10
Simurosertib	>10

Rimtuzalcap	>10
Allitinib	>10
OXOMEMAZINE	>10
Eliapixant	>10
CTB	>10
N-Methylpyrrolidone	>10
Pivagabine	>10
Nitroarginine	>10
L-Threonic acid magnesium salt	>10
Chlormethiazole hydrochloride	>10
Phenidone	>10
IMM-01	>10
Encequidar mesylate	>10
1-Octanol	>10
Dofequidar fumarate	>10
Cipargamin	>10
Amsilarotene	>10
1-piperoylpiperidine	>10
Epidermal Growth Factor Receptor Peptide Acetate	>10
BIO-11006 acetate salt (901117-03-1 free base)	>10
Avexitide	>10
Tirzepatide Acetate(2023788-19-2 free base)	>10
Glypromate acetate(32302-76-4 free base)	>10
COG 133 Acetate	>10
GnRH-I acetate	>10
Anti-Inflammatory Peptide 1 Acetate	>10
AC 187 Acetate	>10
Acetyl Angiotensinogen (1-14), porcine Acetate	>10
Tetrapeptide-30 Acetate	>10
Edotreotide	>10
Nemorexant	>10
AZD7624	>10
Esaxerenone	>10
PTC299	>10
Delgocitinib	>10
Ferric maltol	>10
Brimonidine	>10
Fexofenadine	>10
Tesevatinib	>10
FF-10101	>10
Subasumstat	>10
L-(+)-Arabinose	>10
Venadaparib	>10
Cotadutide acetate	>10

TIC10 Isomer	>10
Ganciclovir sodium	>10
Doxazosin	>10
trifarotene	>10
Selisistat S-enantiomer	>10
Pradefovir mesylate	>10
3,5-Diiodothyropropionic acid	>10
Naquotinib	>10
Cerlapirdine	>10
PXS-4728A	>10
ARRY 520 hydrochloride	>10
Samatasvir	>10
Cinitapride	>10
Apomine	>10
Diphenylcyclopropenone	>10
THIP	>10
AMP-945	>10
R1530	>10
Cedirogant	>10
Idalopirdine Hydrochloride	>10
Nastorazepide	>10
Dexelvucitabine	>10
Rebamipide mofetil	>10
Elacestrant dihydrochloride	>10
AP1189 acetate	>10
Ensifentrine	>10
BI-187004	>10
Senaparib	>10
AVN-101	>10
Nesolicaftor	>10
Myristyl nicotinate	>10
Crolibulin	>10
Fenpyroximate	>10
NG-Nitroarginine methyl ester	>10
TRV-120027 TFA	>10
Aspartyl-alanyl-diketopiperazine	>10
Se-Methylselenocysteine	>10
DL-Tryptophan	>10
Paltusotine	>10
DS-7423	>10
CP-609754	>10
NRC-2694	>10
TL-895	>10
INE963	>10

VX-150	>10
Belzutifan	>10
CC-90001	>10
DS-1971a	>10
Enpatoran	>10
Methylprednisolone Aceponate	>10
JNJ-37822681 dihydrochloride	>10
Linaprazan	>10
KD-026	>10
AZD 4017	>10
Pivanex	>10
δ-Tocotrienol	>10
Eclitasertib	>10
LY2922470	>10
Fenpropidin	>10
Cimicoxib	>10
Valecobulin hydrochloride	>10
Sulindac sulfone	>10
Rineterkib	>10
suvn-911	>10
Bocidelpar	>10
BMS-986176	>10
Tetradecylthioacetic acid	>10
Enavogliflozin	>10
PGLa acetate	>10
Lactisole	>10
Boscalid	>10
Mezigdomide	>10
Elinzanetant	>10
Espindolol	>10
Gepirone	>10
Leteprinim	>10
MDR-1339	>10
(±)-Lisofylline	>10
Sorivudine	>10
JNJ-37822681	>10
Apararenone	>10
Drometrizole Trisiloxane	>10
Aplidine	>10
α-Cyclodextrin	>10
ARRY-382	>10
LMP744 hydrochloride	>10
Ipatasertib dihydrochloride	>10
Opiranserin hydrochloride	>10

Tradipitant	>10
Sivopixant	>10
Ormetoprim	>10
JNJ-67856633	>10
Zandelisib	>10
HKI-357	>10
Avosentan	>10
Terevalefim	>10
Afimetoran	>10
Atrasentan hydrochloride	>10
Pirtobrutinib	>10
Octahydroaminoacridine succinate	>10
Brepocitinib	>10
ARV-471	>10
AZD-1656	>10
L-NMMA acetate	>10
Erteberel	>10
Atuveciclib Racemate	>10
MK-8353	>10
TPX-0046	>10
Sunitinib	>10
Montelukast	>10
Motixafortide TFA(664334-36-5,Free)	>10
Monomethyl fumarate	>10
Tetrahydrocannabivarin	>10
Amodiaquine	>10
Cinacalcet	>10
Chloroquine	>10
Decoglurant	>10
Felypressin	>10
Nedisertib	>10
Saxagliptin	>10
Gusacitinib	>10
Abrocitinib	>10
LXH254	>10
Amantadine	>10
Calcitonin (salmon) Acetate(47931-85-1(free base))	>10
Kisspeptin-10, rat acetate(478507-53-8 free base)	>10
Exendin-4 acetate	>10
Scyllo-Inositol	>10
Trilaciclib hydrochloride	>10
L-Lysine hydrochloride	>10
Laninamivir	>10
(Arg)9 TFA (143413-47-2 free base)	>10

Plerixafor octahydrochloride	>10
Lithium carbonate	>10
Amifostine trihydrate	>10
Etelcalcetide hydrochloride	>10
Phytic acid sodium salt	>10
Piperaquine tetraphosphate tetrahydrate	>10
Disodium succinate	>10
Ibandronate sodium monohydrate	>10
DL-Buthionine-(S,R)-sulfoximine	>10
Folinic Acid Calcium Salt Pentahydrate	>10

Appendix Table S2. Summary of data collection and refinement statistics.

Target protein	SA_{CpfC}
Wavelength	1
Resolution range	28.5-2.185 (2.263-2.185)
Space group	P1
Until cell	51.161, 54.45, 60.434, 69.73, 84.848, 90.072
Total reflections	106619 (15631)
Unique reflections	30538 (3026)
Multiplicity	3.5 (3.5)
Completeness (%)	96.72 (95.97)
Mean I/sigma(I)	8.2 (2.5)
Wilson B-factor	21.17
R-merge	0.087 (0.474)
R-meas	0.103 (0.558)
R-pim	0.054 (0.292)
CC1/2	0.996 (0.899)
CC*	0.998 (0.947)
Reflections used in refinement	30518 (3018)
Reflections used for R-free	1999 (198)
R-work	0.2009 (0.2509)
R-free	0.2600 (0.2877)
CC (work)	0.870
CC (free)	0.799
No. atoms	
Number of non-hydrogen atoms	5162
macromolecules	4906
ligands	2
solvent	254
Protein residues	608
RMS (bonds)	0.008
RMS (angles)	1.00
Ramachandran favored (%)	96.36
Ramachandran allowed (%)	3.48
Ramachandran outliers (%)	0.17
Rotamer outlier (%)	0.75
Clashscore	7.07
Average B-factor	30.64
Macromolecules	30.80
Ligands	58.28
Solvent	27.35
PDB ID	9VI5

Appendix Table S3. The primer sequences used in this study.

Oligonucleotides (5'-3')	Description
TCTTGCCAGCTTTCCCCTTC	*pyJ335* verification-forward
GGTGTAGAGCAGCCTACATTGTATTG	*pyJ335* verification-reverse
ACGACGTTGTAAAACGACGGCCAGTGATATC ATGACTAAAAAAATGGGGTTATTAGTTATGG CTTATGG	Amplification of the *cpfC* -forward
TTCACACAGGAAACAGCTATGACCACTCGAG TTAAAATATAGACTTGATTTCATCAACAA	Amplification of the *cpfC* -reverse
TCTTGCCAGCTTTCCCCTTC	*pyJ335-cpfC* verification-forward
GGACGGTAGTAGTTAGCGCC	*pyJ335*-cpfC verification-reverse
CGCCAGGGTTTTCCCAGTCACGAC	Bacterial colony identification-forward
AGCGGATAACAATTTCACACAGGA	Bacterial colony identification-reverse
CTCTTCGCTATTACGCCAGCT	*cpfC* sequencing-forward
GTGAGTTAGCTCACTCATTAGGC	*cpfC* sequencing-reverse
