# Supplementary material for: SHP-1 agonist SC-43 limits methicillin-resistant Staphylococcus aureus infection through inhibition of heme biosynthesis
Source: EMBO Mol Med. 2026 Apr 10;18(5):1990–2005. doi: 10.1038/s44321-026-00418-4 (PMC13179323; doi:10.1038/s44321-026-00418-4)
Supplement: Supplementary file 11 — Source data Fig. 6 [file 44321_2026_418_MOESM11_ESM.zip › Figure 6/Data-Figure 6B/Notes on the grouping of the mouse experiments.docx]

**Notes on the grouping of the mouse experiments:**

No infection group (1-1, 1-2, 1-3, 1-4, 1-5)

Control (2-1, 2-2, 2-3, 2-4, 2-5)

SC-43 treatment (3-1, 3-2, 3-3, 3-4, 3-5)

Mupirocin treatment (4-1, 4-2, 4-3, 4-4, 4-5)
