# Supplementary figures and images for: SHP-1 agonist SC-43 limits methicillin-resistant Staphylococcus aureus infection through inhibition of heme biosynthesis (part 2 of 2)
Source: EMBO Mol Med. 2026 Apr 10;18(5):1990–2005. doi: 10.1038/s44321-026-00418-4 (PMC13179323; doi:10.1038/s44321-026-00418-4)

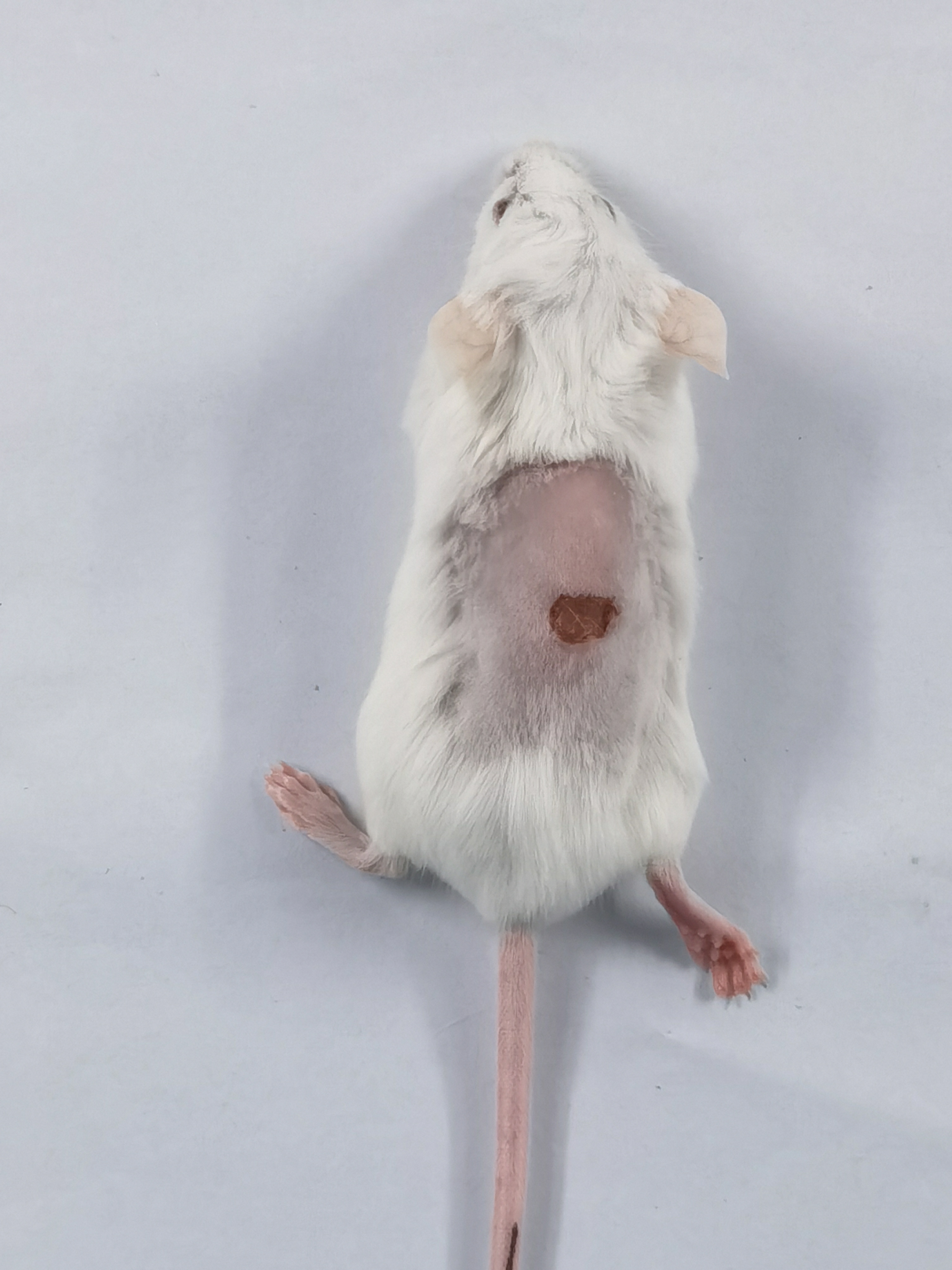

Supplement: Supplementary file 11 — Source data Fig. 6 [file 44321_2026_418_MOESM11_ESM.zip › Figure 6/Data-Figure 6B/Day 1/2-1.jpg]

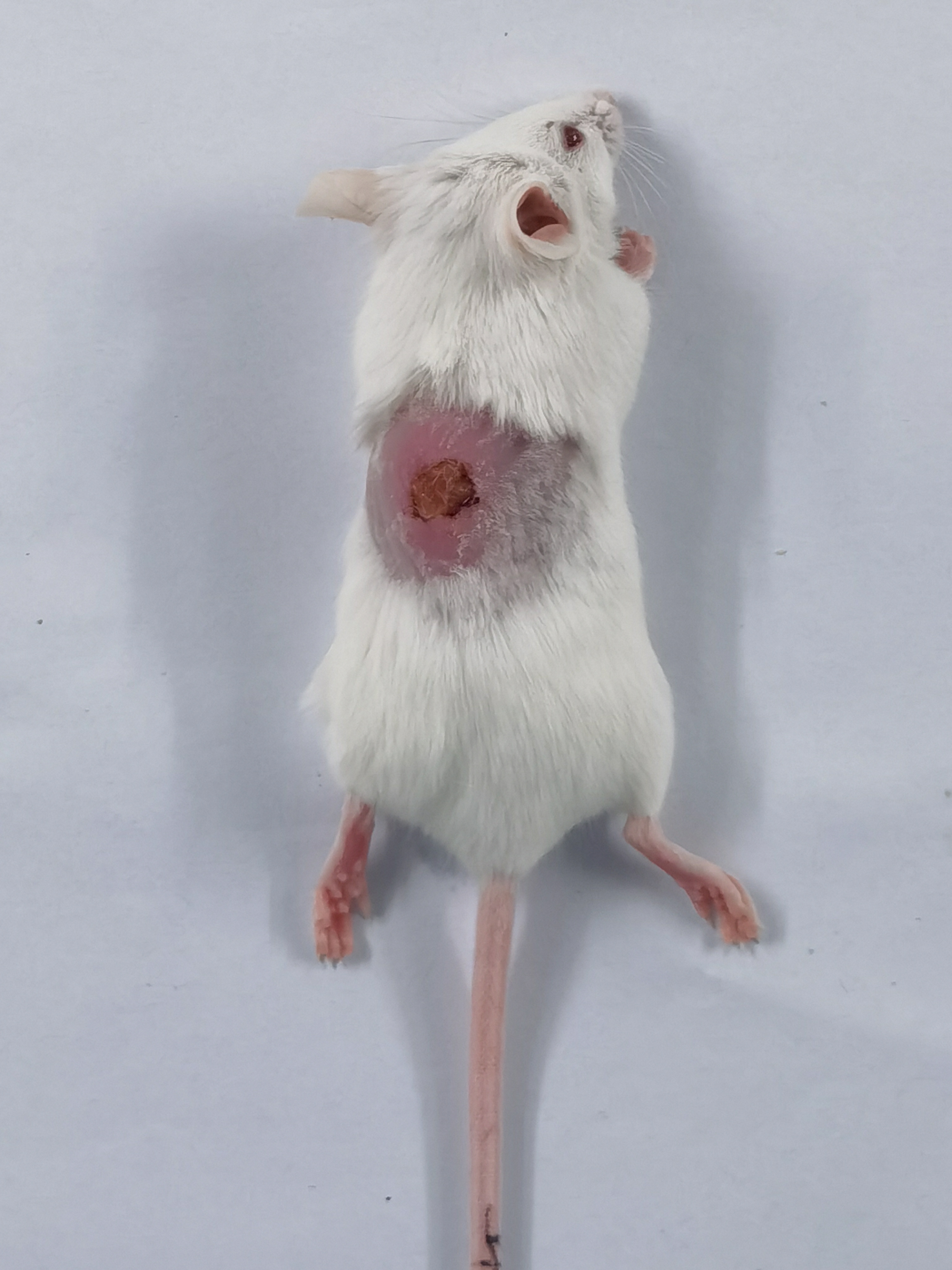

Supplement: Supplementary file 11 — Source data Fig. 6 [file 44321_2026_418_MOESM11_ESM.zip › Figure 6/Data-Figure 6B/Day 1/4-5.jpg]

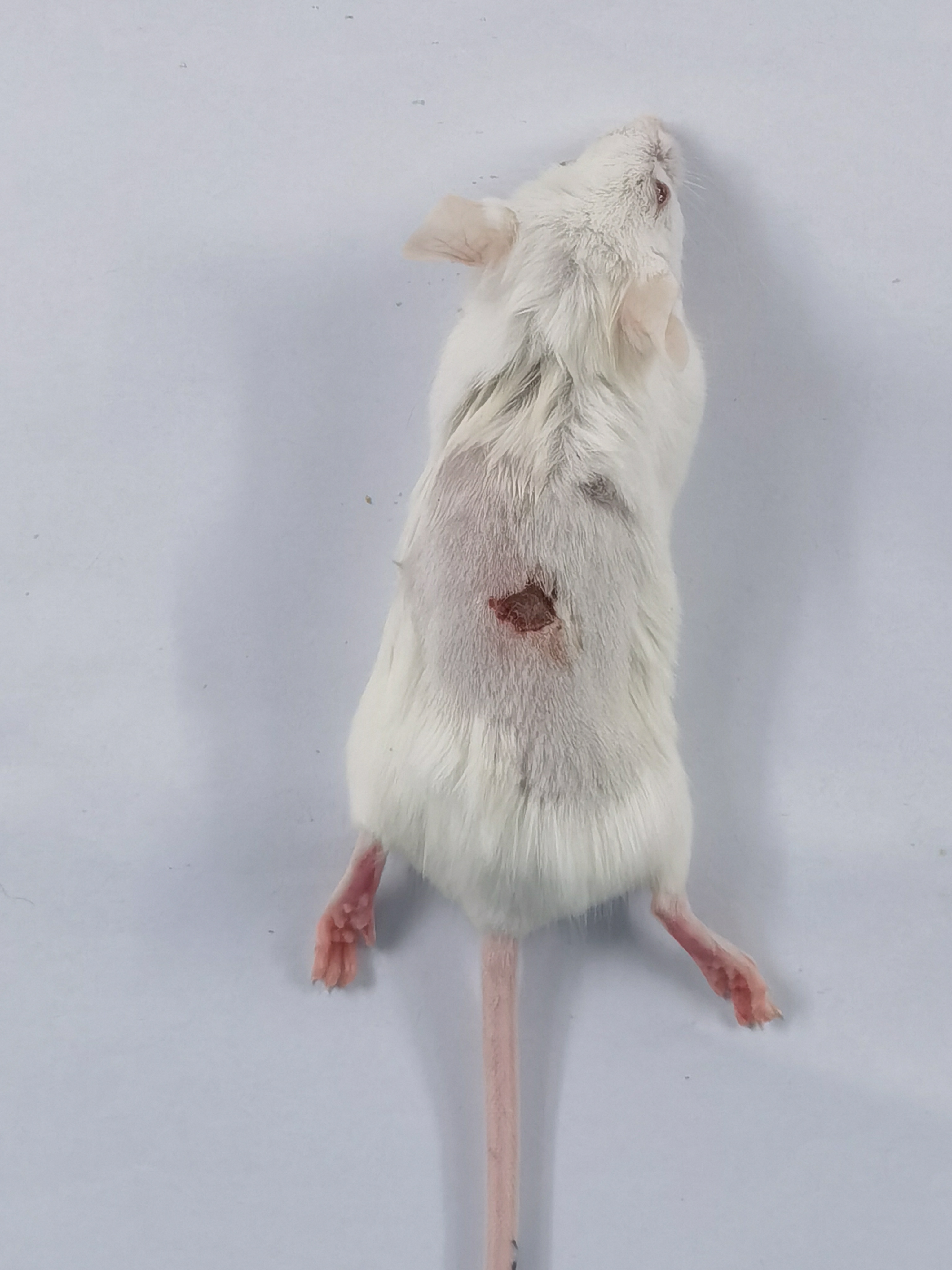

Supplement: Supplementary file 11 — Source data Fig. 6 [file 44321_2026_418_MOESM11_ESM.zip › Figure 6/Data-Figure 6B/Day 1/2-3.jpg]

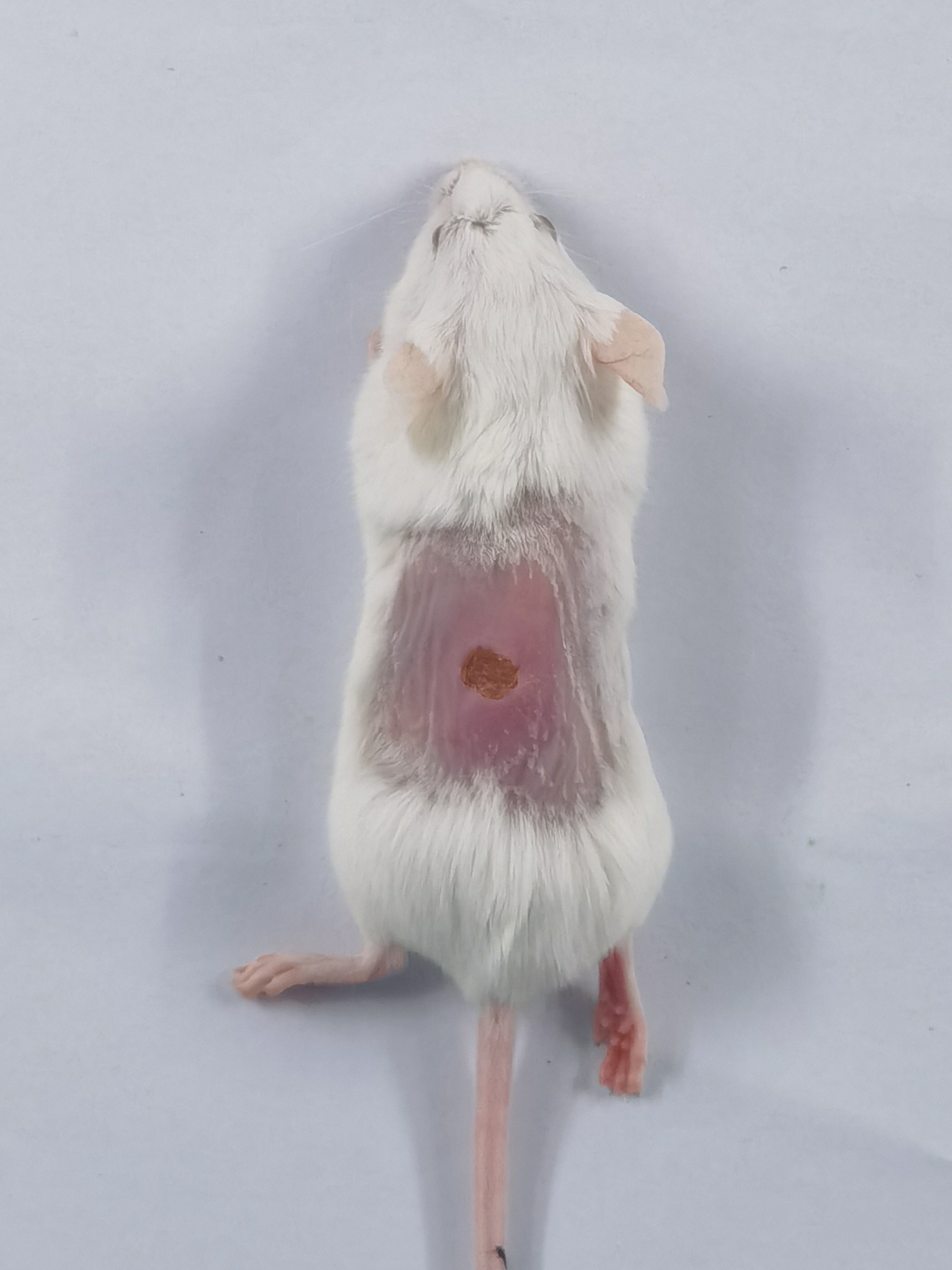

Supplement: Supplementary file 11 — Source data Fig. 6 [file 44321_2026_418_MOESM11_ESM.zip › Figure 6/Data-Figure 6B/Day 1/2-2.jpg]

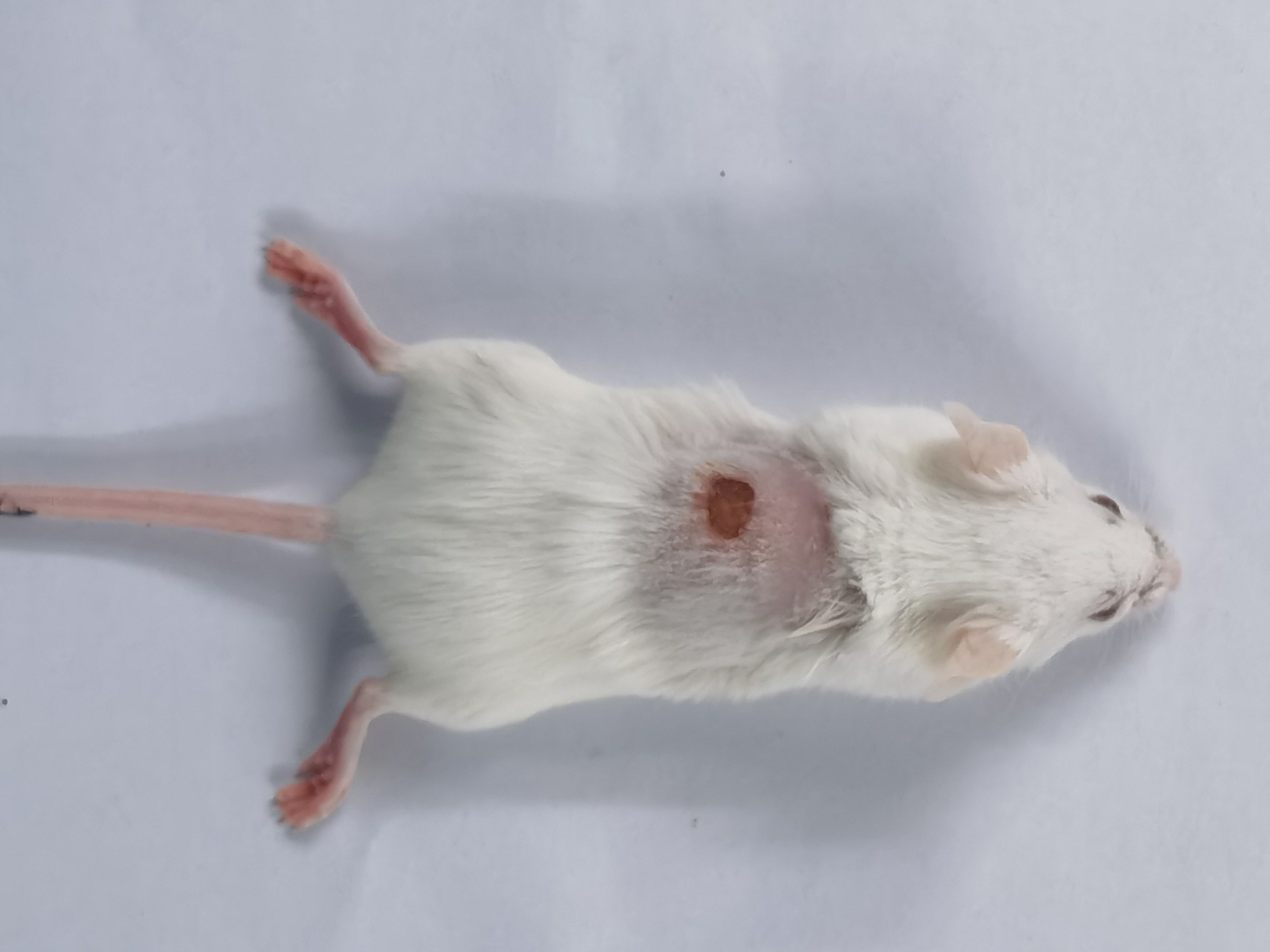

Supplement: Supplementary file 11 — Source data Fig. 6 [file 44321_2026_418_MOESM11_ESM.zip › Figure 6/Data-Figure 6B/Day 1/4-4.jpg]

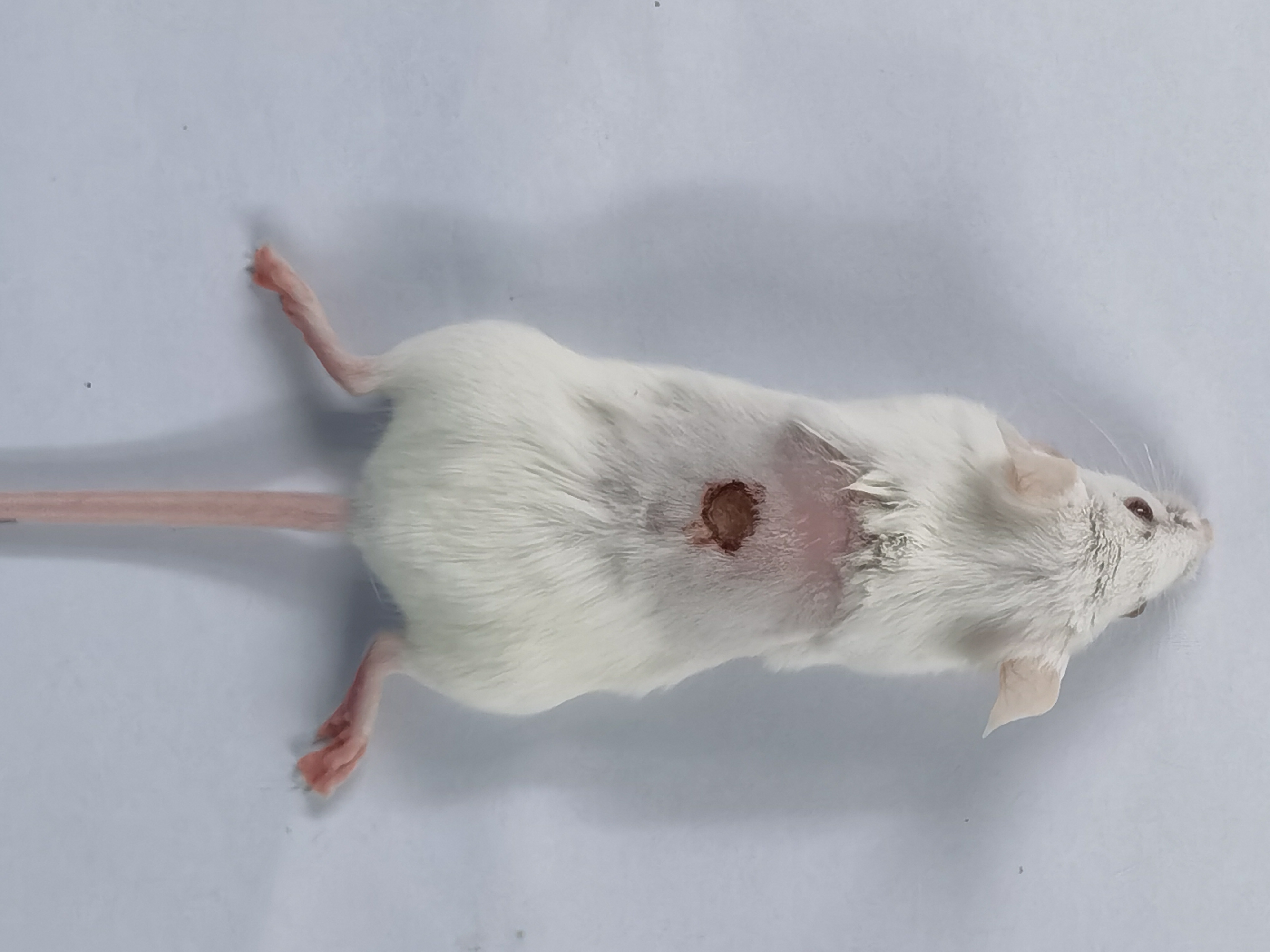

Supplement: Supplementary file 11 — Source data Fig. 6 [file 44321_2026_418_MOESM11_ESM.zip › Figure 6/Data-Figure 6B/Day 1/4-1.jpg]

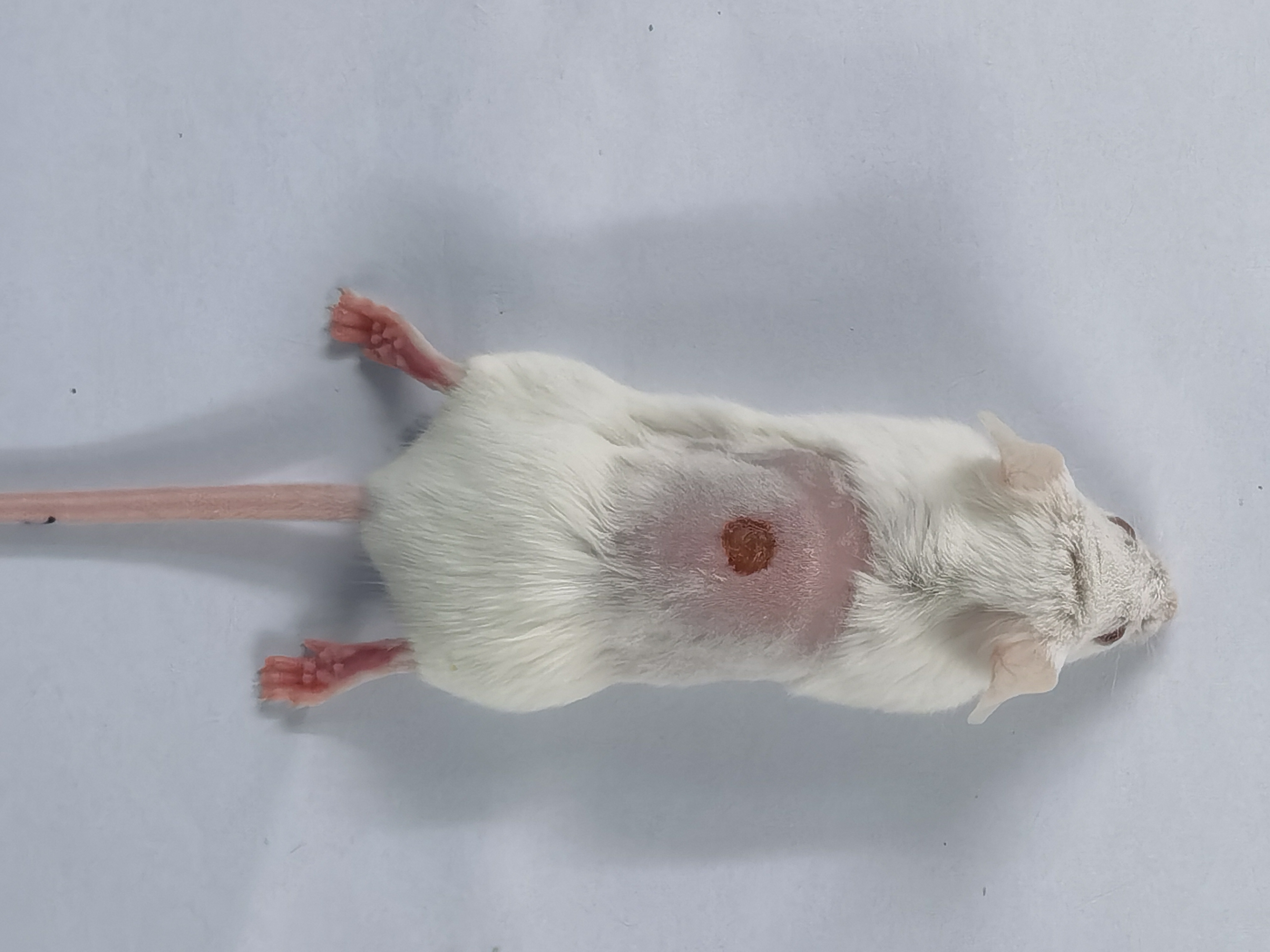

Supplement: Supplementary file 11 — Source data Fig. 6 [file 44321_2026_418_MOESM11_ESM.zip › Figure 6/Data-Figure 6B/Day 1/4-3.jpg]

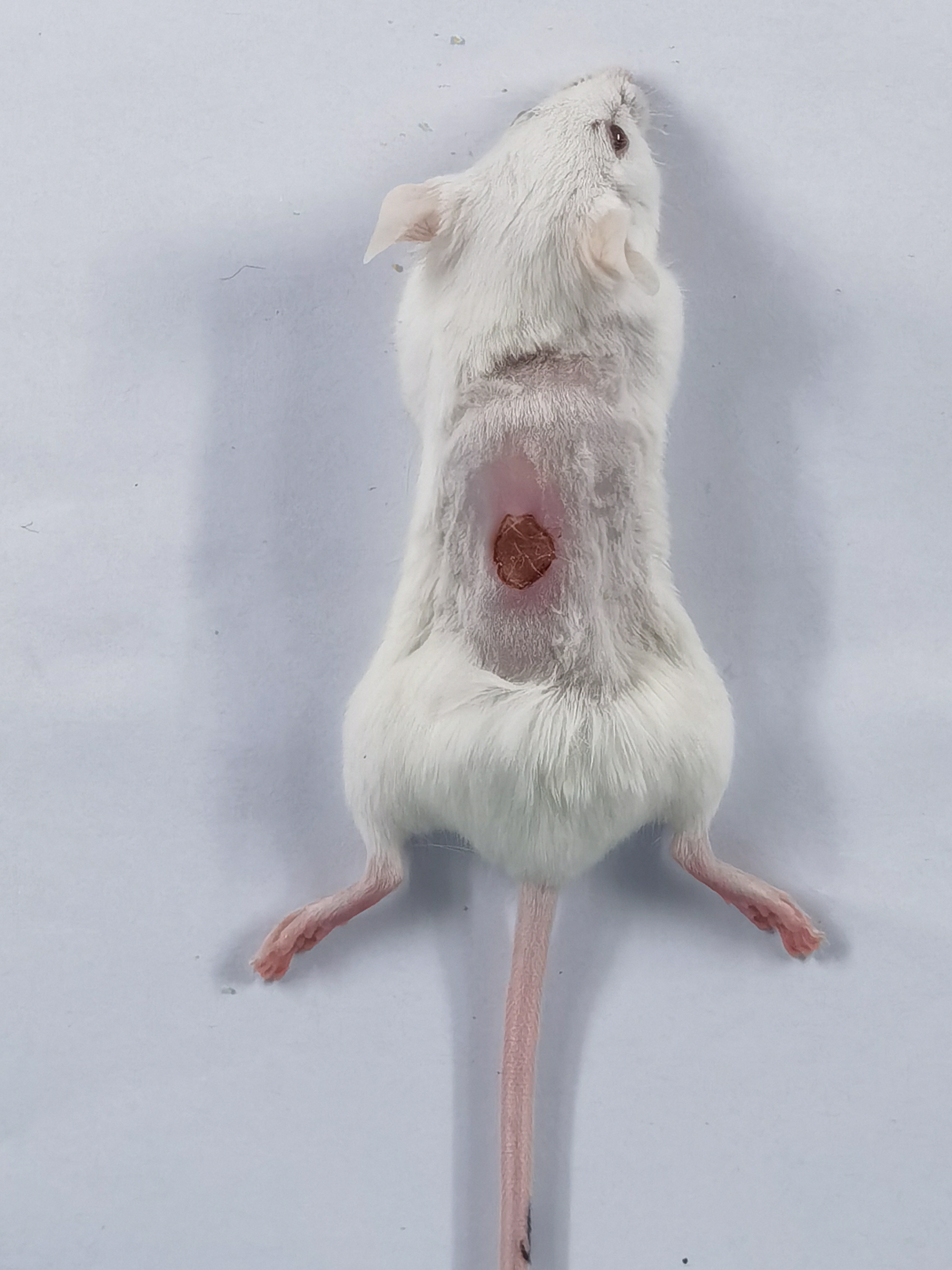

Supplement: Supplementary file 11 — Source data Fig. 6 [file 44321_2026_418_MOESM11_ESM.zip › Figure 6/Data-Figure 6B/Day 1/2-5.jpg]

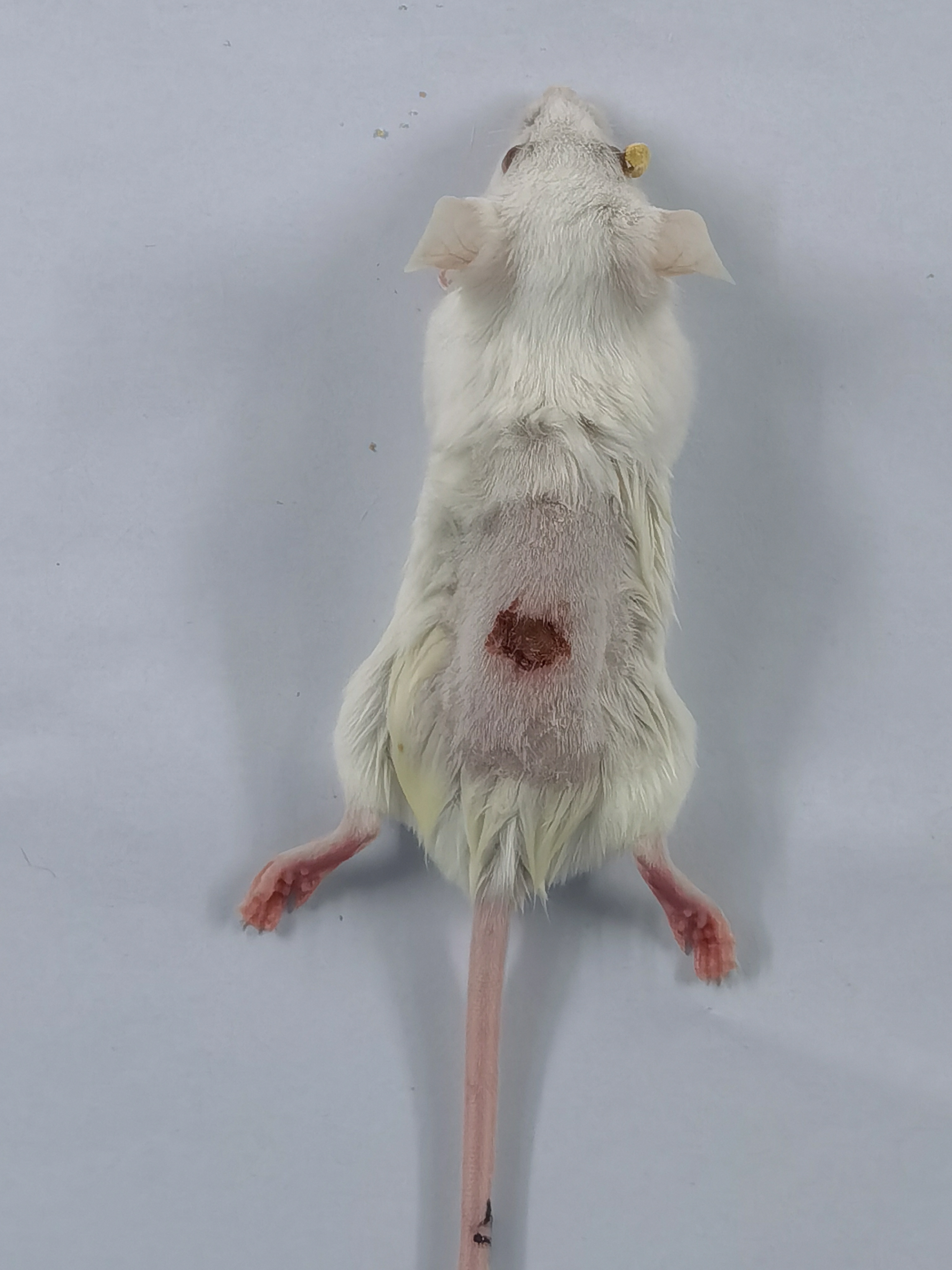

Supplement: Supplementary file 11 — Source data Fig. 6 [file 44321_2026_418_MOESM11_ESM.zip › Figure 6/Data-Figure 6B/Day 1/2-4.jpg]

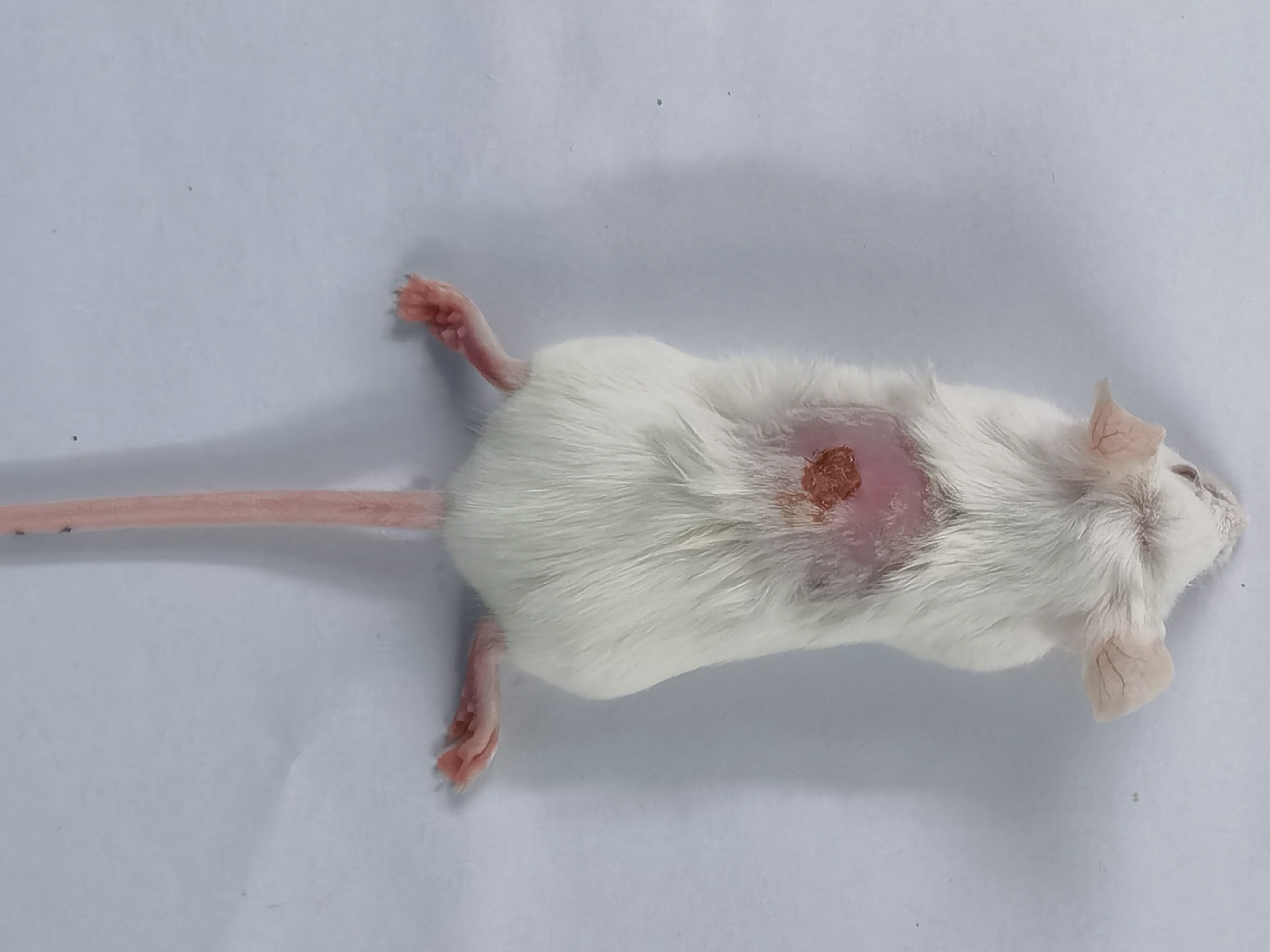

Supplement: Supplementary file 11 — Source data Fig. 6 [file 44321_2026_418_MOESM11_ESM.zip › Figure 6/Data-Figure 6B/Day 1/4-2.jpg]

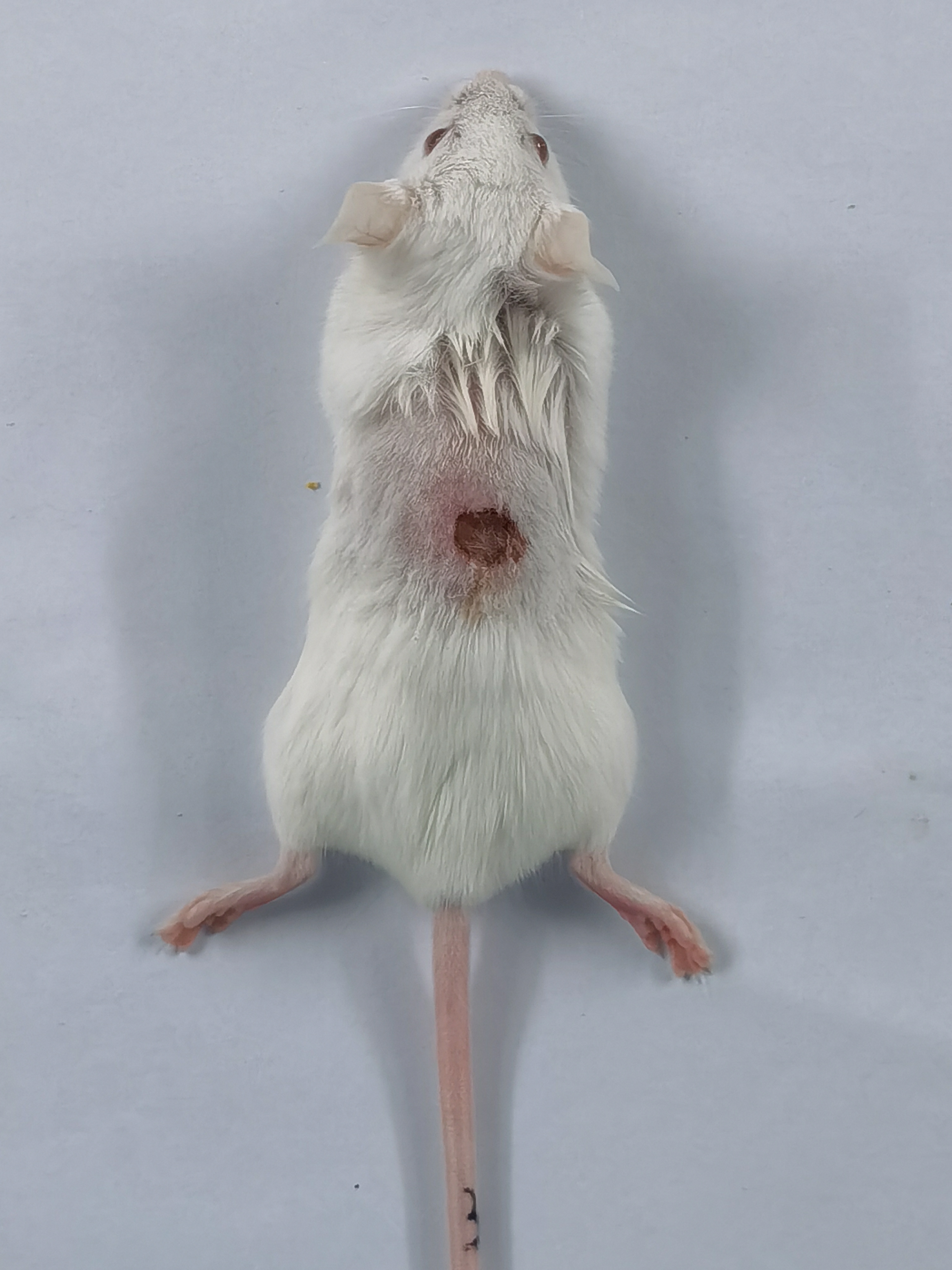

Supplement: Supplementary file 11 — Source data Fig. 6 [file 44321_2026_418_MOESM11_ESM.zip › Figure 6/Data-Figure 6B/Day 1/3-3.jpg]

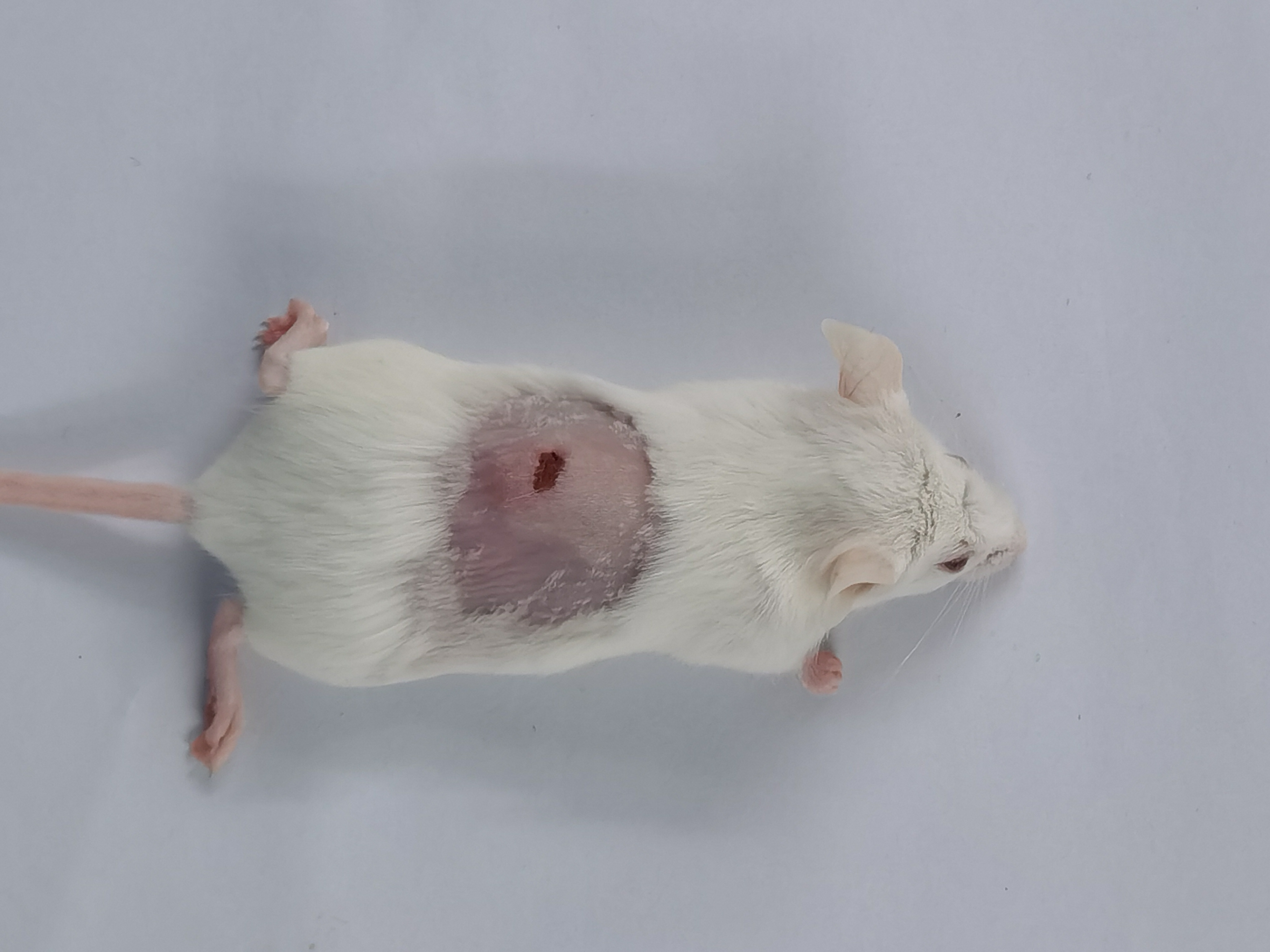

Supplement: Supplementary file 11 — Source data Fig. 6 [file 44321_2026_418_MOESM11_ESM.zip › Figure 6/Data-Figure 6B/Day 1/1-1.jpg]

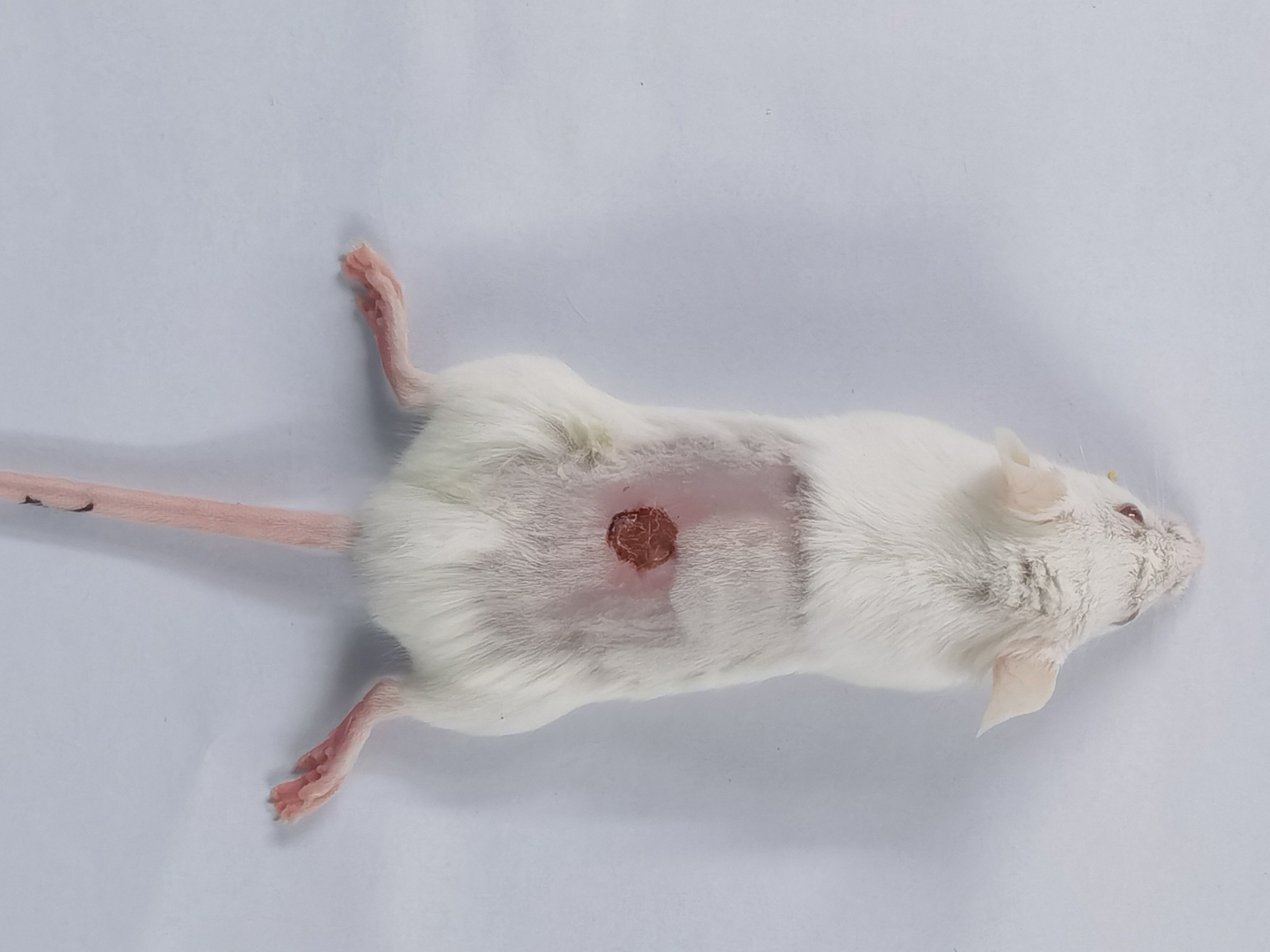

Supplement: Supplementary file 11 — Source data Fig. 6 [file 44321_2026_418_MOESM11_ESM.zip › Figure 6/Data-Figure 6B/Day 1/3-2.jpg]

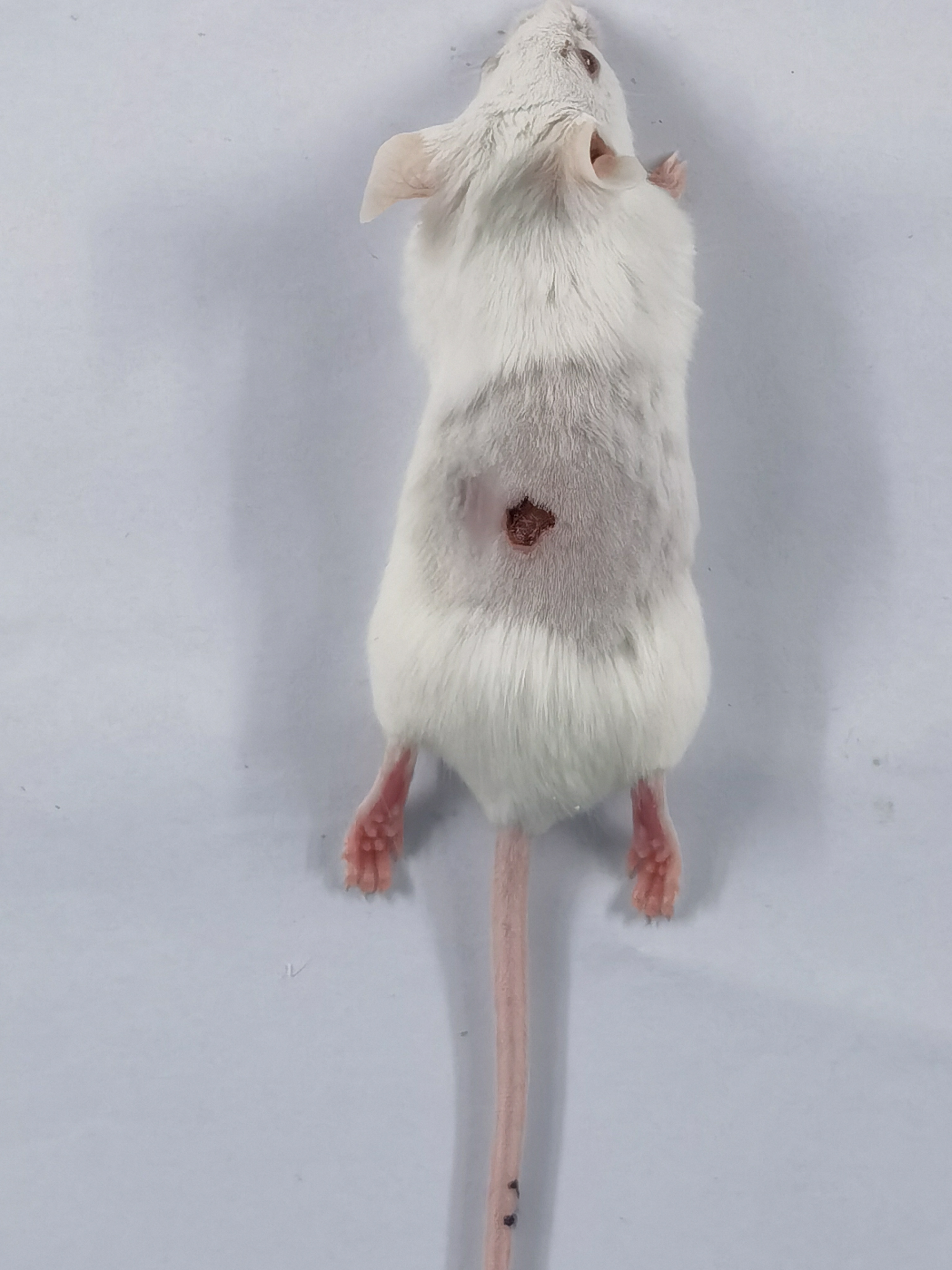

Supplement: Supplementary file 11 — Source data Fig. 6 [file 44321_2026_418_MOESM11_ESM.zip › Figure 6/Data-Figure 6B/Day 1/1-2.jpg]

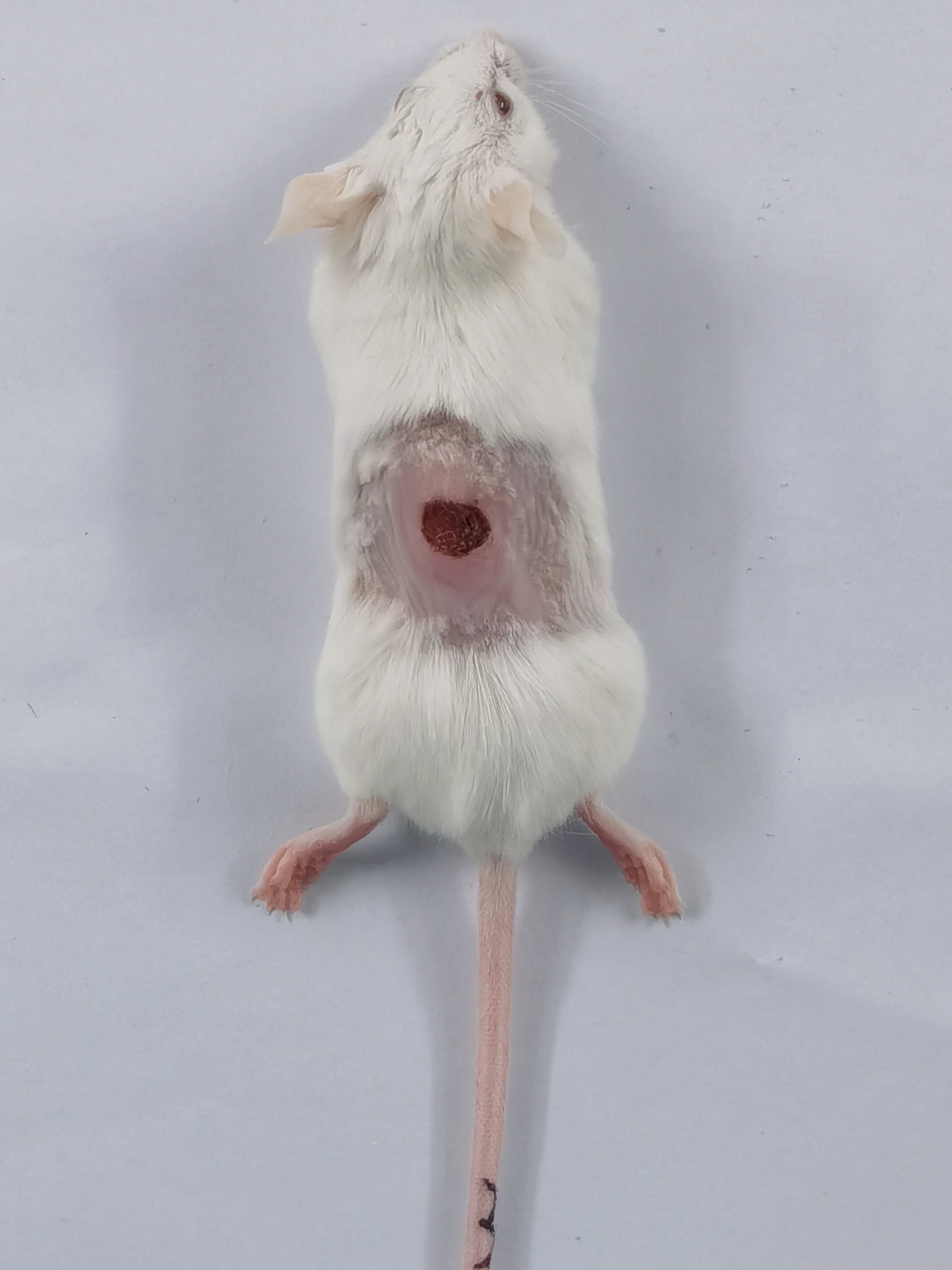

Supplement: Supplementary file 11 — Source data Fig. 6 [file 44321_2026_418_MOESM11_ESM.zip › Figure 6/Data-Figure 6B/Day 1/1-3.jpg]

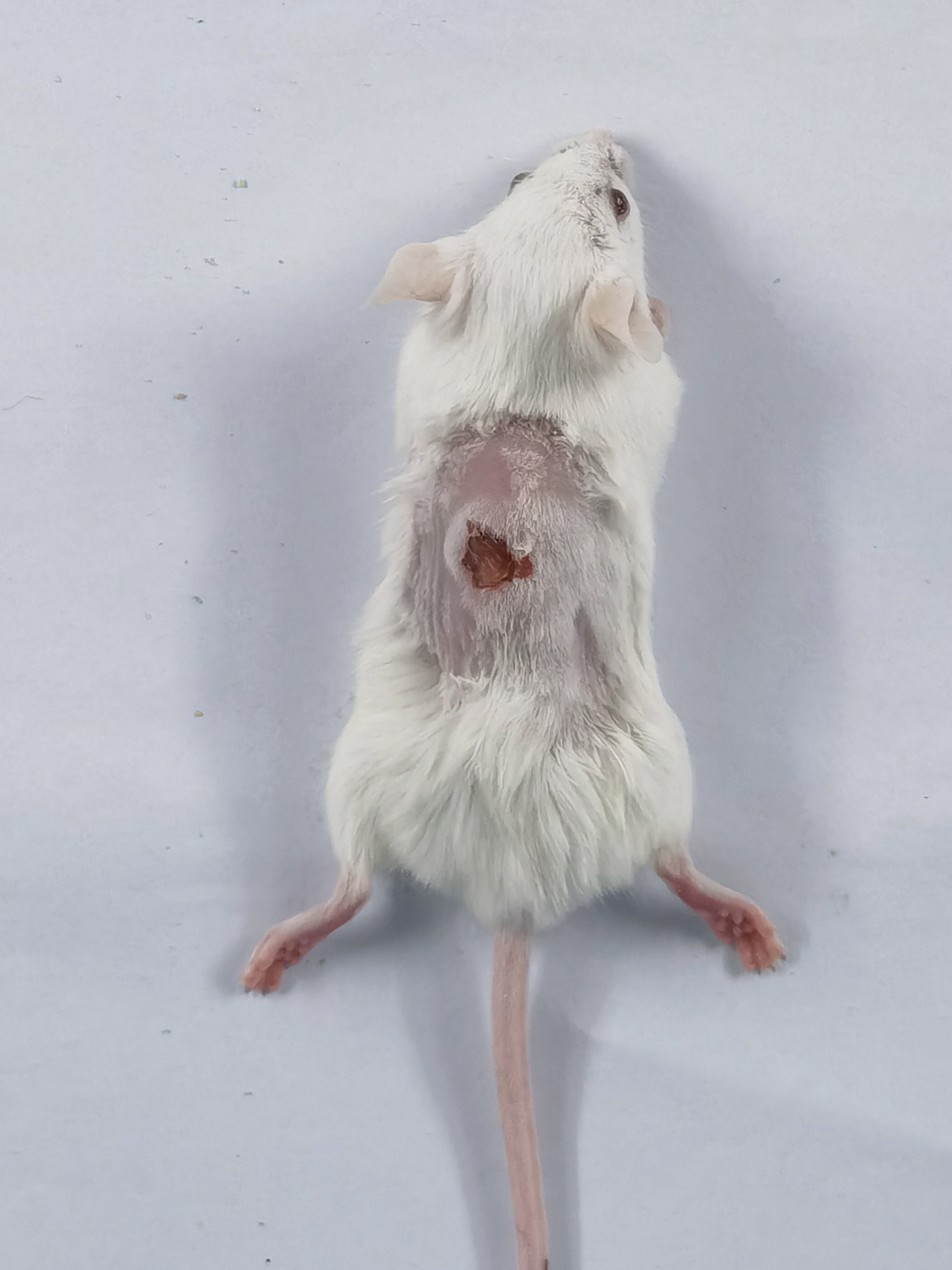

Supplement: Supplementary file 11 — Source data Fig. 6 [file 44321_2026_418_MOESM11_ESM.zip › Figure 6/Data-Figure 6B/Day 1/3-1.jpg]

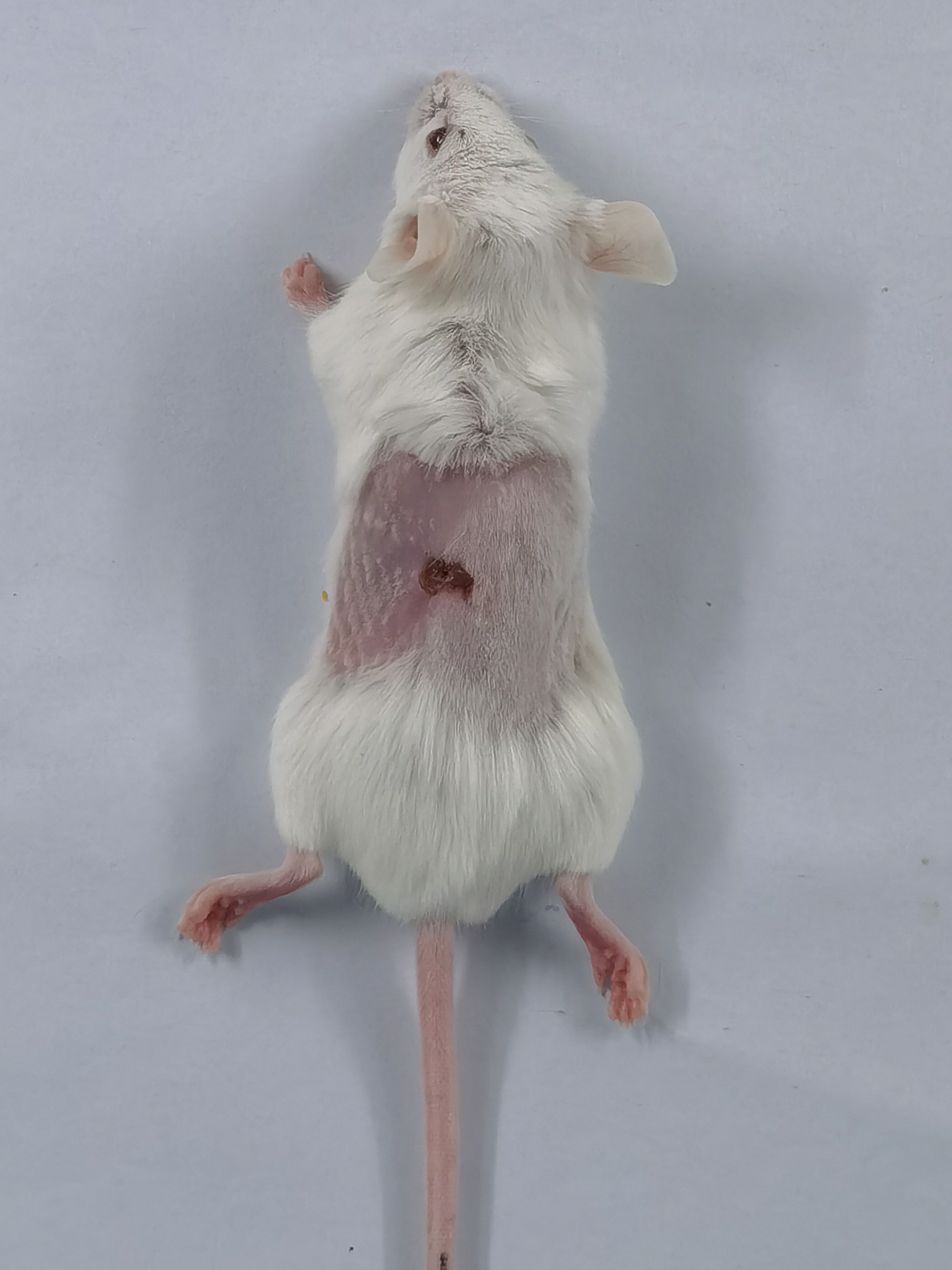

Supplement: Supplementary file 11 — Source data Fig. 6 [file 44321_2026_418_MOESM11_ESM.zip › Figure 6/Data-Figure 6B/Day 1/3-5.jpg]

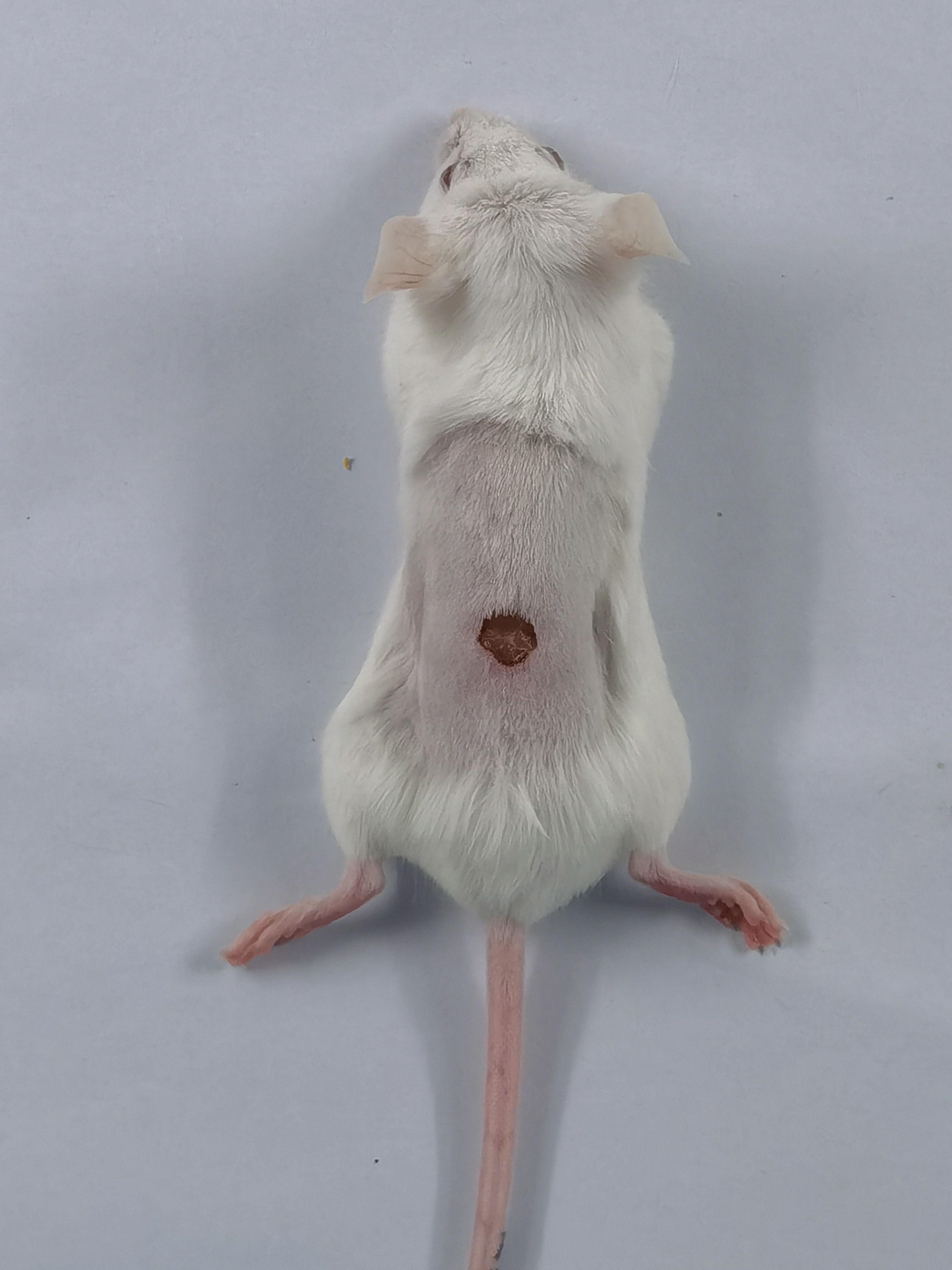

Supplement: Supplementary file 11 — Source data Fig. 6 [file 44321_2026_418_MOESM11_ESM.zip › Figure 6/Data-Figure 6B/Day 1/3-4.jpg]

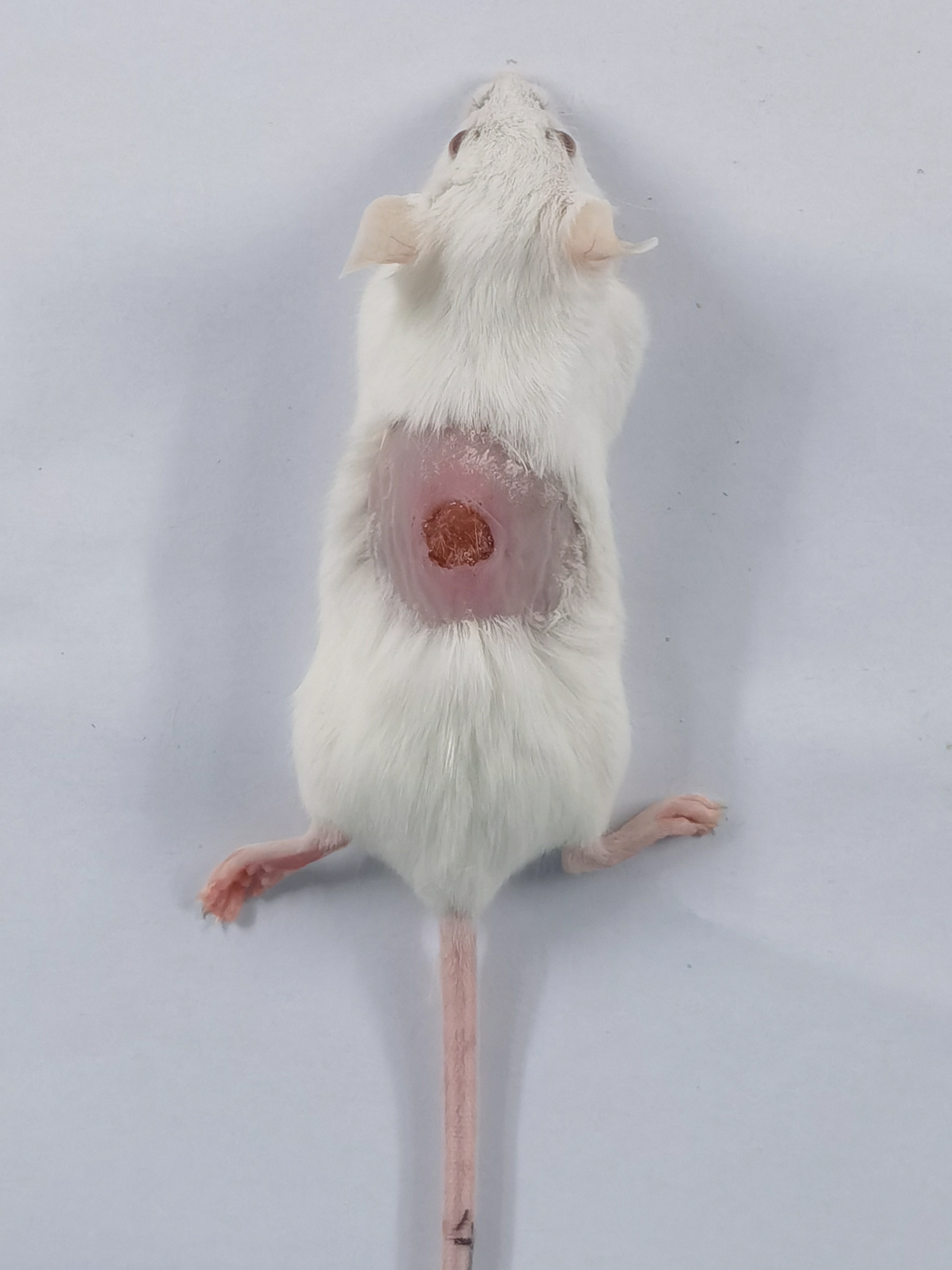

Supplement: Supplementary file 11 — Source data Fig. 6 [file 44321_2026_418_MOESM11_ESM.zip › Figure 6/Data-Figure 6B/Day 1/1-4.jpg]

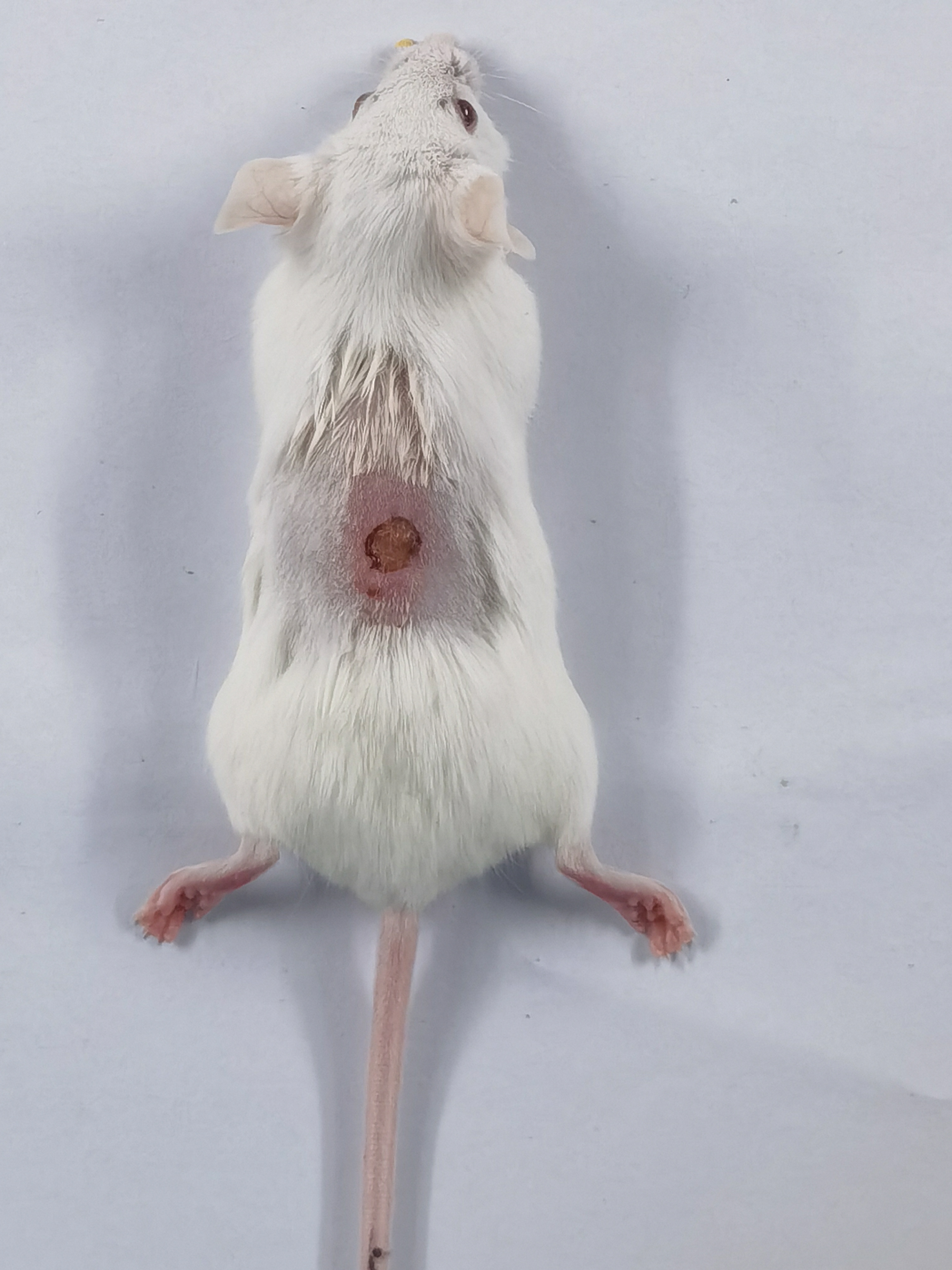

Supplement: Supplementary file 11 — Source data Fig. 6 [file 44321_2026_418_MOESM11_ESM.zip › Figure 6/Data-Figure 6B/Day 1/1-5.jpg]

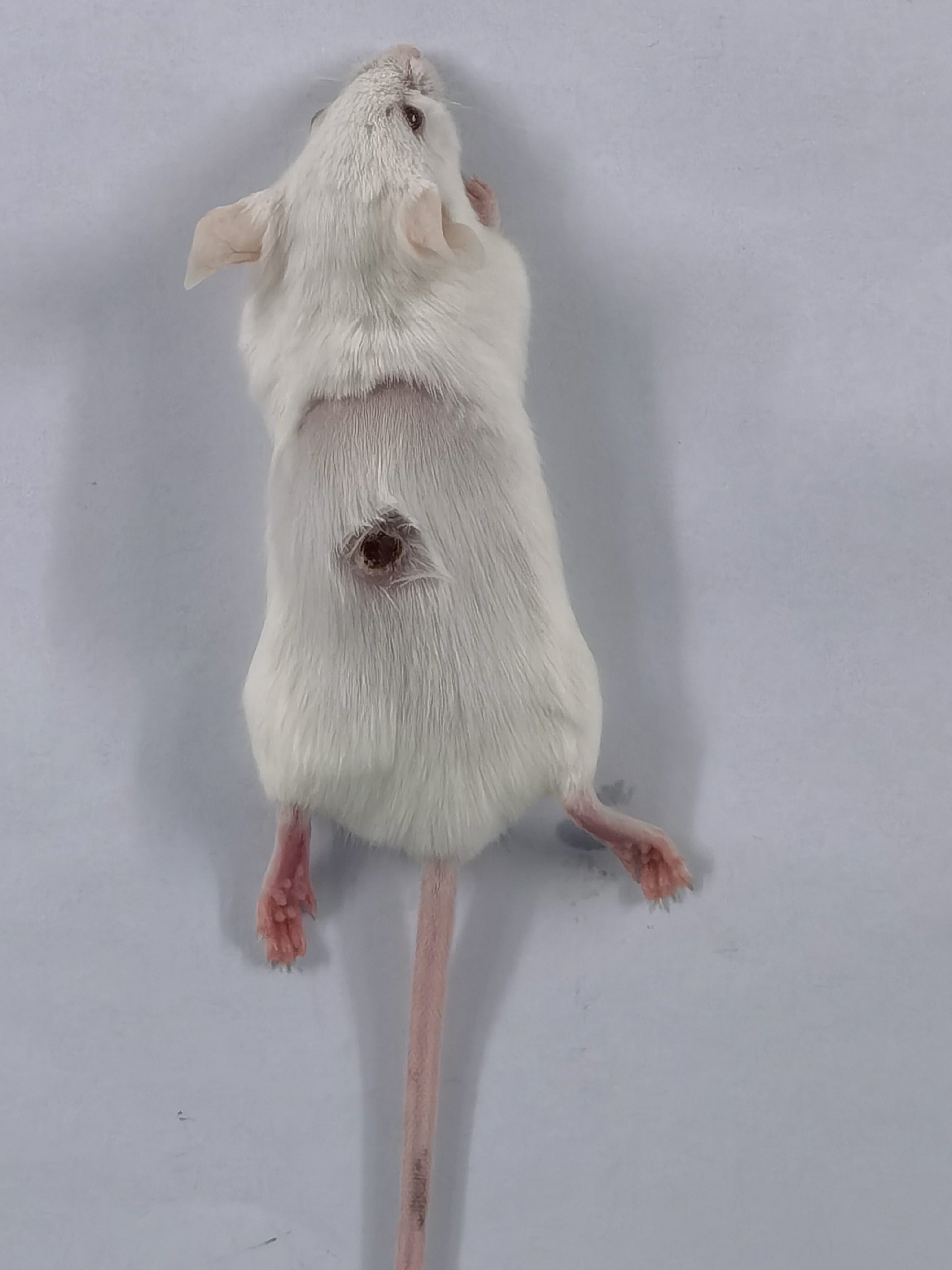

Supplement: Supplementary file 11 — Source data Fig. 6 [file 44321_2026_418_MOESM11_ESM.zip › Figure 6/Data-Figure 6B/Day 7/2-1.jpg]

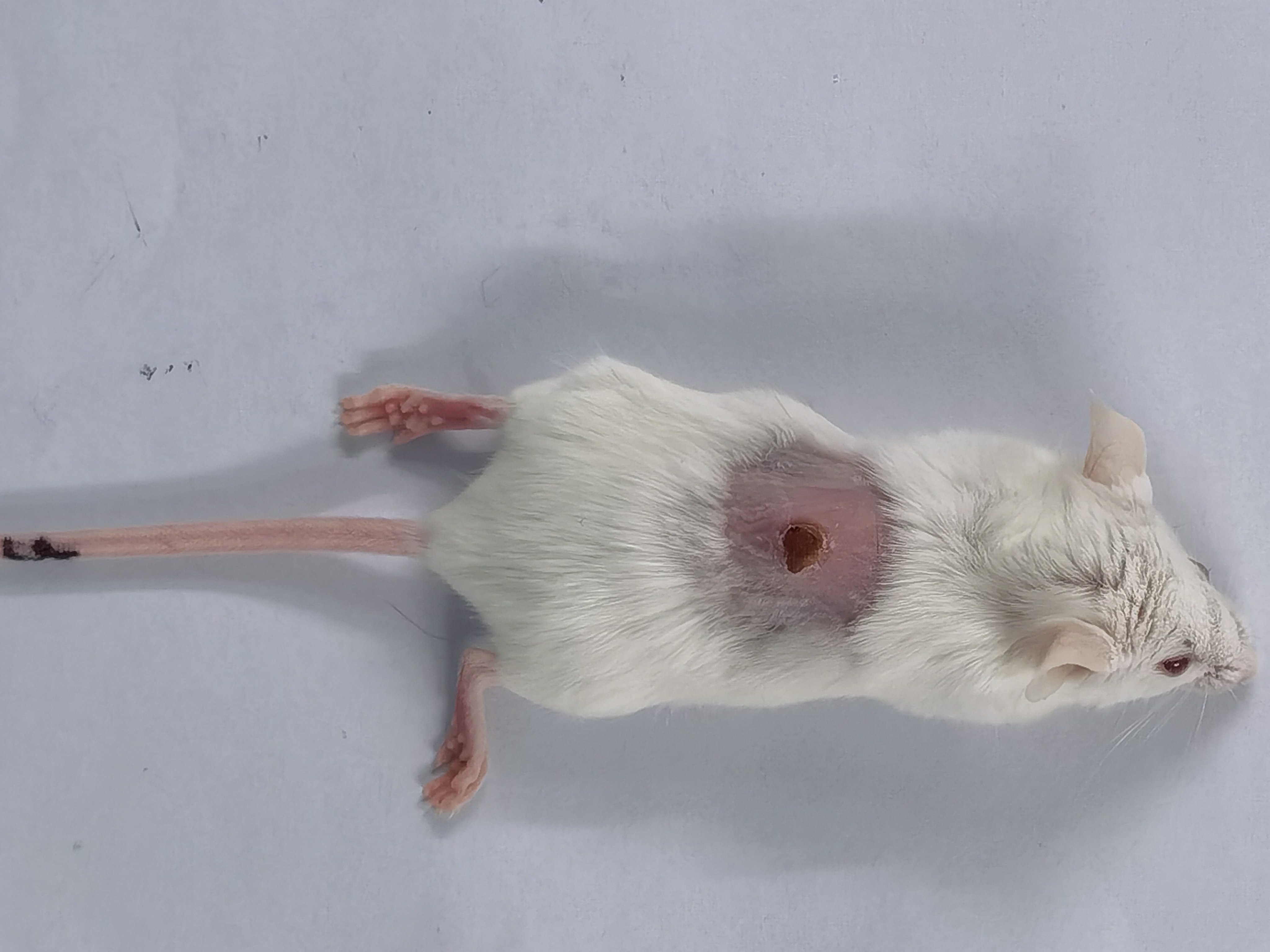

Supplement: Supplementary file 11 — Source data Fig. 6 [file 44321_2026_418_MOESM11_ESM.zip › Figure 6/Data-Figure 6B/Day 7/4-5.jpg]

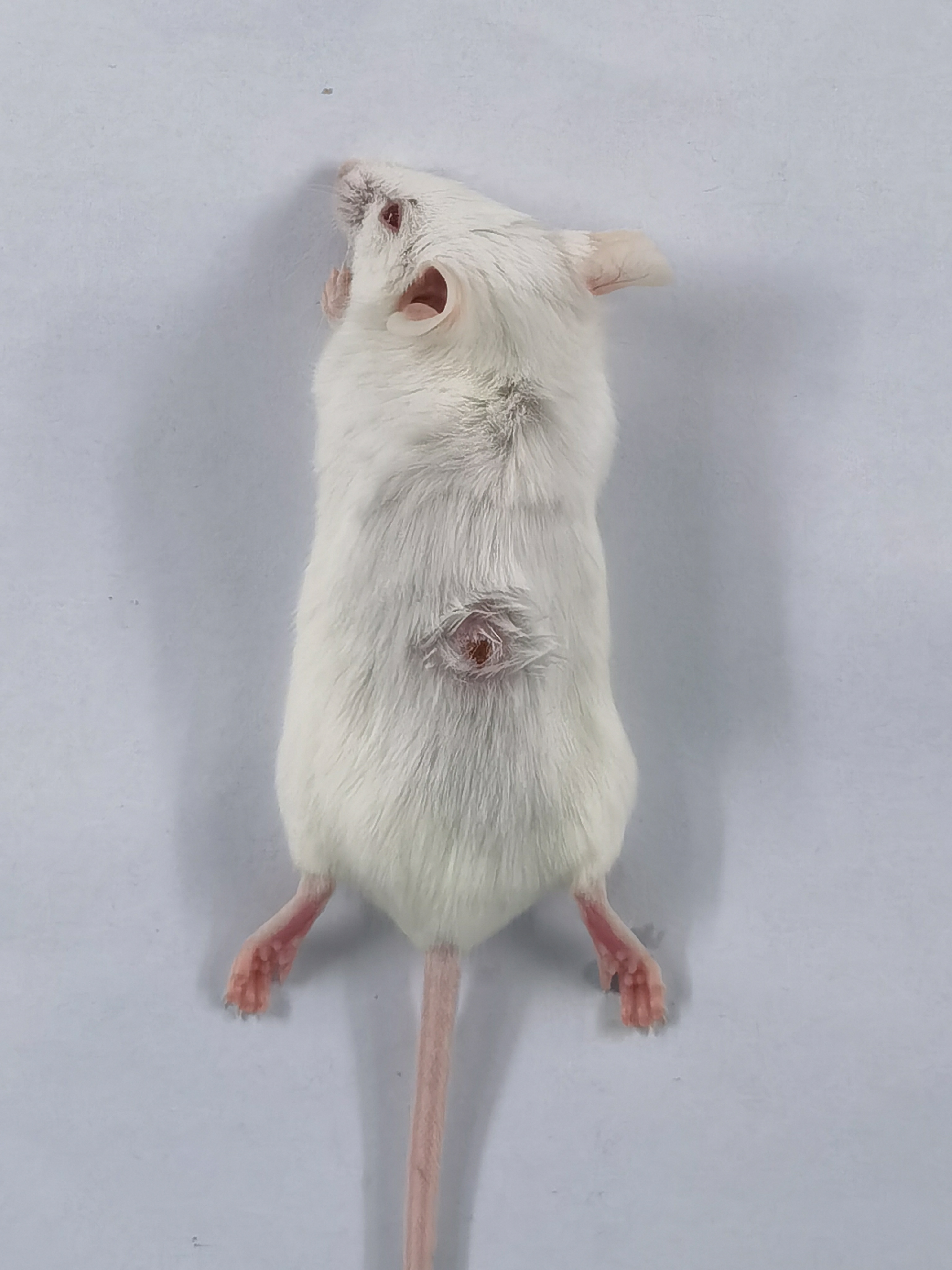

Supplement: Supplementary file 11 — Source data Fig. 6 [file 44321_2026_418_MOESM11_ESM.zip › Figure 6/Data-Figure 6B/Day 7/2-3.jpg]

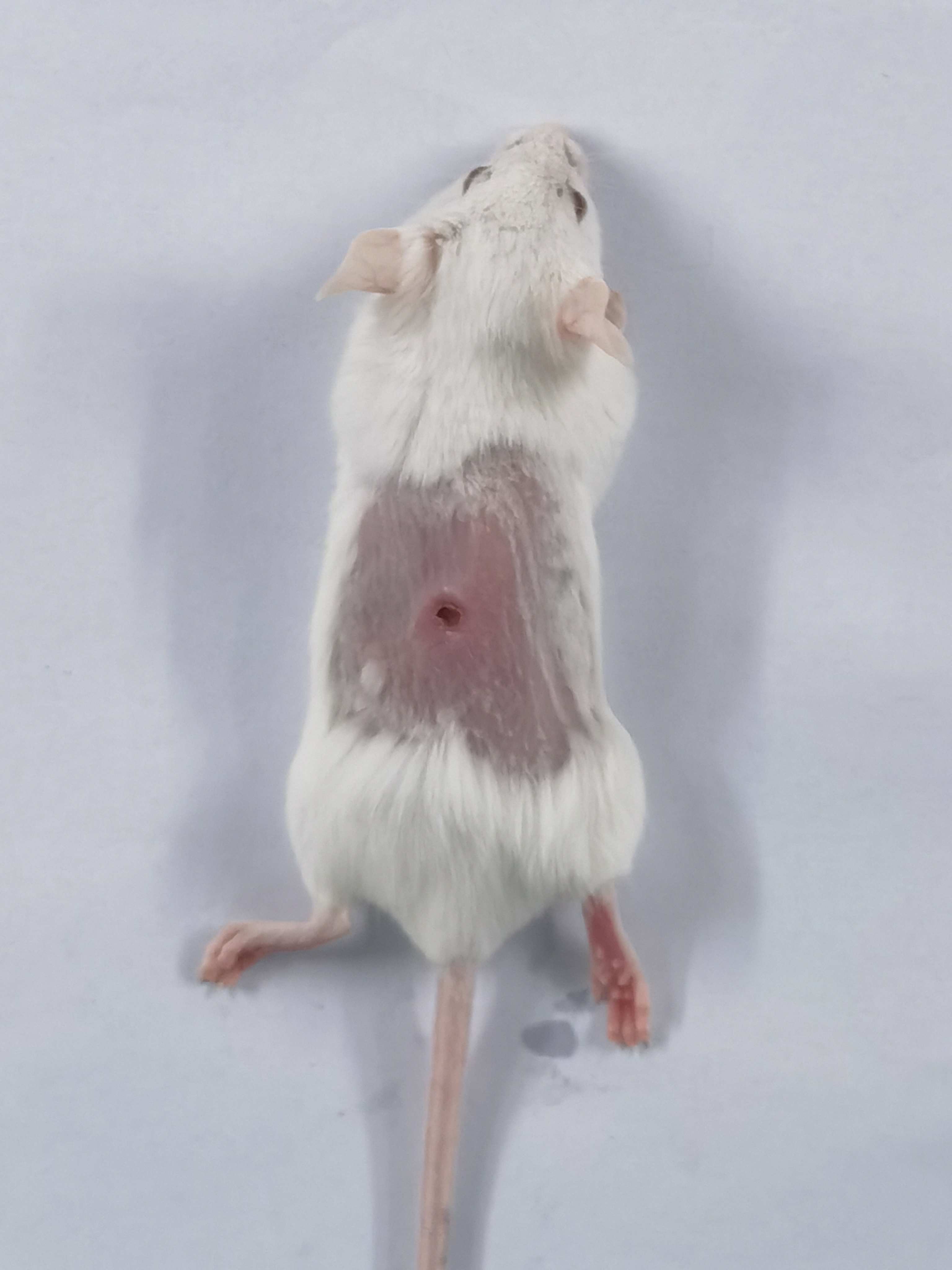

Supplement: Supplementary file 11 — Source data Fig. 6 [file 44321_2026_418_MOESM11_ESM.zip › Figure 6/Data-Figure 6B/Day 7/2-2.jpg]

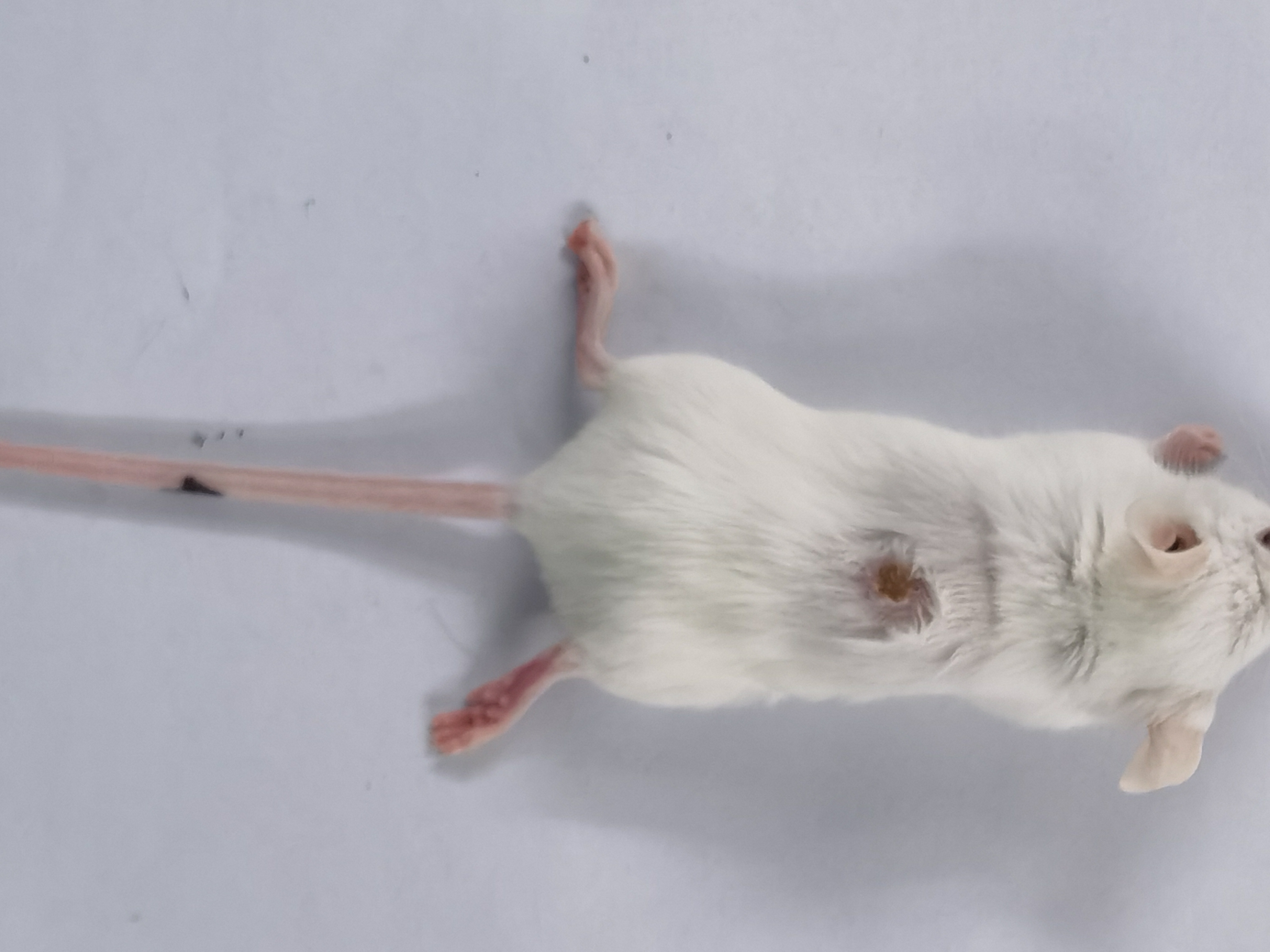

Supplement: Supplementary file 11 — Source data Fig. 6 [file 44321_2026_418_MOESM11_ESM.zip › Figure 6/Data-Figure 6B/Day 7/4-4.jpg]

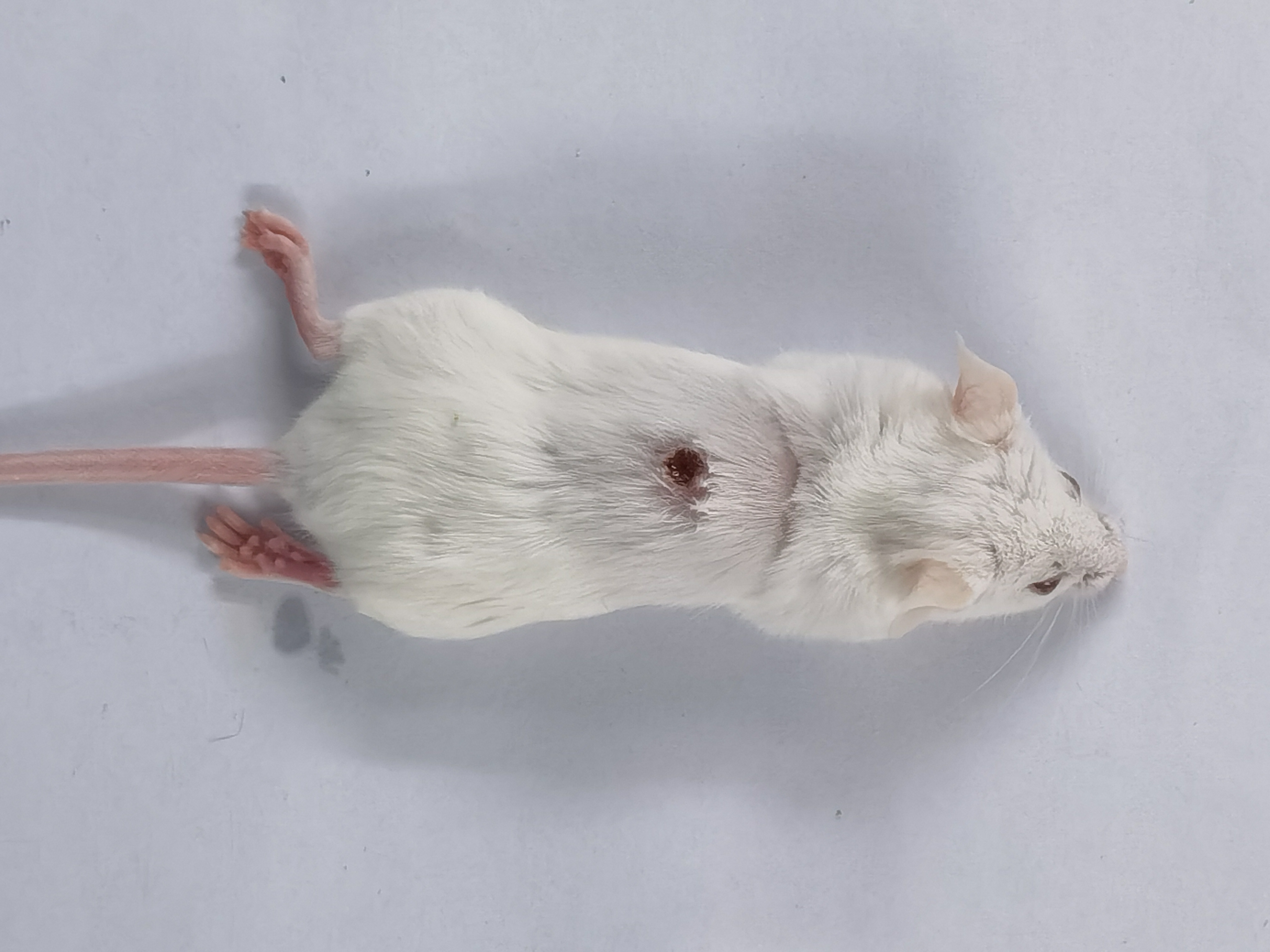

Supplement: Supplementary file 11 — Source data Fig. 6 [file 44321_2026_418_MOESM11_ESM.zip › Figure 6/Data-Figure 6B/Day 7/4-1.jpg]

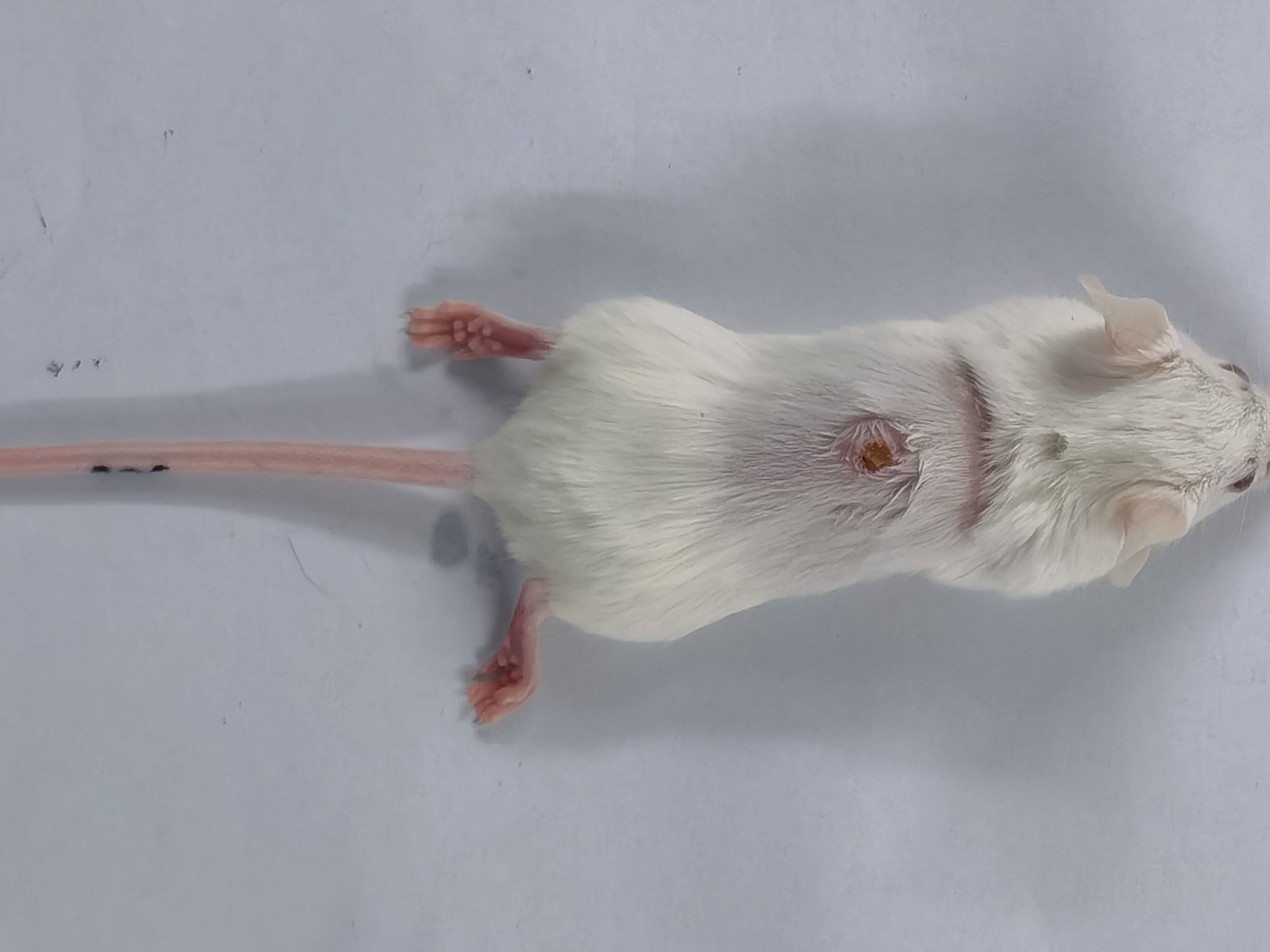

Supplement: Supplementary file 11 — Source data Fig. 6 [file 44321_2026_418_MOESM11_ESM.zip › Figure 6/Data-Figure 6B/Day 7/4-3.jpg]

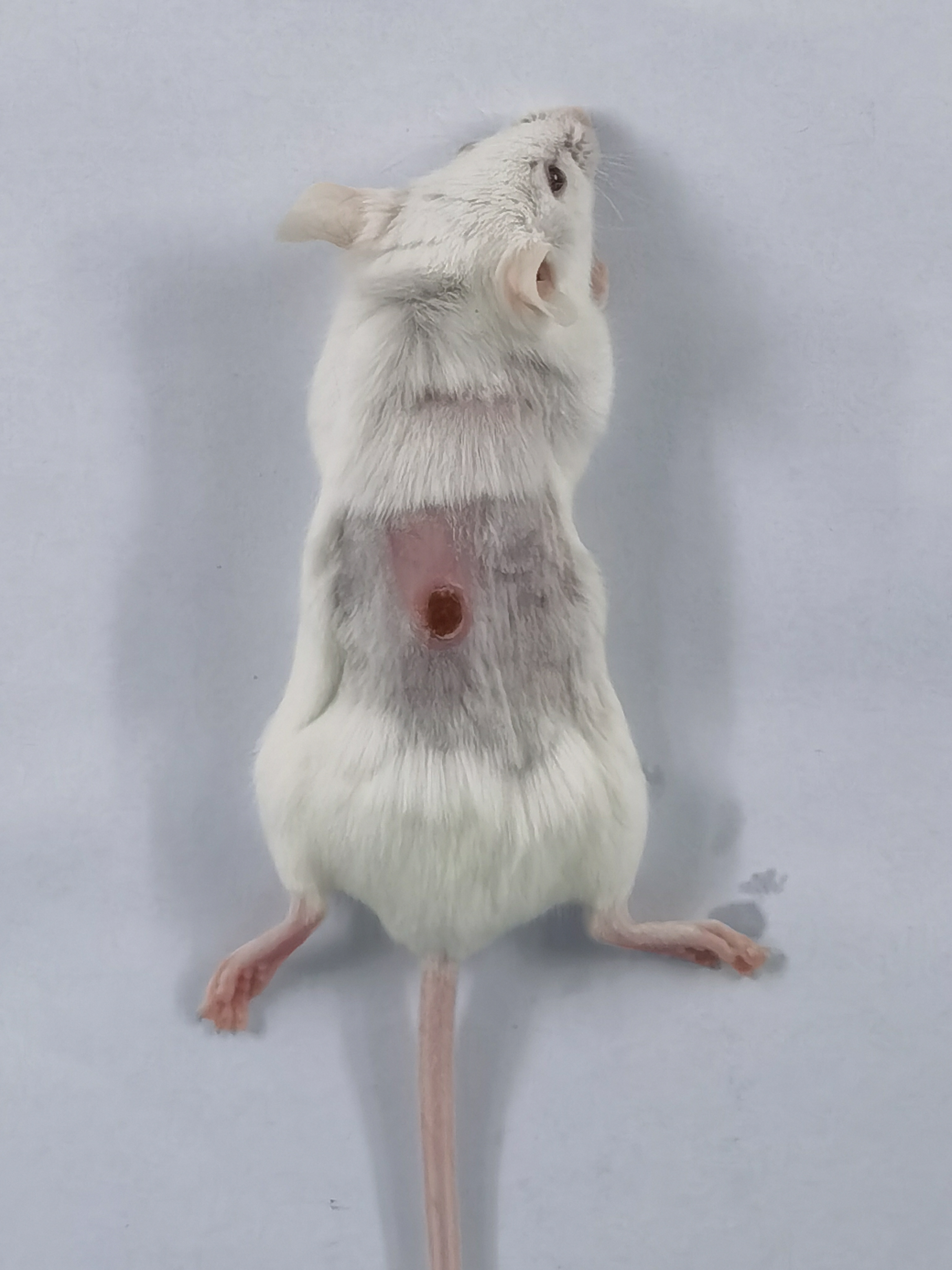

Supplement: Supplementary file 11 — Source data Fig. 6 [file 44321_2026_418_MOESM11_ESM.zip › Figure 6/Data-Figure 6B/Day 7/2-5.jpg]

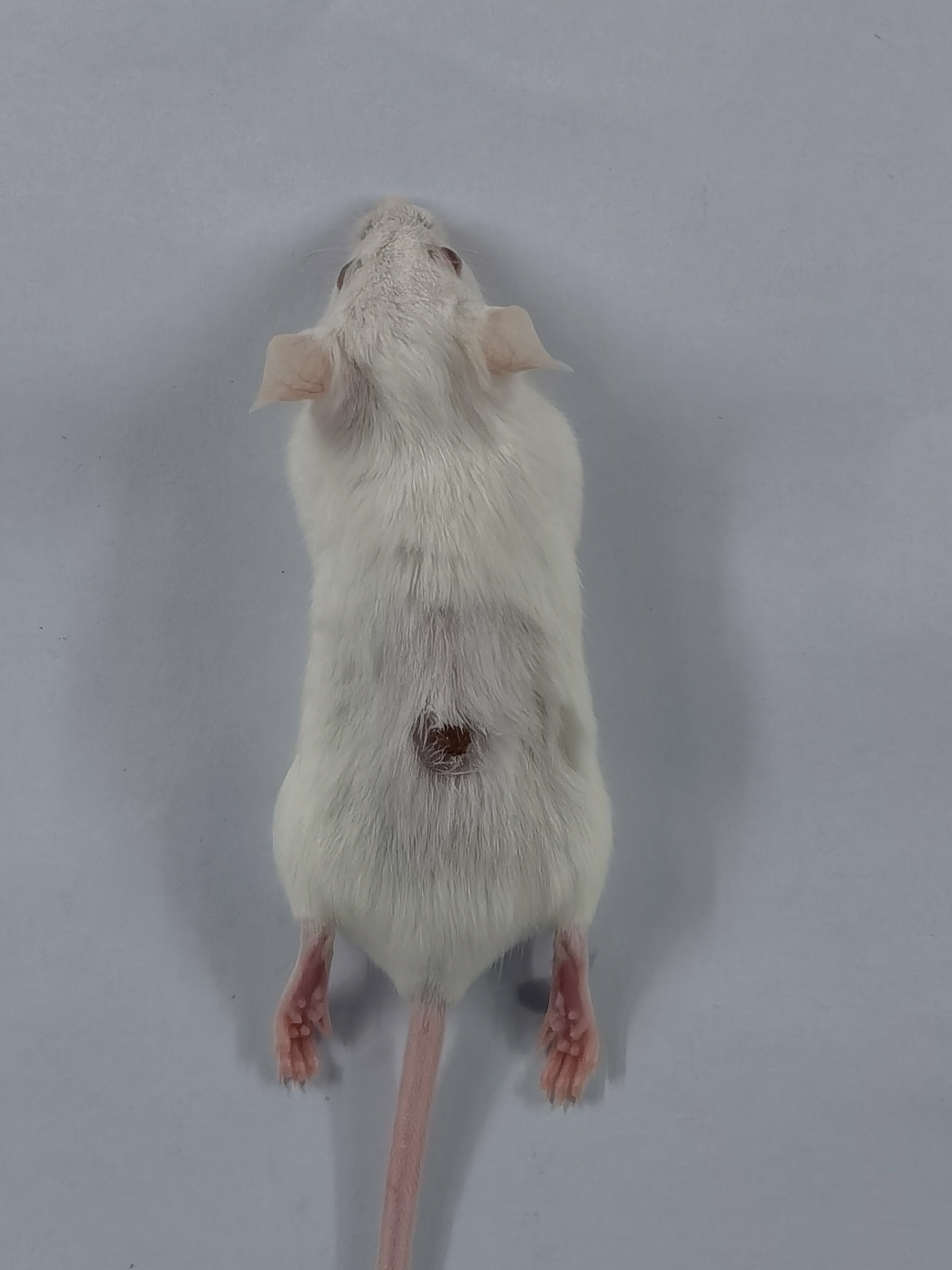

Supplement: Supplementary file 11 — Source data Fig. 6 [file 44321_2026_418_MOESM11_ESM.zip › Figure 6/Data-Figure 6B/Day 7/2-4.jpg]

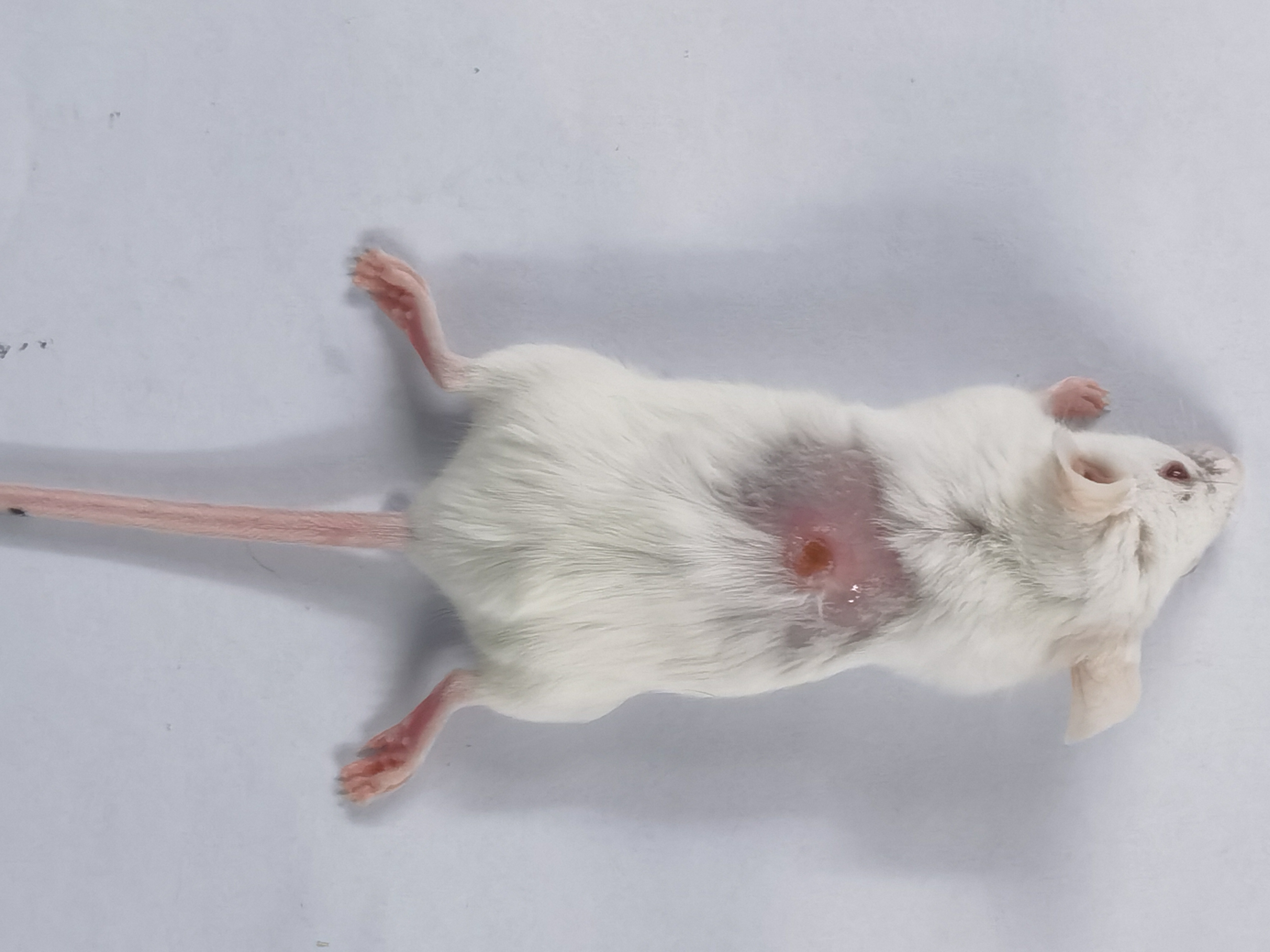

Supplement: Supplementary file 11 — Source data Fig. 6 [file 44321_2026_418_MOESM11_ESM.zip › Figure 6/Data-Figure 6B/Day 7/4-2.jpg]

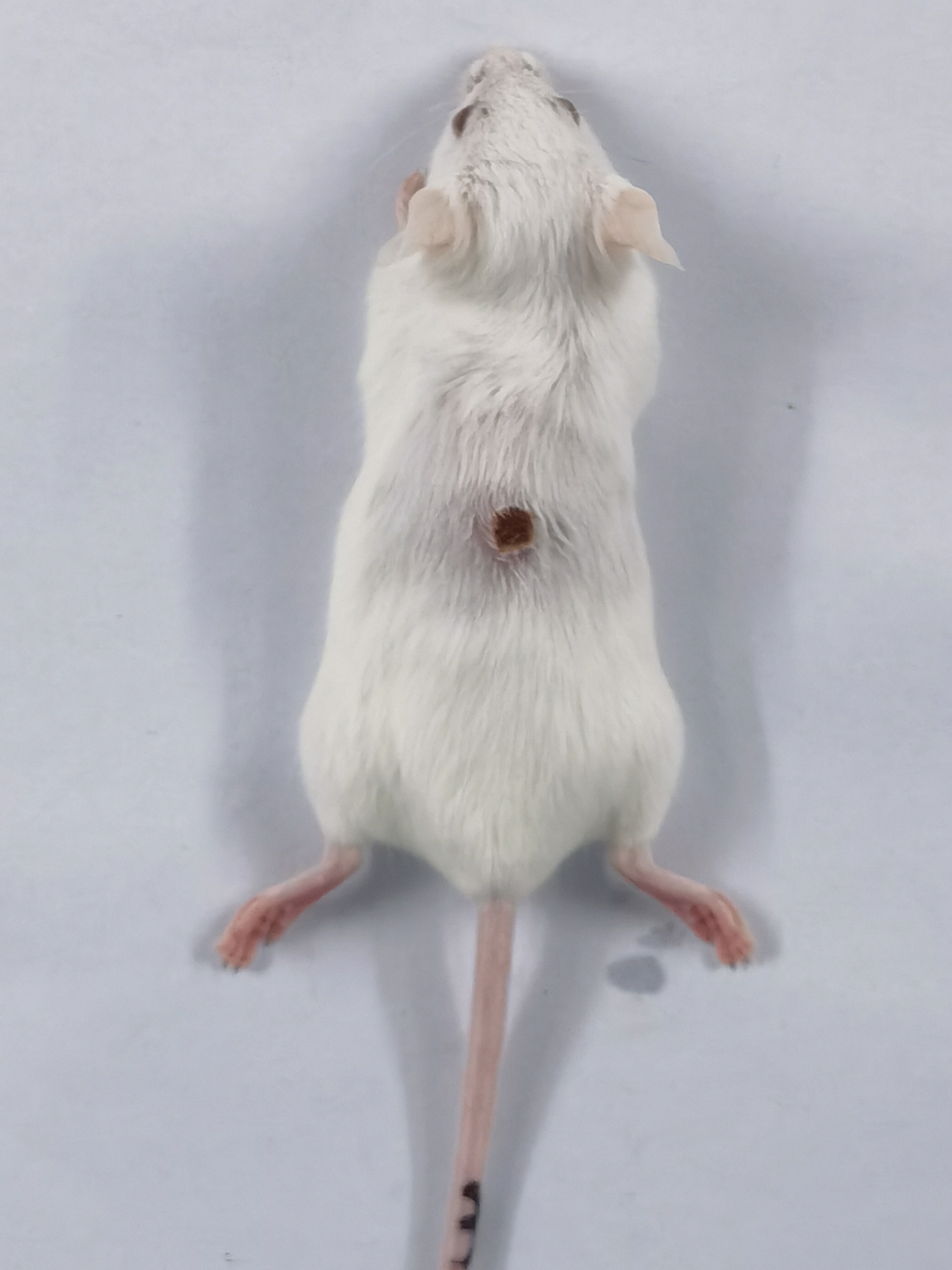

Supplement: Supplementary file 11 — Source data Fig. 6 [file 44321_2026_418_MOESM11_ESM.zip › Figure 6/Data-Figure 6B/Day 7/3-3.jpg]

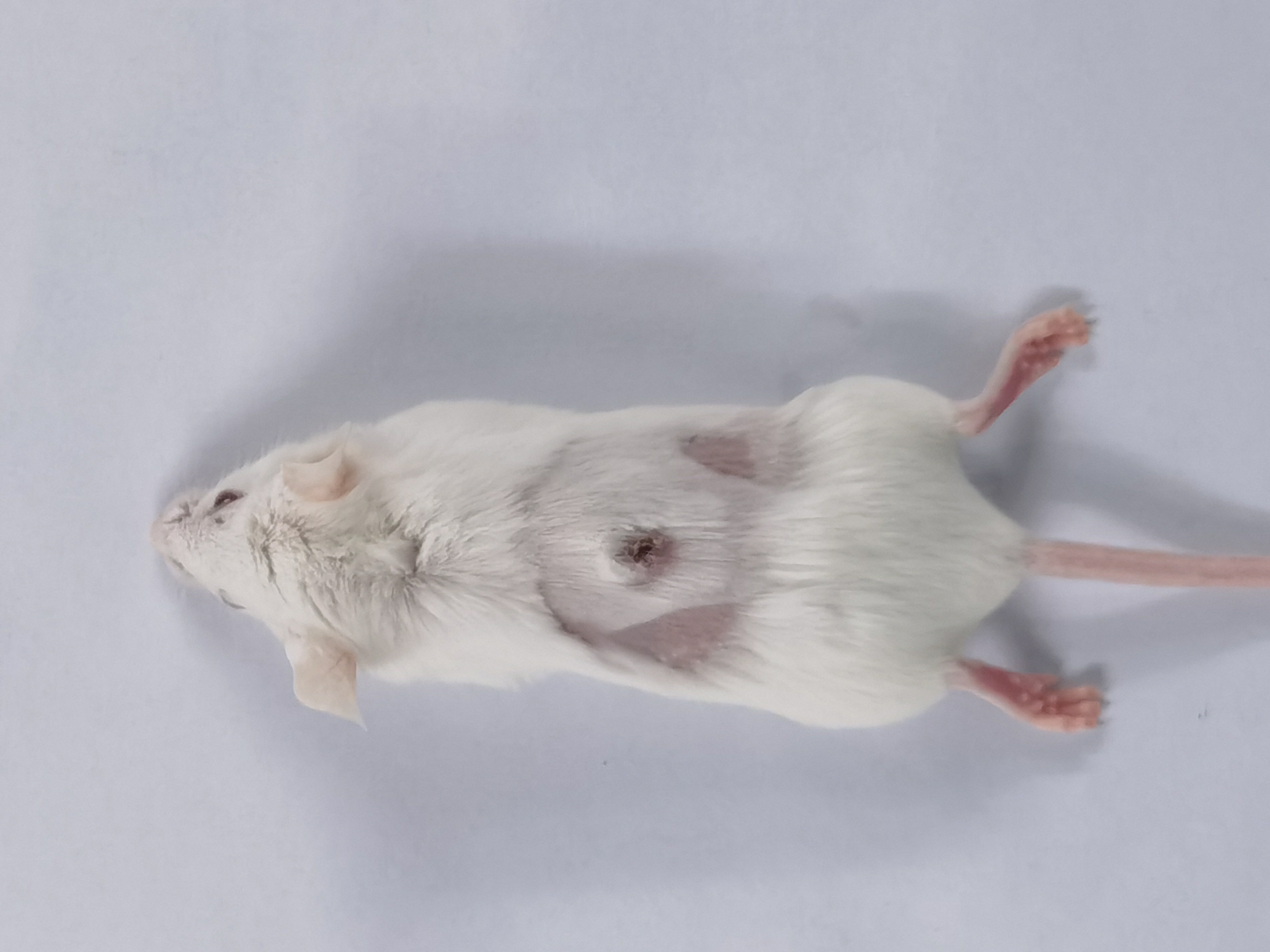

Supplement: Supplementary file 11 — Source data Fig. 6 [file 44321_2026_418_MOESM11_ESM.zip › Figure 6/Data-Figure 6B/Day 7/1-1.jpg]

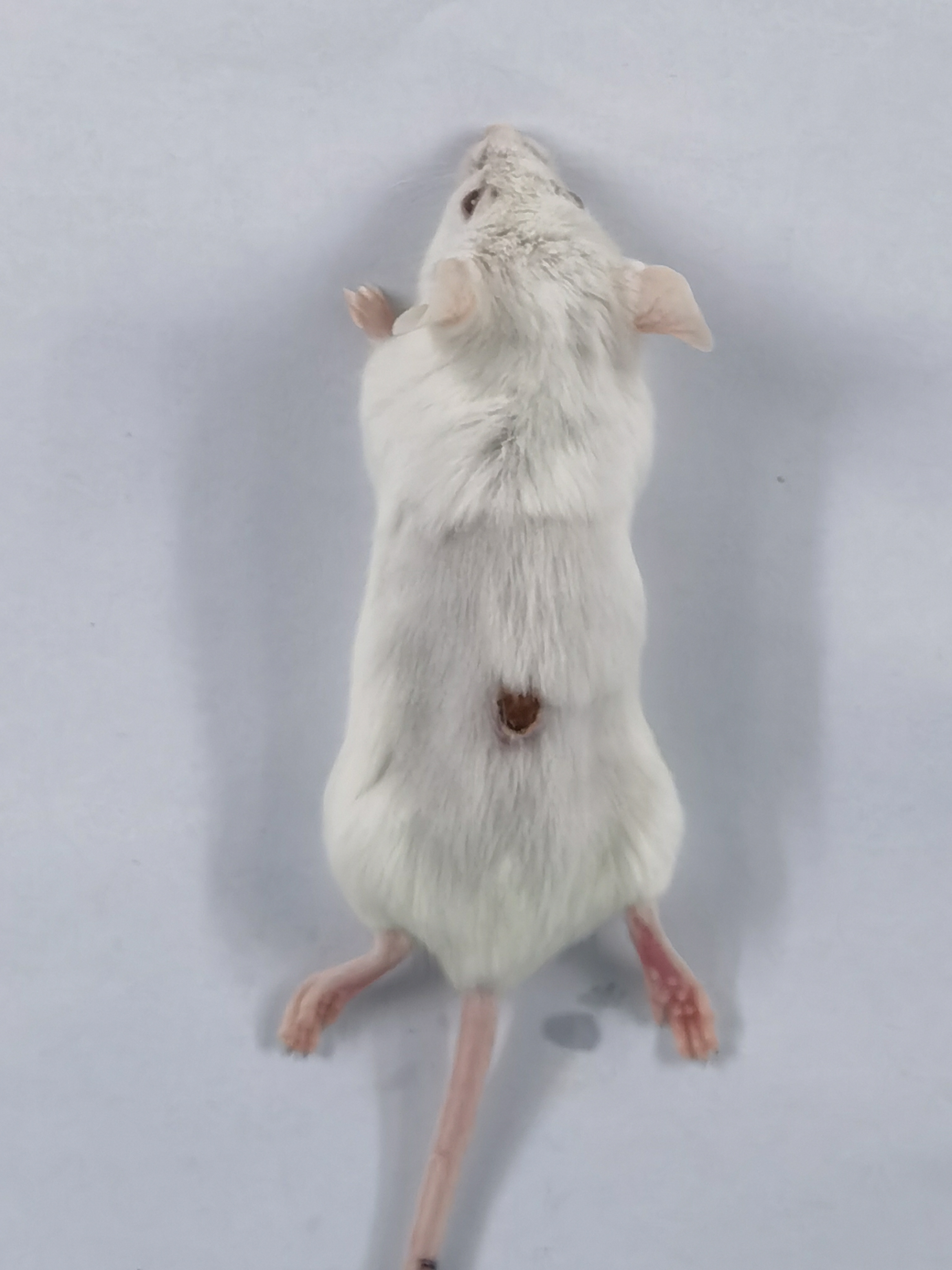

Supplement: Supplementary file 11 — Source data Fig. 6 [file 44321_2026_418_MOESM11_ESM.zip › Figure 6/Data-Figure 6B/Day 7/3-2.jpg]

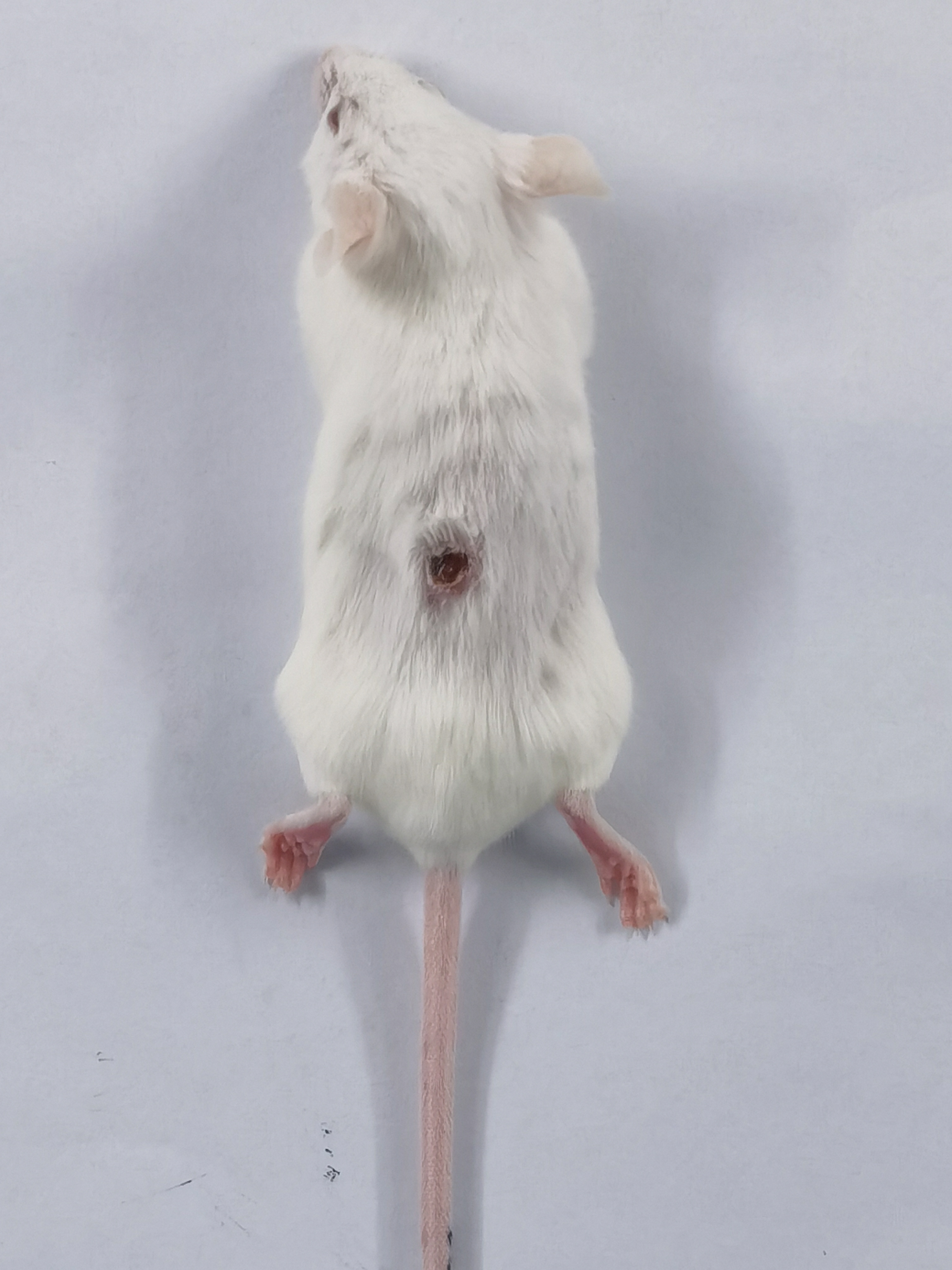

Supplement: Supplementary file 11 — Source data Fig. 6 [file 44321_2026_418_MOESM11_ESM.zip › Figure 6/Data-Figure 6B/Day 7/1-2.jpg]

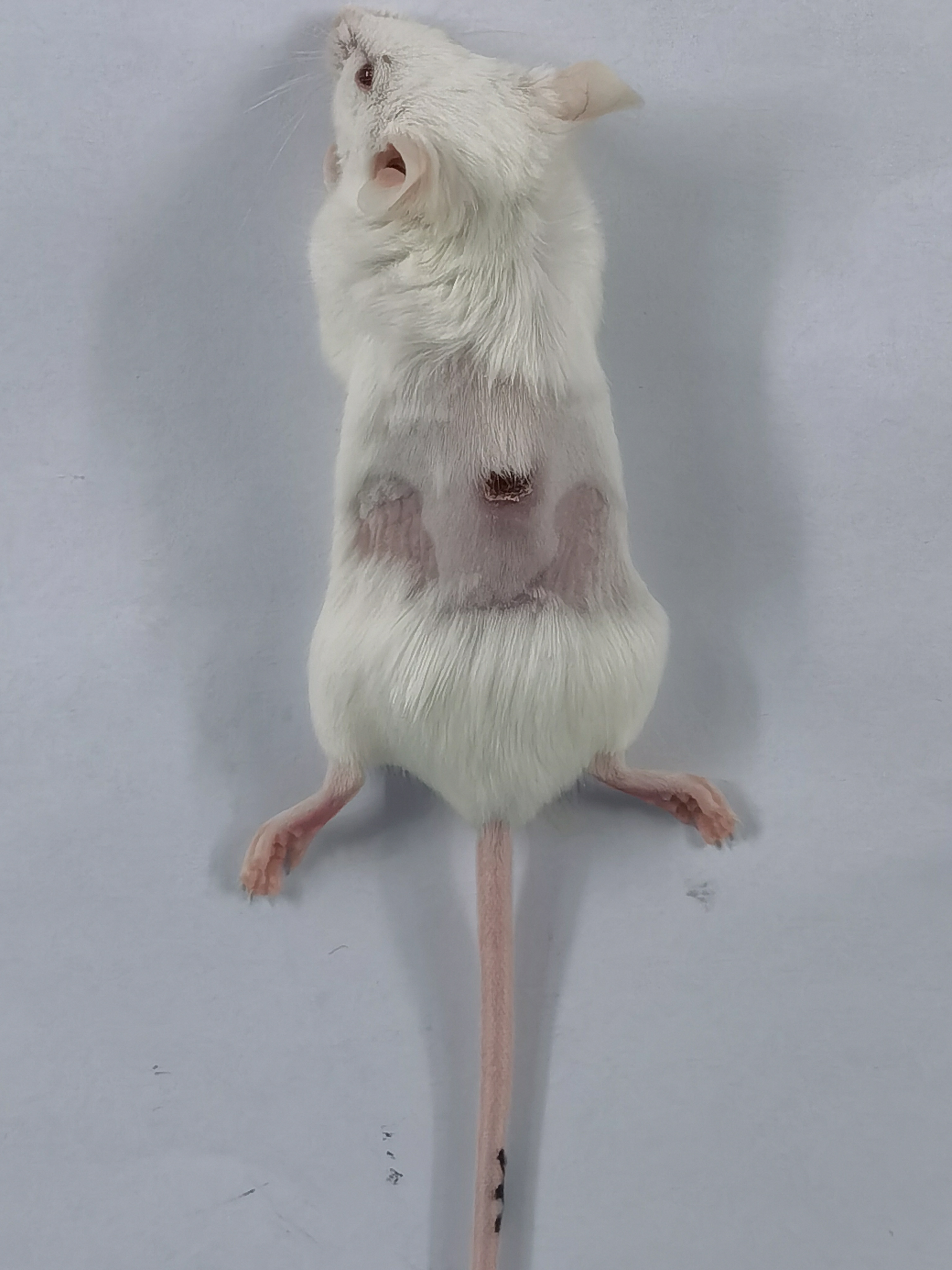

Supplement: Supplementary file 11 — Source data Fig. 6 [file 44321_2026_418_MOESM11_ESM.zip › Figure 6/Data-Figure 6B/Day 7/1-3.jpg]

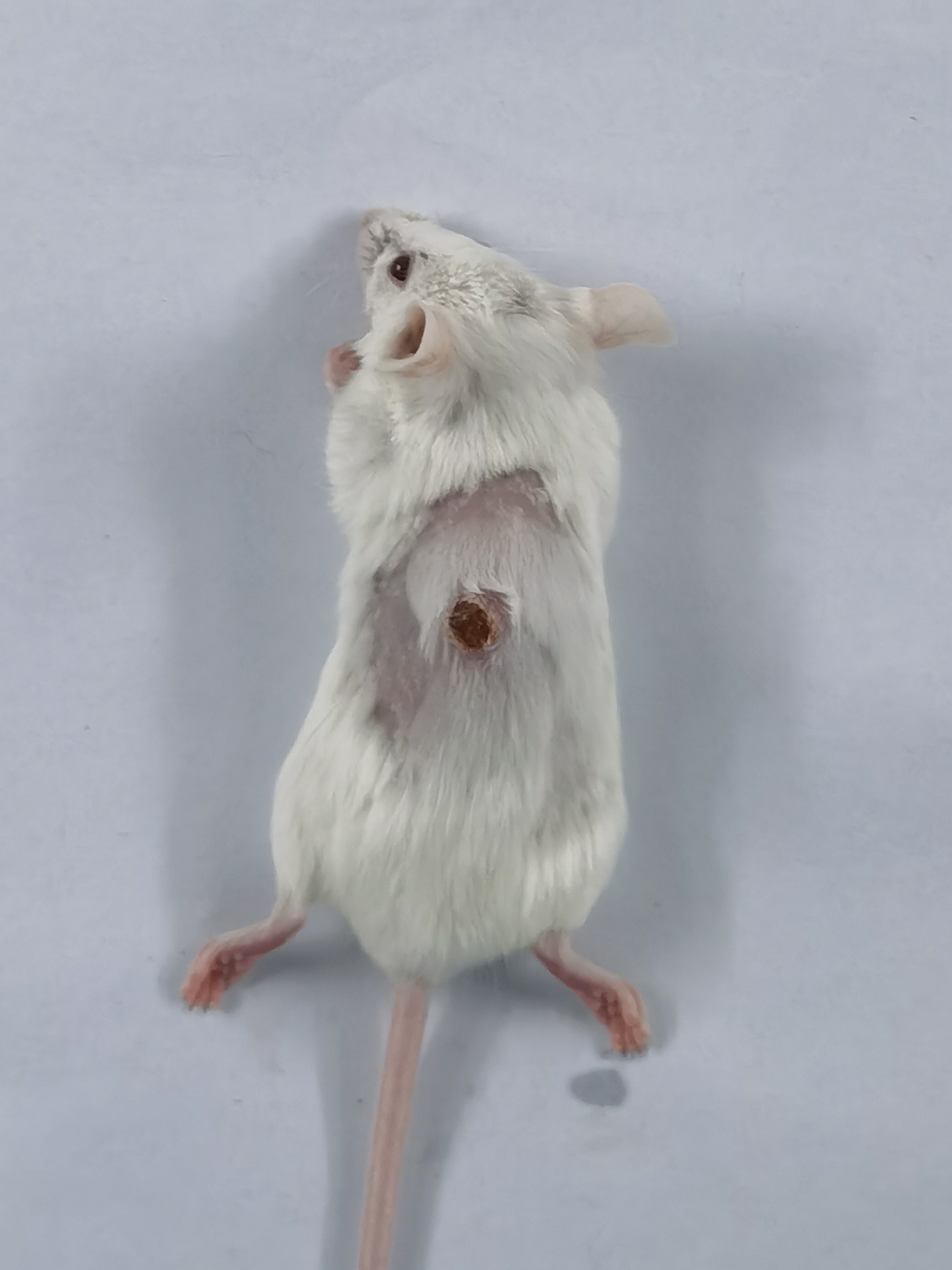

Supplement: Supplementary file 11 — Source data Fig. 6 [file 44321_2026_418_MOESM11_ESM.zip › Figure 6/Data-Figure 6B/Day 7/3-1.jpg]

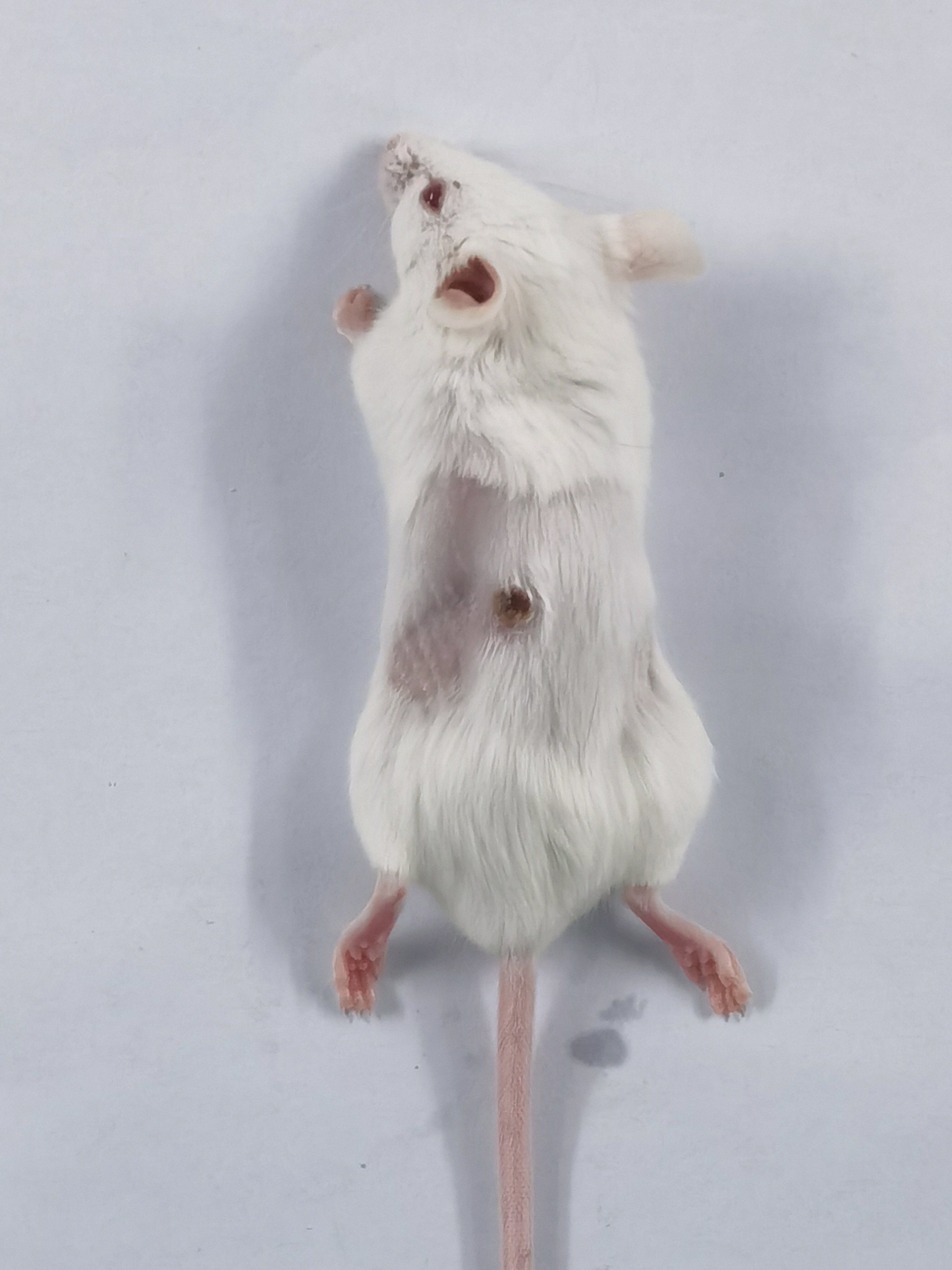

Supplement: Supplementary file 11 — Source data Fig. 6 [file 44321_2026_418_MOESM11_ESM.zip › Figure 6/Data-Figure 6B/Day 7/3-5.jpg]

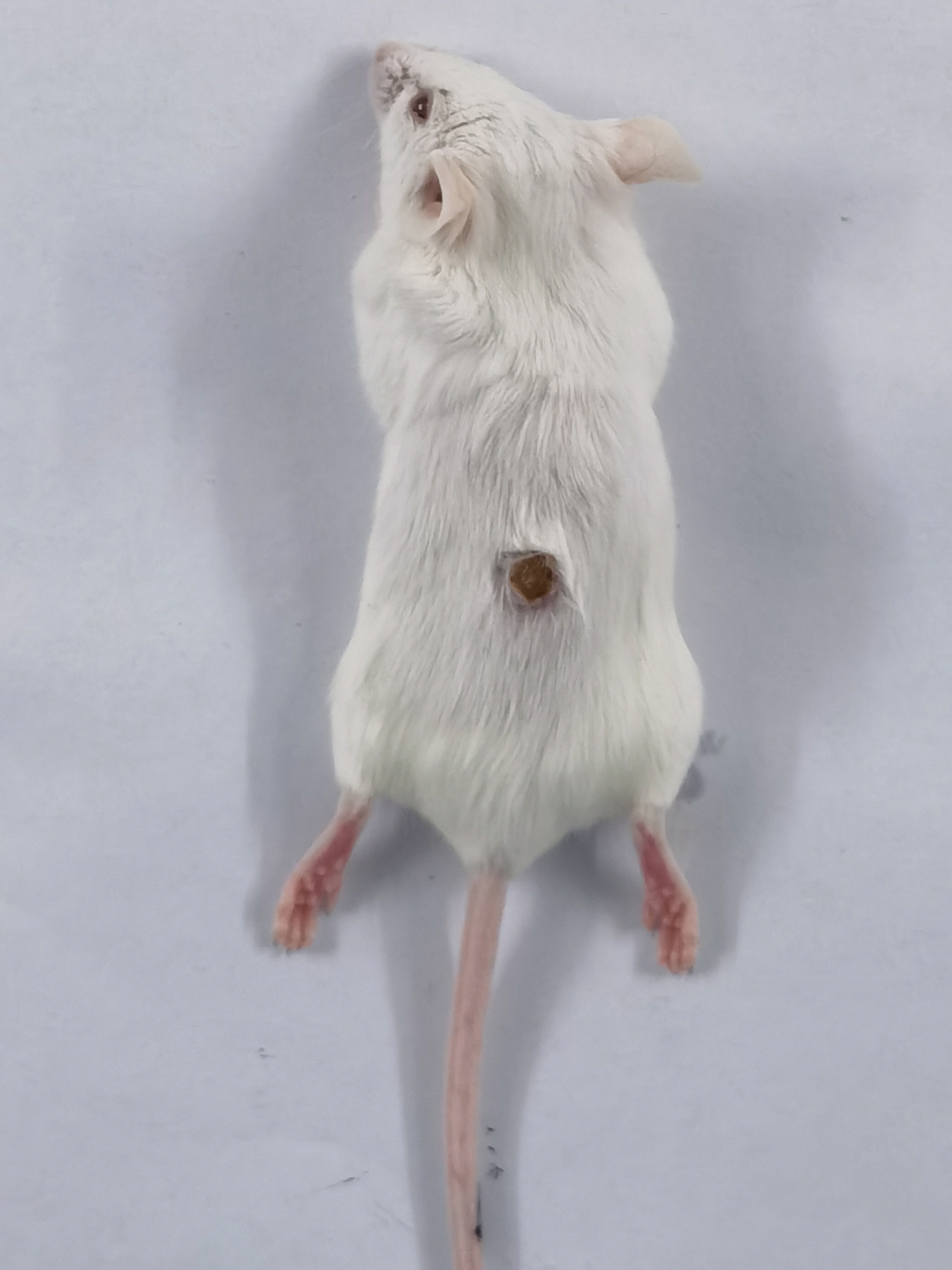

Supplement: Supplementary file 11 — Source data Fig. 6 [file 44321_2026_418_MOESM11_ESM.zip › Figure 6/Data-Figure 6B/Day 7/3-4.jpg]

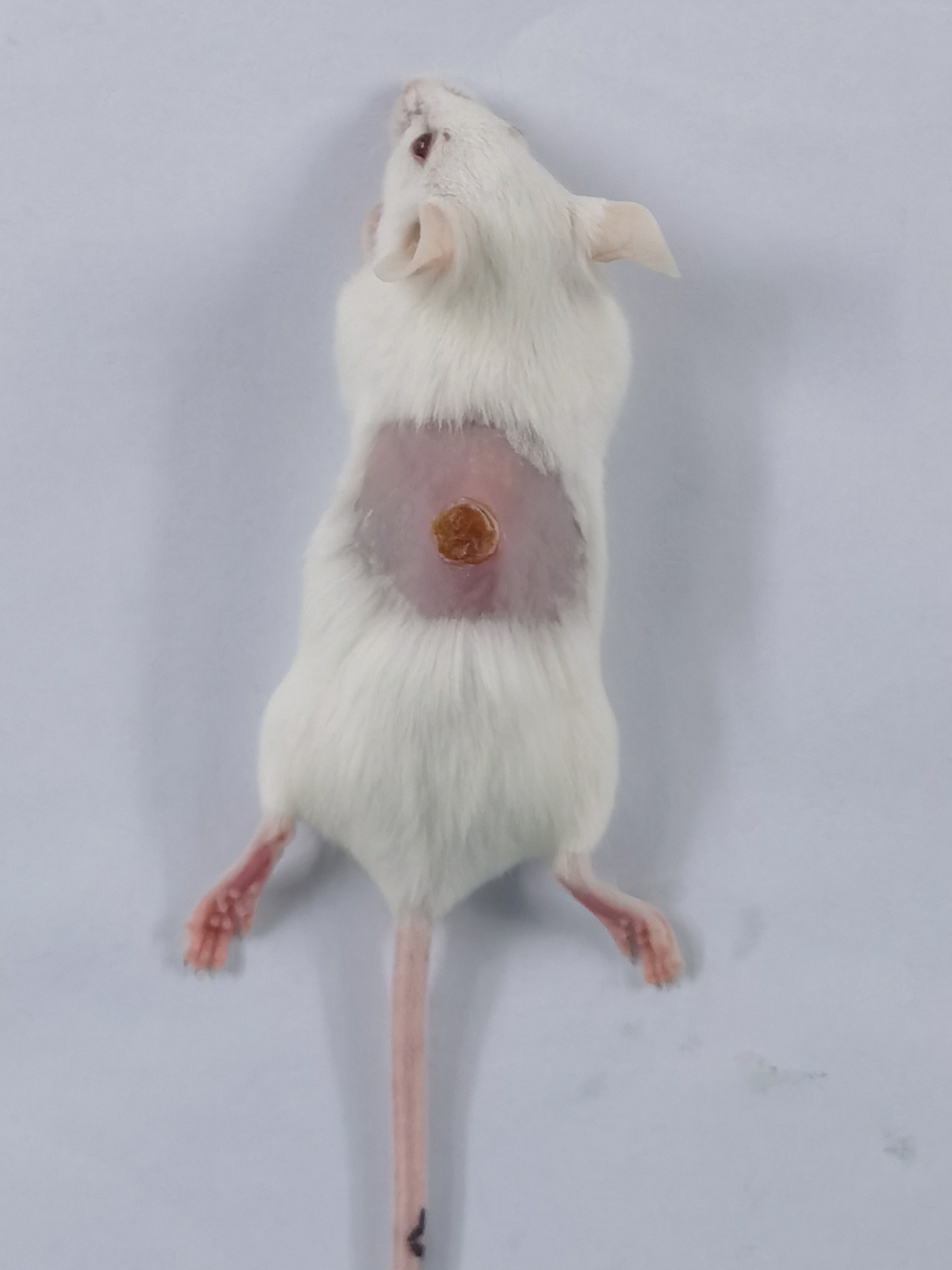

Supplement: Supplementary file 11 — Source data Fig. 6 [file 44321_2026_418_MOESM11_ESM.zip › Figure 6/Data-Figure 6B/Day 7/1-4.jpg]

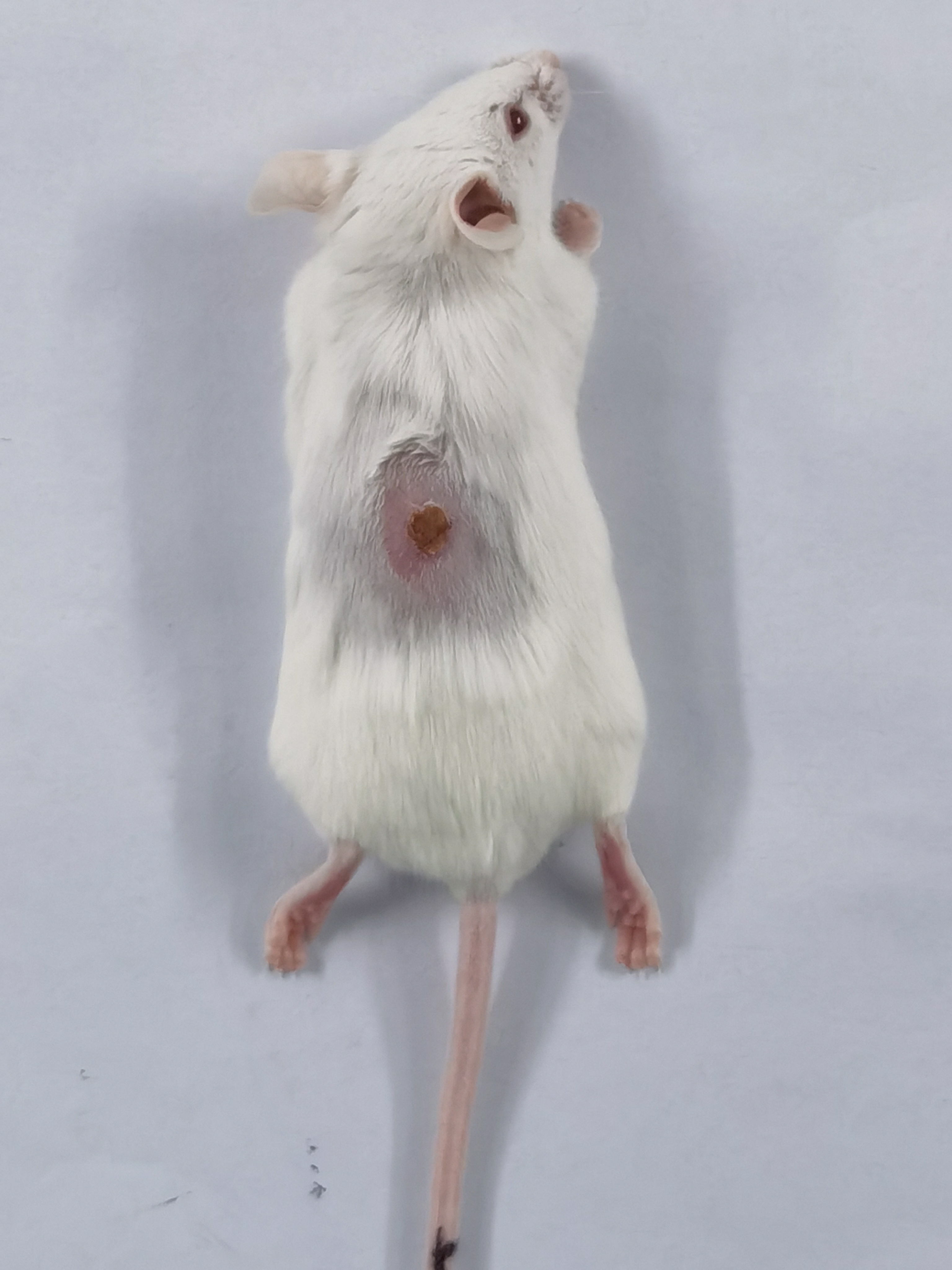

Supplement: Supplementary file 11 — Source data Fig. 6 [file 44321_2026_418_MOESM11_ESM.zip › Figure 6/Data-Figure 6B/Day 7/1-5.jpg]
