# Supplementary figures and images for: SHP-1 agonist SC-43 limits methicillin-resistant Staphylococcus aureus infection through inhibition of heme biosynthesis (part 1 of 2)
Source: EMBO Mol Med. 2026 Apr 10;18(5):1990–2005. doi: 10.1038/s44321-026-00418-4 (PMC13179323; doi:10.1038/s44321-026-00418-4)

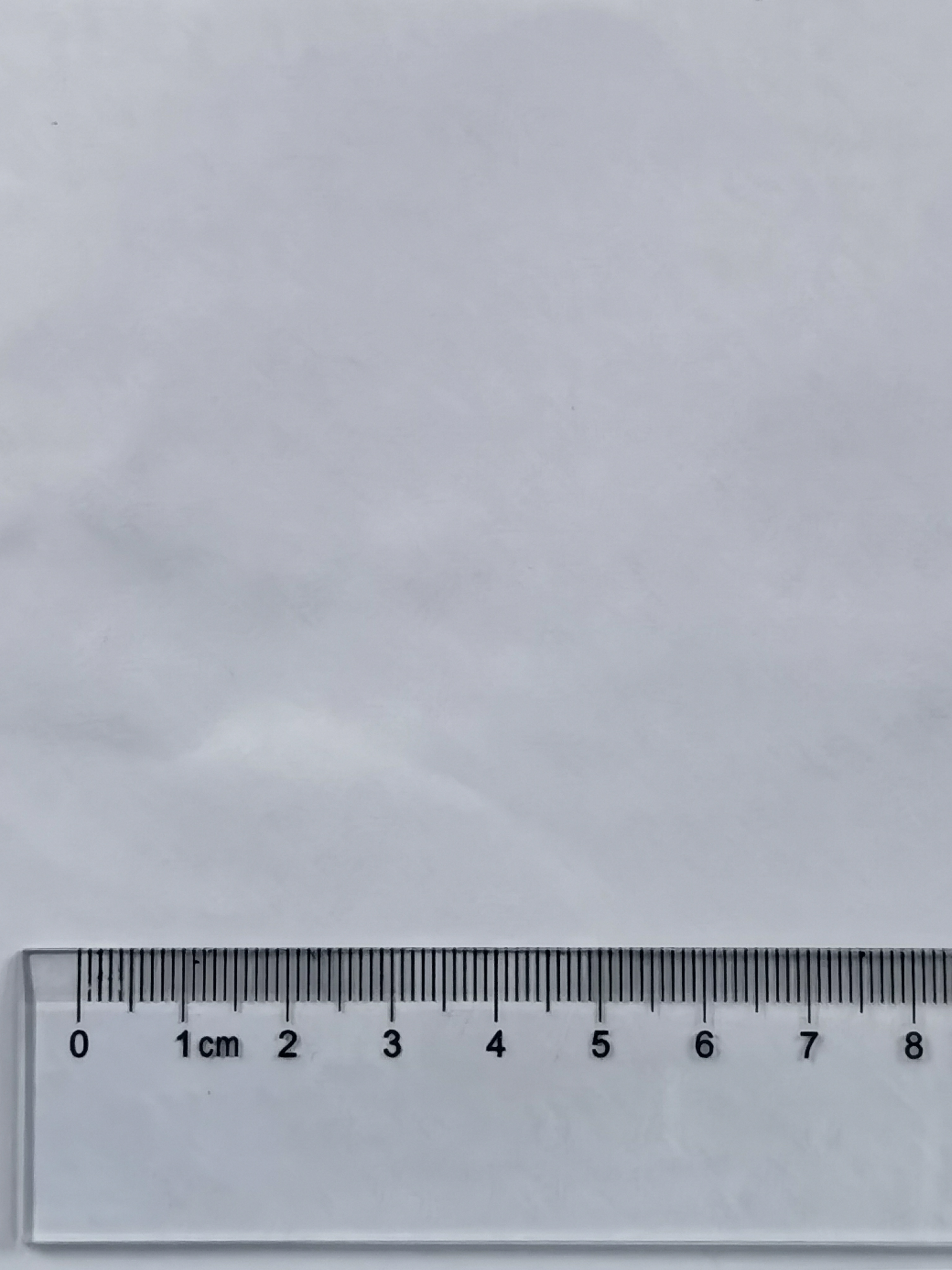

Supplement: Supplementary file 11 — Source data Fig. 6 [file 44321_2026_418_MOESM11_ESM.zip › Figure 6/Data-Figure 6B/ruler.jpg]

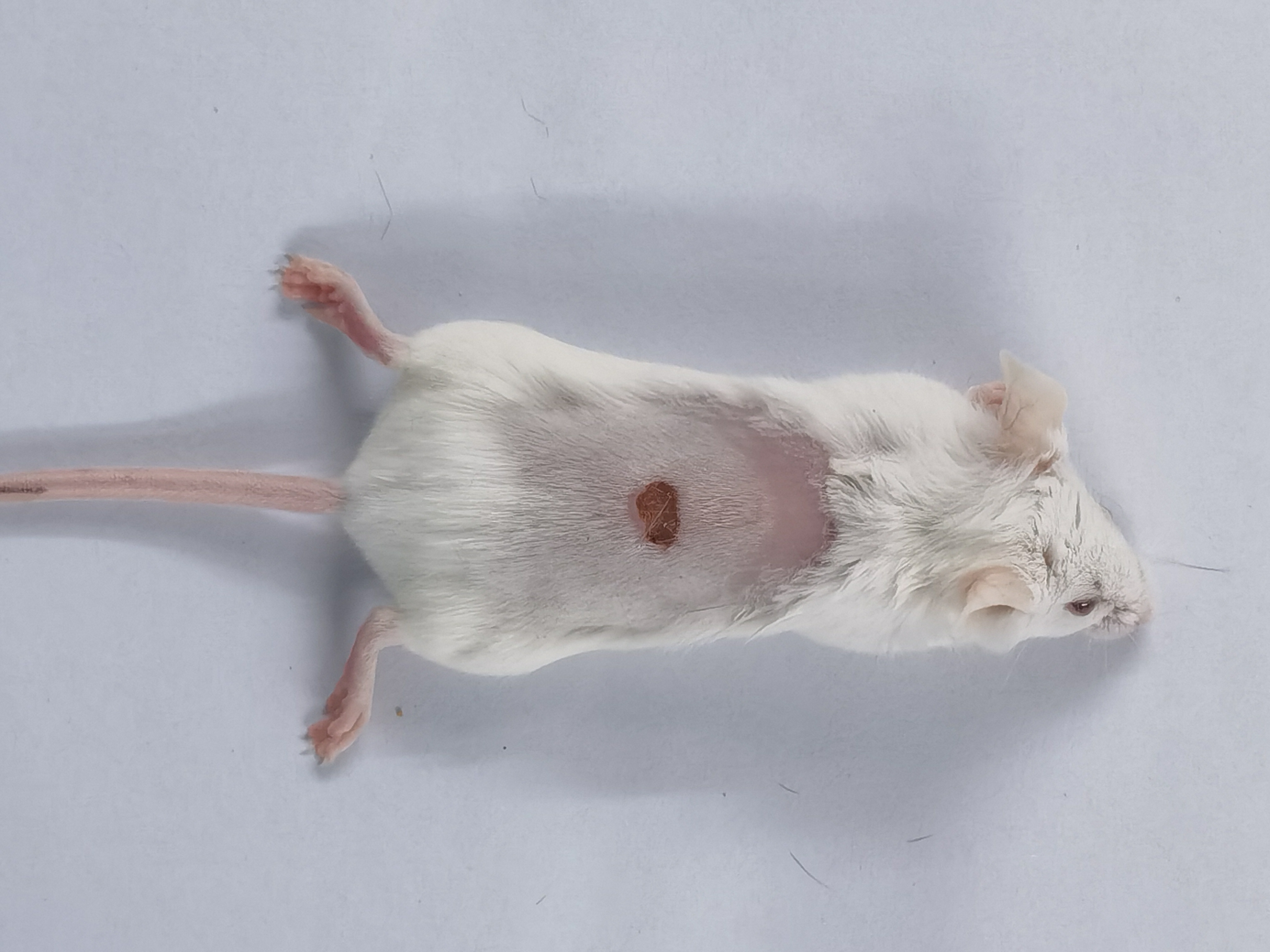

Supplement: Supplementary file 11 — Source data Fig. 6 [file 44321_2026_418_MOESM11_ESM.zip › Figure 6/Data-Figure 6B/Day 2/2-1.jpg]

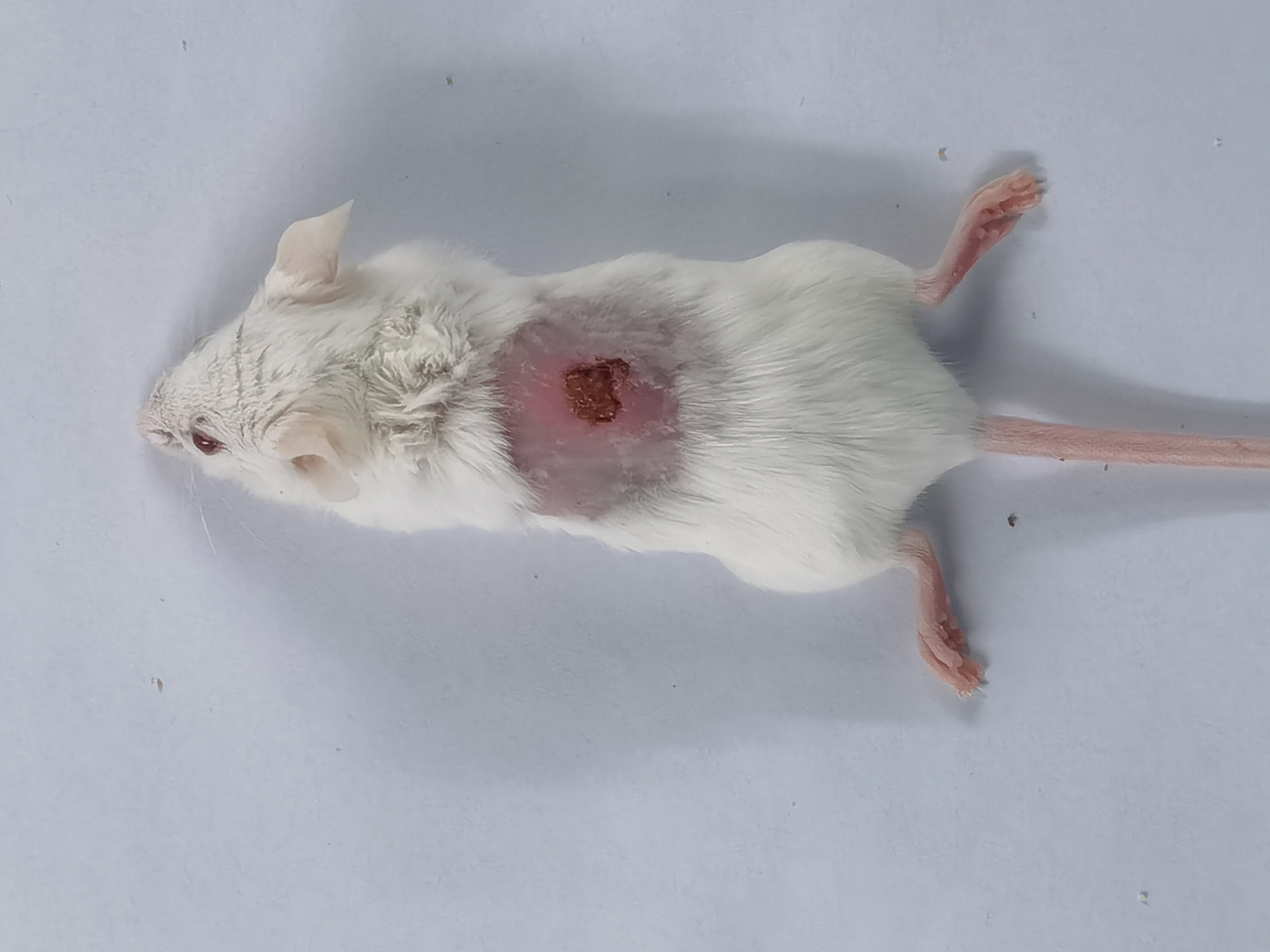

Supplement: Supplementary file 11 — Source data Fig. 6 [file 44321_2026_418_MOESM11_ESM.zip › Figure 6/Data-Figure 6B/Day 2/4-5.jpg]

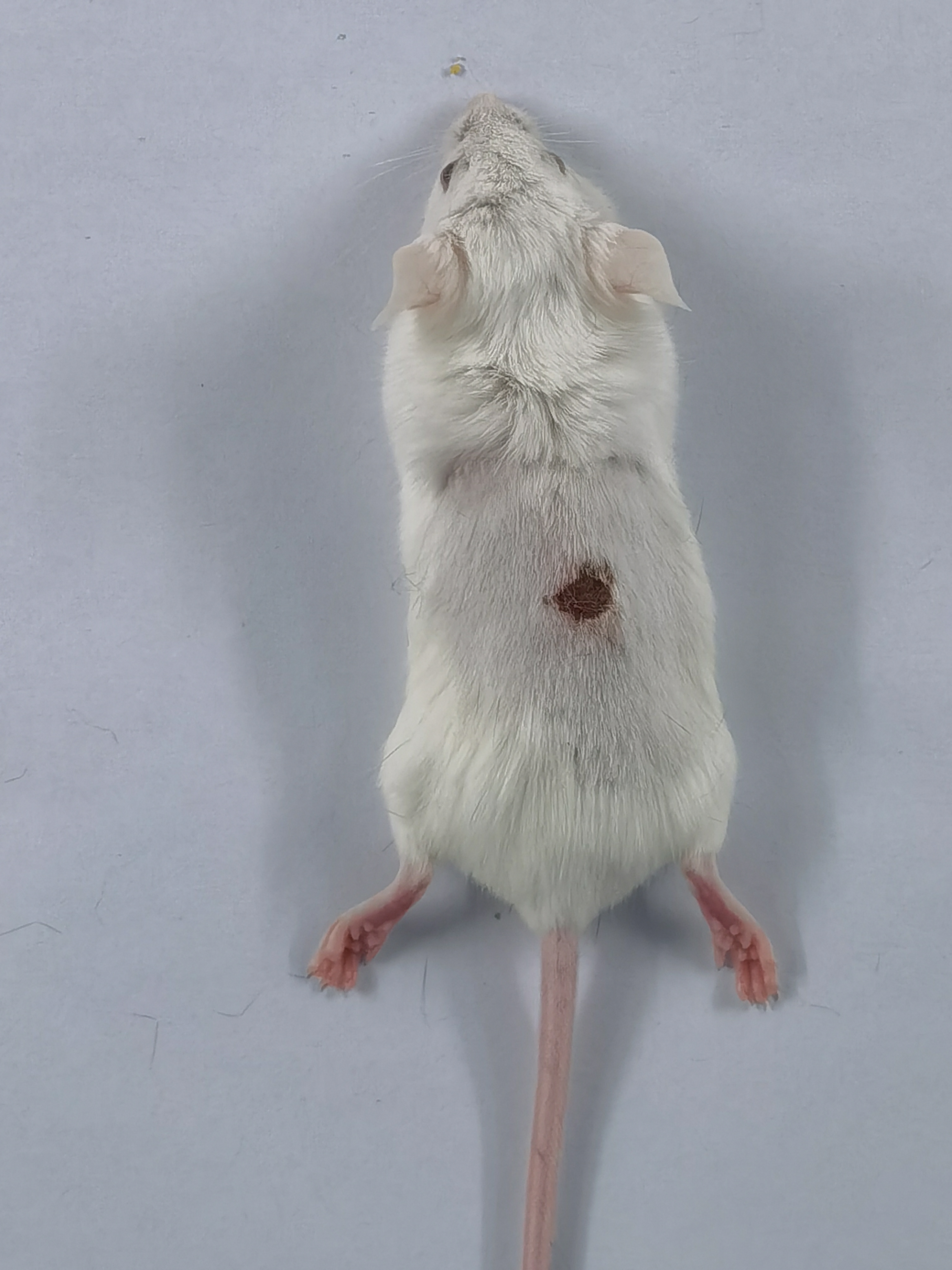

Supplement: Supplementary file 11 — Source data Fig. 6 [file 44321_2026_418_MOESM11_ESM.zip › Figure 6/Data-Figure 6B/Day 2/2-3.jpg]

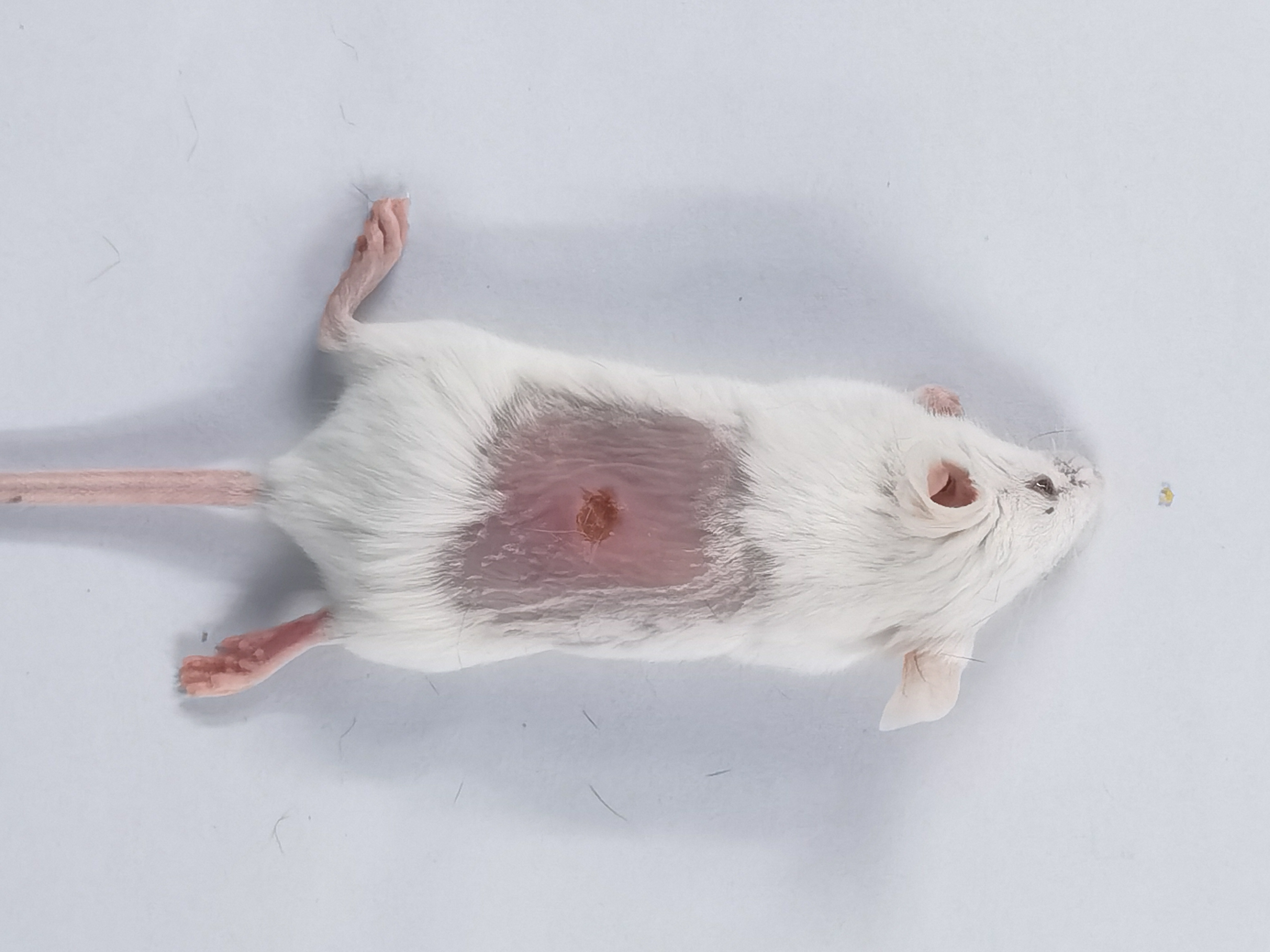

Supplement: Supplementary file 11 — Source data Fig. 6 [file 44321_2026_418_MOESM11_ESM.zip › Figure 6/Data-Figure 6B/Day 2/2-2.jpg]

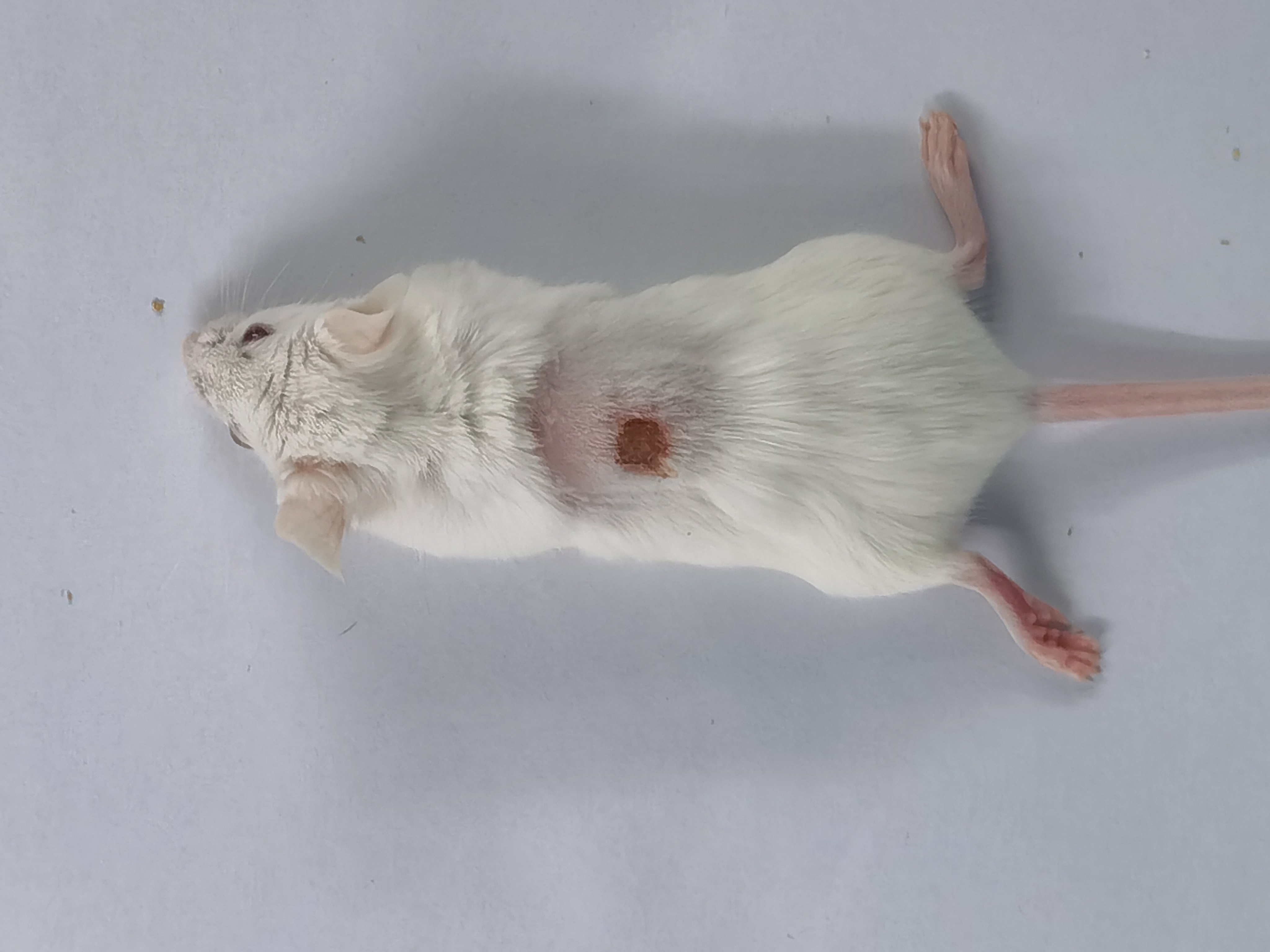

Supplement: Supplementary file 11 — Source data Fig. 6 [file 44321_2026_418_MOESM11_ESM.zip › Figure 6/Data-Figure 6B/Day 2/4-4.jpg]

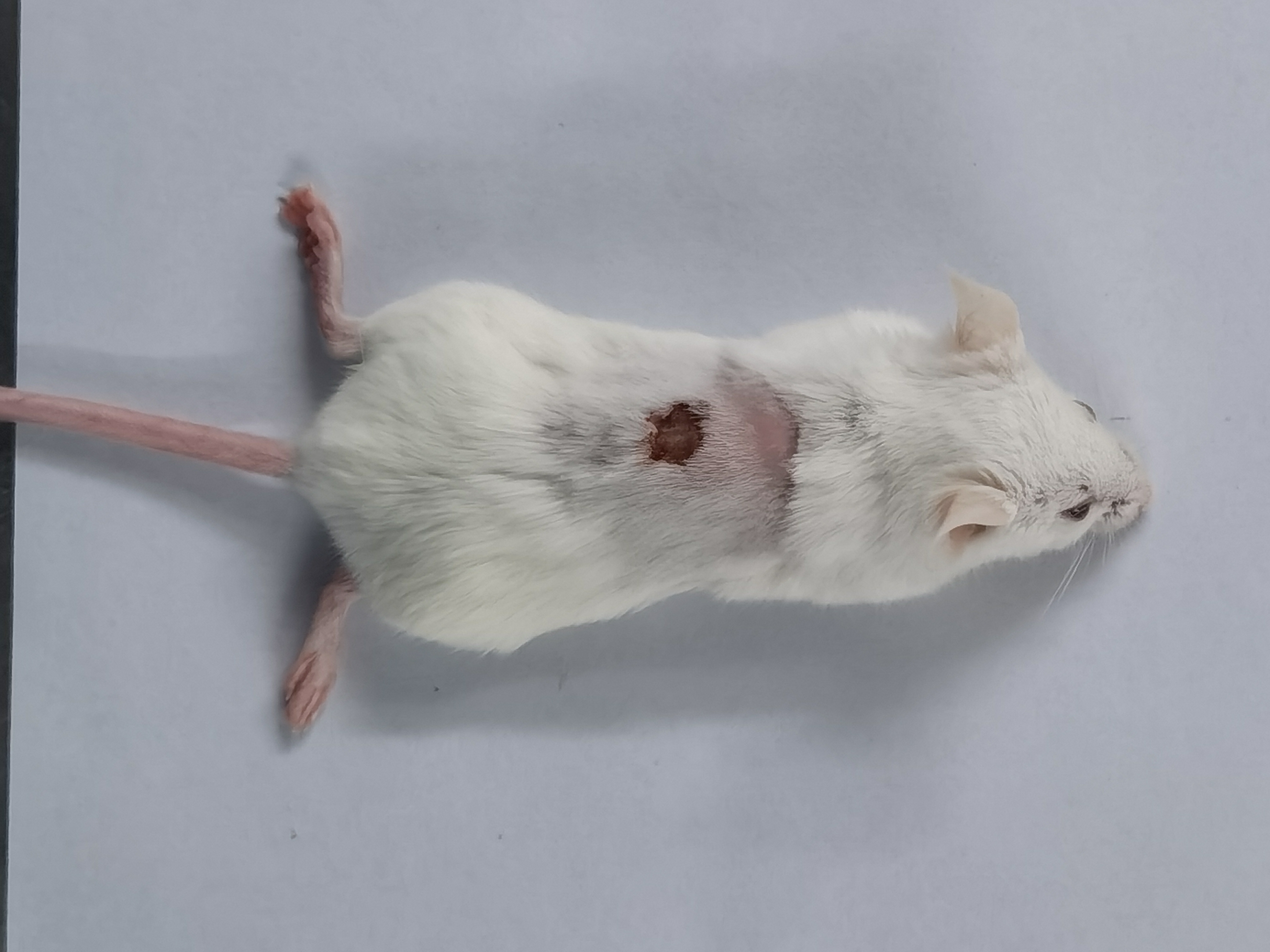

Supplement: Supplementary file 11 — Source data Fig. 6 [file 44321_2026_418_MOESM11_ESM.zip › Figure 6/Data-Figure 6B/Day 2/4-1.jpg]

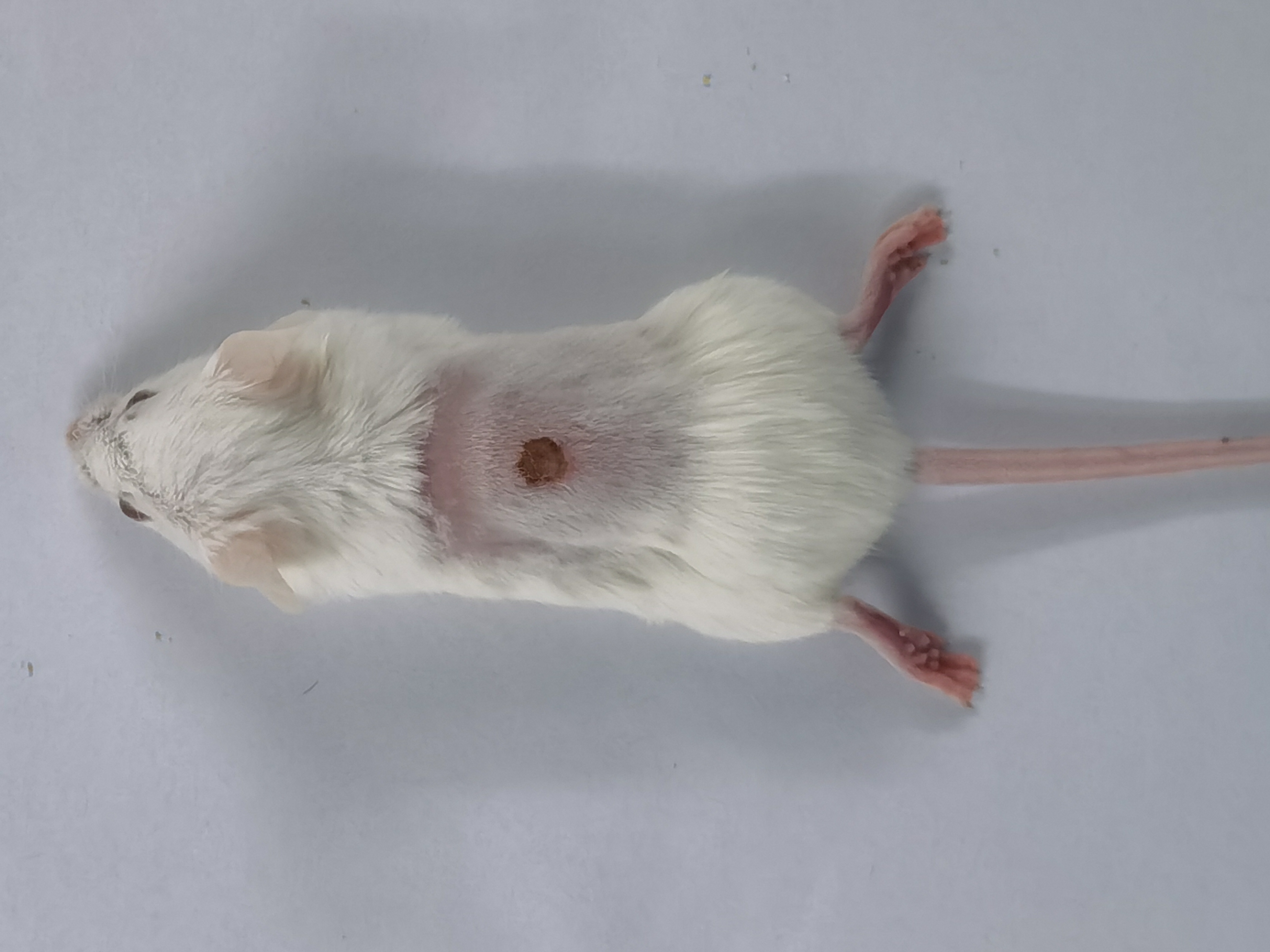

Supplement: Supplementary file 11 — Source data Fig. 6 [file 44321_2026_418_MOESM11_ESM.zip › Figure 6/Data-Figure 6B/Day 2/4-3.jpg]

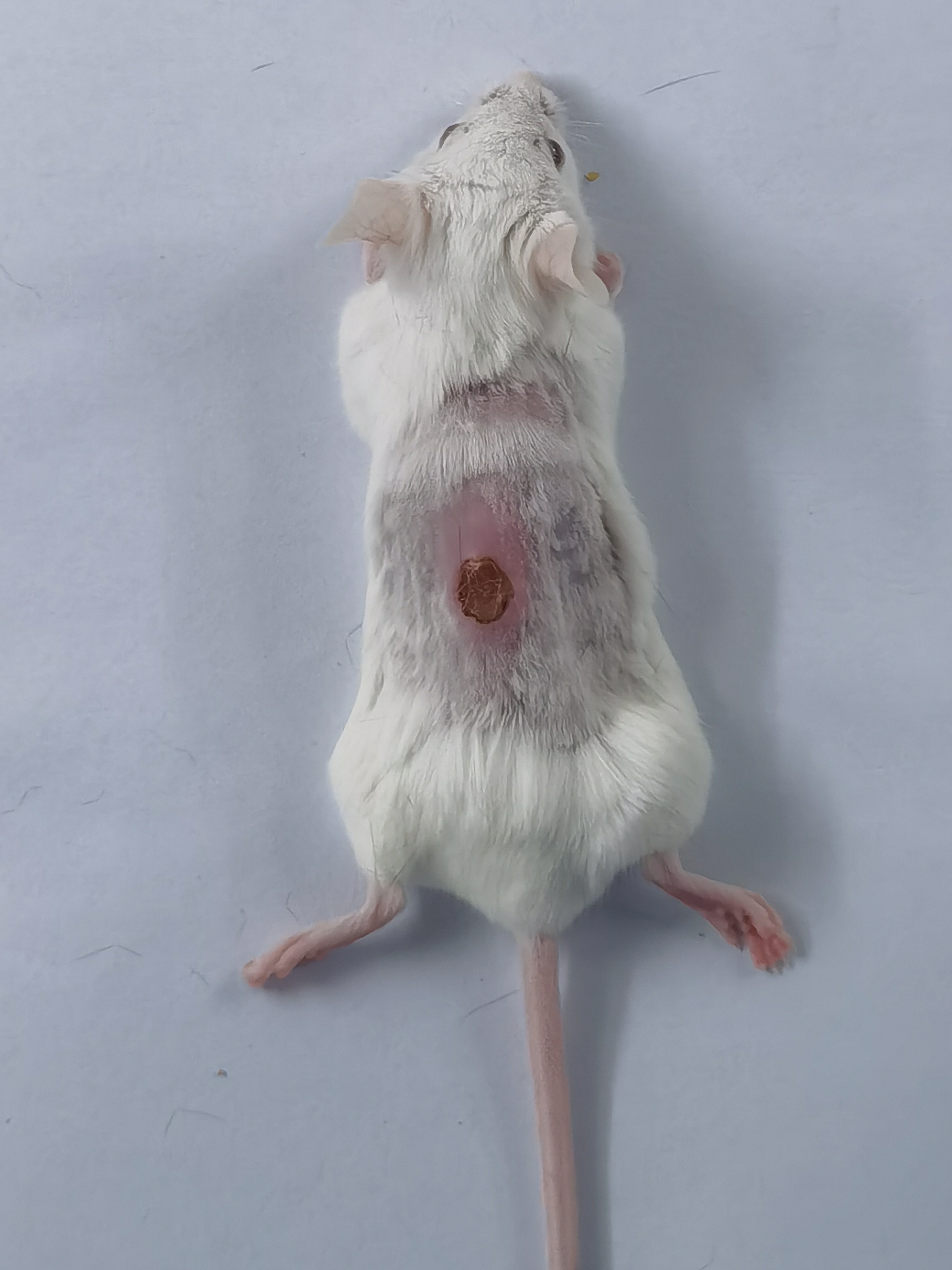

Supplement: Supplementary file 11 — Source data Fig. 6 [file 44321_2026_418_MOESM11_ESM.zip › Figure 6/Data-Figure 6B/Day 2/2-5.jpg]

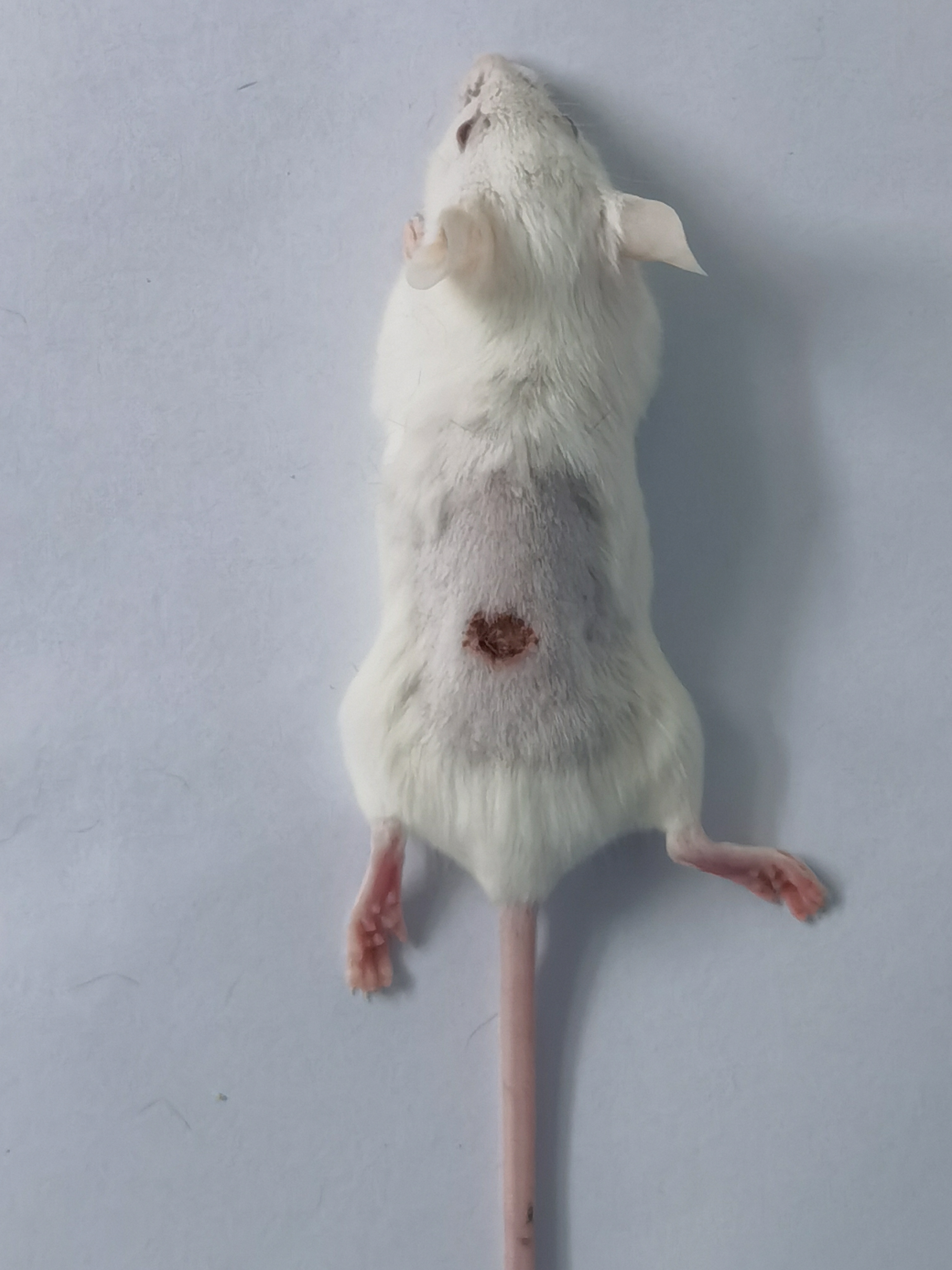

Supplement: Supplementary file 11 — Source data Fig. 6 [file 44321_2026_418_MOESM11_ESM.zip › Figure 6/Data-Figure 6B/Day 2/2-4.jpg]

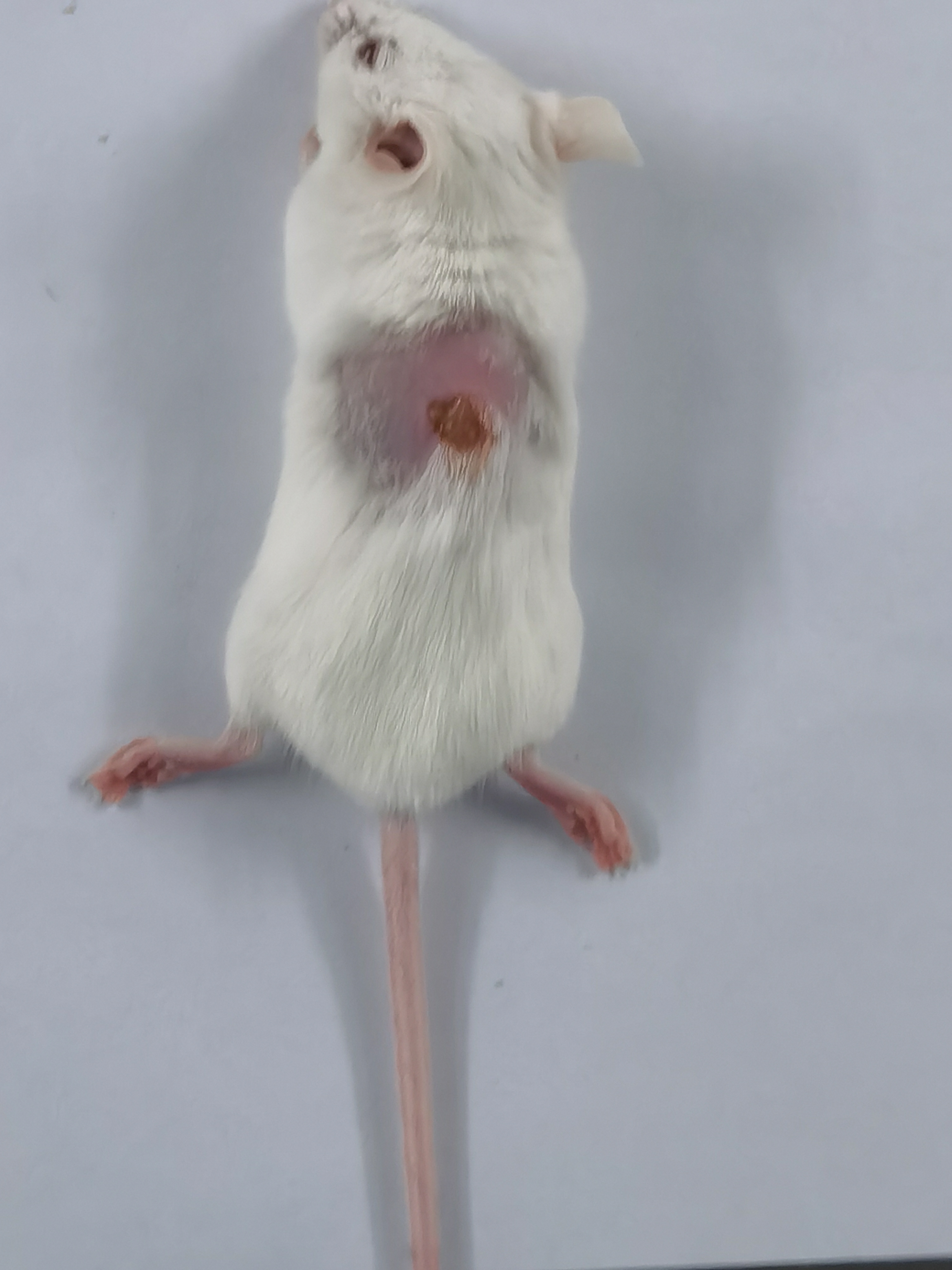

Supplement: Supplementary file 11 — Source data Fig. 6 [file 44321_2026_418_MOESM11_ESM.zip › Figure 6/Data-Figure 6B/Day 2/4-2.jpg]

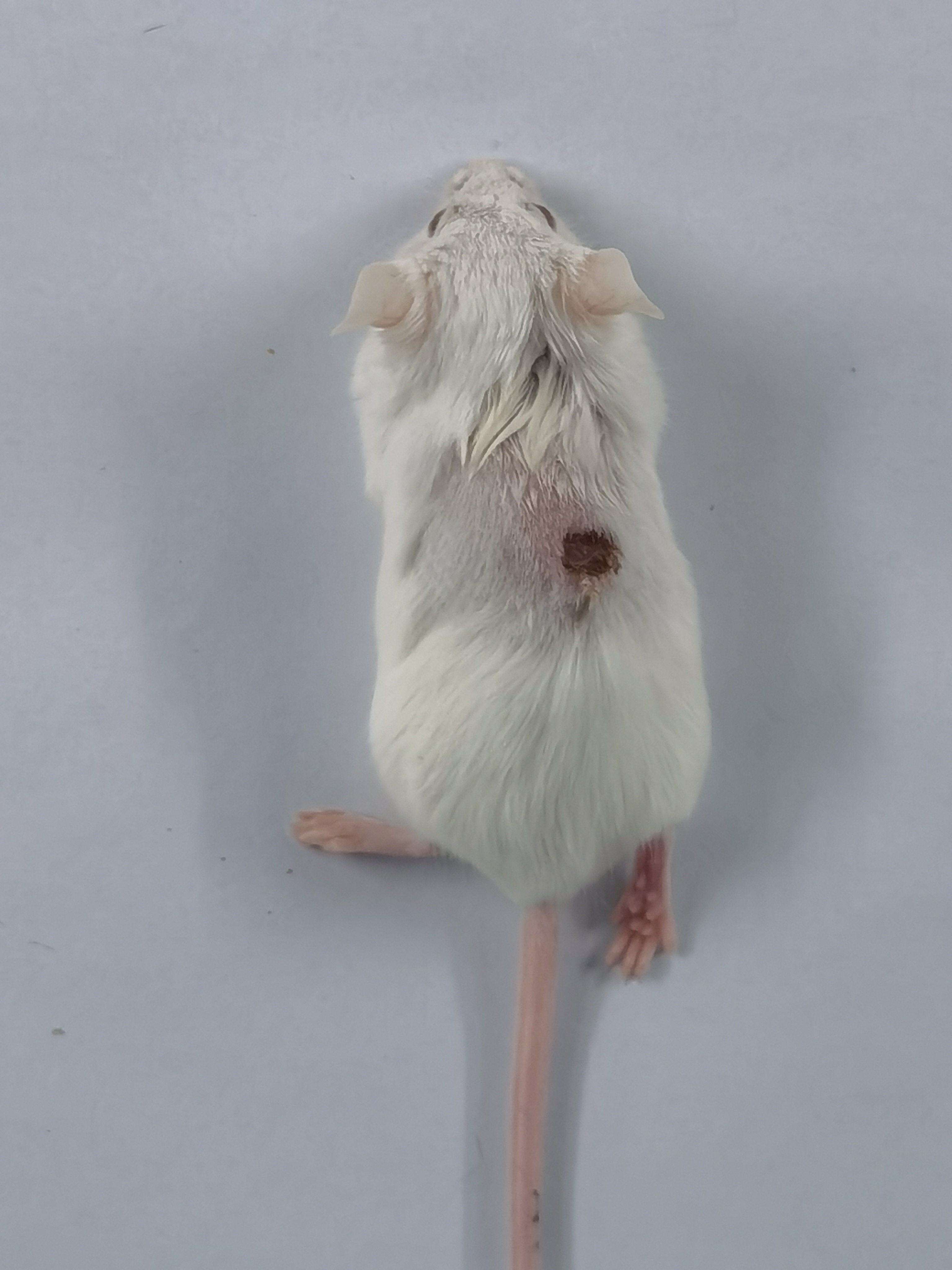

Supplement: Supplementary file 11 — Source data Fig. 6 [file 44321_2026_418_MOESM11_ESM.zip › Figure 6/Data-Figure 6B/Day 2/3-3.jpg]

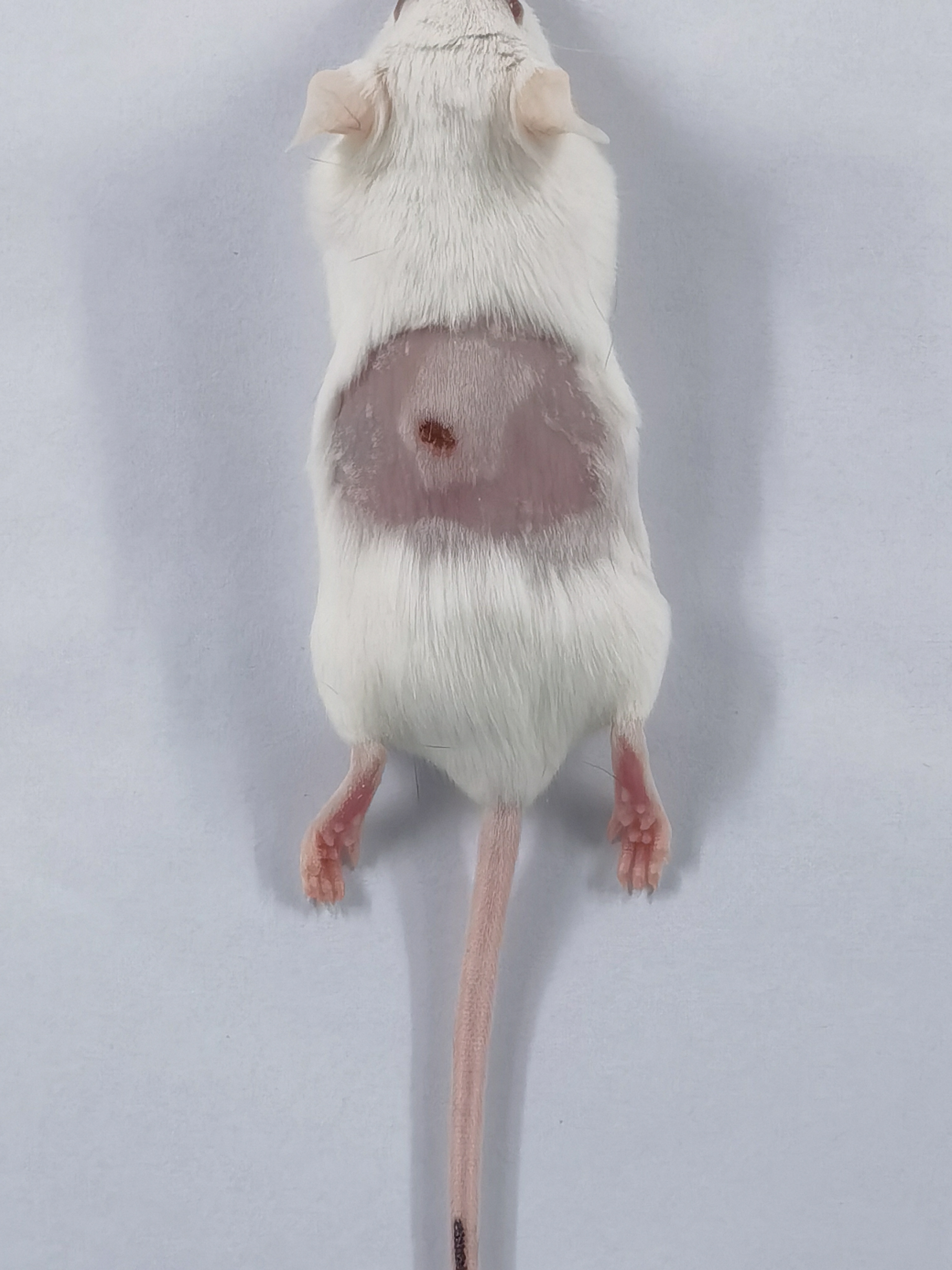

Supplement: Supplementary file 11 — Source data Fig. 6 [file 44321_2026_418_MOESM11_ESM.zip › Figure 6/Data-Figure 6B/Day 2/1-1.jpg]

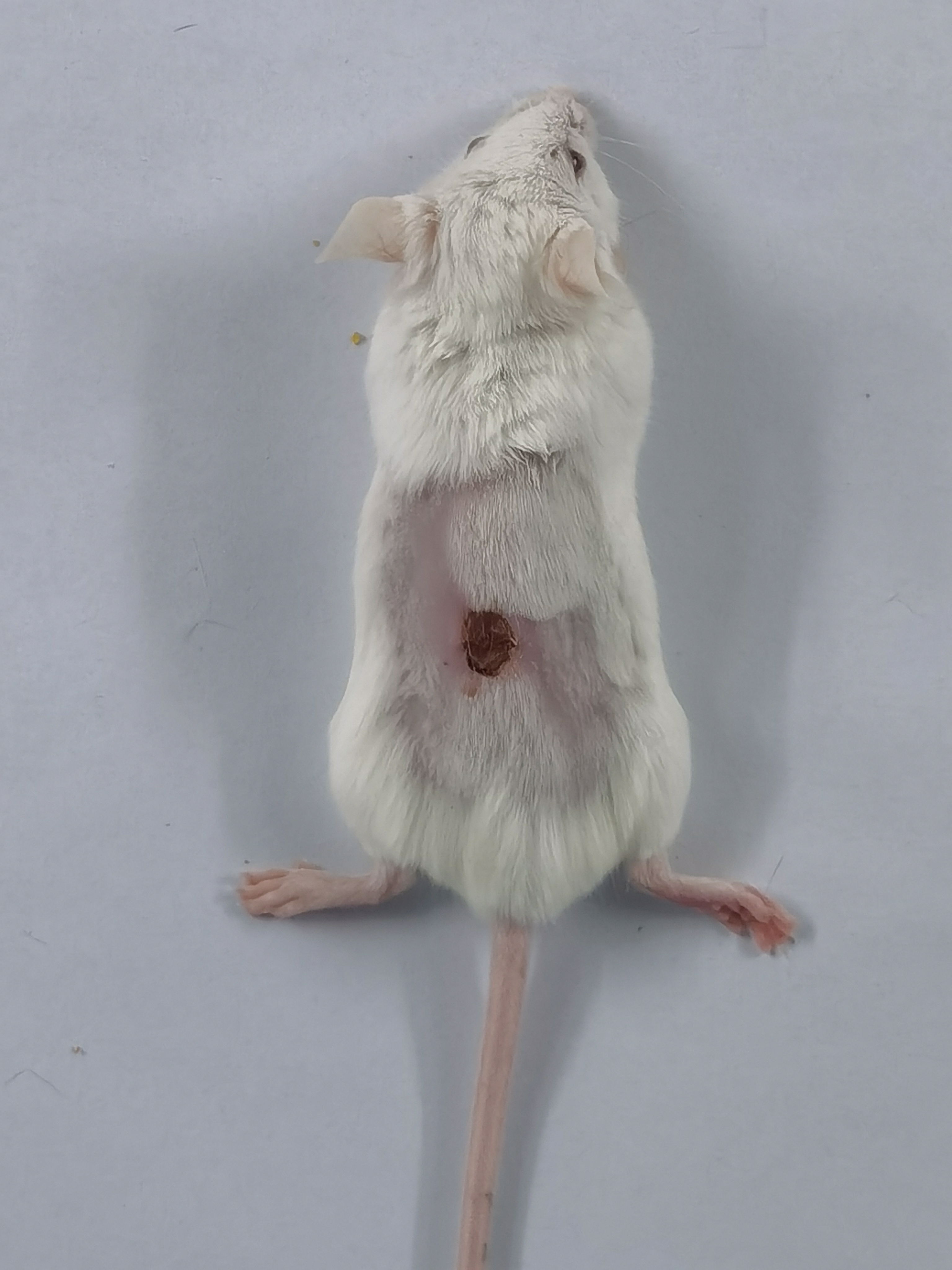

Supplement: Supplementary file 11 — Source data Fig. 6 [file 44321_2026_418_MOESM11_ESM.zip › Figure 6/Data-Figure 6B/Day 2/3-2.jpg]

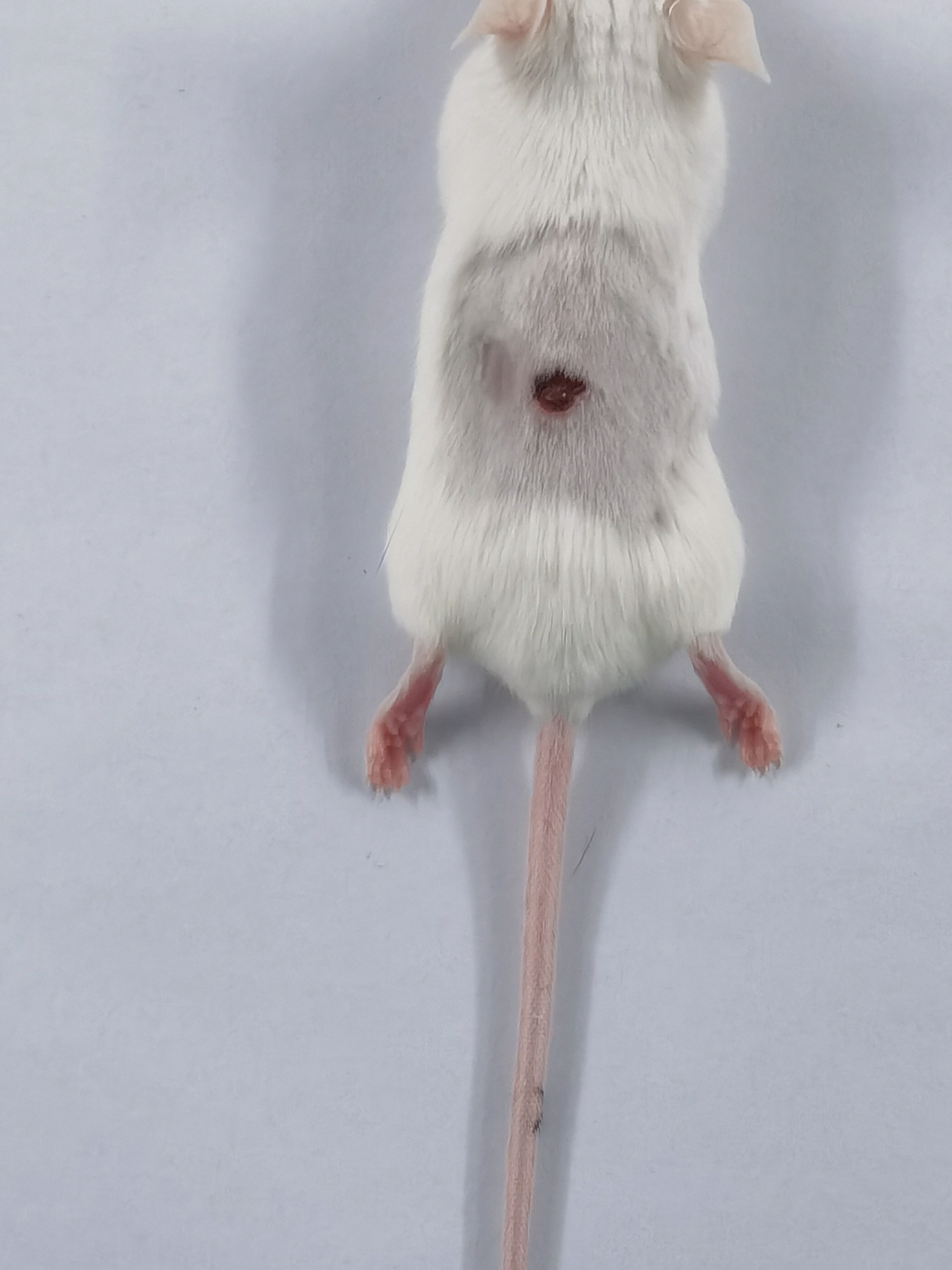

Supplement: Supplementary file 11 — Source data Fig. 6 [file 44321_2026_418_MOESM11_ESM.zip › Figure 6/Data-Figure 6B/Day 2/1-2.jpg]

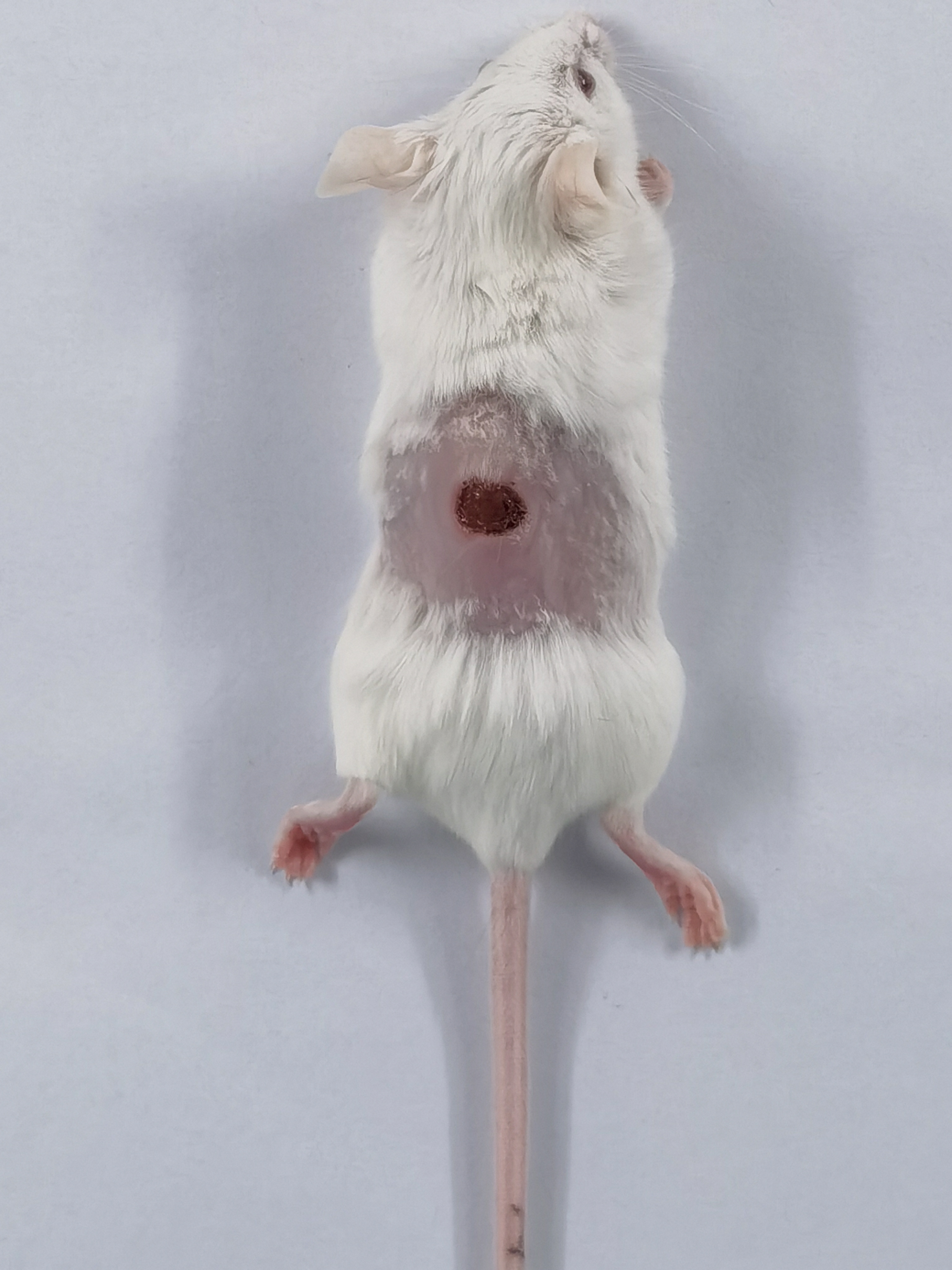

Supplement: Supplementary file 11 — Source data Fig. 6 [file 44321_2026_418_MOESM11_ESM.zip › Figure 6/Data-Figure 6B/Day 2/1-3.jpg]

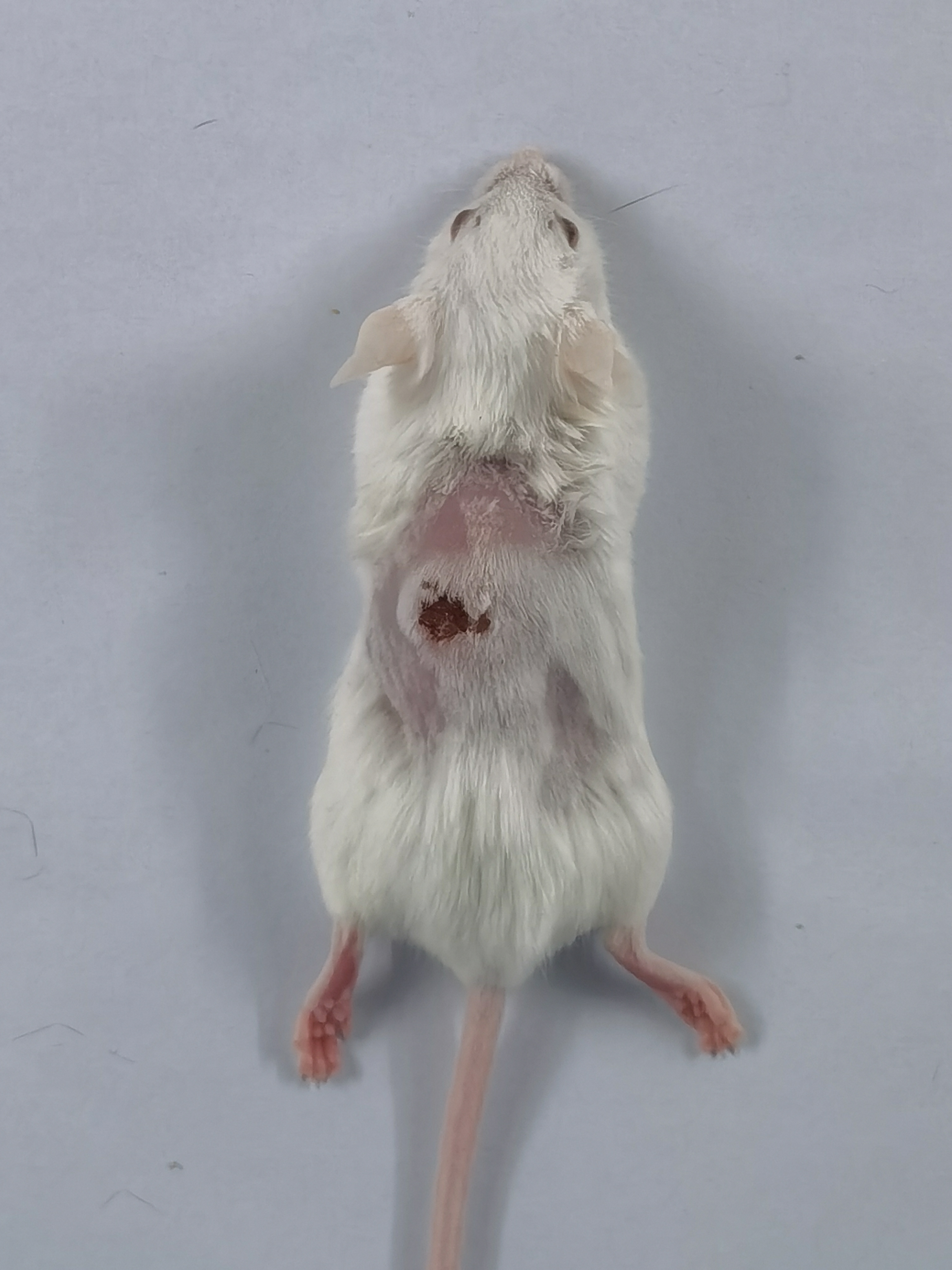

Supplement: Supplementary file 11 — Source data Fig. 6 [file 44321_2026_418_MOESM11_ESM.zip › Figure 6/Data-Figure 6B/Day 2/3-1.jpg]

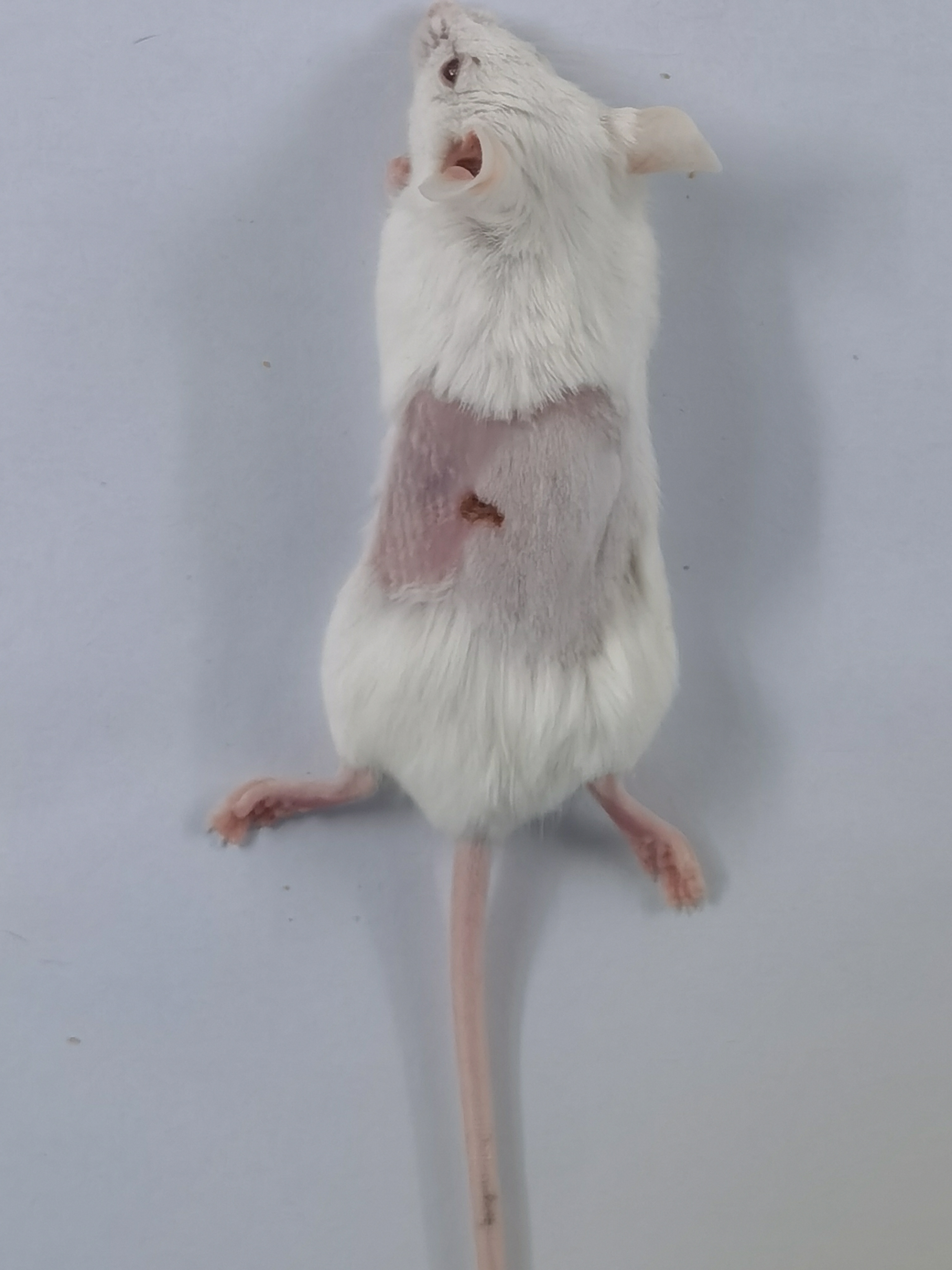

Supplement: Supplementary file 11 — Source data Fig. 6 [file 44321_2026_418_MOESM11_ESM.zip › Figure 6/Data-Figure 6B/Day 2/3-5.jpg]

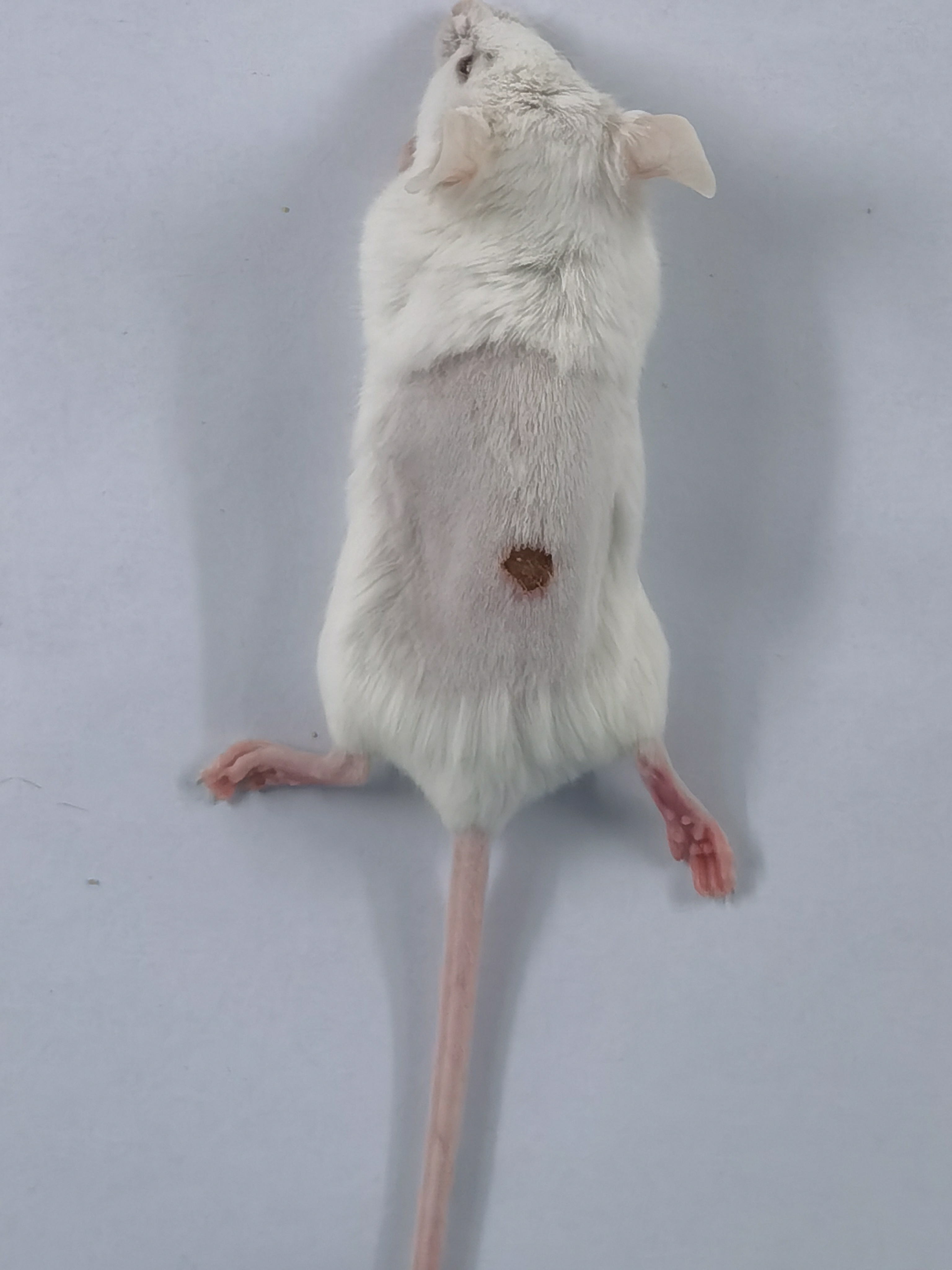

Supplement: Supplementary file 11 — Source data Fig. 6 [file 44321_2026_418_MOESM11_ESM.zip › Figure 6/Data-Figure 6B/Day 2/3-4.jpg]

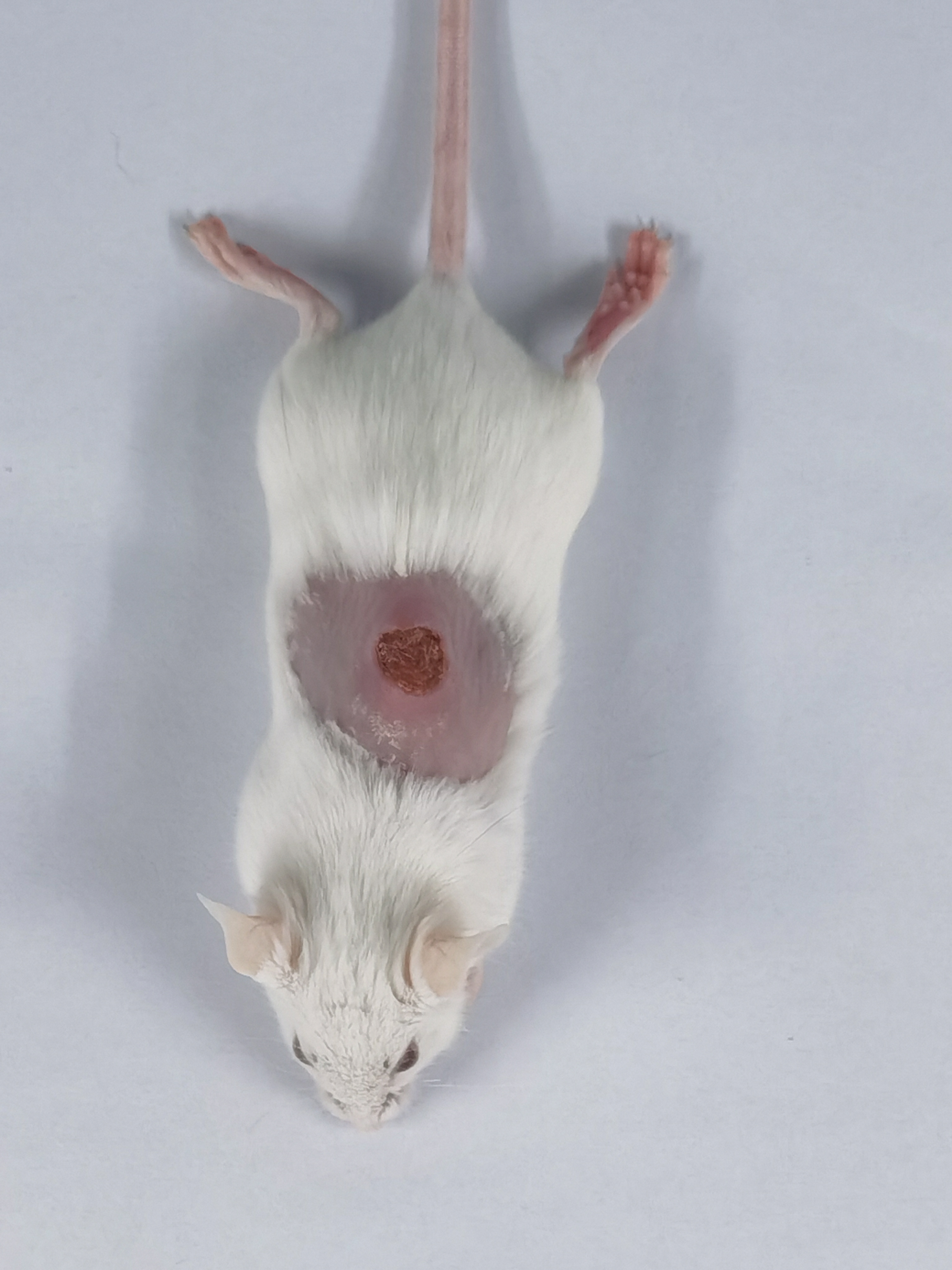

Supplement: Supplementary file 11 — Source data Fig. 6 [file 44321_2026_418_MOESM11_ESM.zip › Figure 6/Data-Figure 6B/Day 2/1-4.jpg]

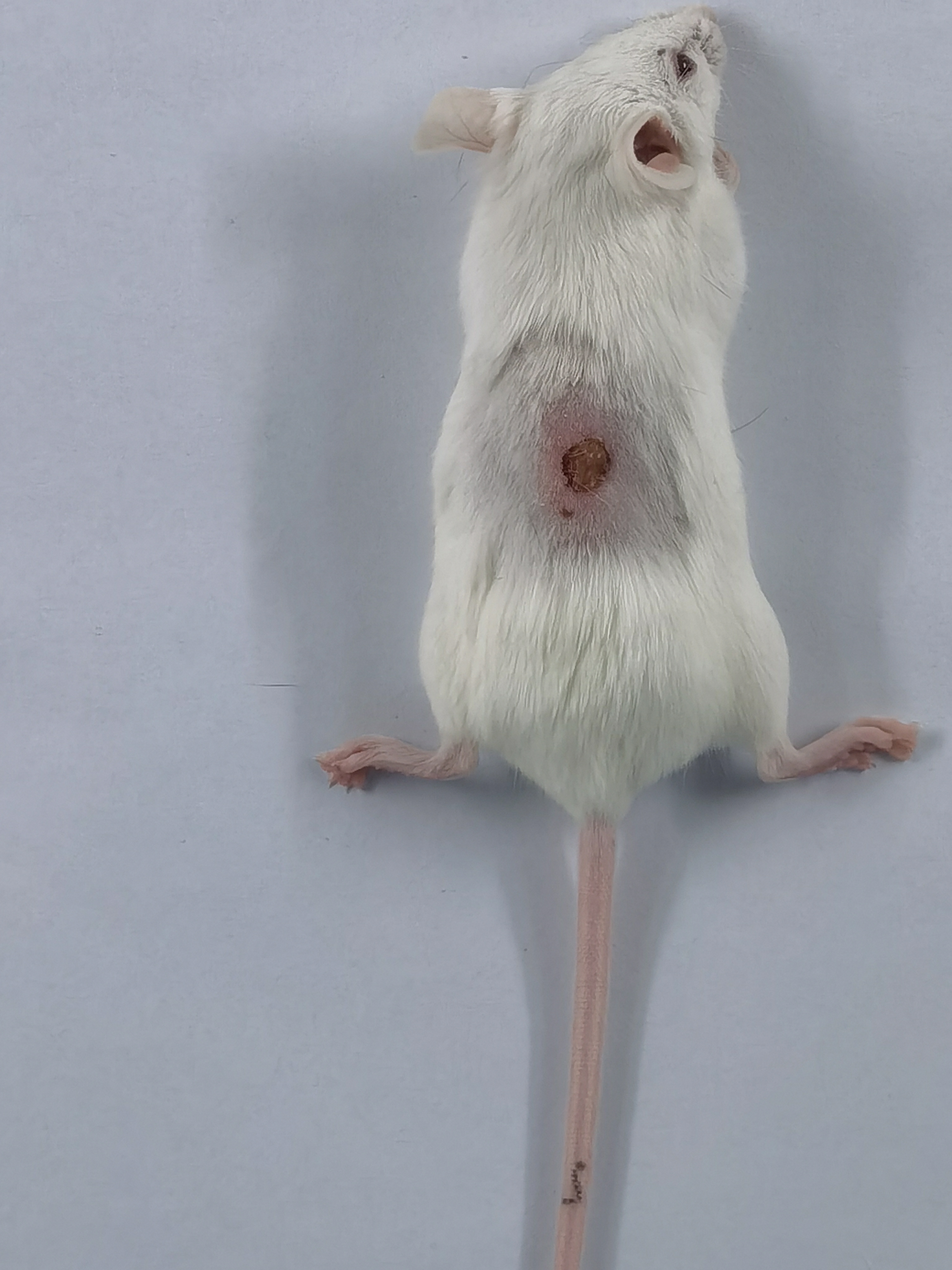

Supplement: Supplementary file 11 — Source data Fig. 6 [file 44321_2026_418_MOESM11_ESM.zip › Figure 6/Data-Figure 6B/Day 2/1-5.jpg]

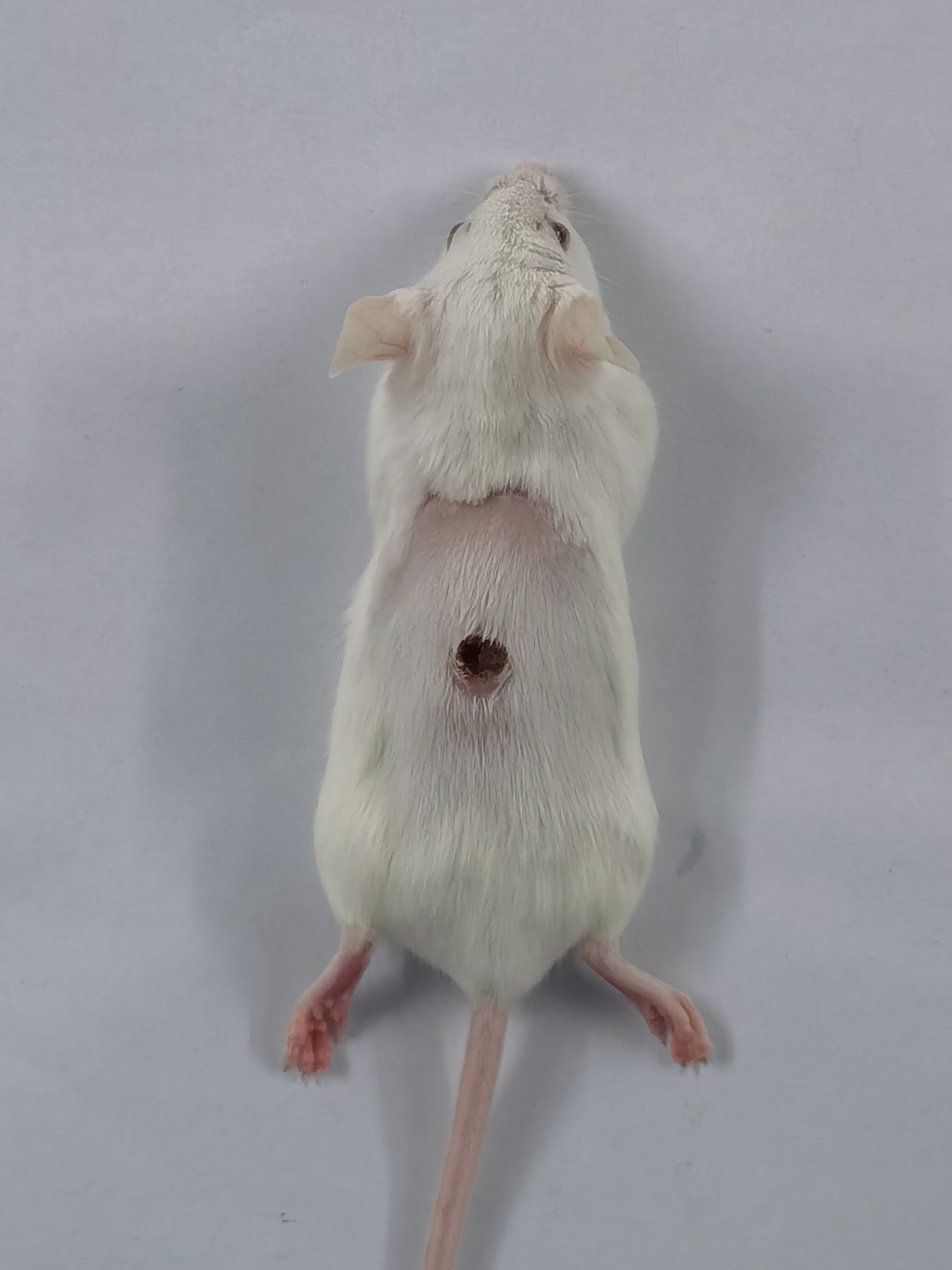

Supplement: Supplementary file 11 — Source data Fig. 6 [file 44321_2026_418_MOESM11_ESM.zip › Figure 6/Data-Figure 6B/Day 5/2-1.jpg]

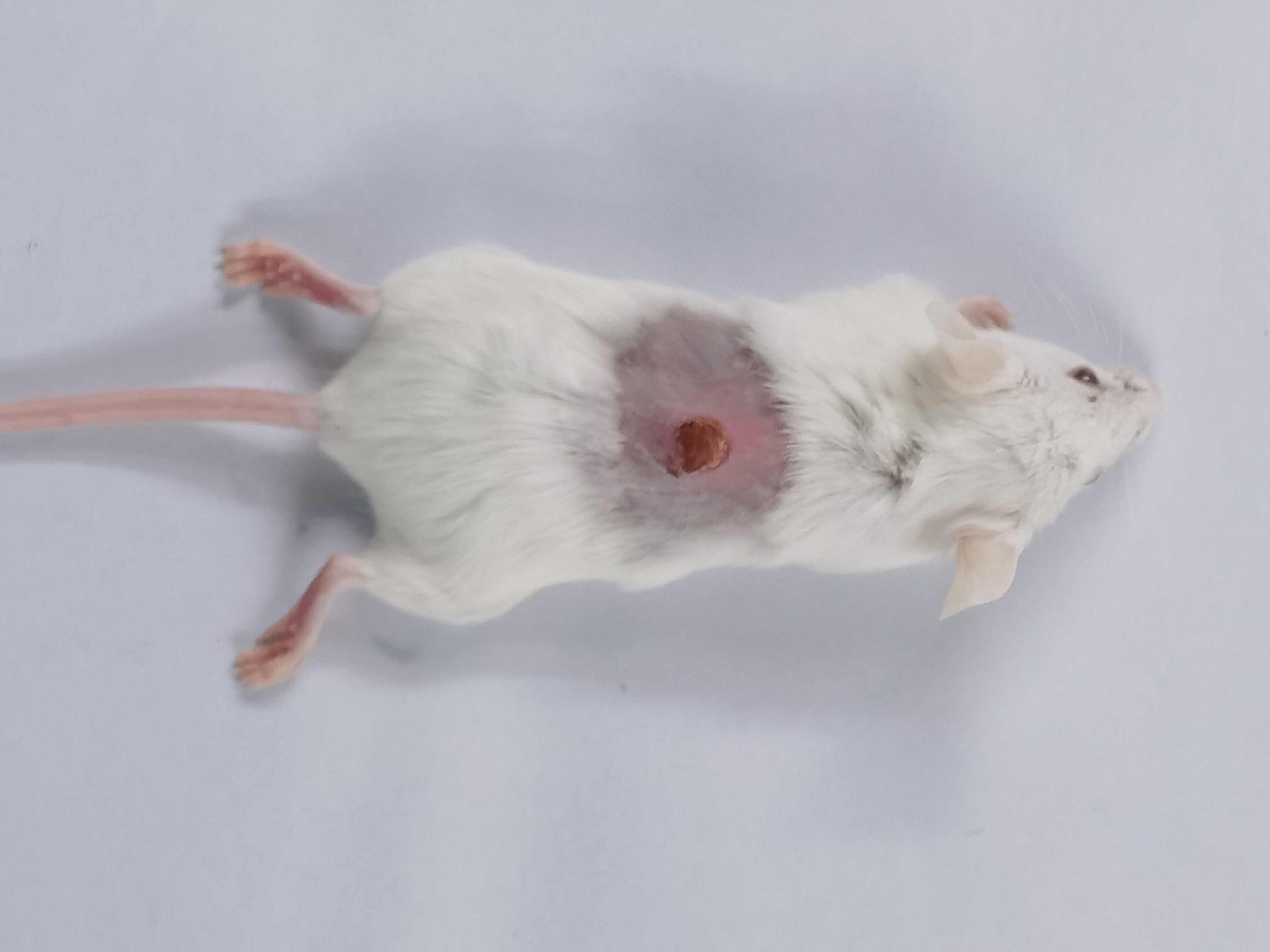

Supplement: Supplementary file 11 — Source data Fig. 6 [file 44321_2026_418_MOESM11_ESM.zip › Figure 6/Data-Figure 6B/Day 5/4-5.jpg]

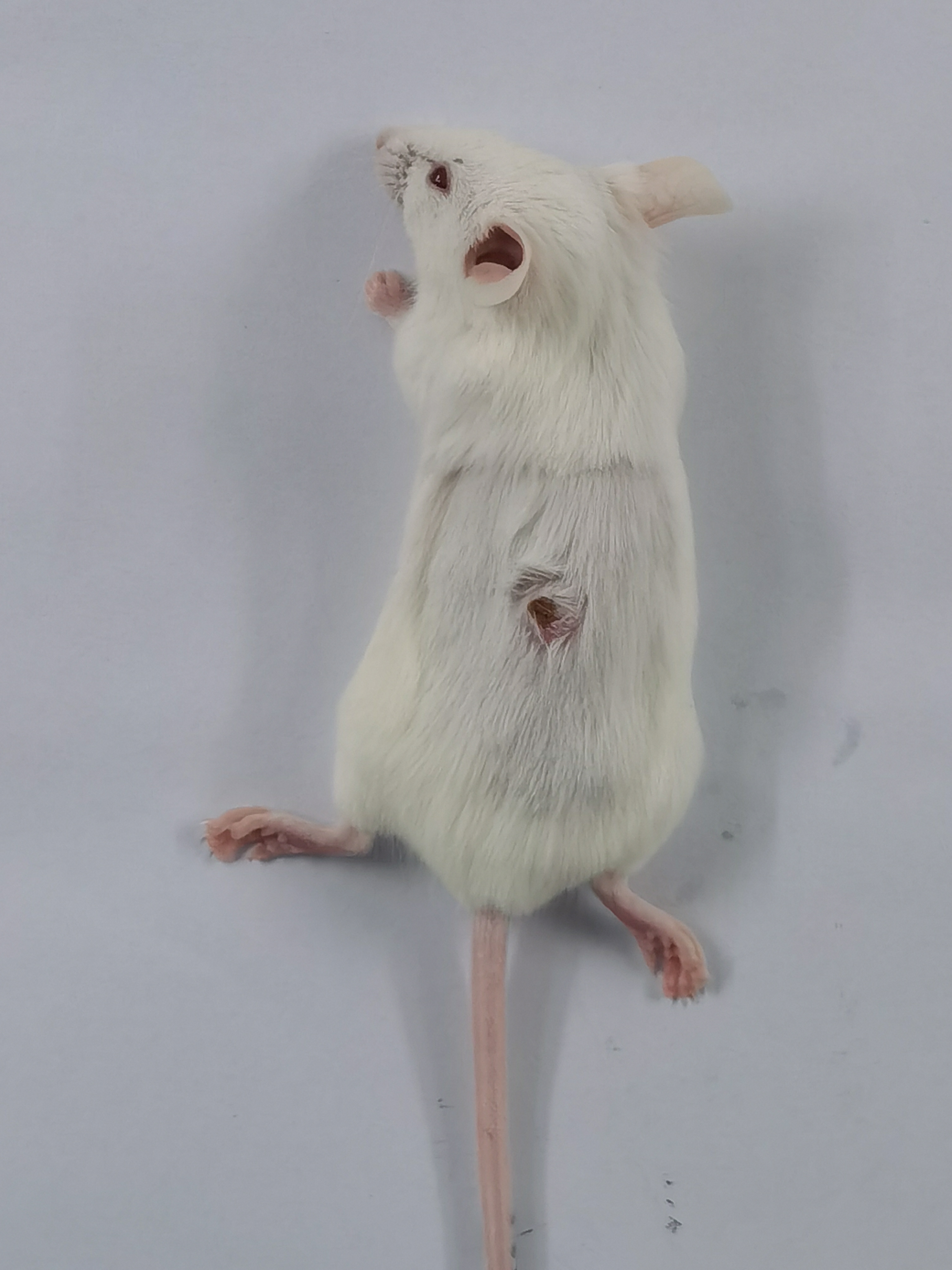

Supplement: Supplementary file 11 — Source data Fig. 6 [file 44321_2026_418_MOESM11_ESM.zip › Figure 6/Data-Figure 6B/Day 5/2-3.jpg]

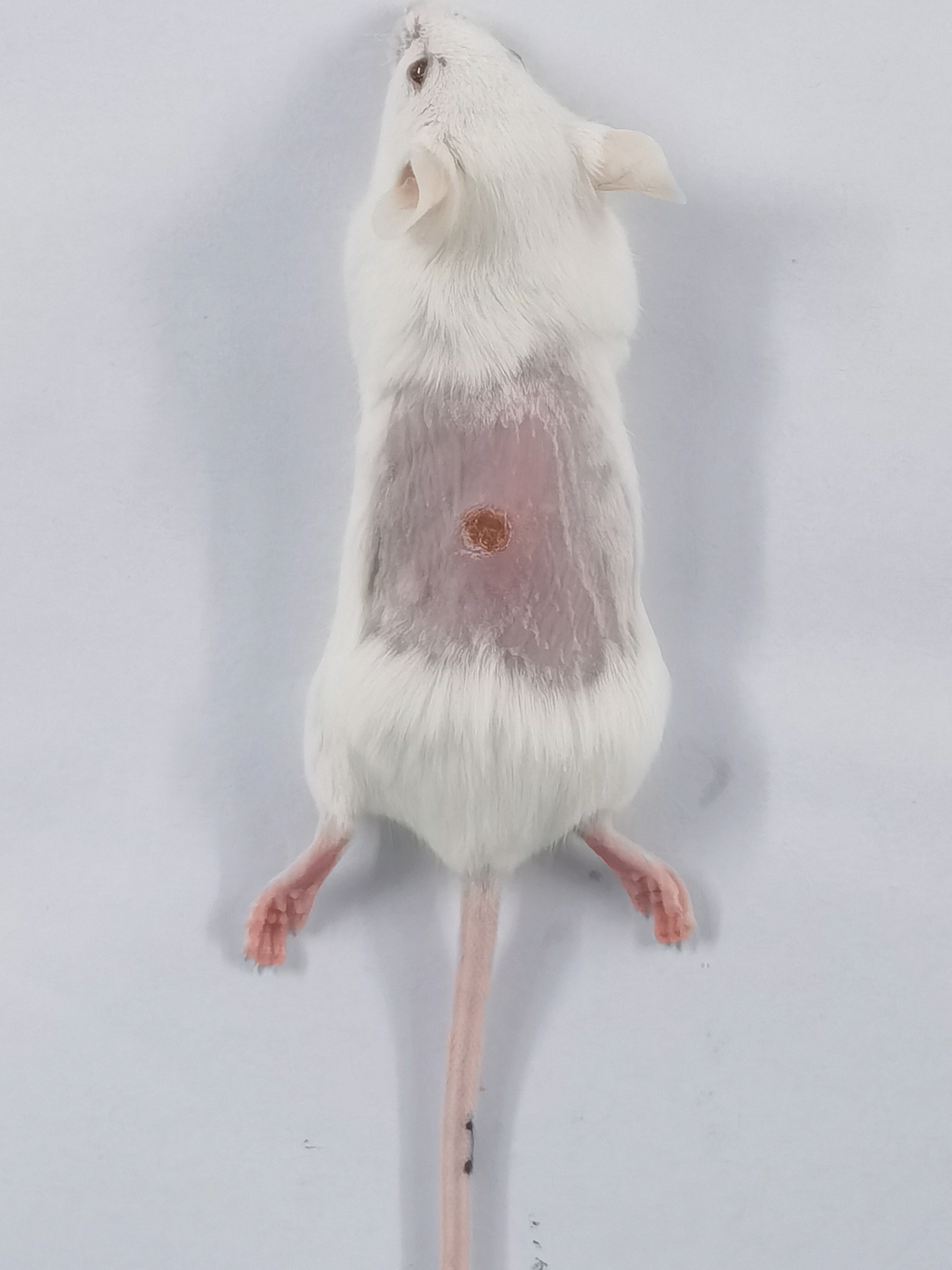

Supplement: Supplementary file 11 — Source data Fig. 6 [file 44321_2026_418_MOESM11_ESM.zip › Figure 6/Data-Figure 6B/Day 5/2-2.jpg]

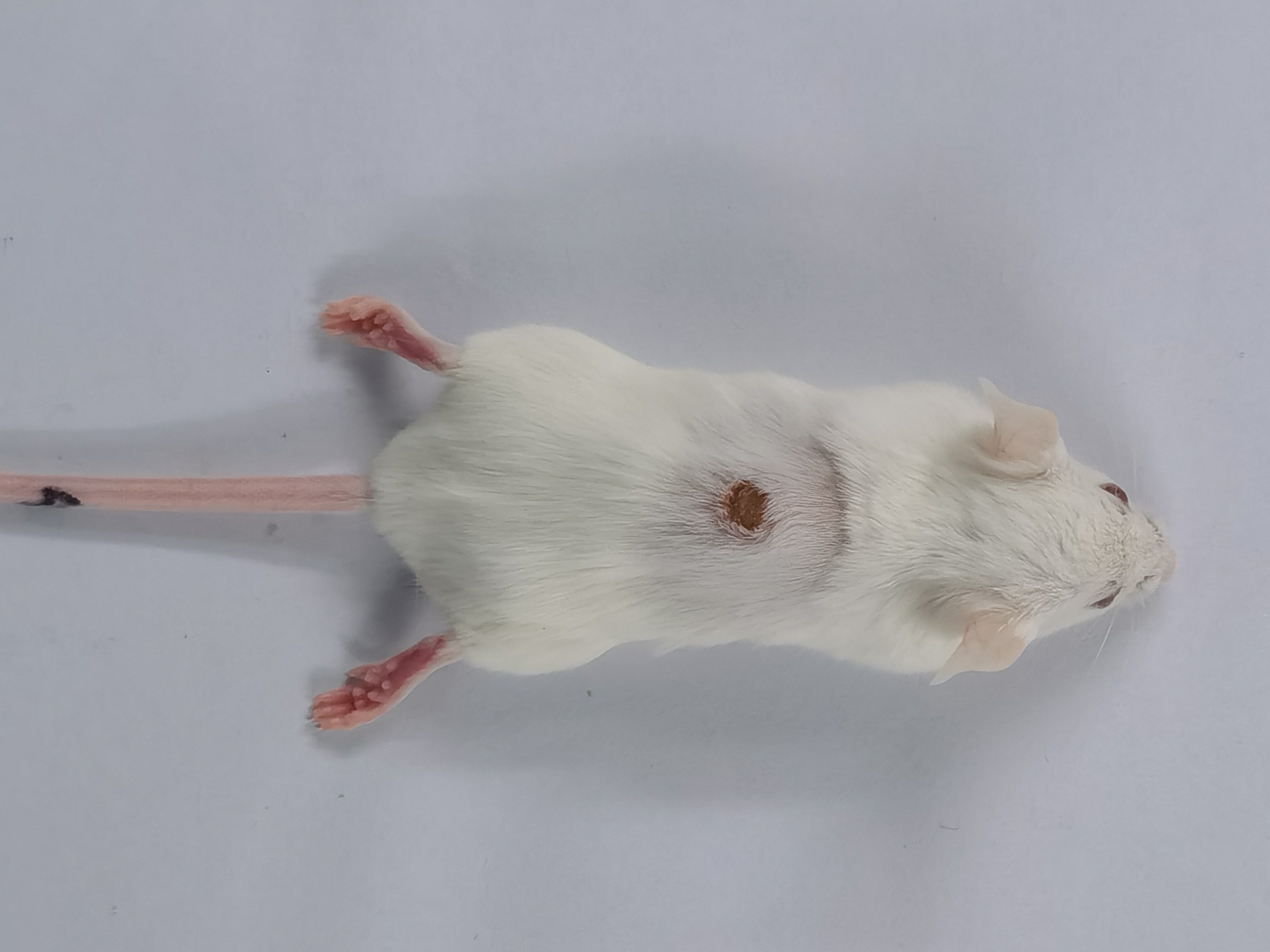

Supplement: Supplementary file 11 — Source data Fig. 6 [file 44321_2026_418_MOESM11_ESM.zip › Figure 6/Data-Figure 6B/Day 5/4-4.jpg]

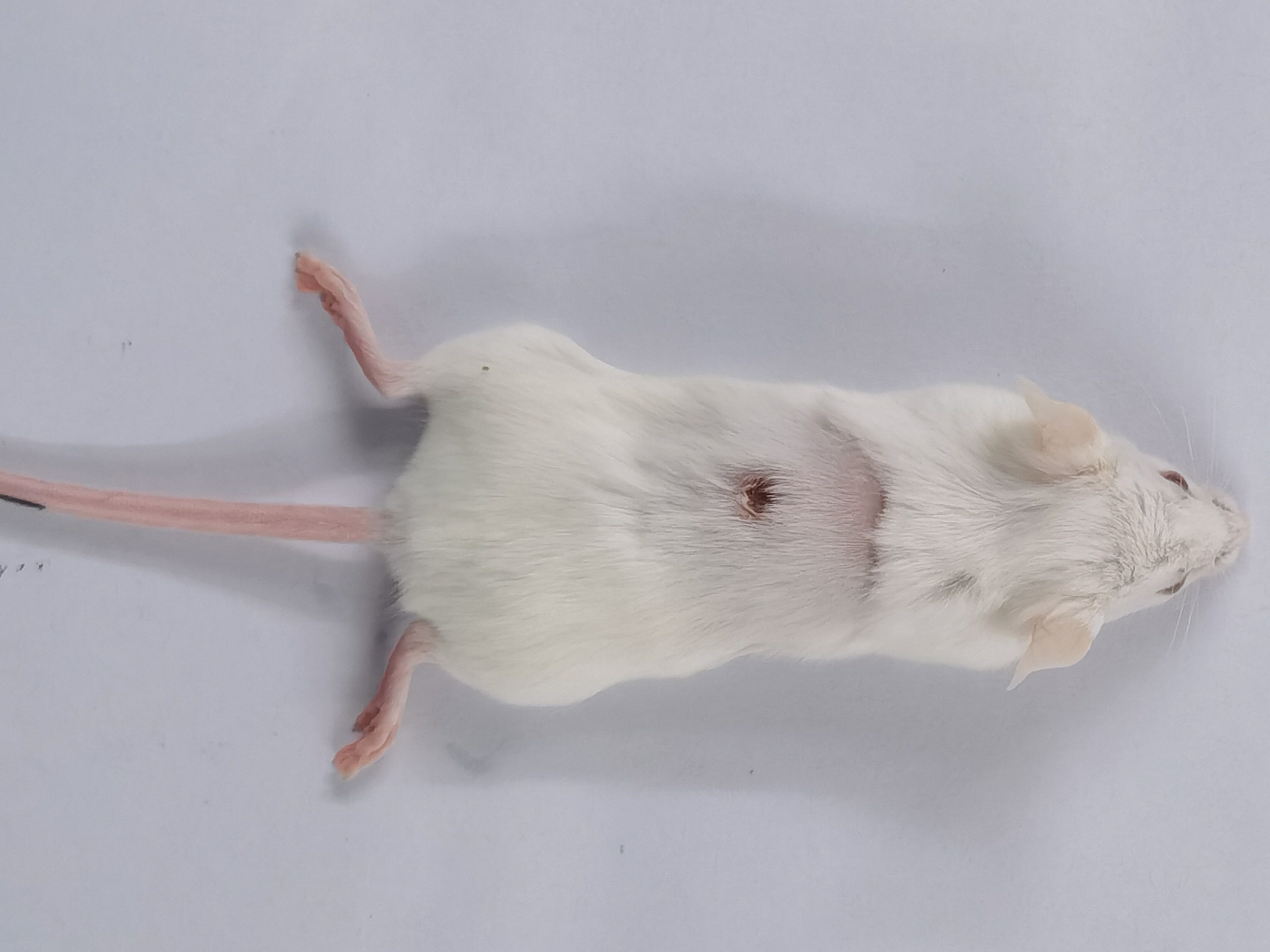

Supplement: Supplementary file 11 — Source data Fig. 6 [file 44321_2026_418_MOESM11_ESM.zip › Figure 6/Data-Figure 6B/Day 5/4-1.jpg]

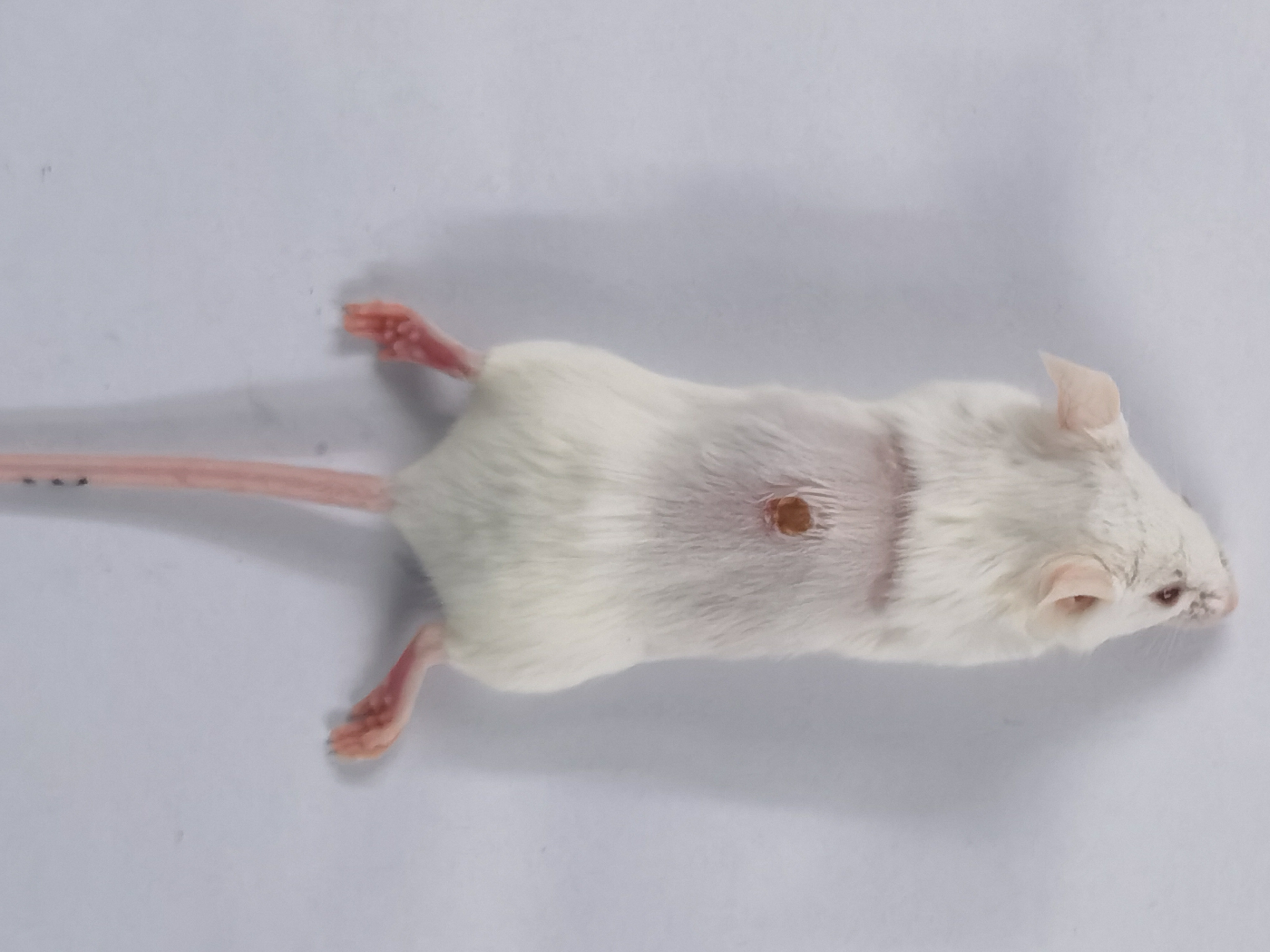

Supplement: Supplementary file 11 — Source data Fig. 6 [file 44321_2026_418_MOESM11_ESM.zip › Figure 6/Data-Figure 6B/Day 5/4-3.jpg]

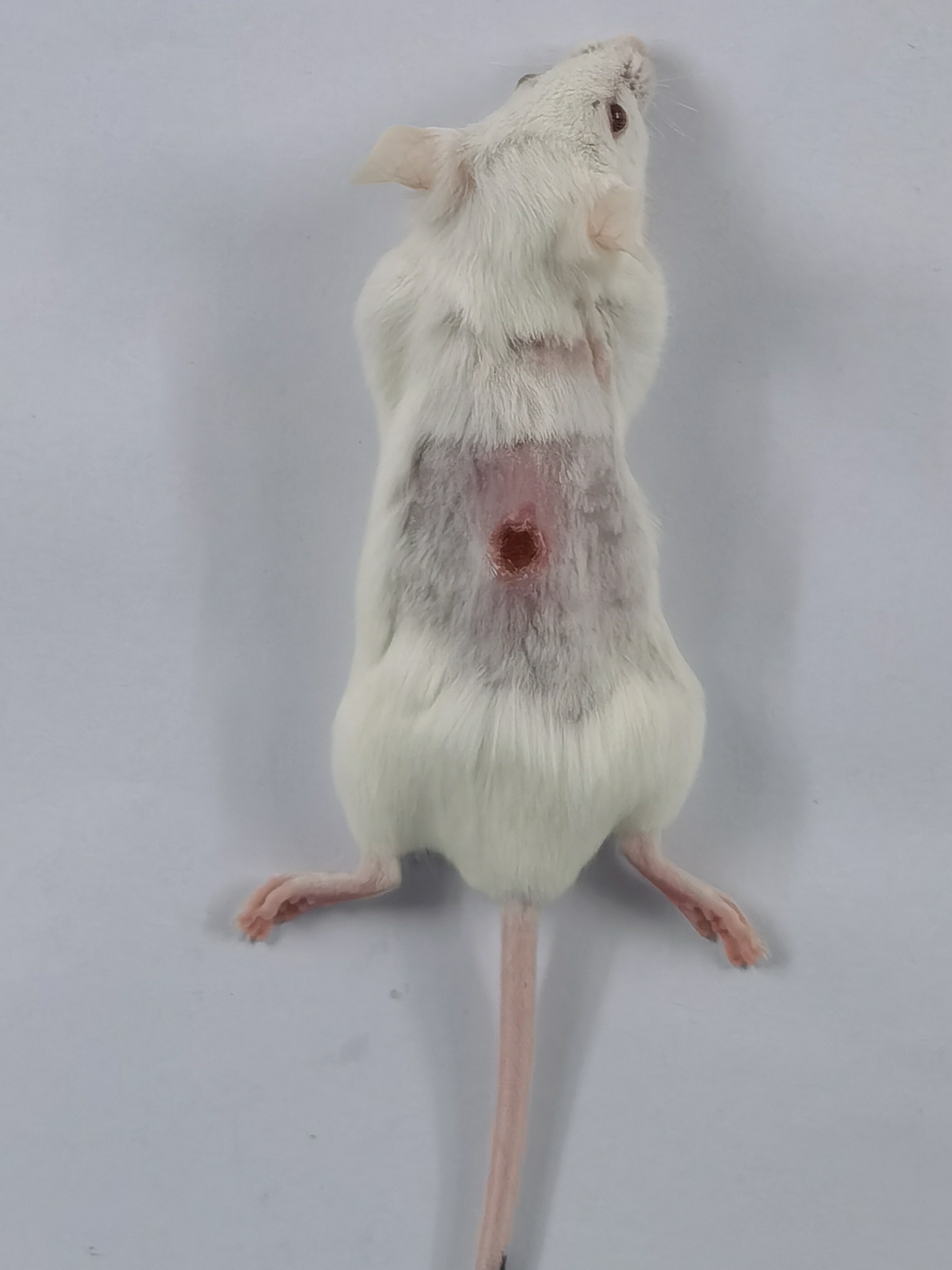

Supplement: Supplementary file 11 — Source data Fig. 6 [file 44321_2026_418_MOESM11_ESM.zip › Figure 6/Data-Figure 6B/Day 5/2-5.jpg]

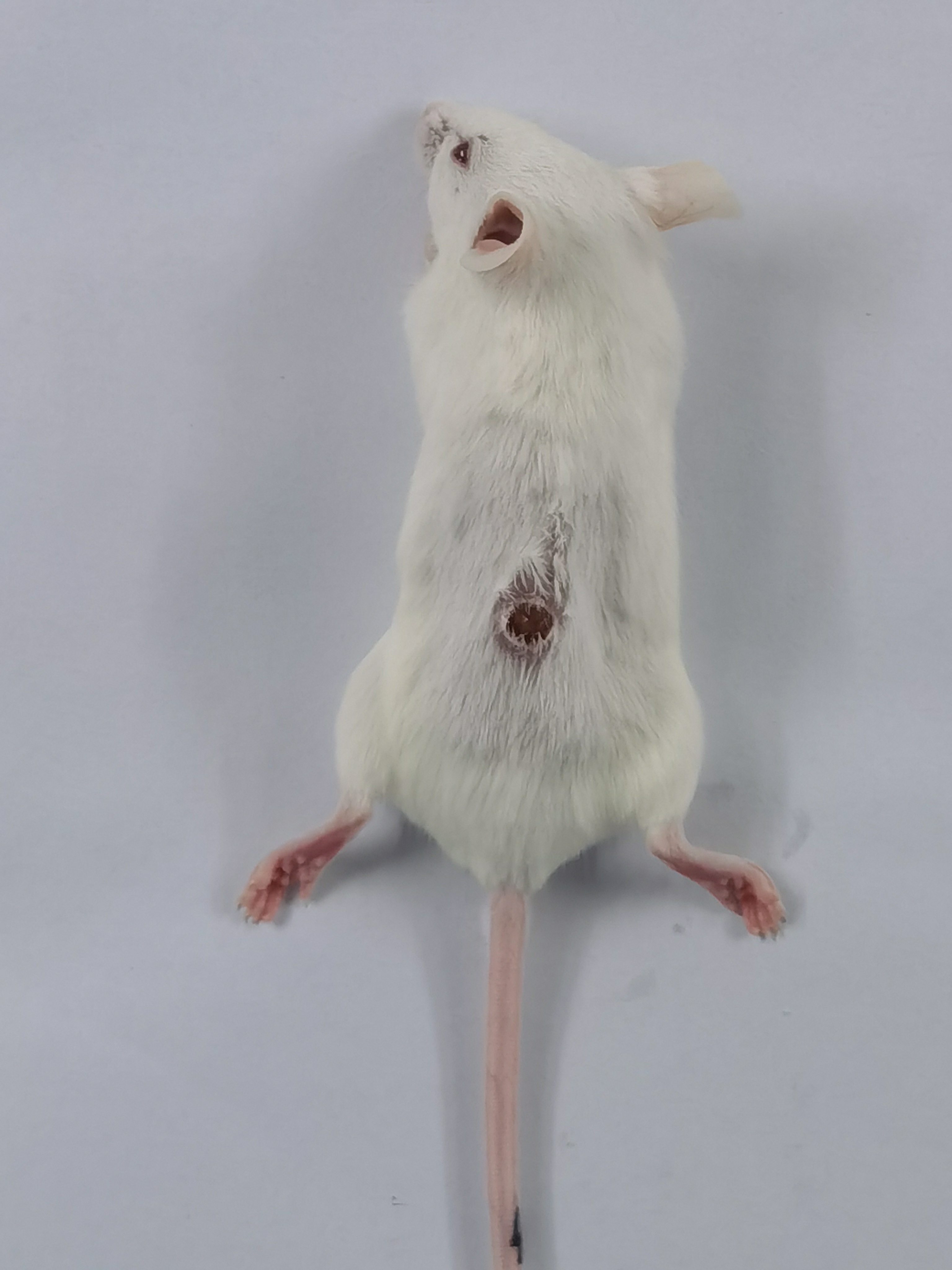

Supplement: Supplementary file 11 — Source data Fig. 6 [file 44321_2026_418_MOESM11_ESM.zip › Figure 6/Data-Figure 6B/Day 5/2-4.jpg]

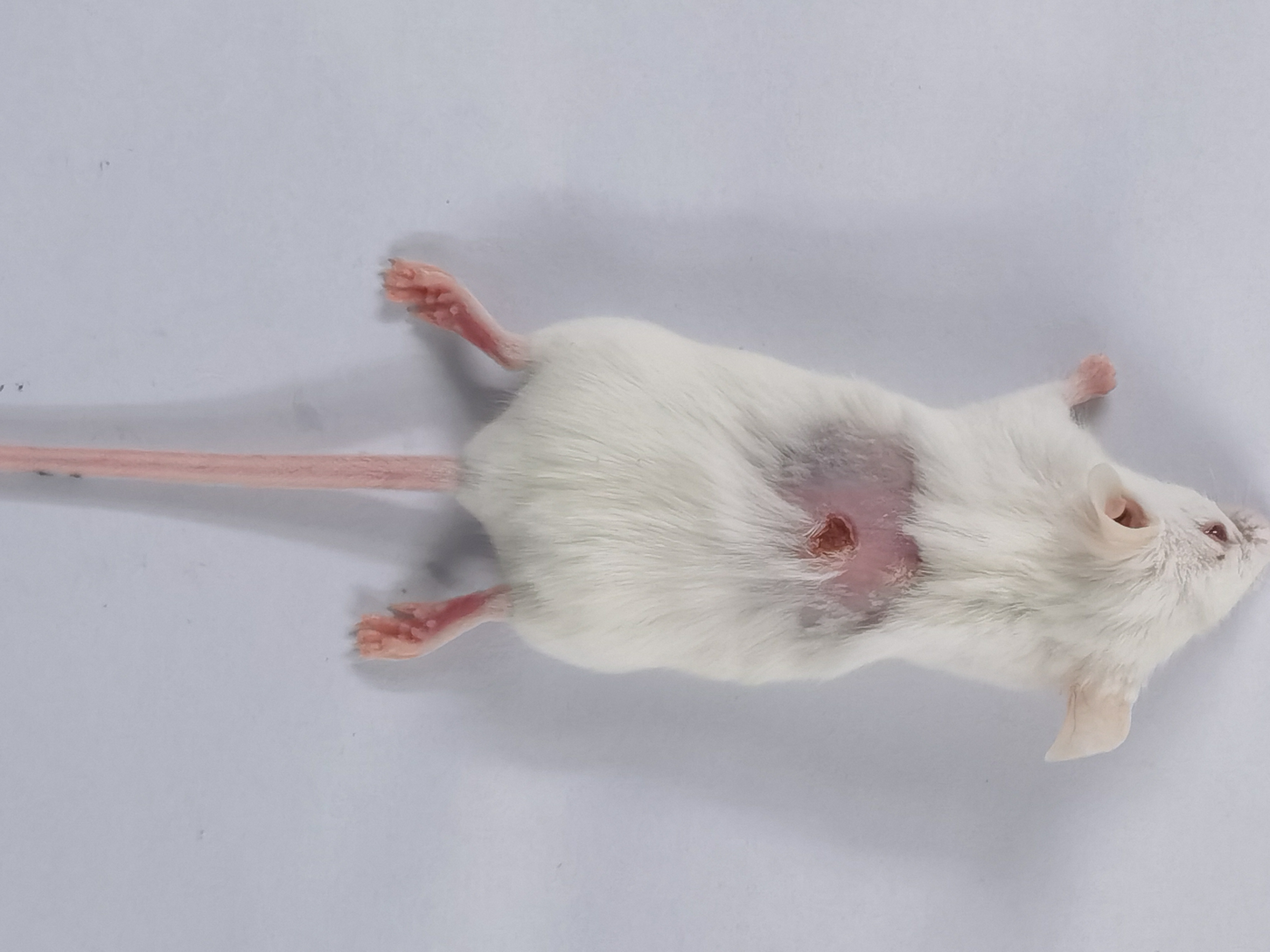

Supplement: Supplementary file 11 — Source data Fig. 6 [file 44321_2026_418_MOESM11_ESM.zip › Figure 6/Data-Figure 6B/Day 5/4-2.jpg]

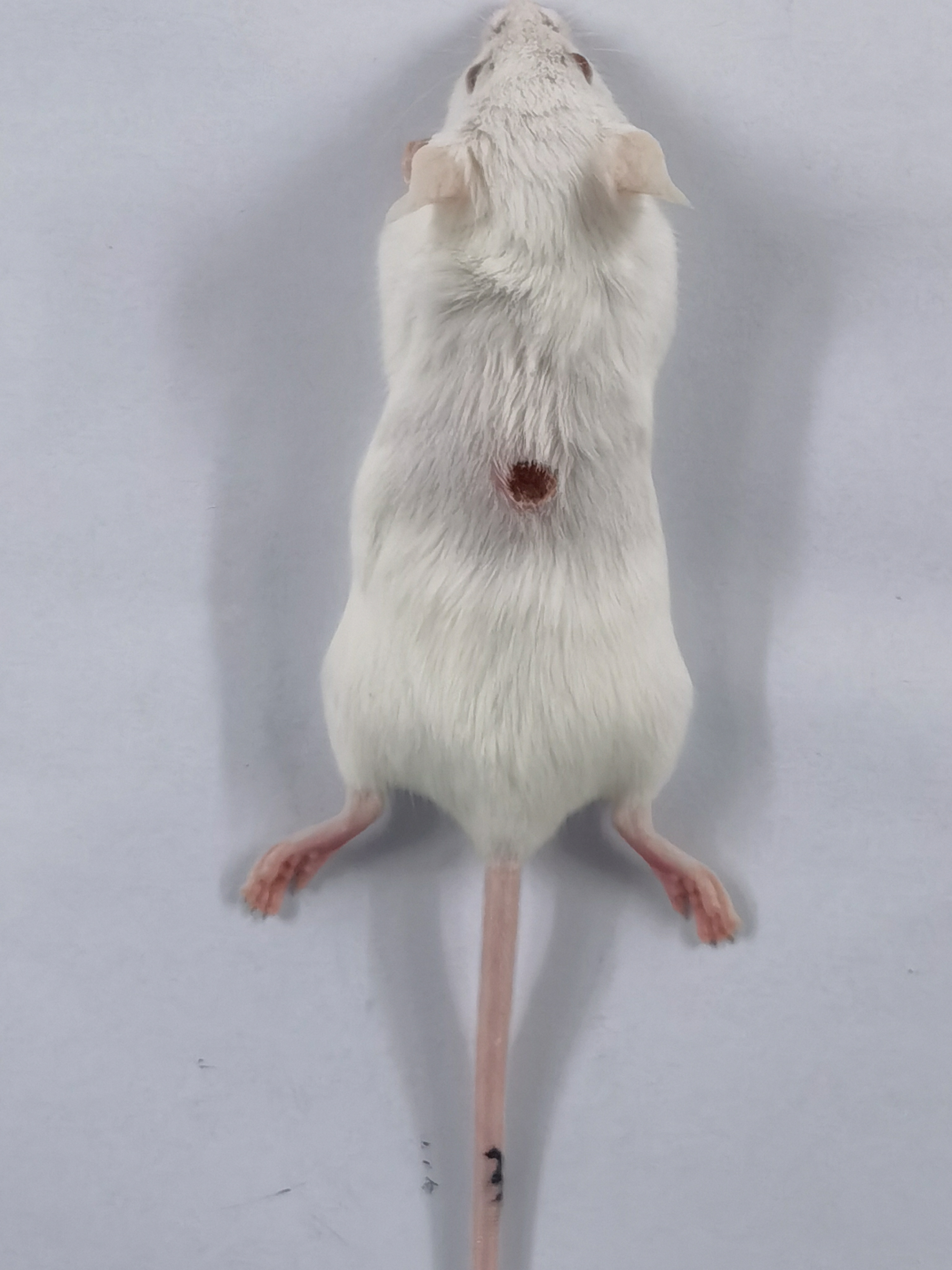

Supplement: Supplementary file 11 — Source data Fig. 6 [file 44321_2026_418_MOESM11_ESM.zip › Figure 6/Data-Figure 6B/Day 5/3-3.jpg]

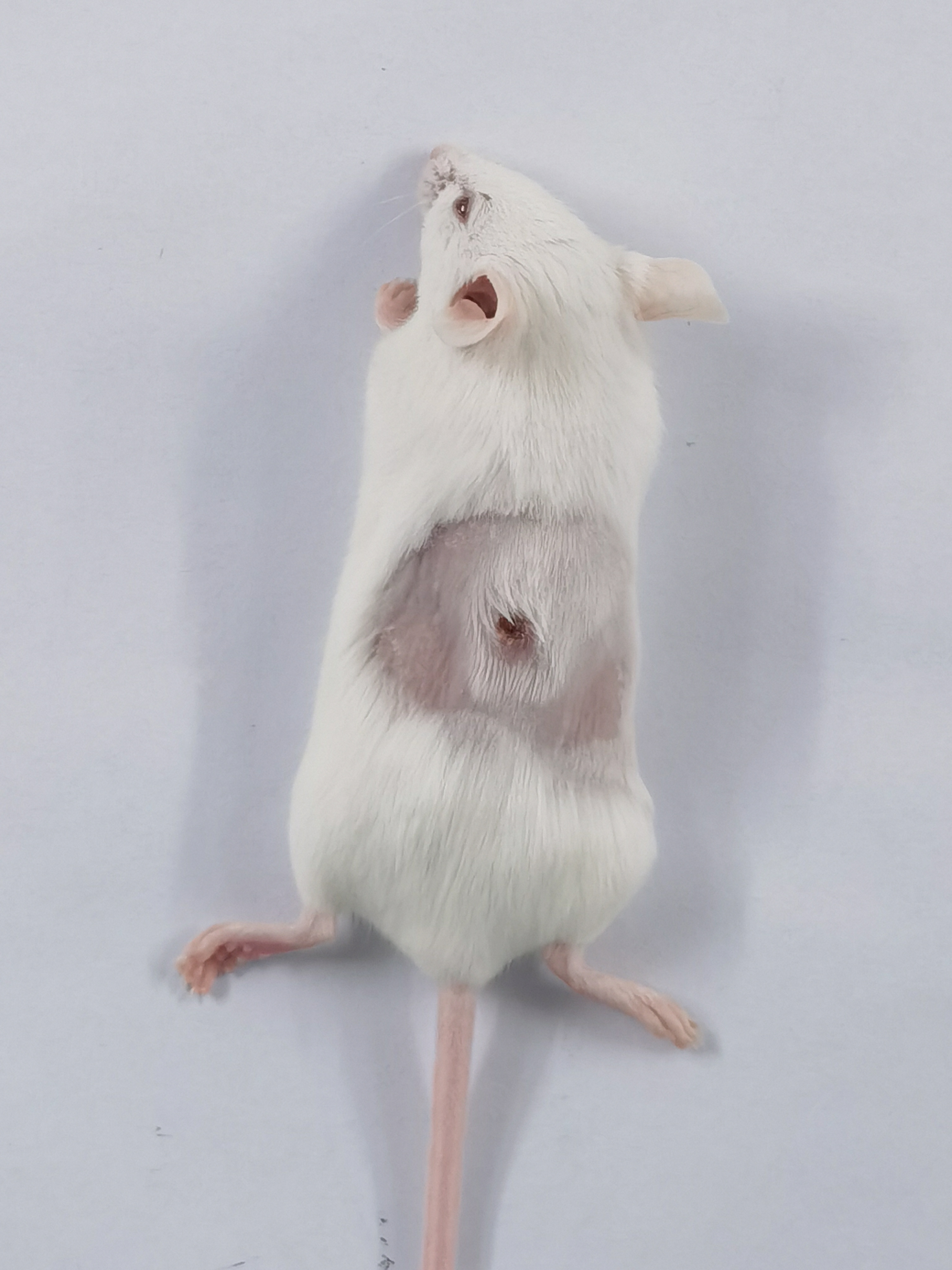

Supplement: Supplementary file 11 — Source data Fig. 6 [file 44321_2026_418_MOESM11_ESM.zip › Figure 6/Data-Figure 6B/Day 5/1-1.jpg]

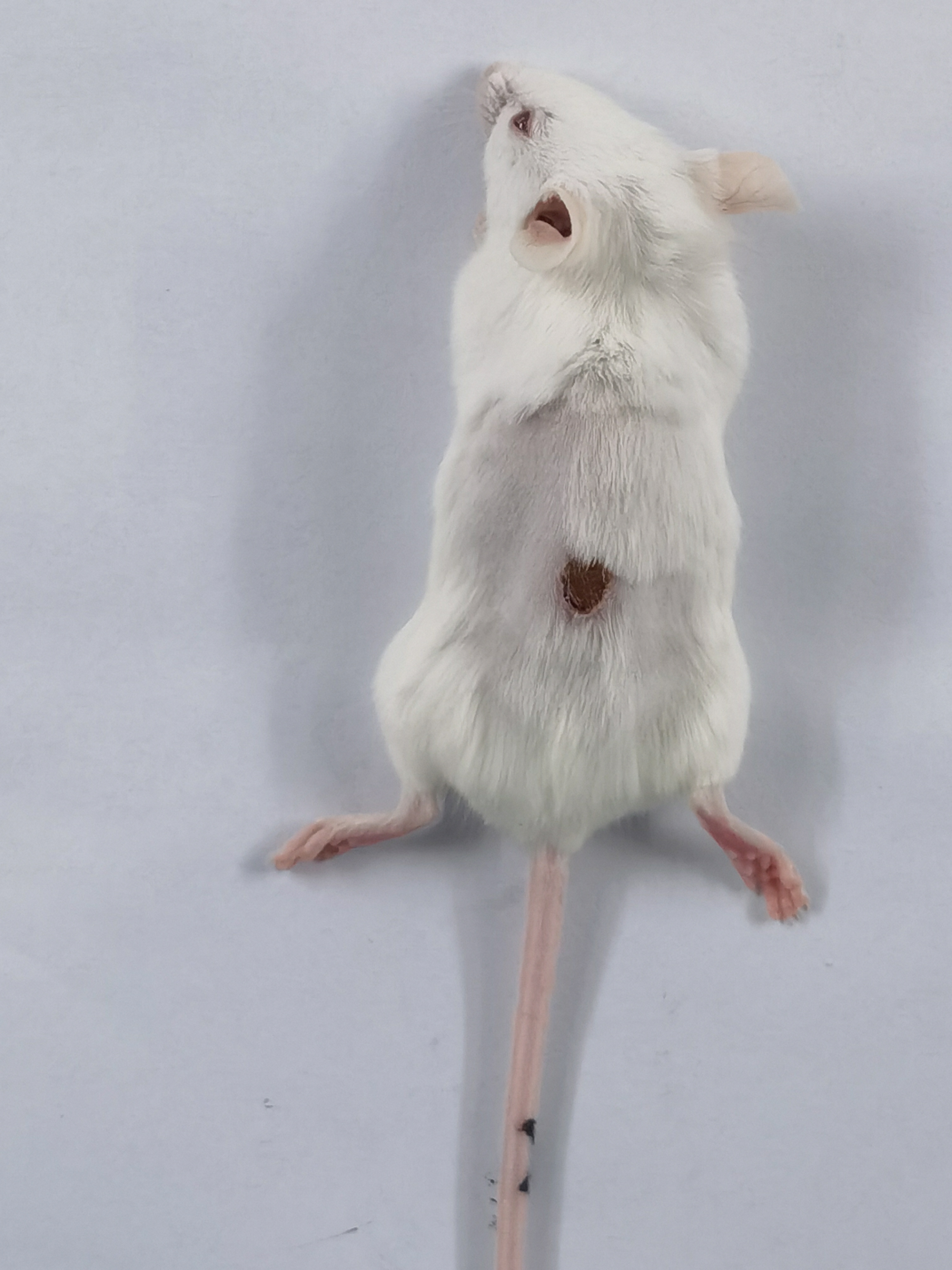

Supplement: Supplementary file 11 — Source data Fig. 6 [file 44321_2026_418_MOESM11_ESM.zip › Figure 6/Data-Figure 6B/Day 5/3-2.jpg]

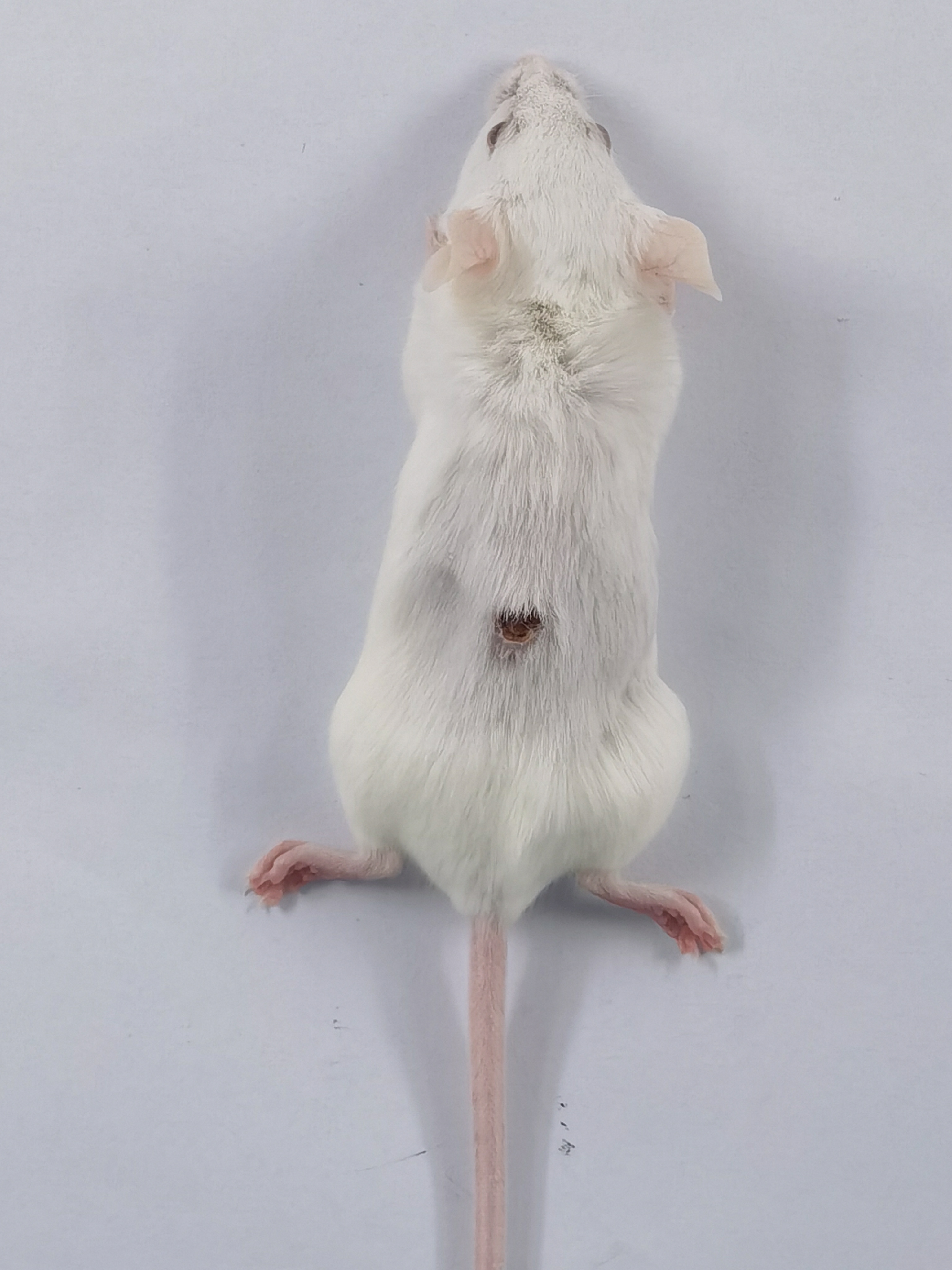

Supplement: Supplementary file 11 — Source data Fig. 6 [file 44321_2026_418_MOESM11_ESM.zip › Figure 6/Data-Figure 6B/Day 5/1-2.jpg]

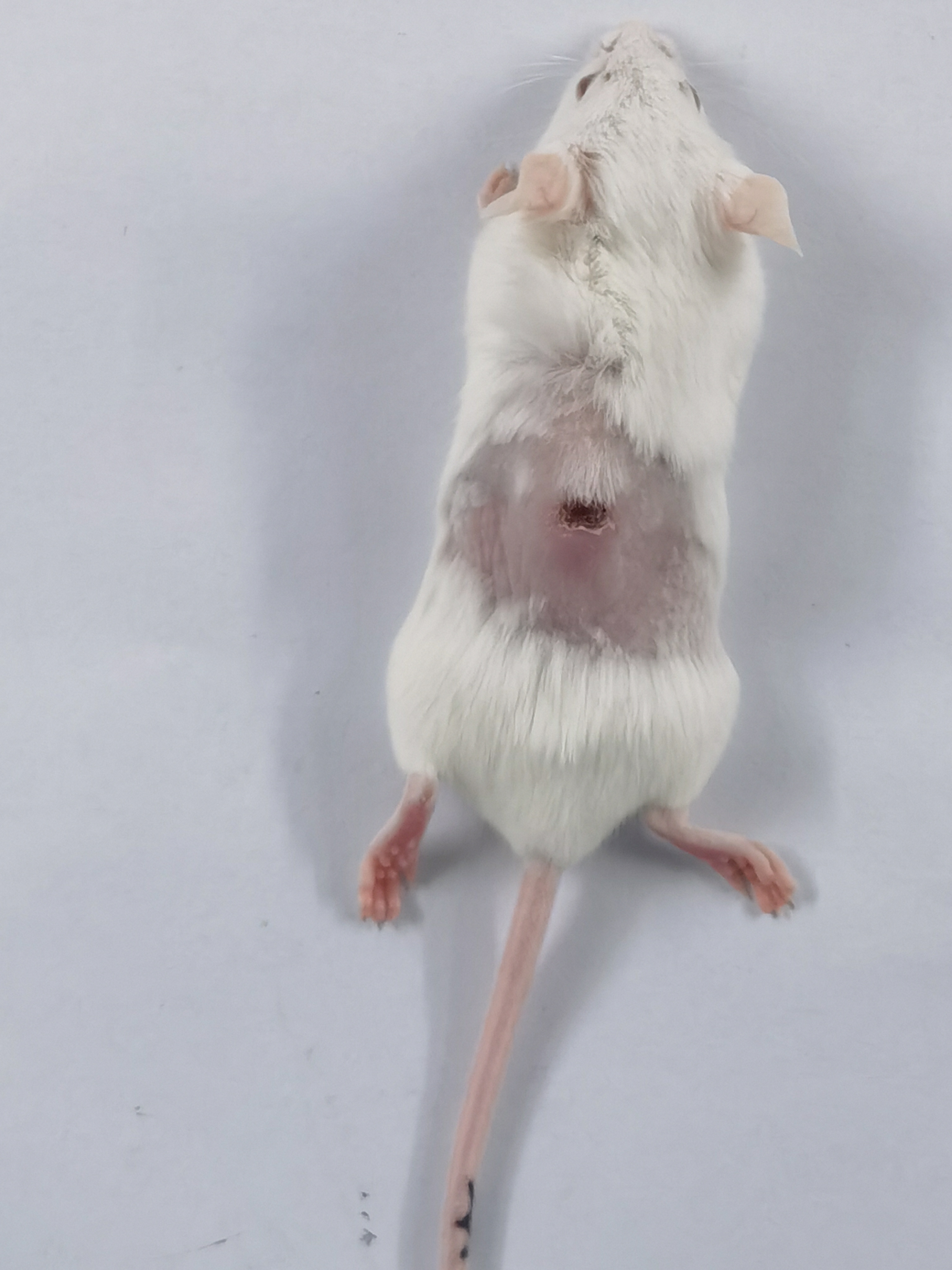

Supplement: Supplementary file 11 — Source data Fig. 6 [file 44321_2026_418_MOESM11_ESM.zip › Figure 6/Data-Figure 6B/Day 5/1-3.jpg]

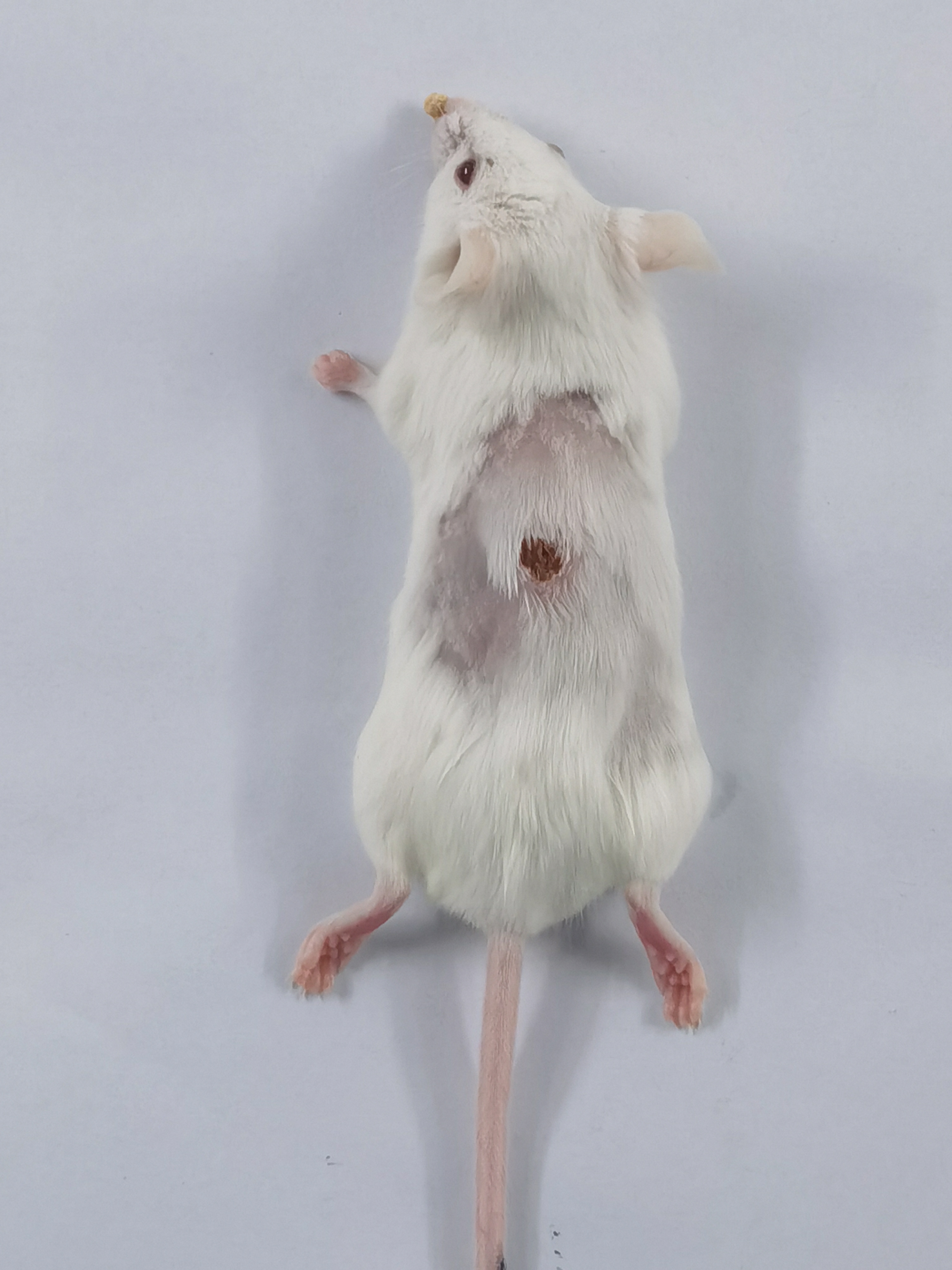

Supplement: Supplementary file 11 — Source data Fig. 6 [file 44321_2026_418_MOESM11_ESM.zip › Figure 6/Data-Figure 6B/Day 5/3-1.jpg]

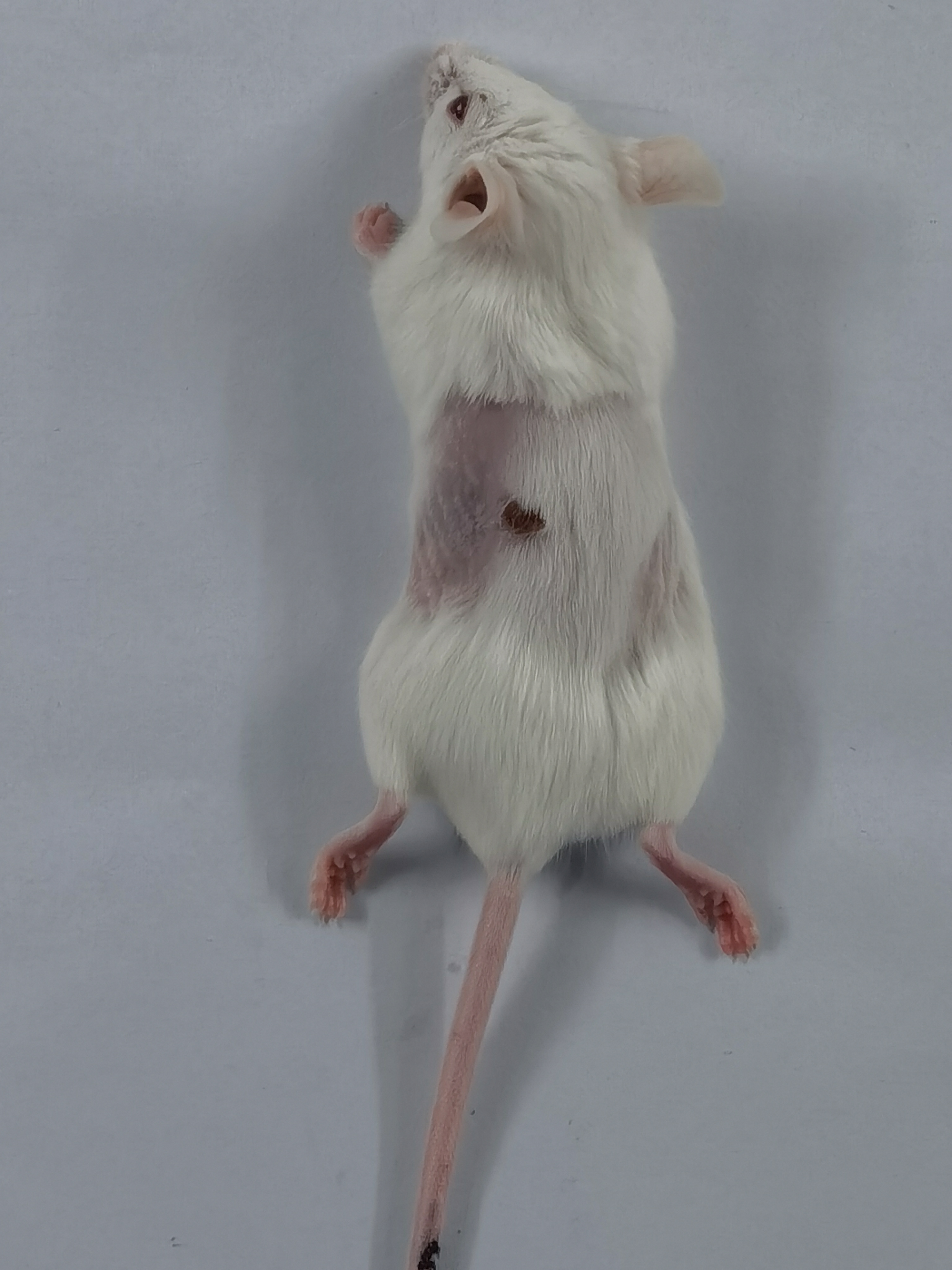

Supplement: Supplementary file 11 — Source data Fig. 6 [file 44321_2026_418_MOESM11_ESM.zip › Figure 6/Data-Figure 6B/Day 5/3-5.jpg]

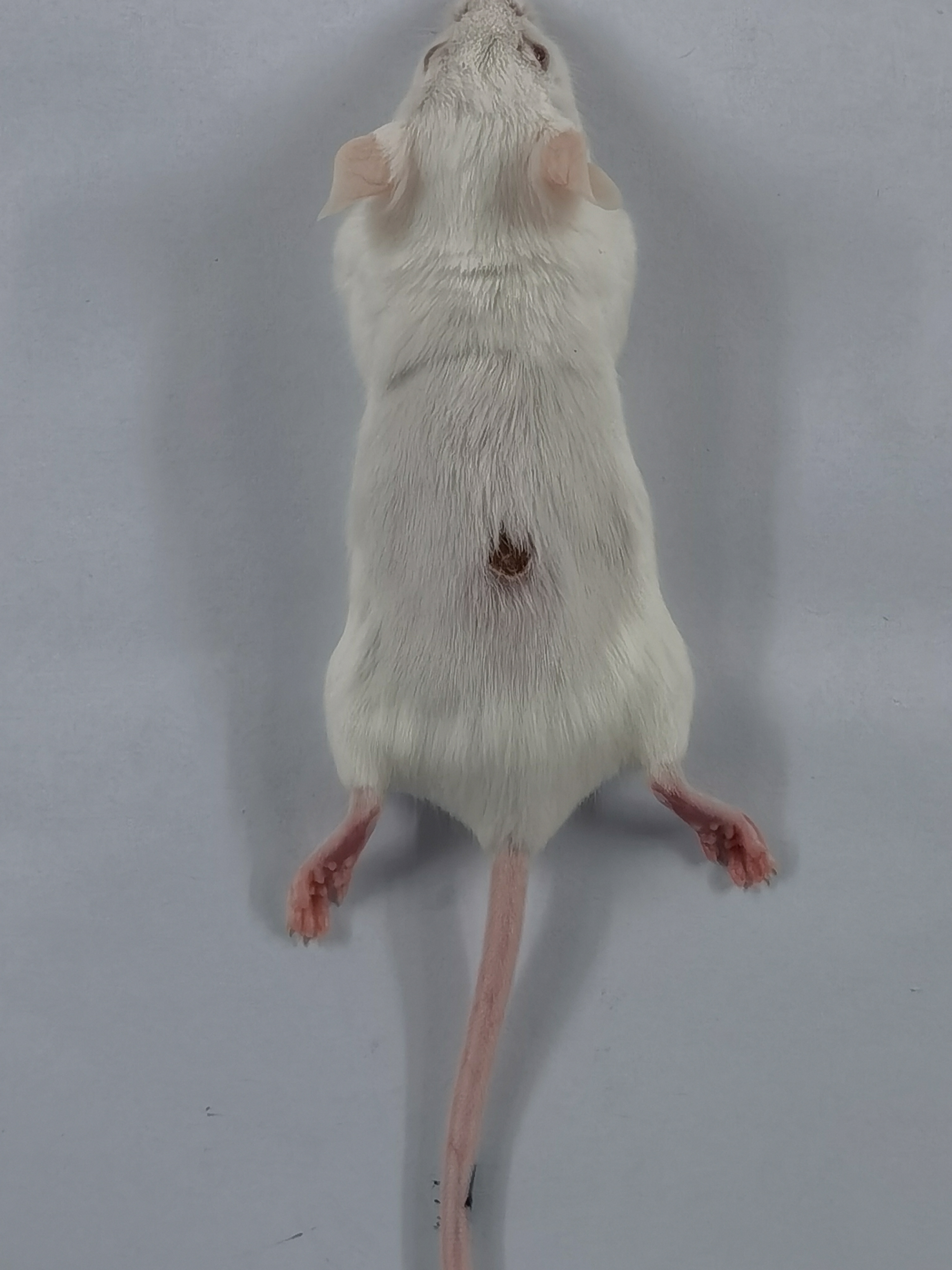

Supplement: Supplementary file 11 — Source data Fig. 6 [file 44321_2026_418_MOESM11_ESM.zip › Figure 6/Data-Figure 6B/Day 5/3-4.jpg]

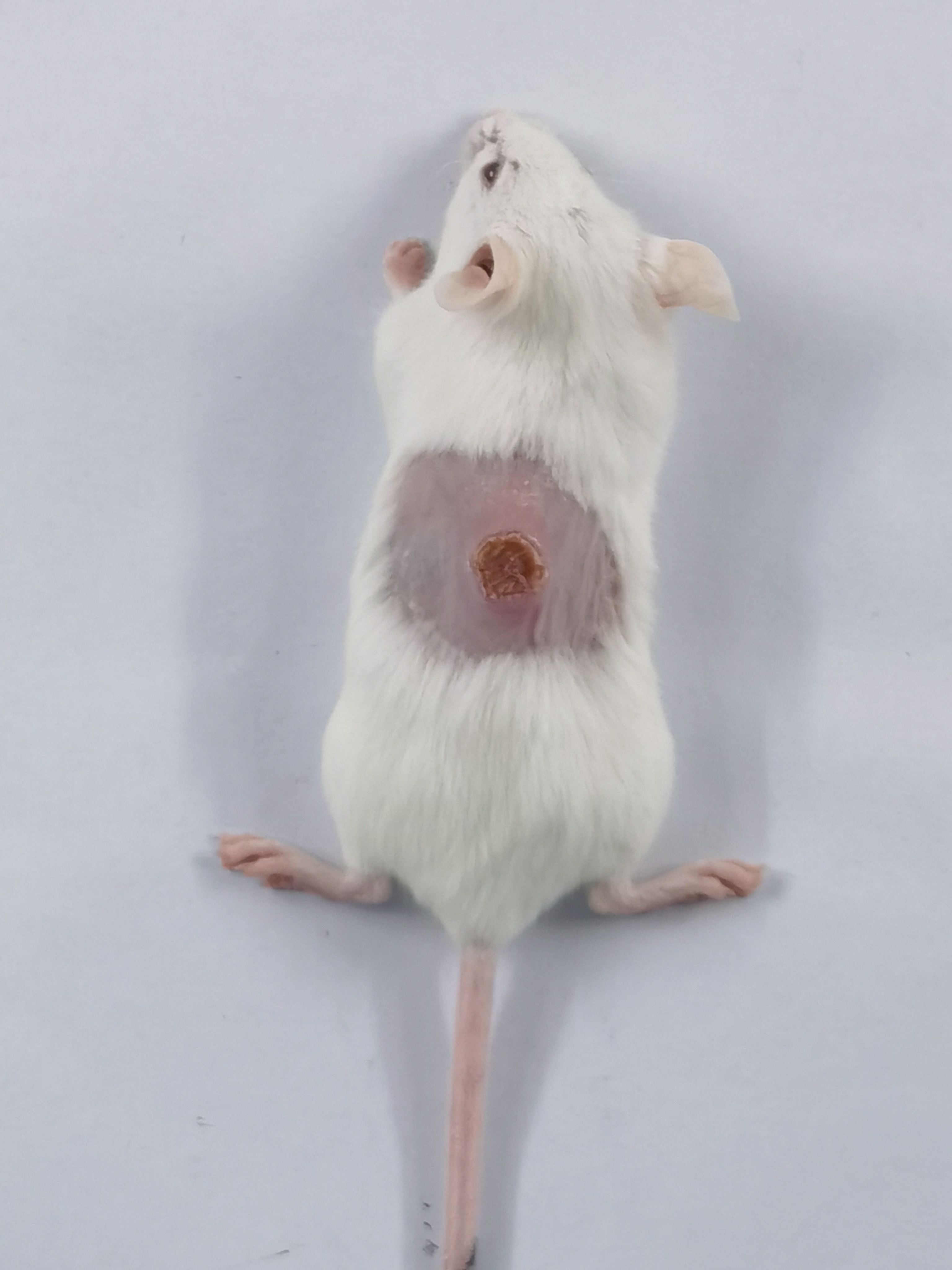

Supplement: Supplementary file 11 — Source data Fig. 6 [file 44321_2026_418_MOESM11_ESM.zip › Figure 6/Data-Figure 6B/Day 5/1-4.jpg]

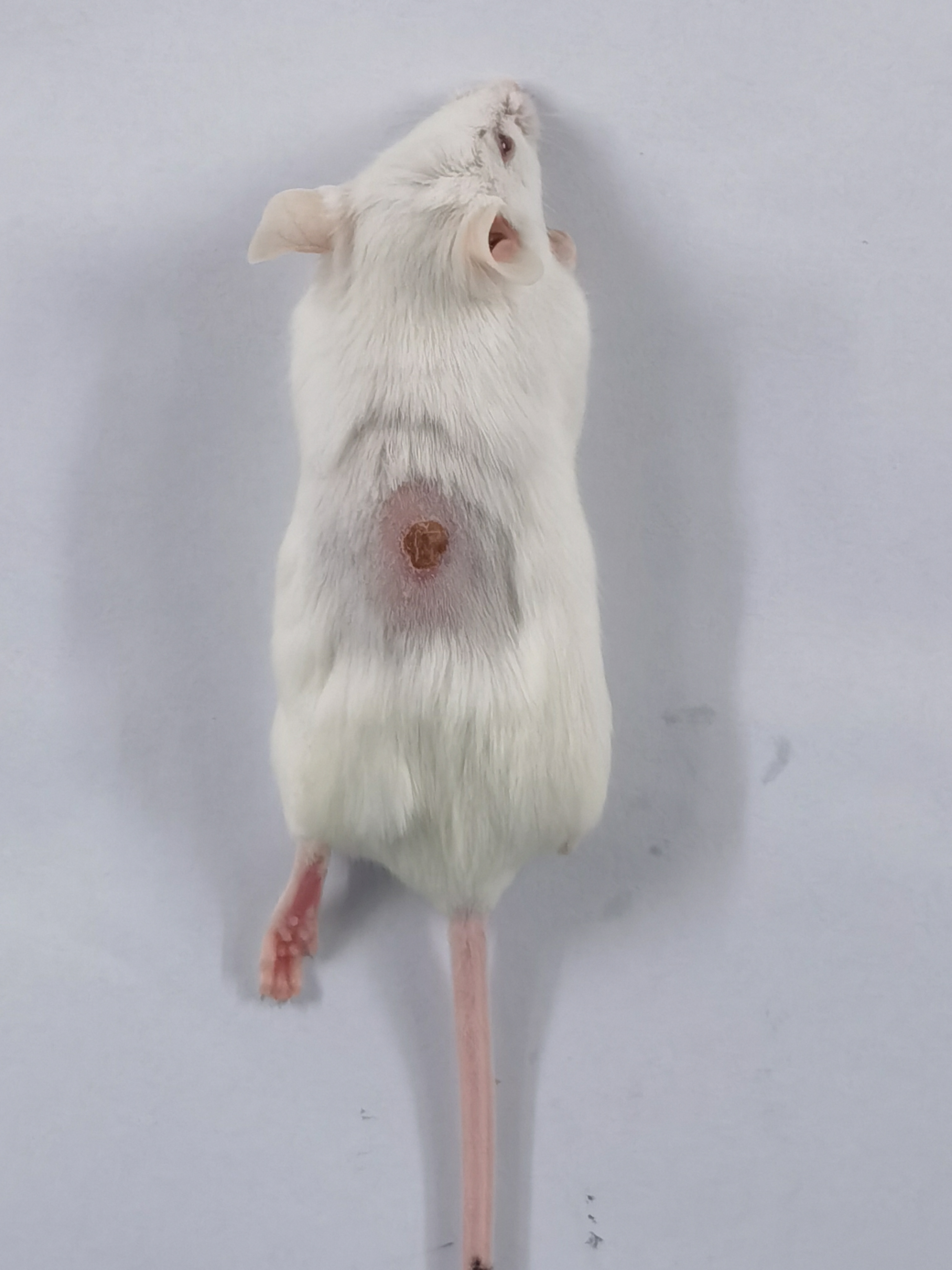

Supplement: Supplementary file 11 — Source data Fig. 6 [file 44321_2026_418_MOESM11_ESM.zip › Figure 6/Data-Figure 6B/Day 5/1-5.jpg]

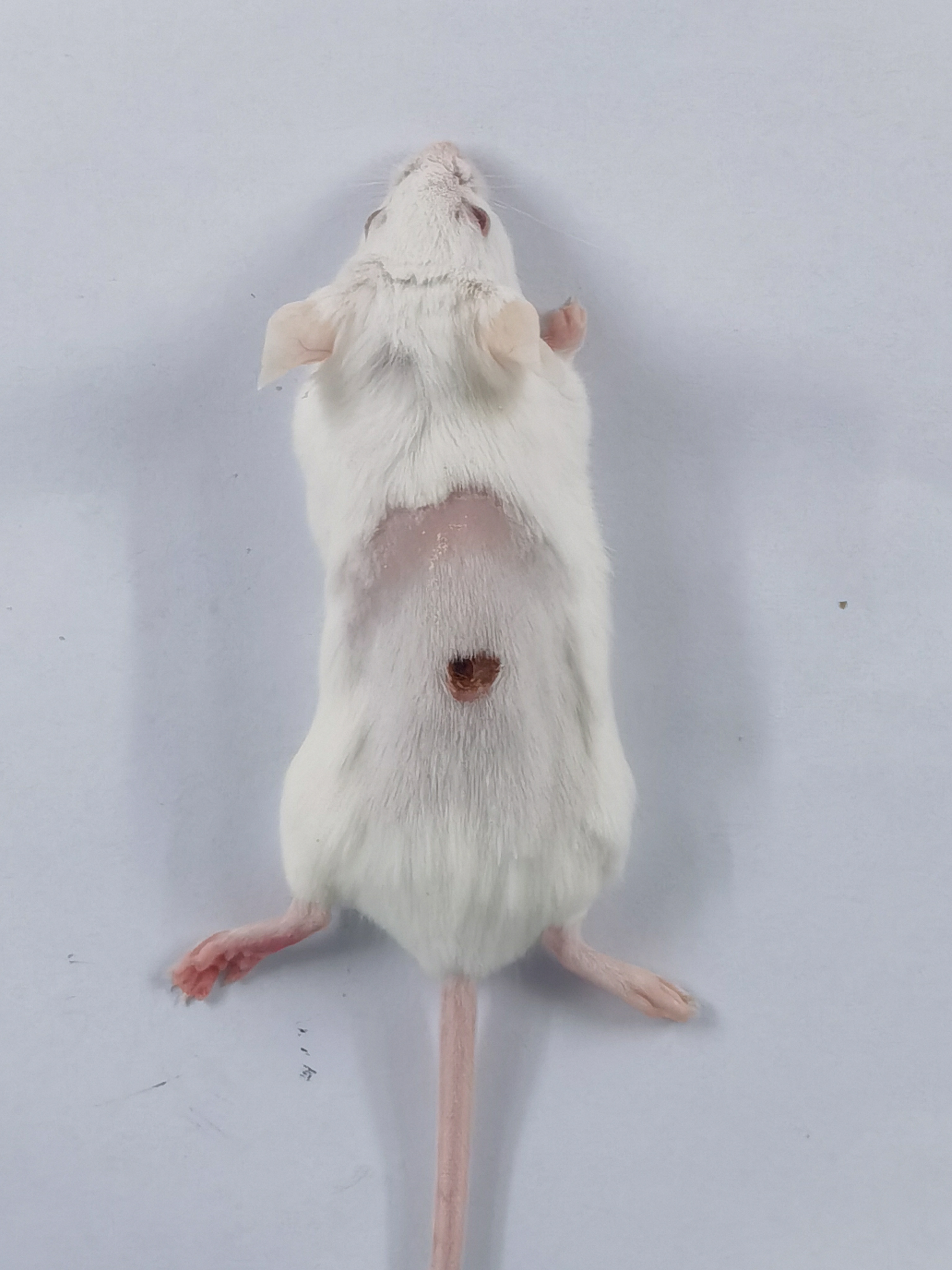

Supplement: Supplementary file 11 — Source data Fig. 6 [file 44321_2026_418_MOESM11_ESM.zip › Figure 6/Data-Figure 6B/Day 4/2-1.jpg]

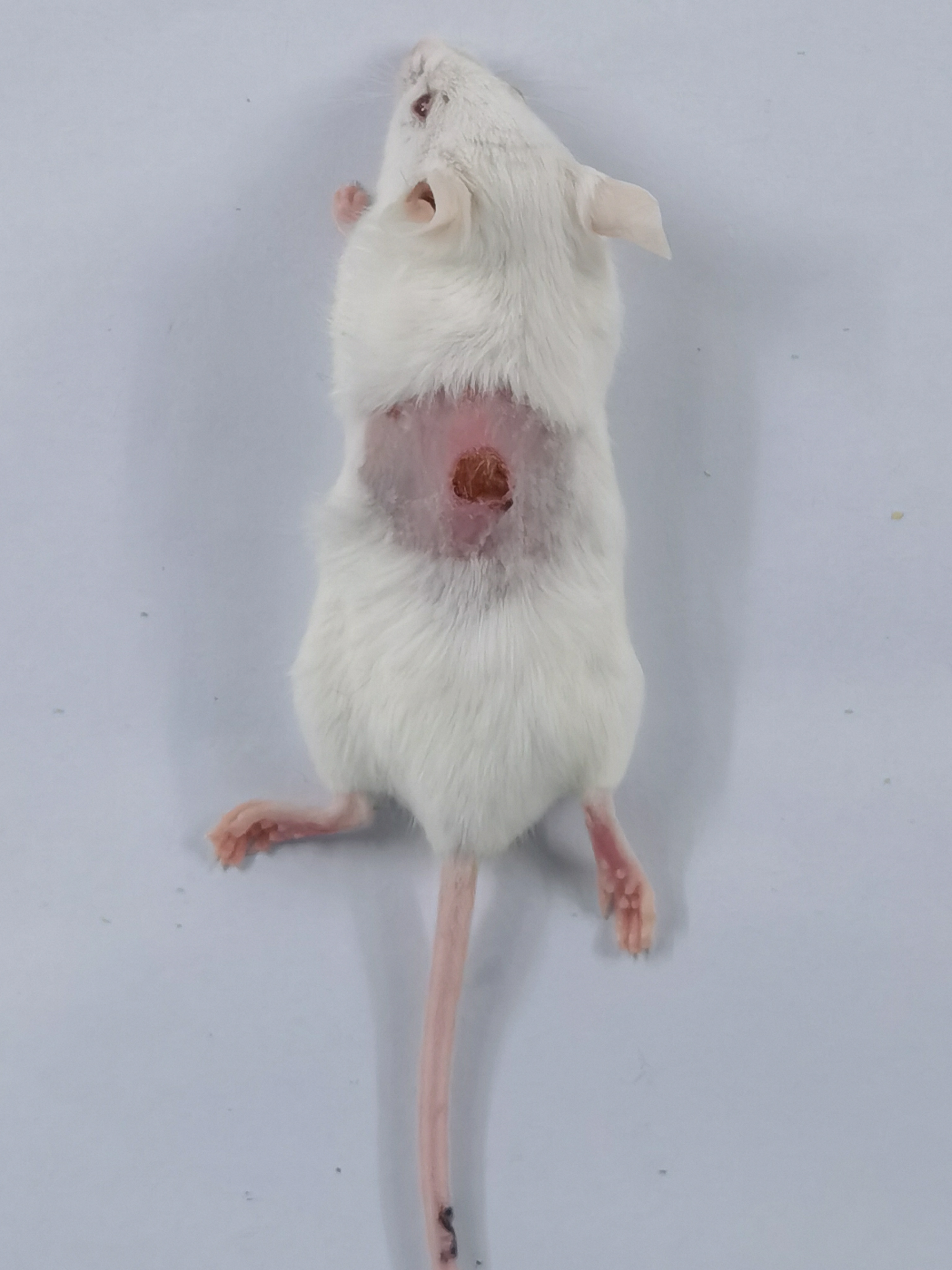

Supplement: Supplementary file 11 — Source data Fig. 6 [file 44321_2026_418_MOESM11_ESM.zip › Figure 6/Data-Figure 6B/Day 4/4-5.jpg]

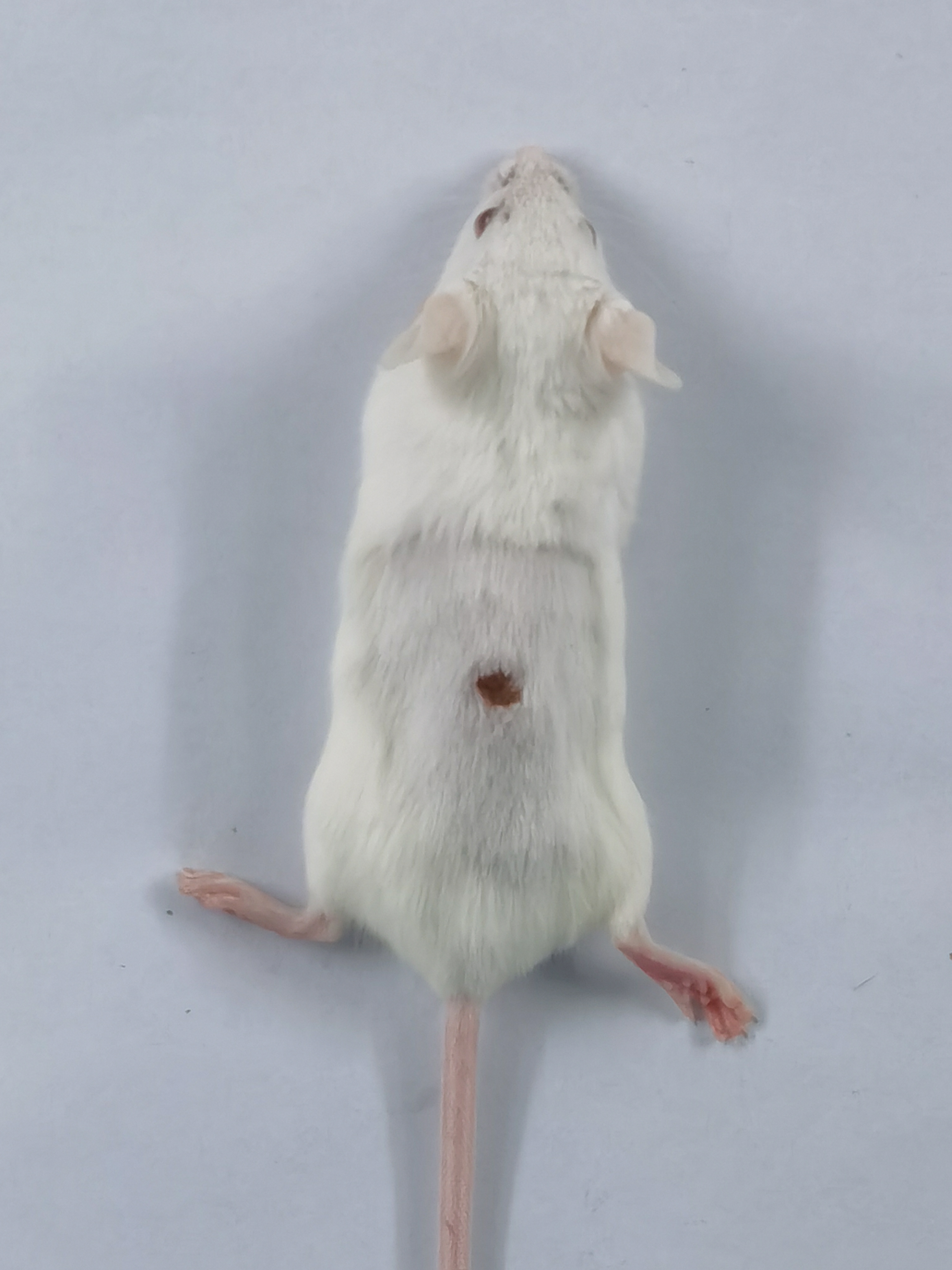

Supplement: Supplementary file 11 — Source data Fig. 6 [file 44321_2026_418_MOESM11_ESM.zip › Figure 6/Data-Figure 6B/Day 4/2-3.jpg]

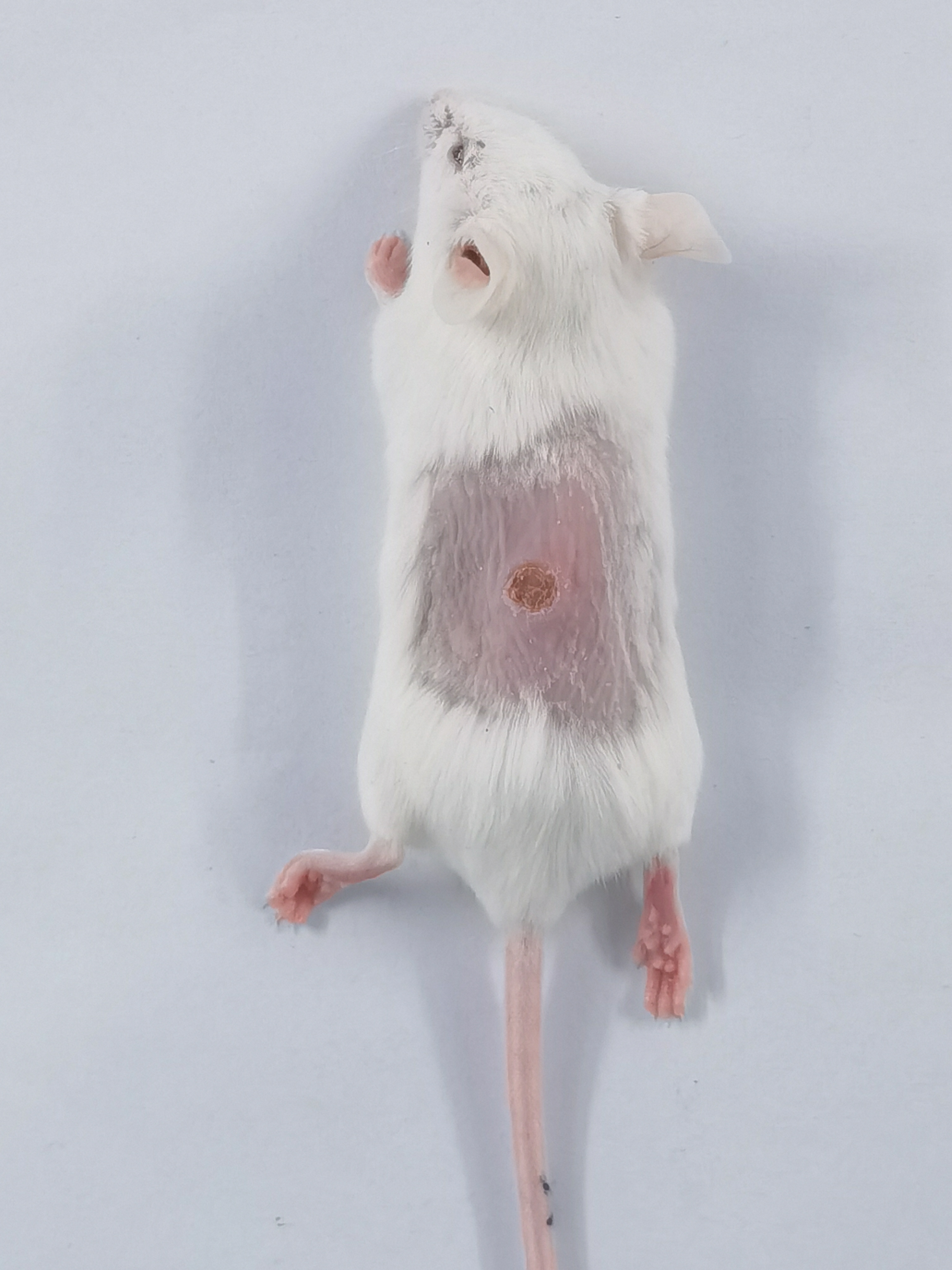

Supplement: Supplementary file 11 — Source data Fig. 6 [file 44321_2026_418_MOESM11_ESM.zip › Figure 6/Data-Figure 6B/Day 4/2-2.jpg]

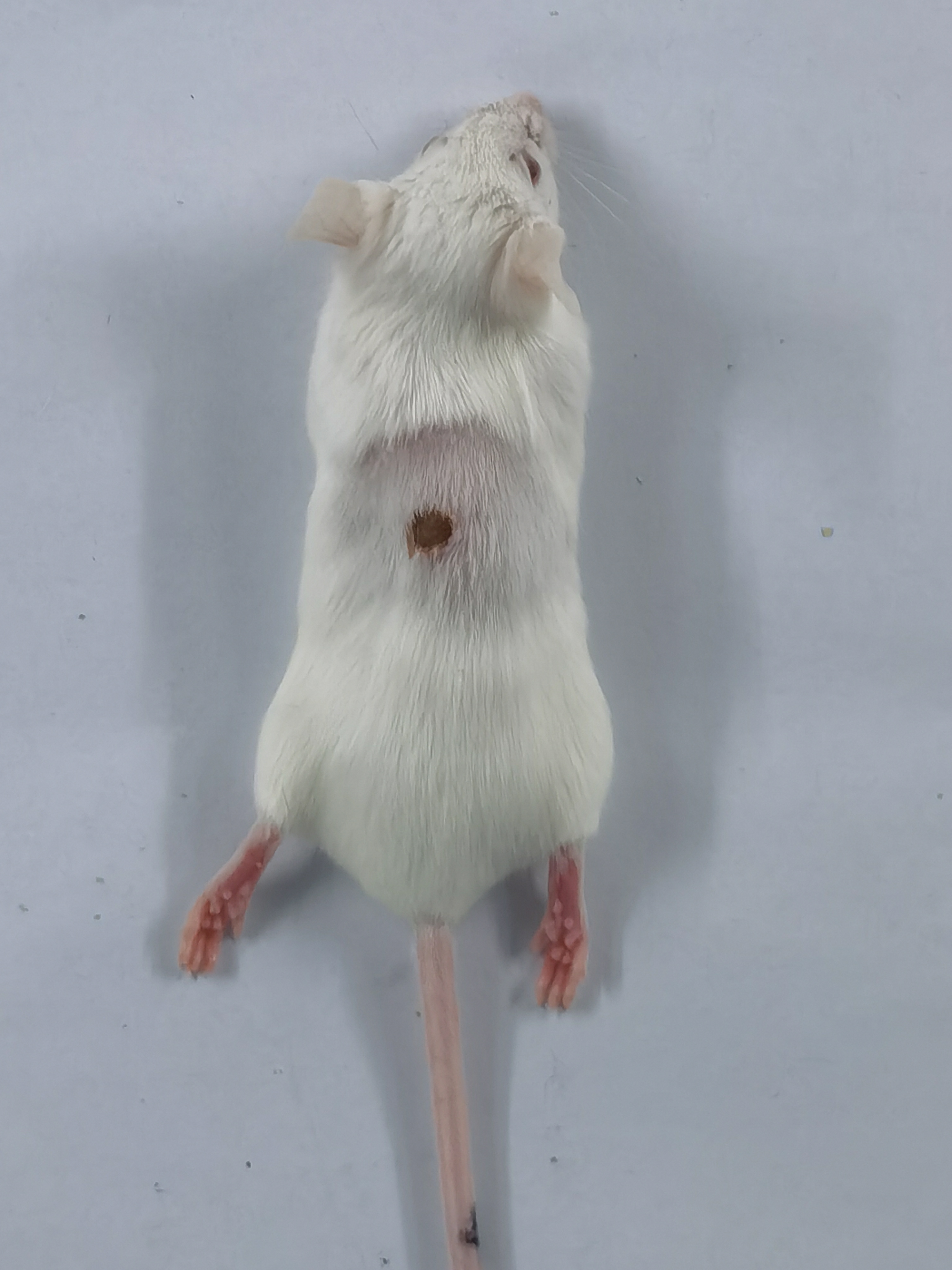

Supplement: Supplementary file 11 — Source data Fig. 6 [file 44321_2026_418_MOESM11_ESM.zip › Figure 6/Data-Figure 6B/Day 4/4-4.jpg]

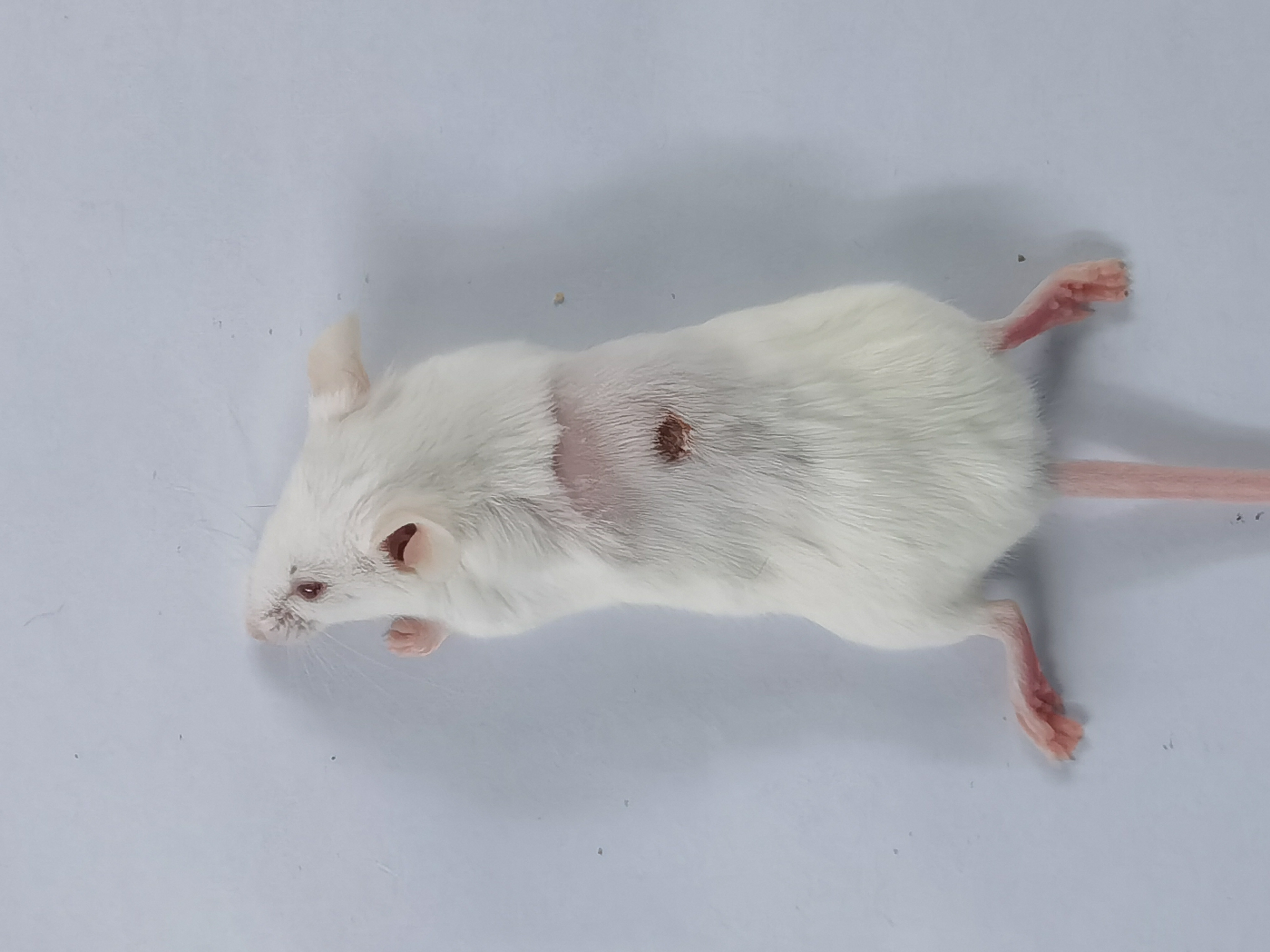

Supplement: Supplementary file 11 — Source data Fig. 6 [file 44321_2026_418_MOESM11_ESM.zip › Figure 6/Data-Figure 6B/Day 4/4-1.jpg]

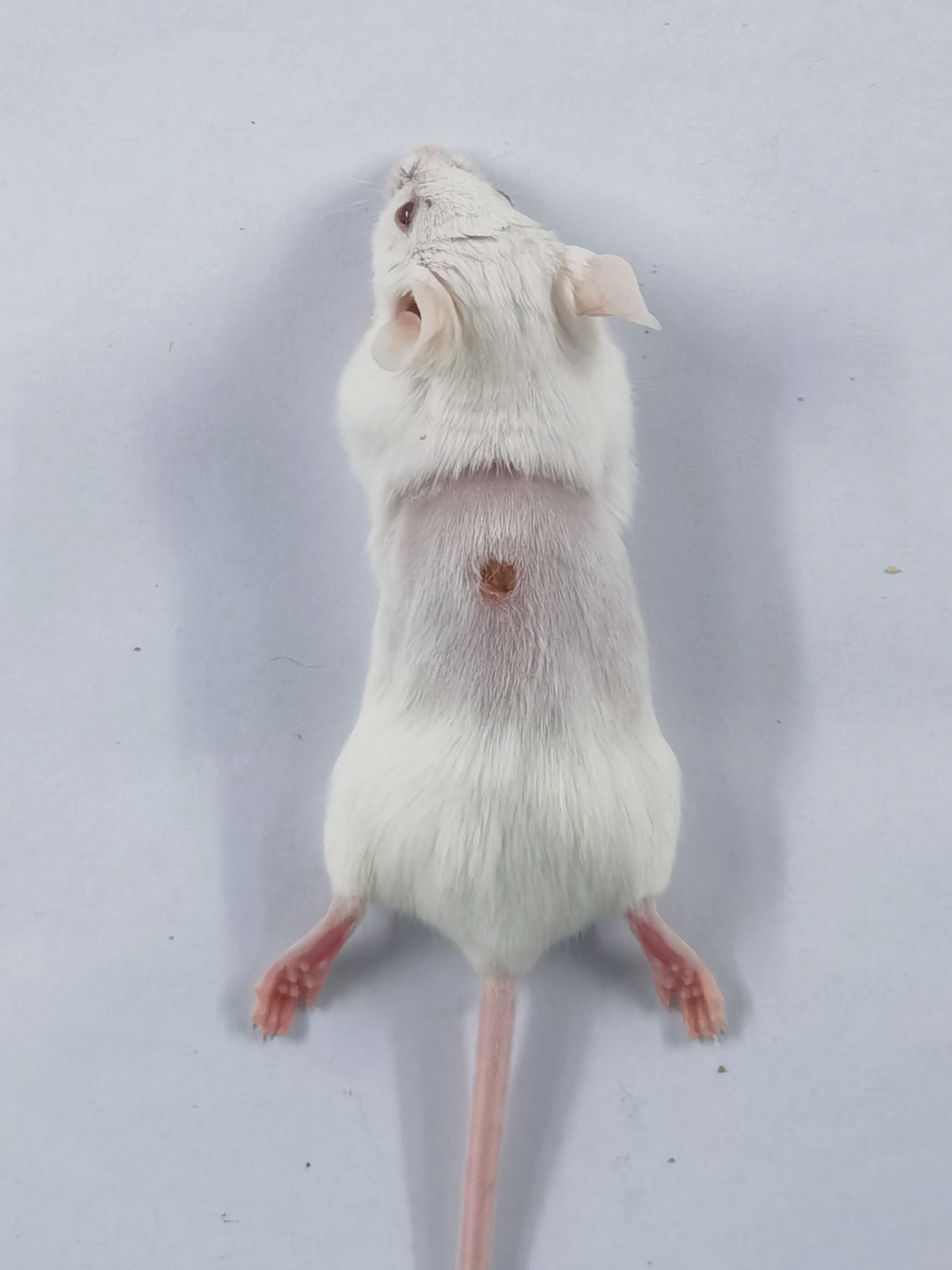

Supplement: Supplementary file 11 — Source data Fig. 6 [file 44321_2026_418_MOESM11_ESM.zip › Figure 6/Data-Figure 6B/Day 4/4-3.jpg]

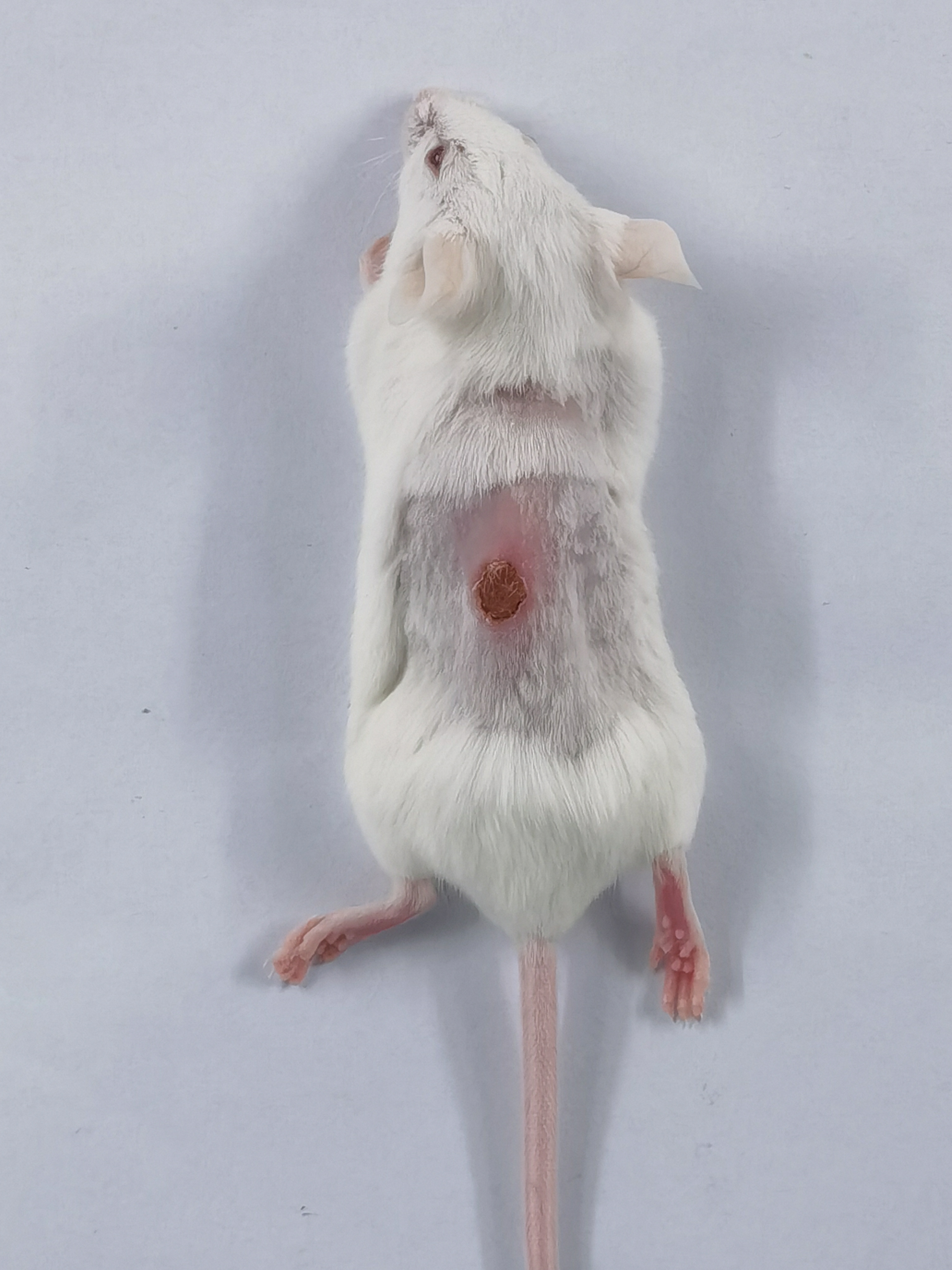

Supplement: Supplementary file 11 — Source data Fig. 6 [file 44321_2026_418_MOESM11_ESM.zip › Figure 6/Data-Figure 6B/Day 4/2-5.jpg]

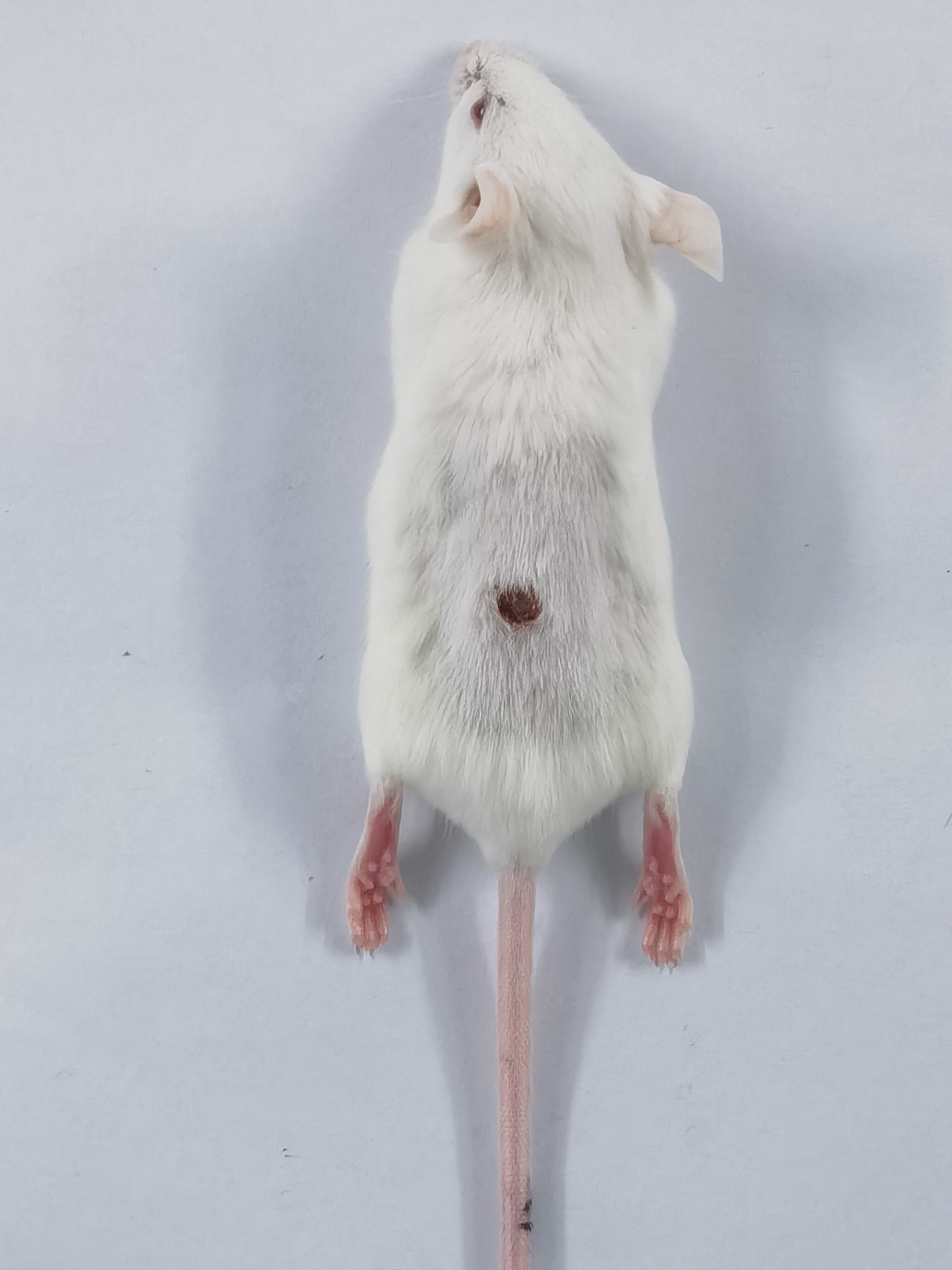

Supplement: Supplementary file 11 — Source data Fig. 6 [file 44321_2026_418_MOESM11_ESM.zip › Figure 6/Data-Figure 6B/Day 4/2-4.jpg]

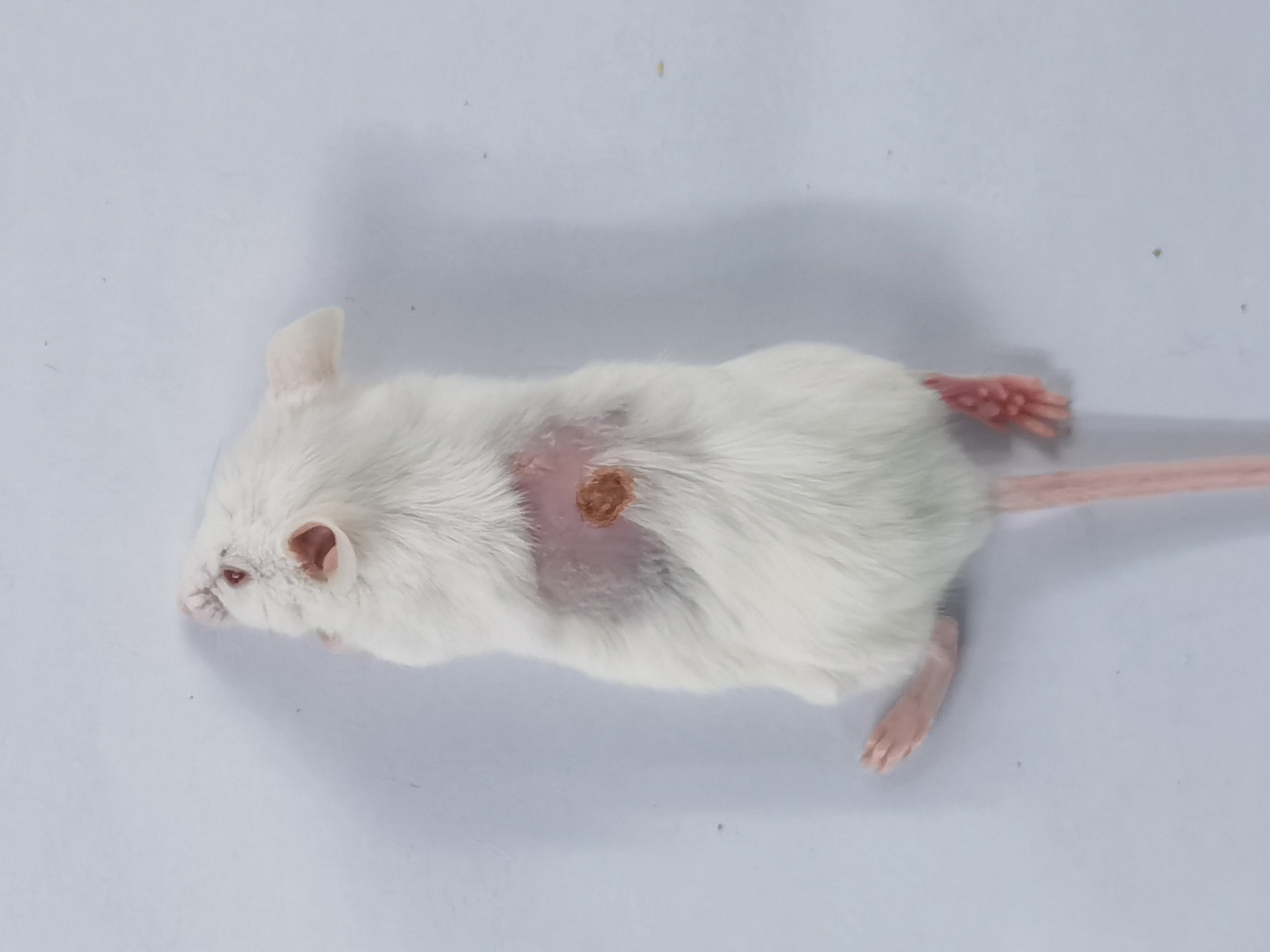

Supplement: Supplementary file 11 — Source data Fig. 6 [file 44321_2026_418_MOESM11_ESM.zip › Figure 6/Data-Figure 6B/Day 4/4-2.jpg]

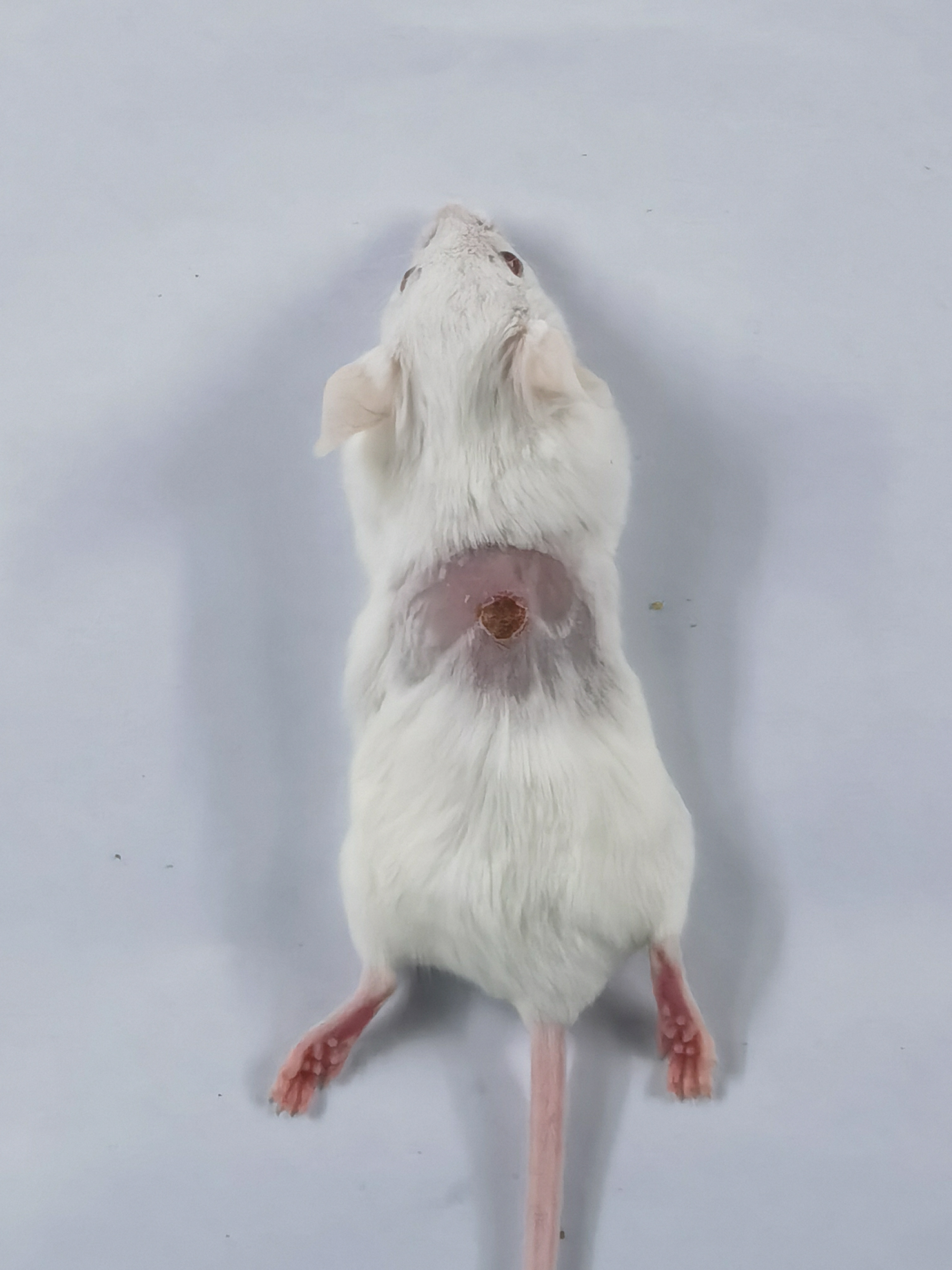

Supplement: Supplementary file 11 — Source data Fig. 6 [file 44321_2026_418_MOESM11_ESM.zip › Figure 6/Data-Figure 6B/Day 4/3-3.jpg]

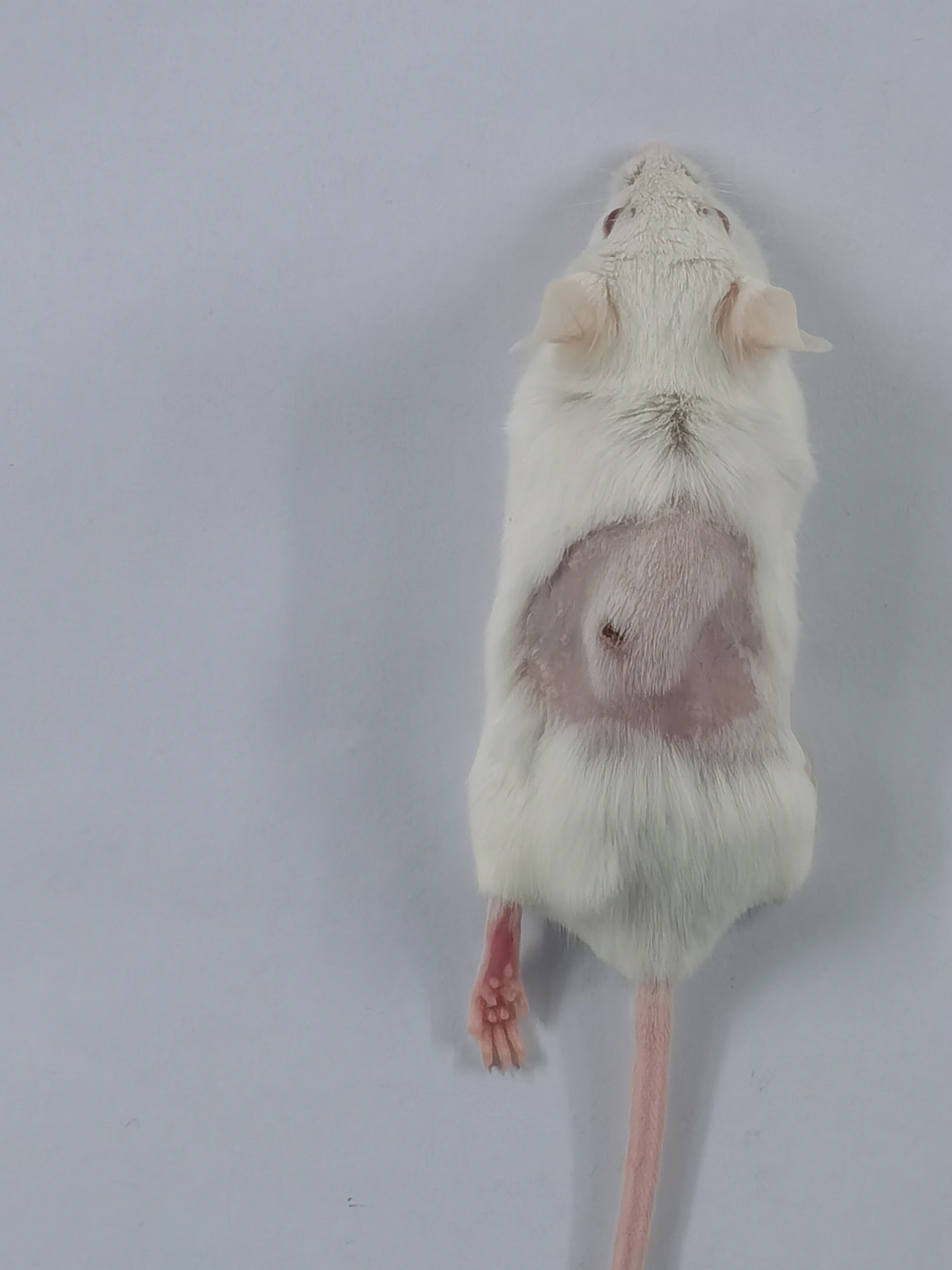

Supplement: Supplementary file 11 — Source data Fig. 6 [file 44321_2026_418_MOESM11_ESM.zip › Figure 6/Data-Figure 6B/Day 4/1-1.jpg]

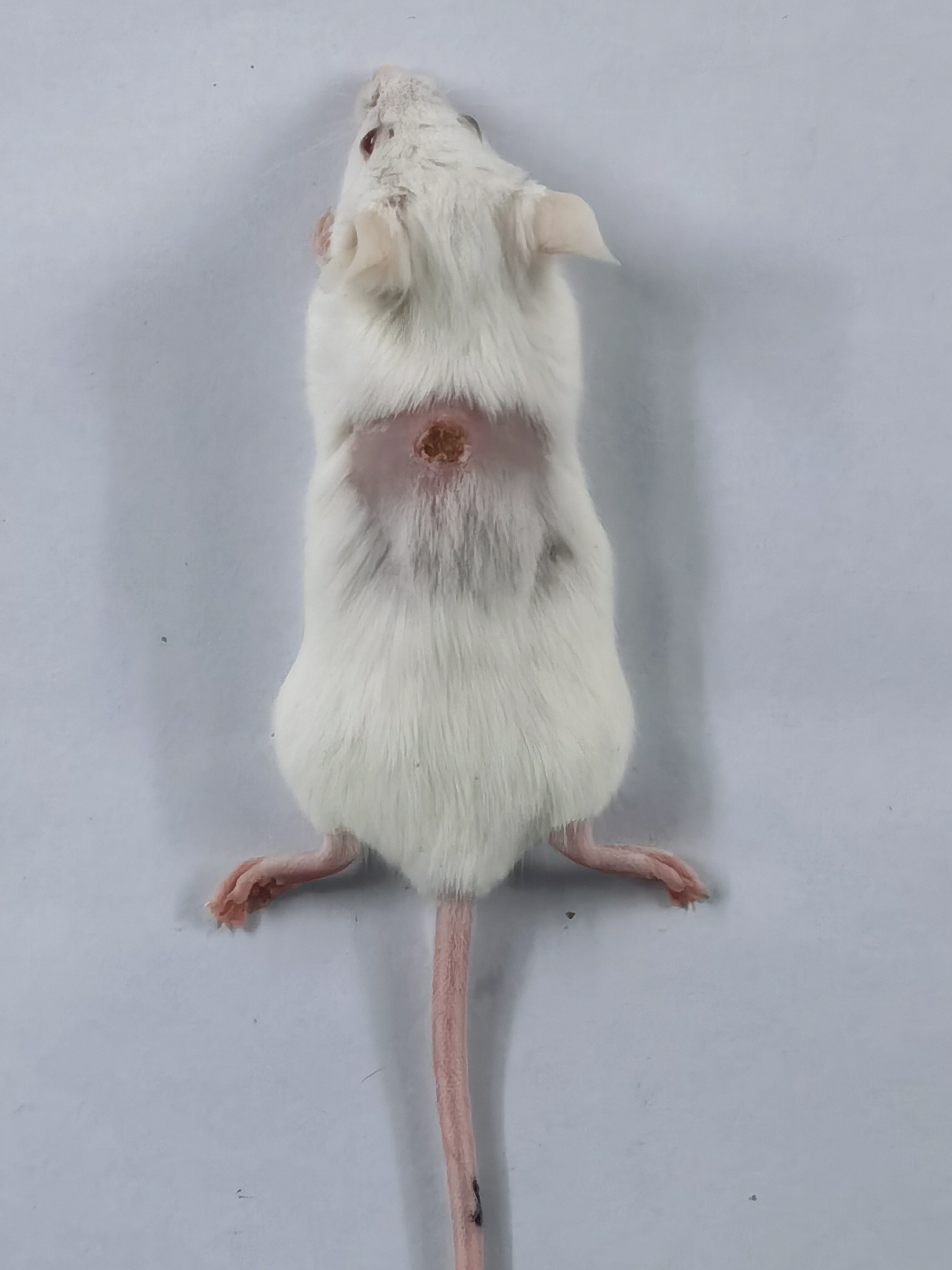

Supplement: Supplementary file 11 — Source data Fig. 6 [file 44321_2026_418_MOESM11_ESM.zip › Figure 6/Data-Figure 6B/Day 4/3-2.jpg]

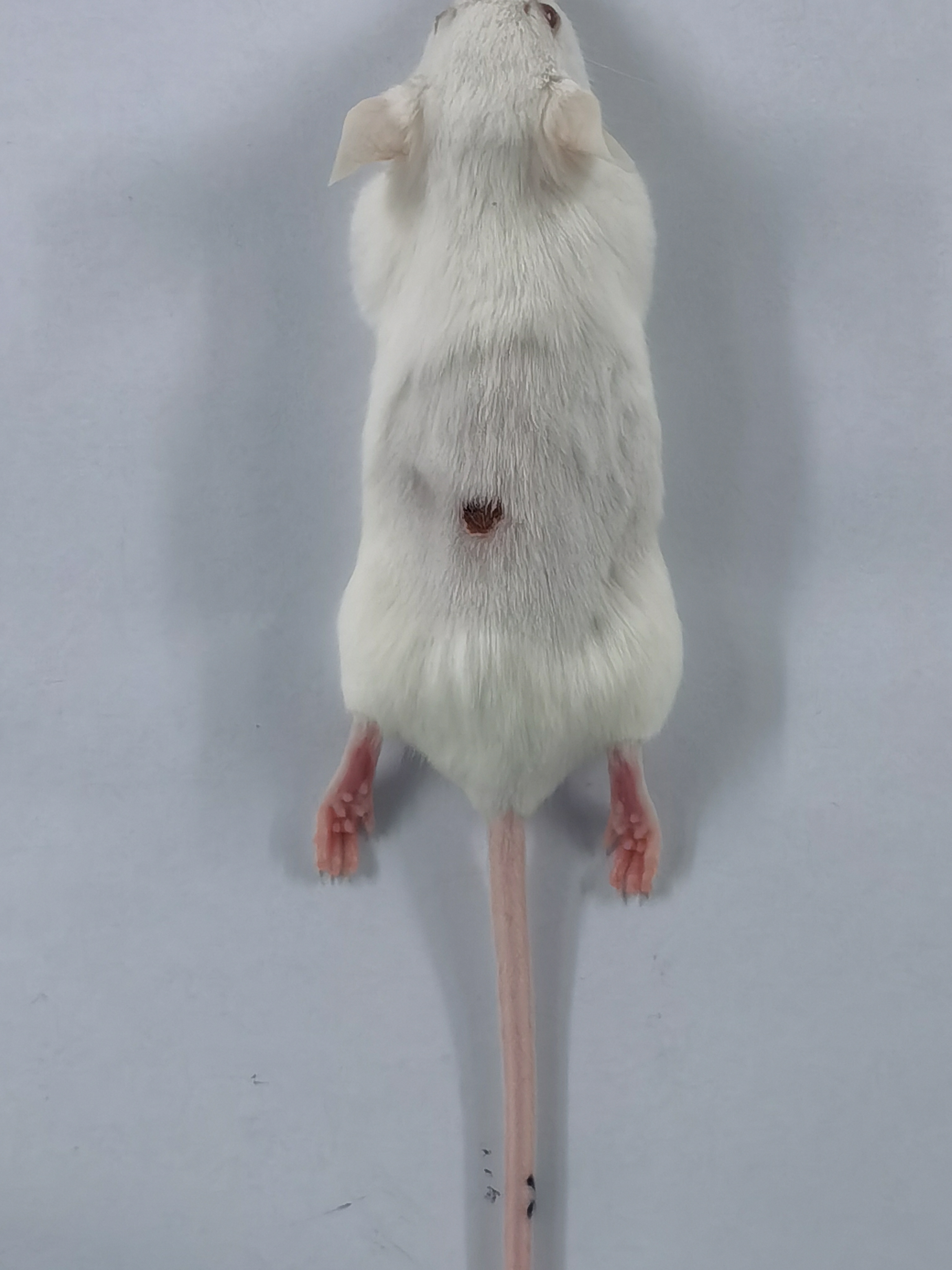

Supplement: Supplementary file 11 — Source data Fig. 6 [file 44321_2026_418_MOESM11_ESM.zip › Figure 6/Data-Figure 6B/Day 4/1-2.jpg]

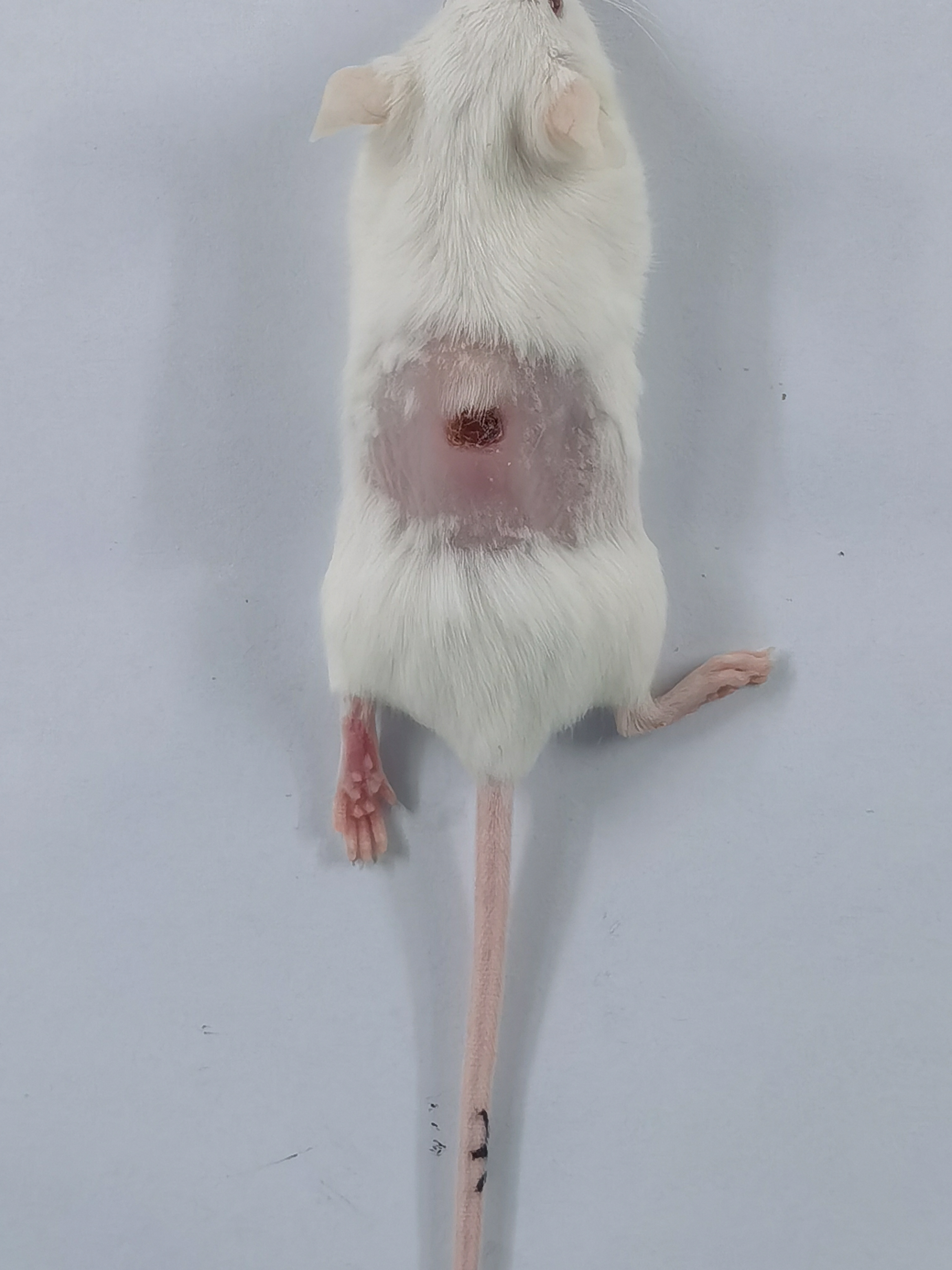

Supplement: Supplementary file 11 — Source data Fig. 6 [file 44321_2026_418_MOESM11_ESM.zip › Figure 6/Data-Figure 6B/Day 4/1-3.jpg]

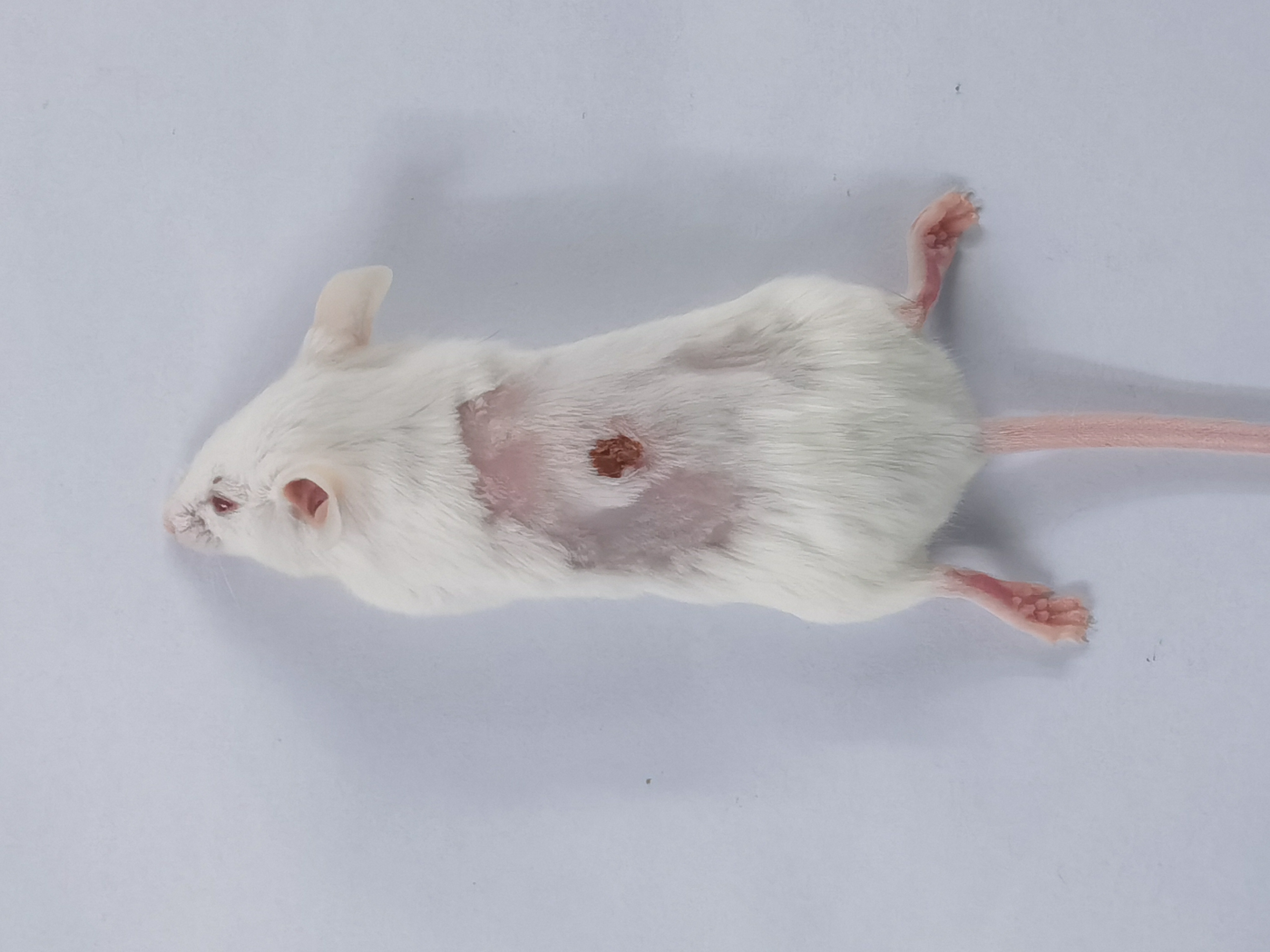

Supplement: Supplementary file 11 — Source data Fig. 6 [file 44321_2026_418_MOESM11_ESM.zip › Figure 6/Data-Figure 6B/Day 4/3-1.jpg]

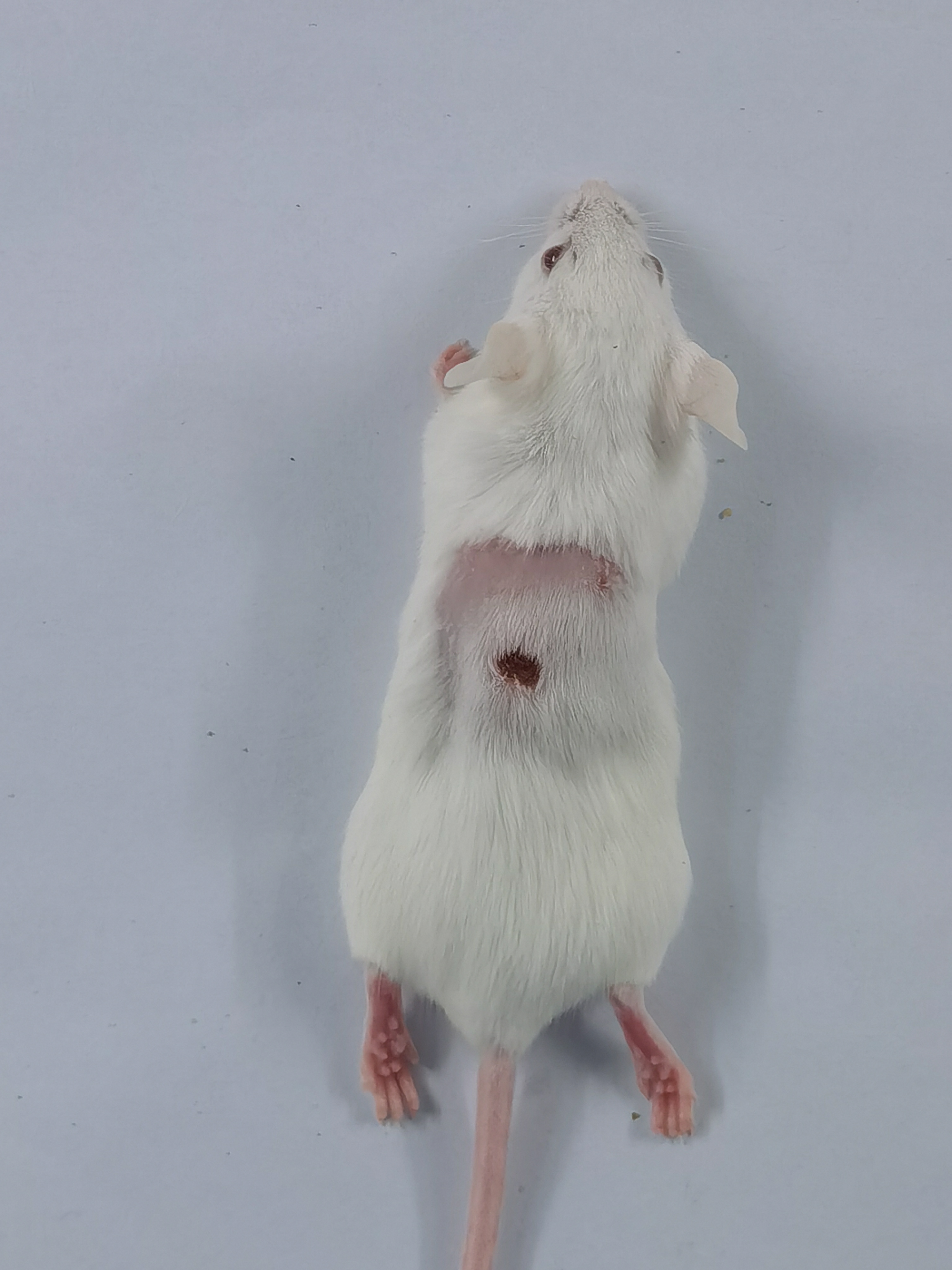

Supplement: Supplementary file 11 — Source data Fig. 6 [file 44321_2026_418_MOESM11_ESM.zip › Figure 6/Data-Figure 6B/Day 4/3-4.jpg]

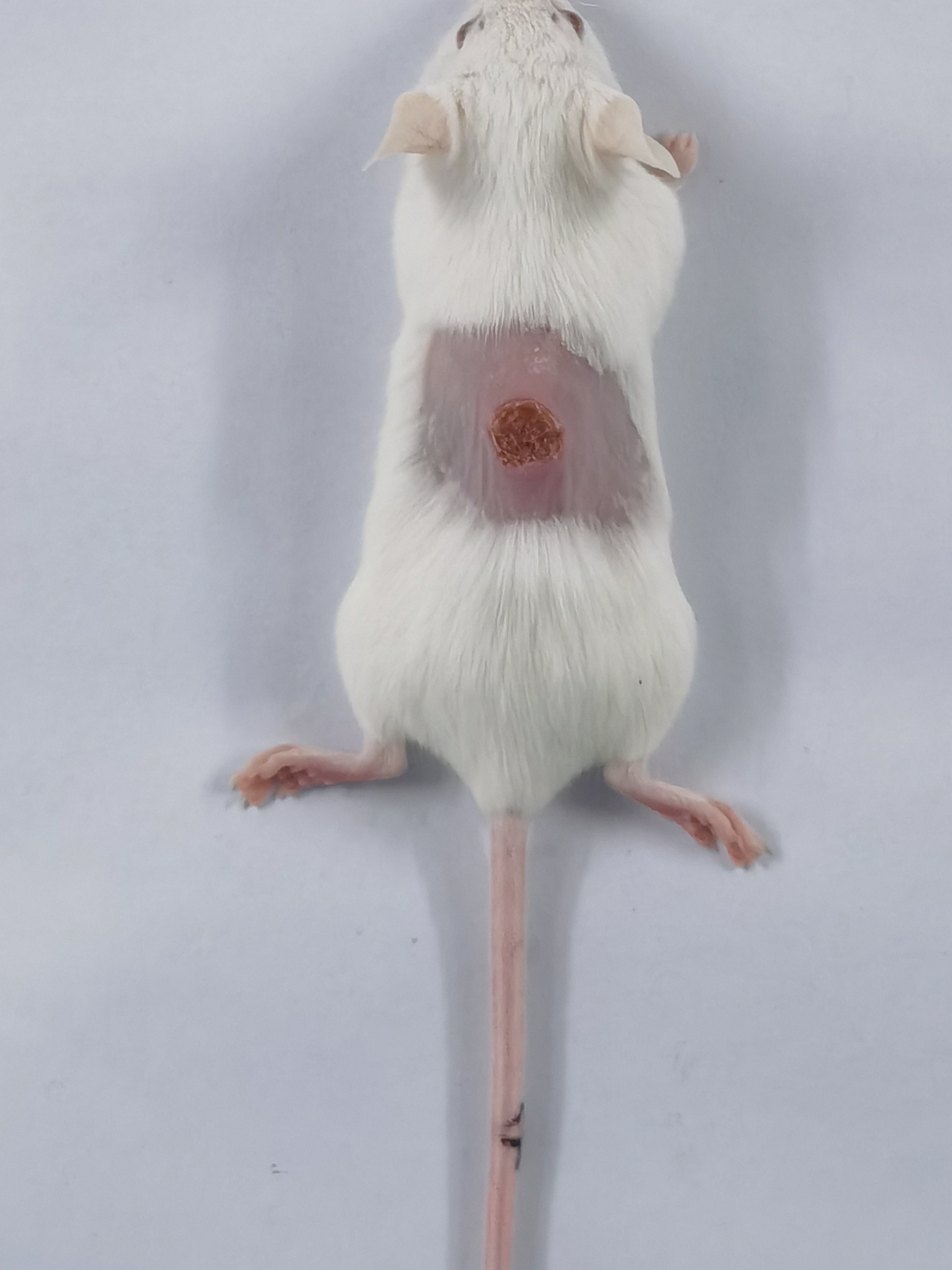

Supplement: Supplementary file 11 — Source data Fig. 6 [file 44321_2026_418_MOESM11_ESM.zip › Figure 6/Data-Figure 6B/Day 4/1-4.jpg]

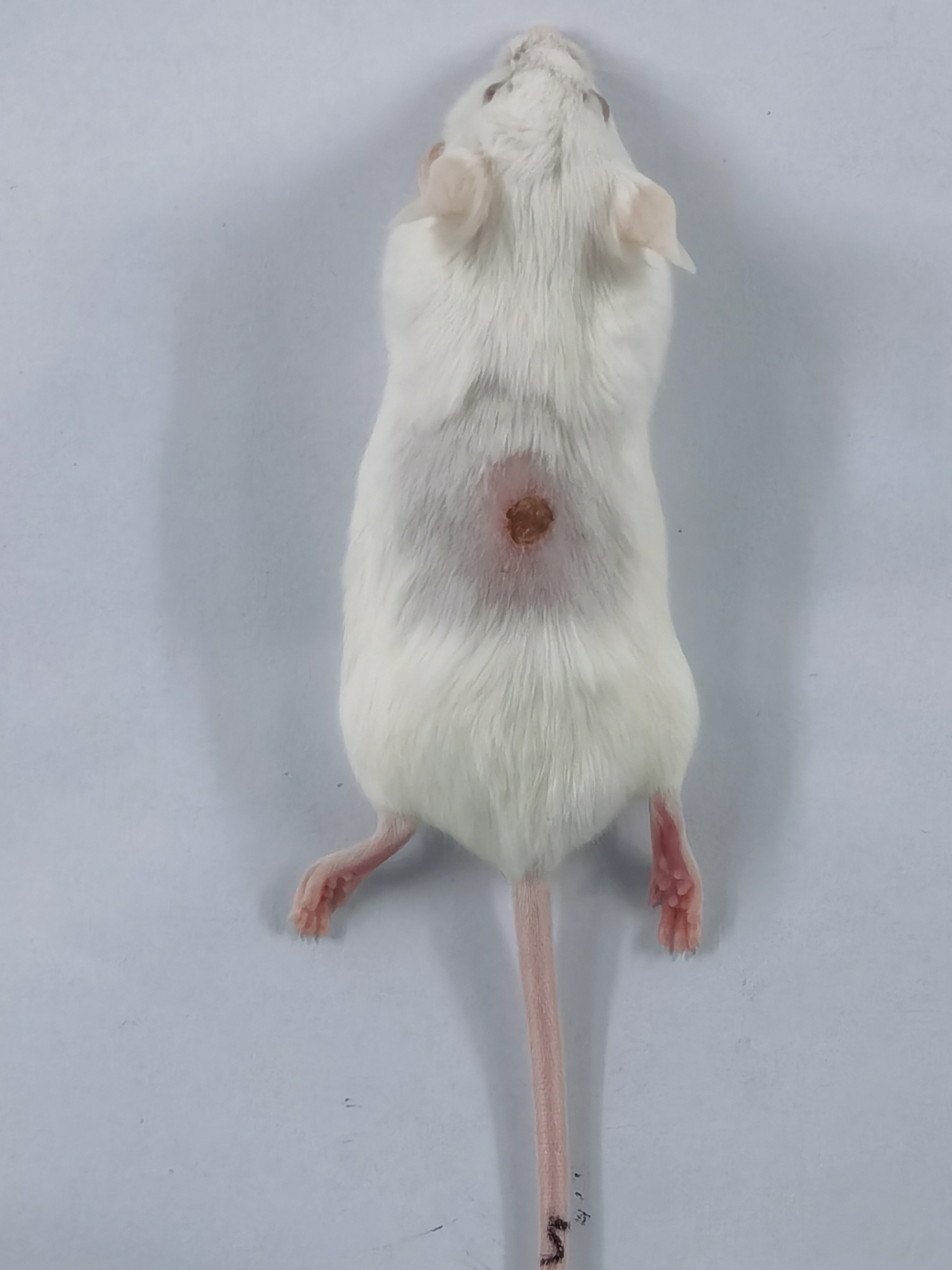

Supplement: Supplementary file 11 — Source data Fig. 6 [file 44321_2026_418_MOESM11_ESM.zip › Figure 6/Data-Figure 6B/Day 4/1-5.jpg]

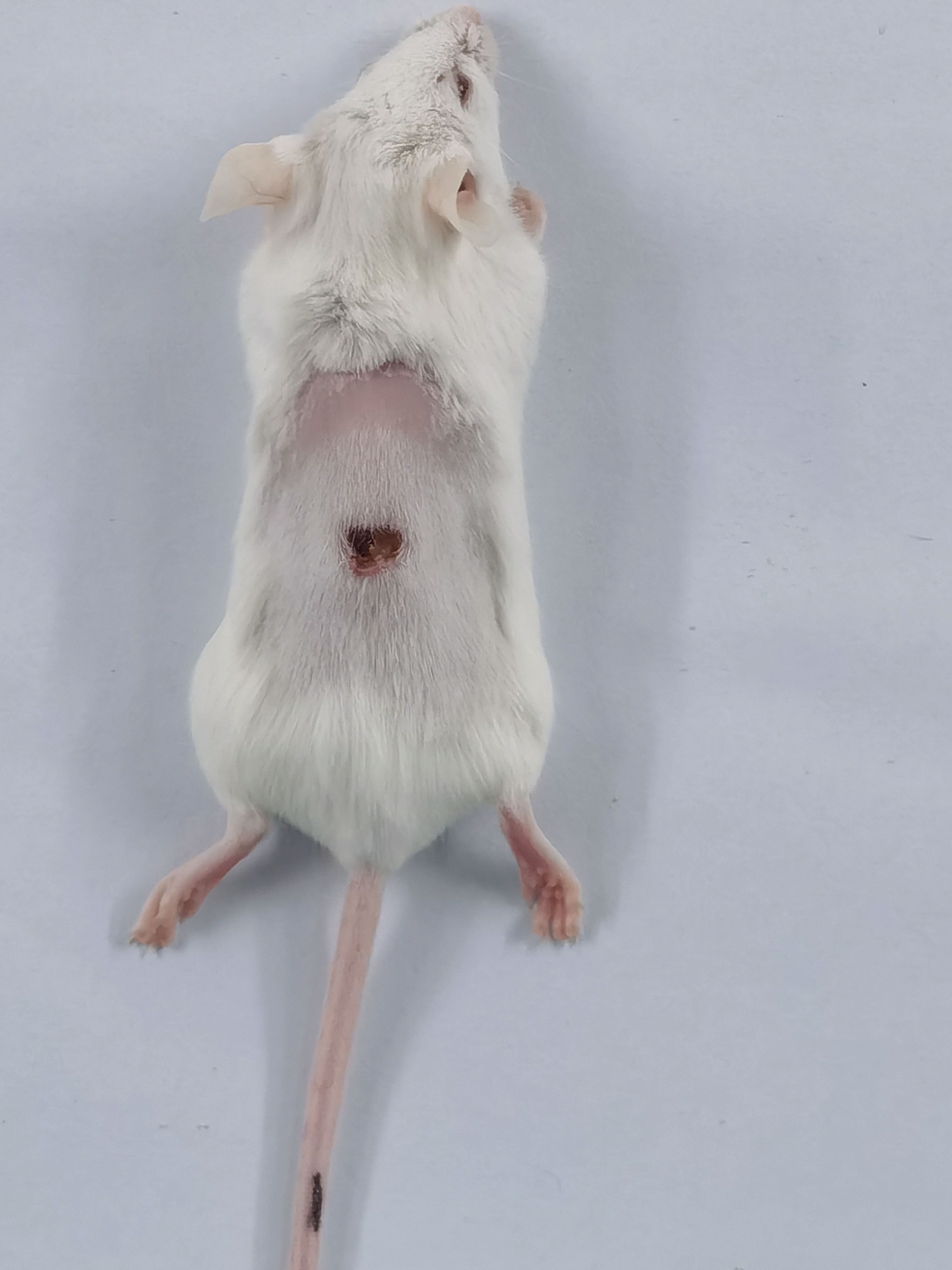

Supplement: Supplementary file 11 — Source data Fig. 6 [file 44321_2026_418_MOESM11_ESM.zip › Figure 6/Data-Figure 6B/Day 3/2-1.jpg]

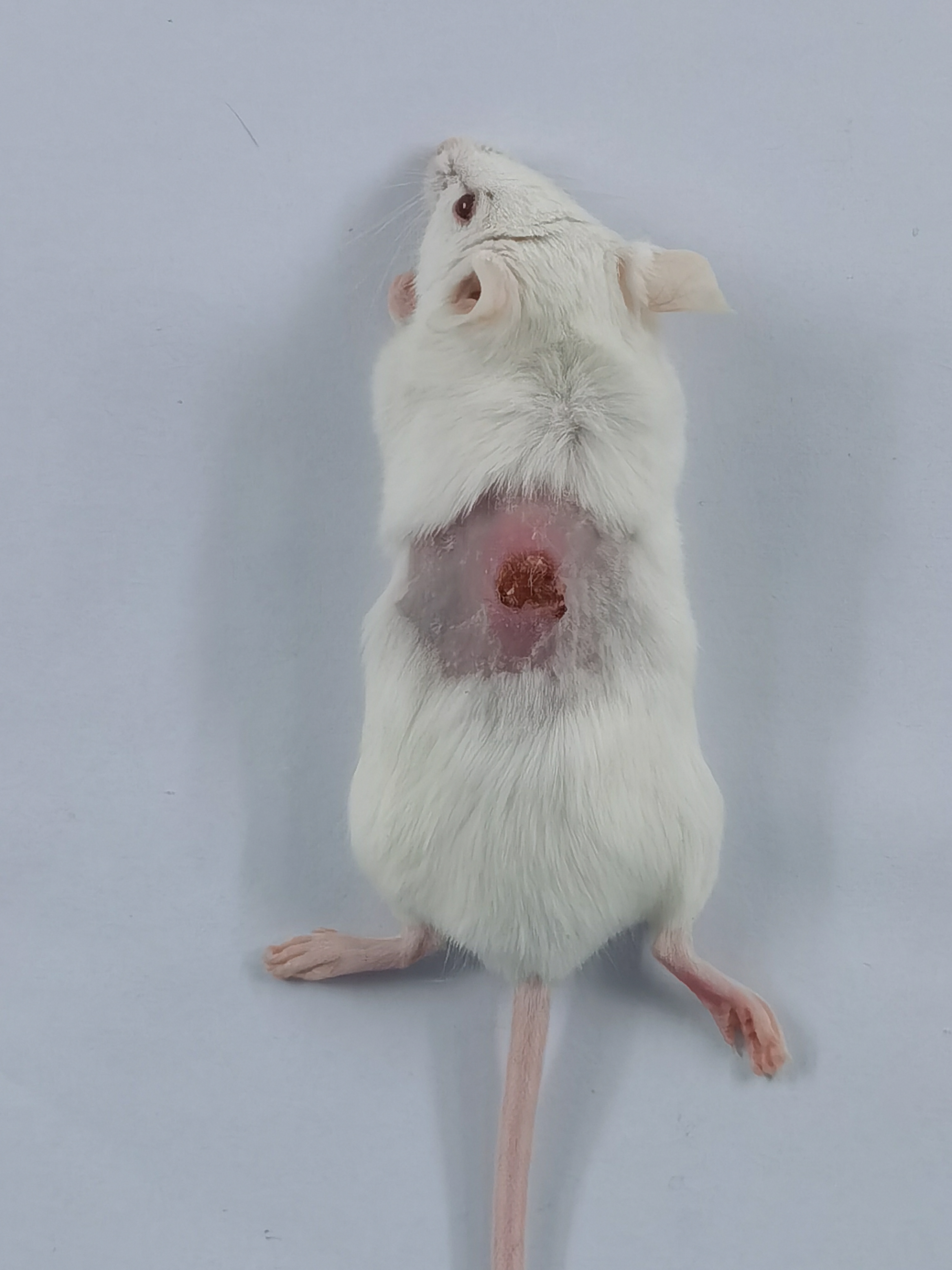

Supplement: Supplementary file 11 — Source data Fig. 6 [file 44321_2026_418_MOESM11_ESM.zip › Figure 6/Data-Figure 6B/Day 3/4-5.jpg]

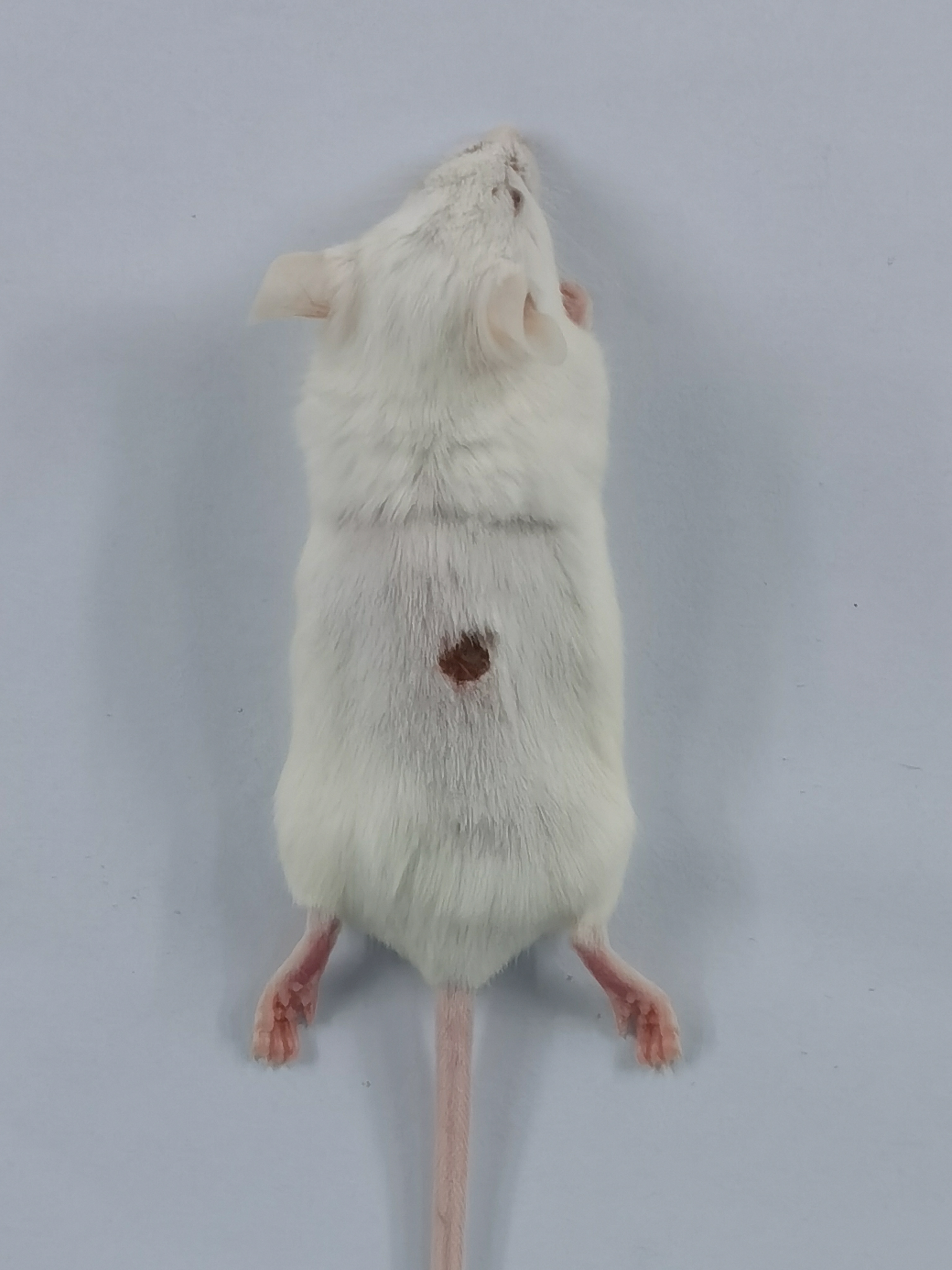

Supplement: Supplementary file 11 — Source data Fig. 6 [file 44321_2026_418_MOESM11_ESM.zip › Figure 6/Data-Figure 6B/Day 3/2-3.jpg]

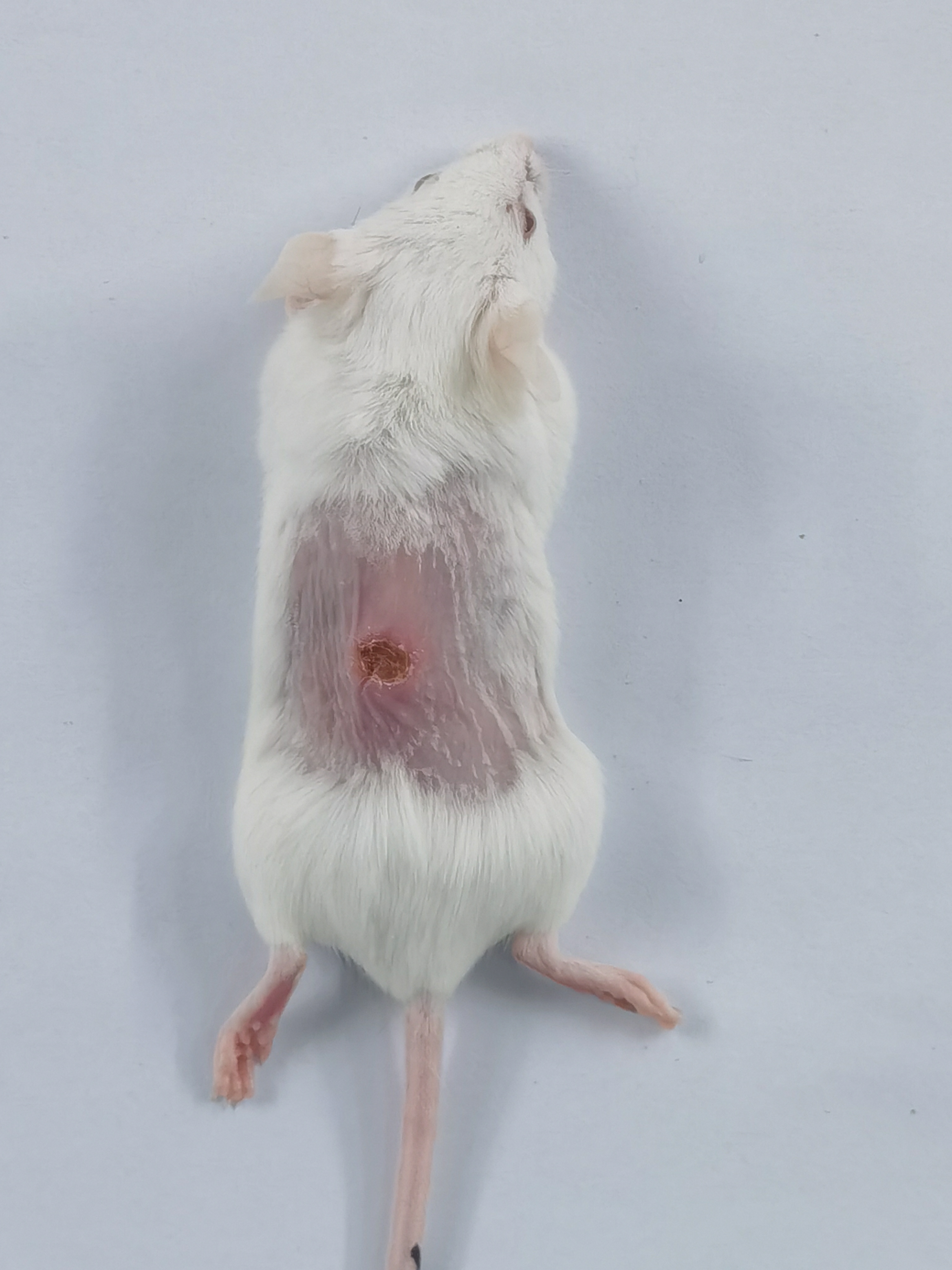

Supplement: Supplementary file 11 — Source data Fig. 6 [file 44321_2026_418_MOESM11_ESM.zip › Figure 6/Data-Figure 6B/Day 3/2-2.jpg]

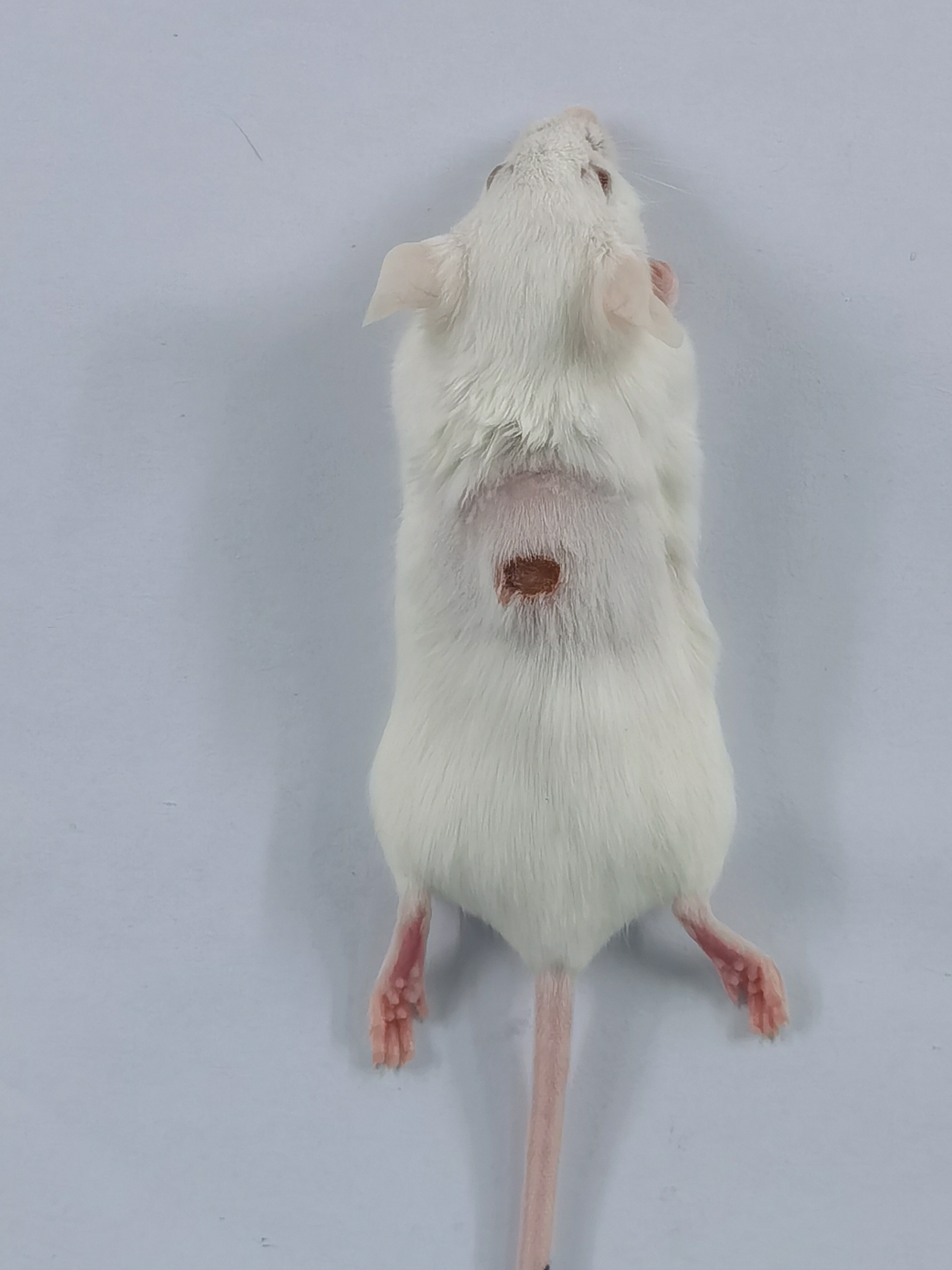

Supplement: Supplementary file 11 — Source data Fig. 6 [file 44321_2026_418_MOESM11_ESM.zip › Figure 6/Data-Figure 6B/Day 3/4-4.jpg]

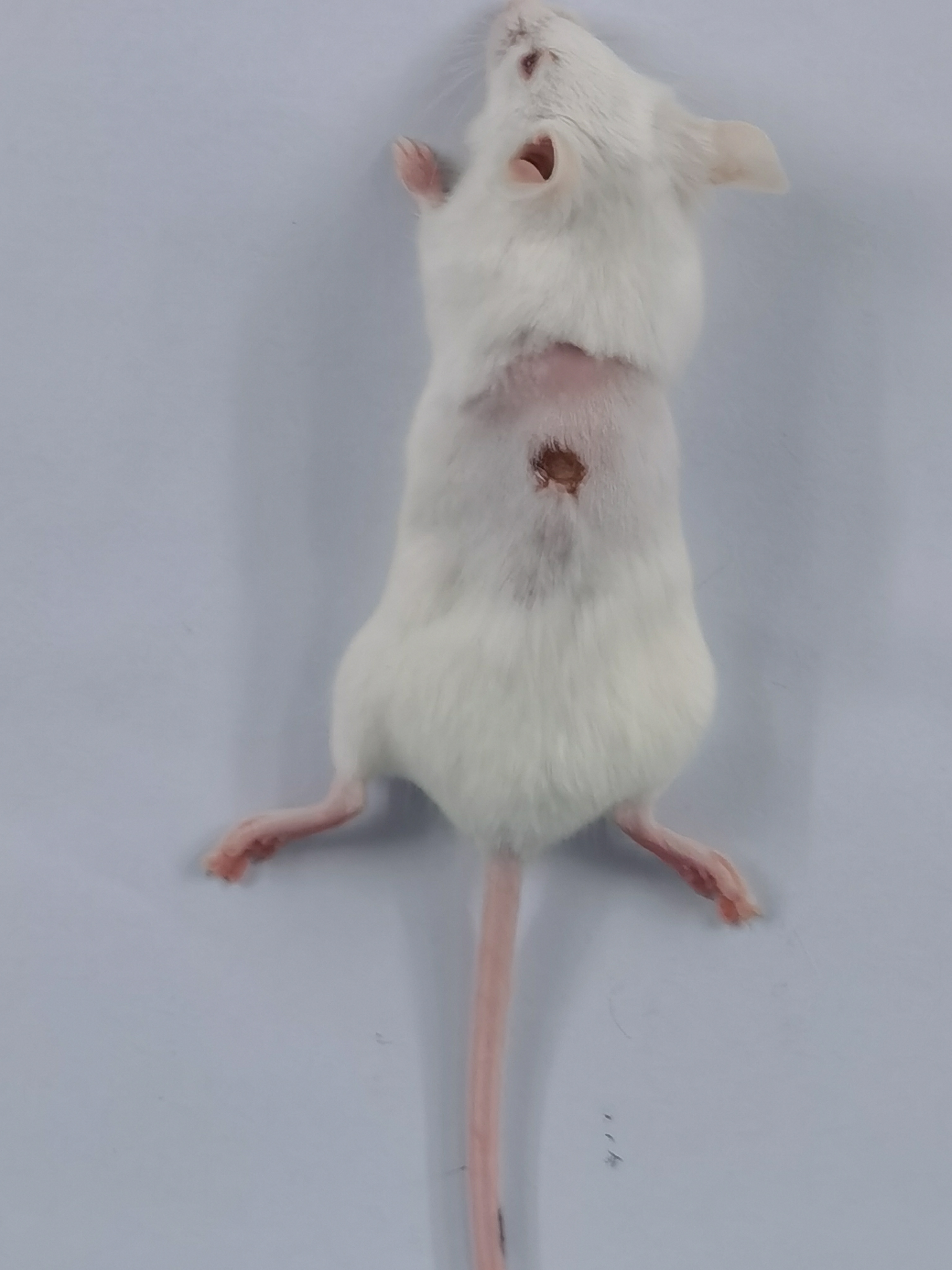

Supplement: Supplementary file 11 — Source data Fig. 6 [file 44321_2026_418_MOESM11_ESM.zip › Figure 6/Data-Figure 6B/Day 3/4-1.jpg]

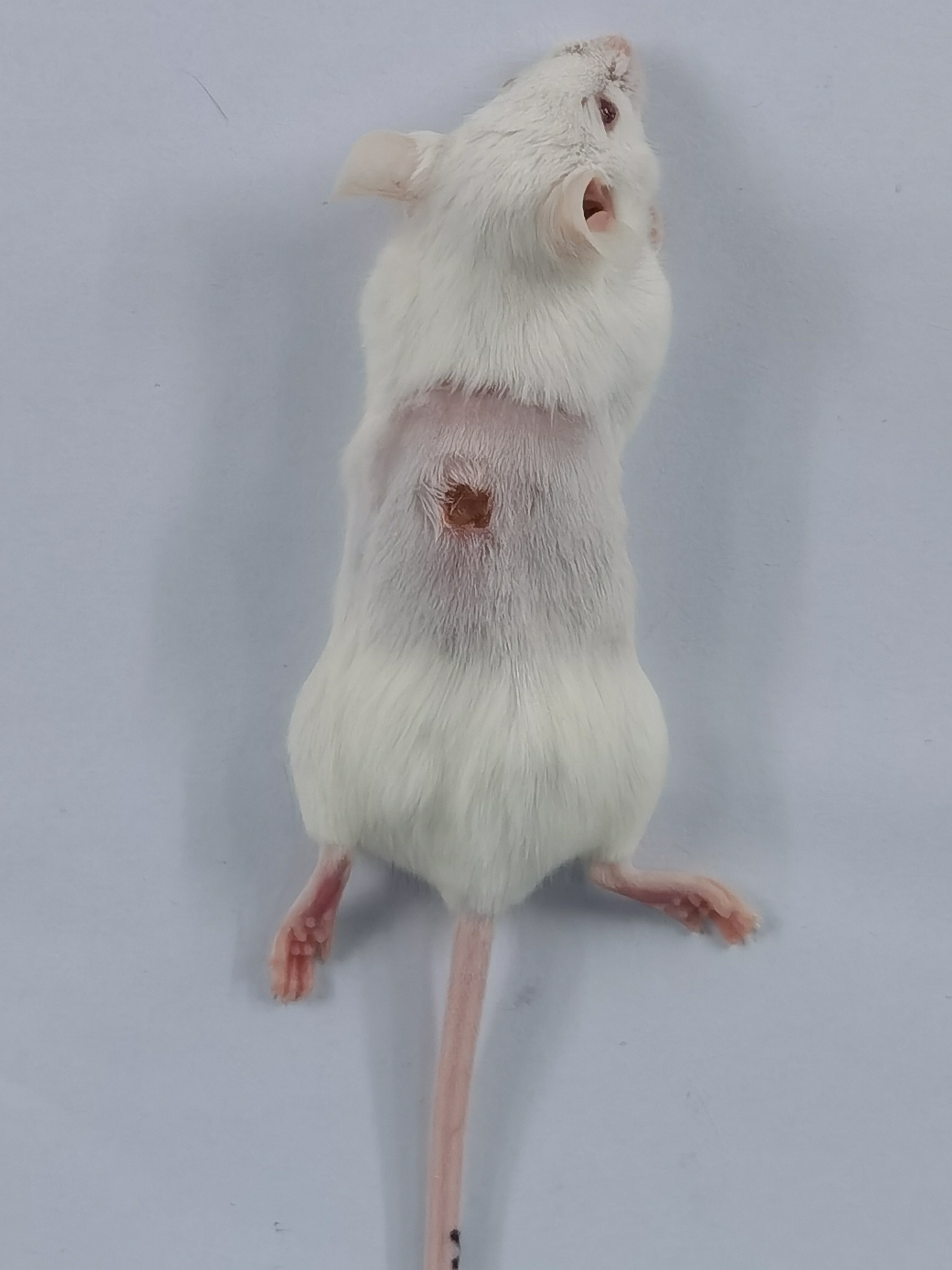

Supplement: Supplementary file 11 — Source data Fig. 6 [file 44321_2026_418_MOESM11_ESM.zip › Figure 6/Data-Figure 6B/Day 3/4-3.jpg]

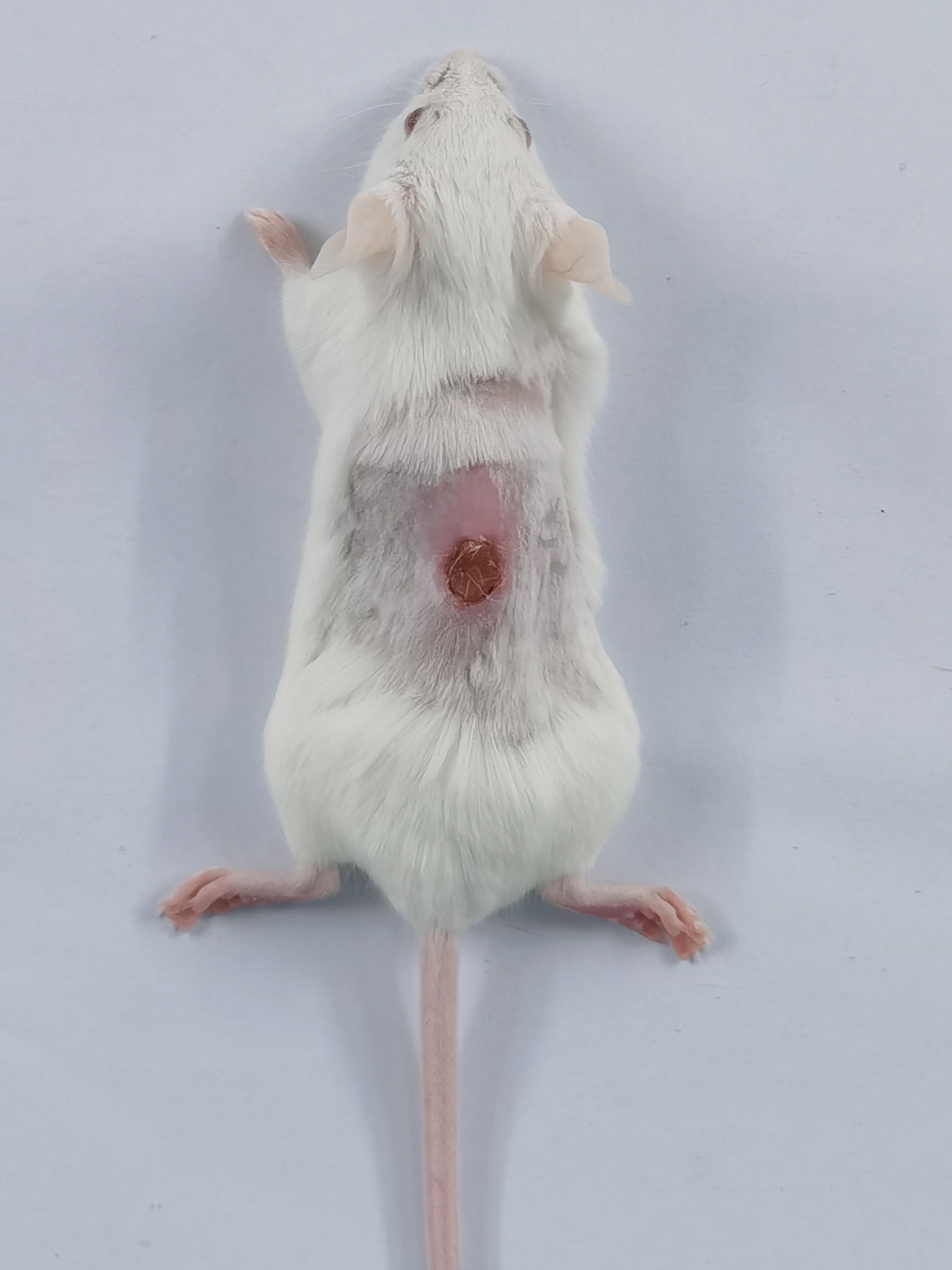

Supplement: Supplementary file 11 — Source data Fig. 6 [file 44321_2026_418_MOESM11_ESM.zip › Figure 6/Data-Figure 6B/Day 3/2-5.jpg]

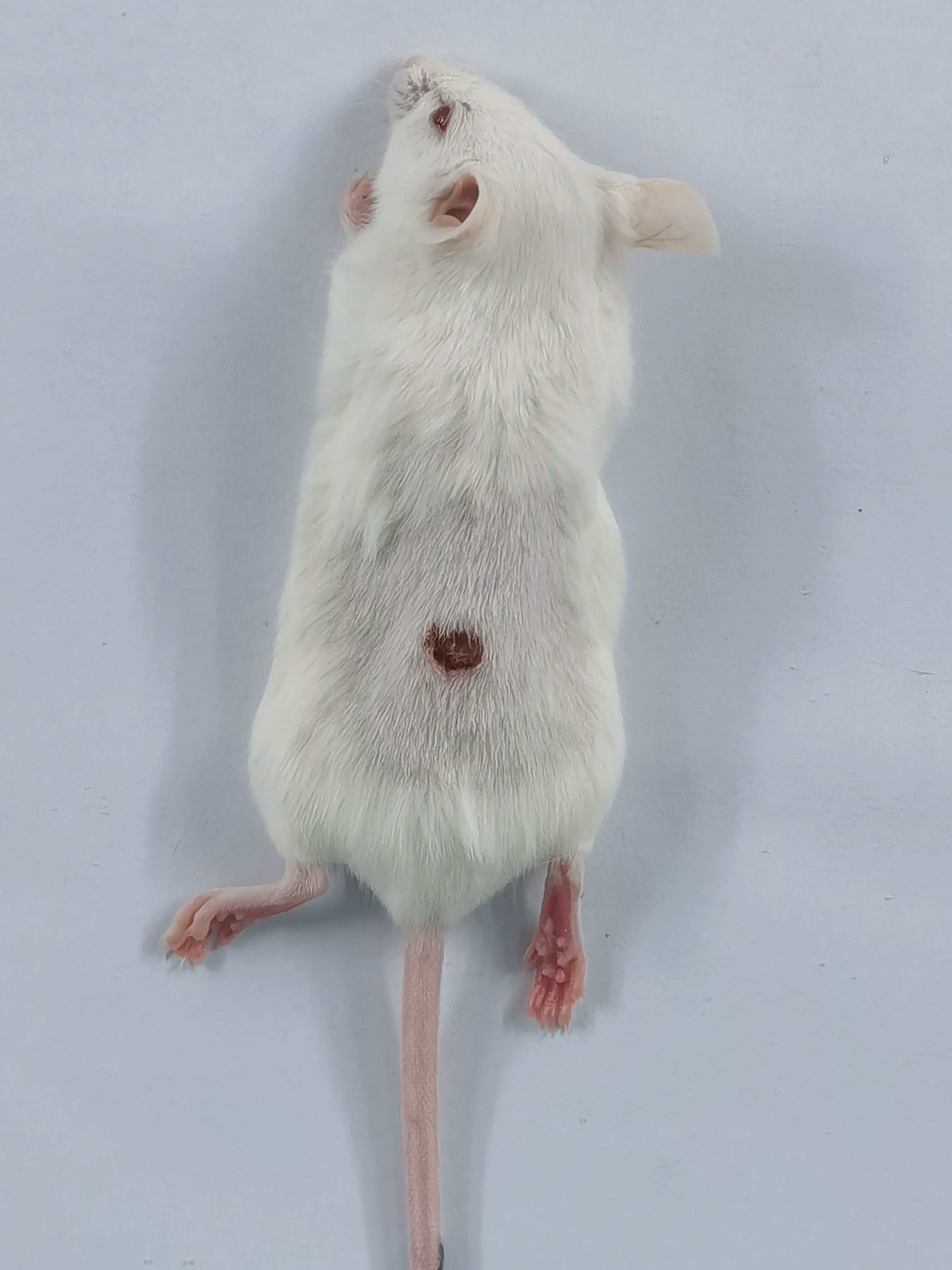

Supplement: Supplementary file 11 — Source data Fig. 6 [file 44321_2026_418_MOESM11_ESM.zip › Figure 6/Data-Figure 6B/Day 3/2-4.jpg]

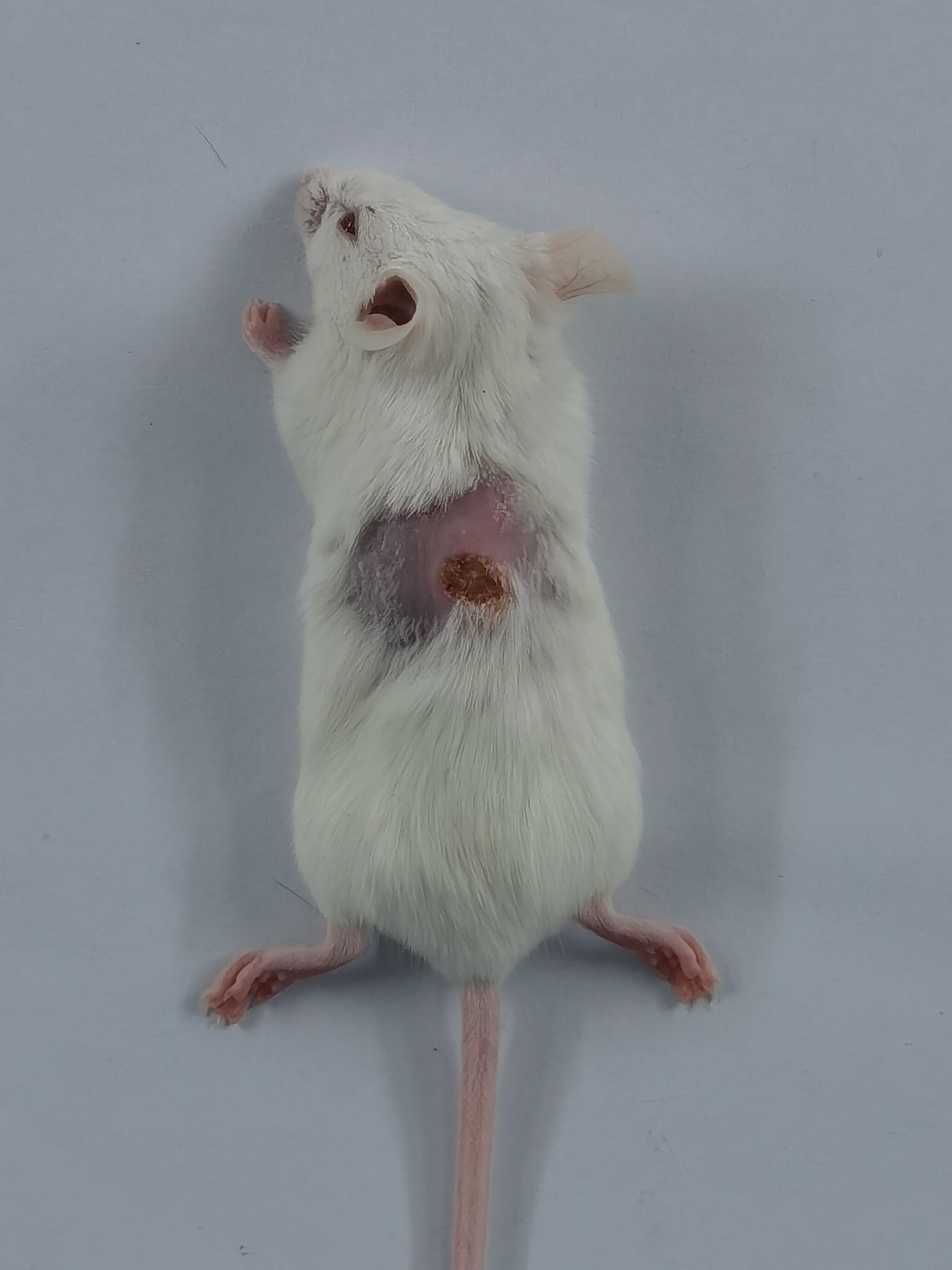

Supplement: Supplementary file 11 — Source data Fig. 6 [file 44321_2026_418_MOESM11_ESM.zip › Figure 6/Data-Figure 6B/Day 3/4-2.jpg]

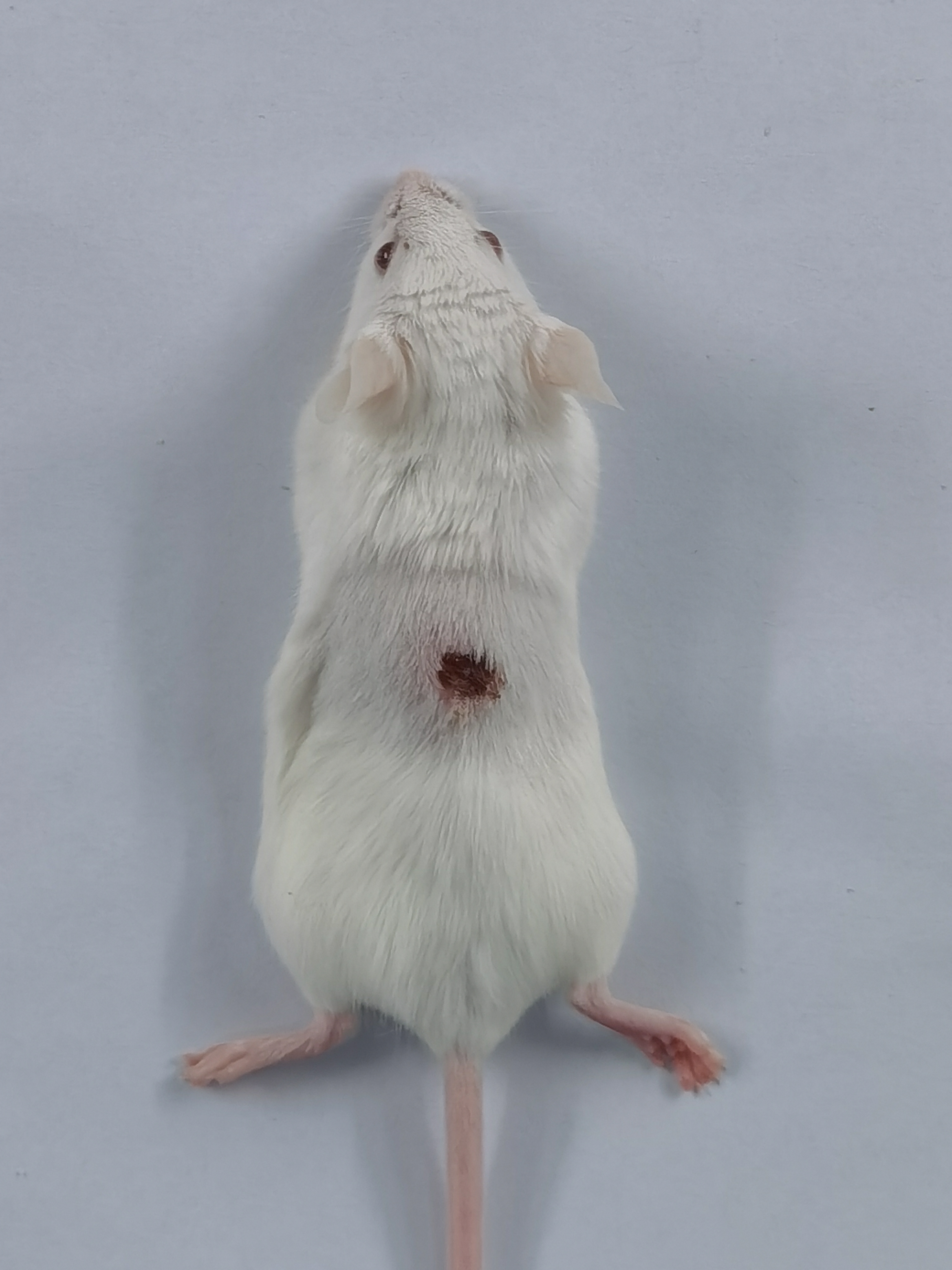

Supplement: Supplementary file 11 — Source data Fig. 6 [file 44321_2026_418_MOESM11_ESM.zip › Figure 6/Data-Figure 6B/Day 3/3-3.jpg]

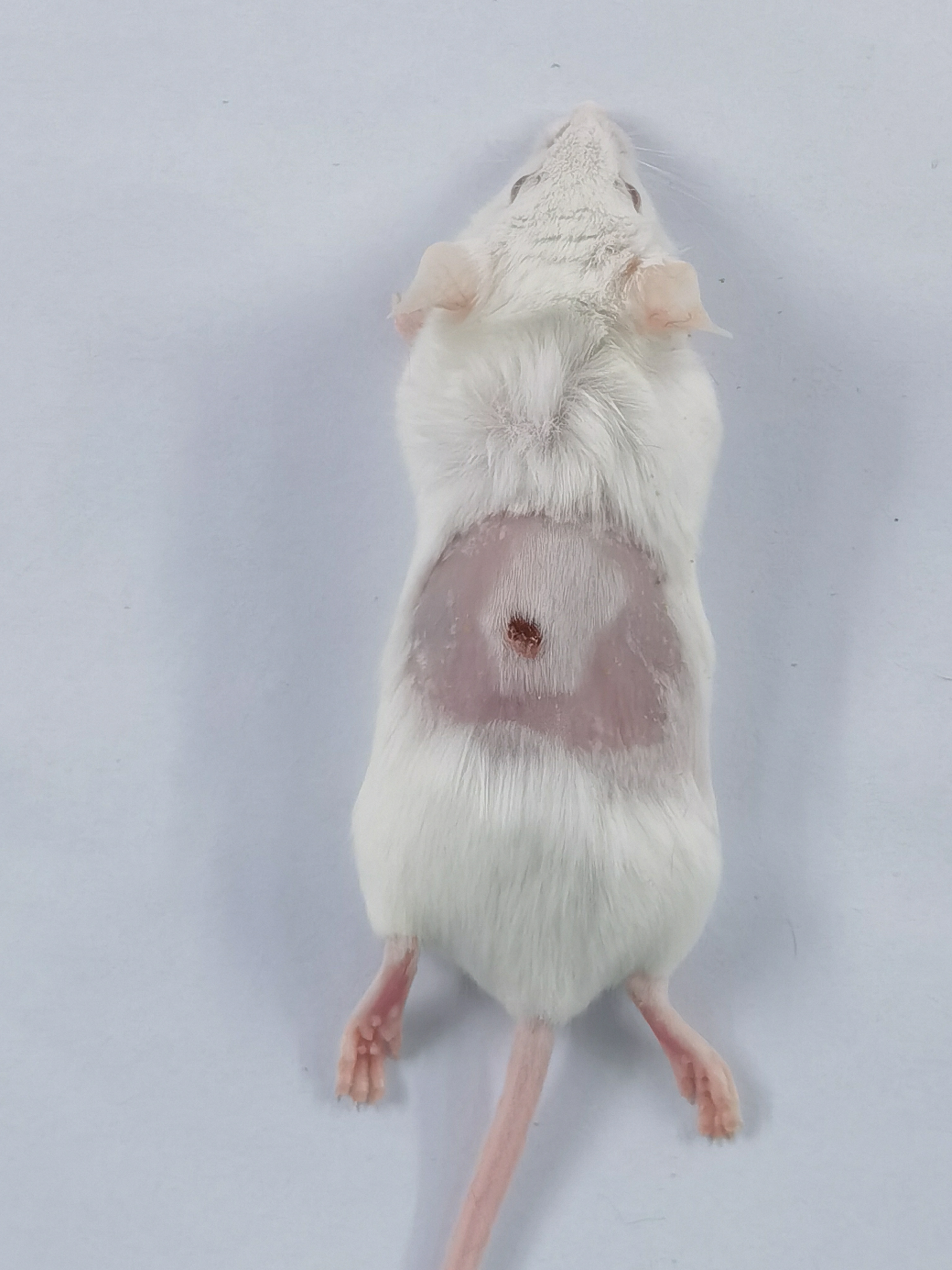

Supplement: Supplementary file 11 — Source data Fig. 6 [file 44321_2026_418_MOESM11_ESM.zip › Figure 6/Data-Figure 6B/Day 3/1-1.jpg]

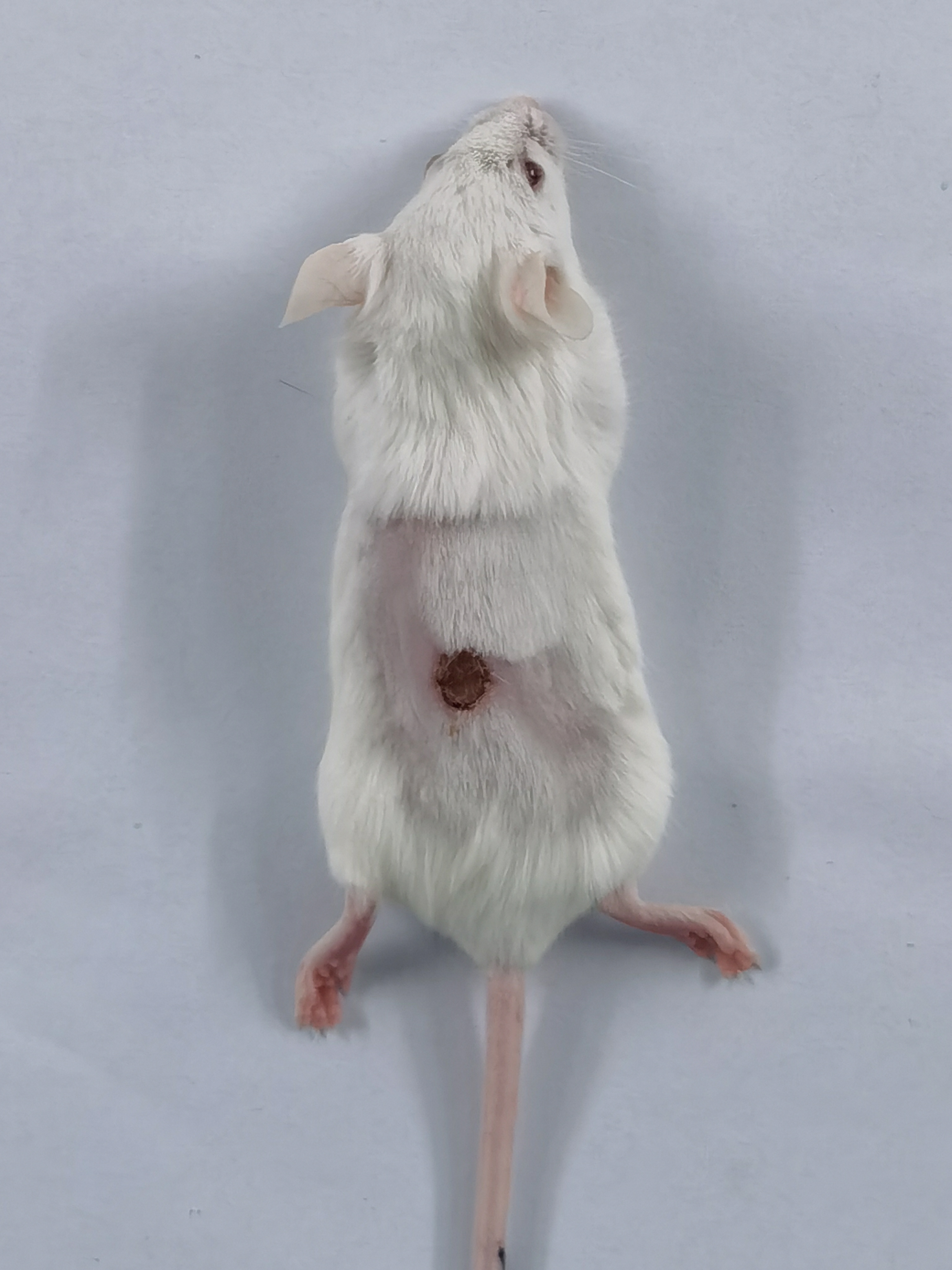

Supplement: Supplementary file 11 — Source data Fig. 6 [file 44321_2026_418_MOESM11_ESM.zip › Figure 6/Data-Figure 6B/Day 3/3-2.jpg]

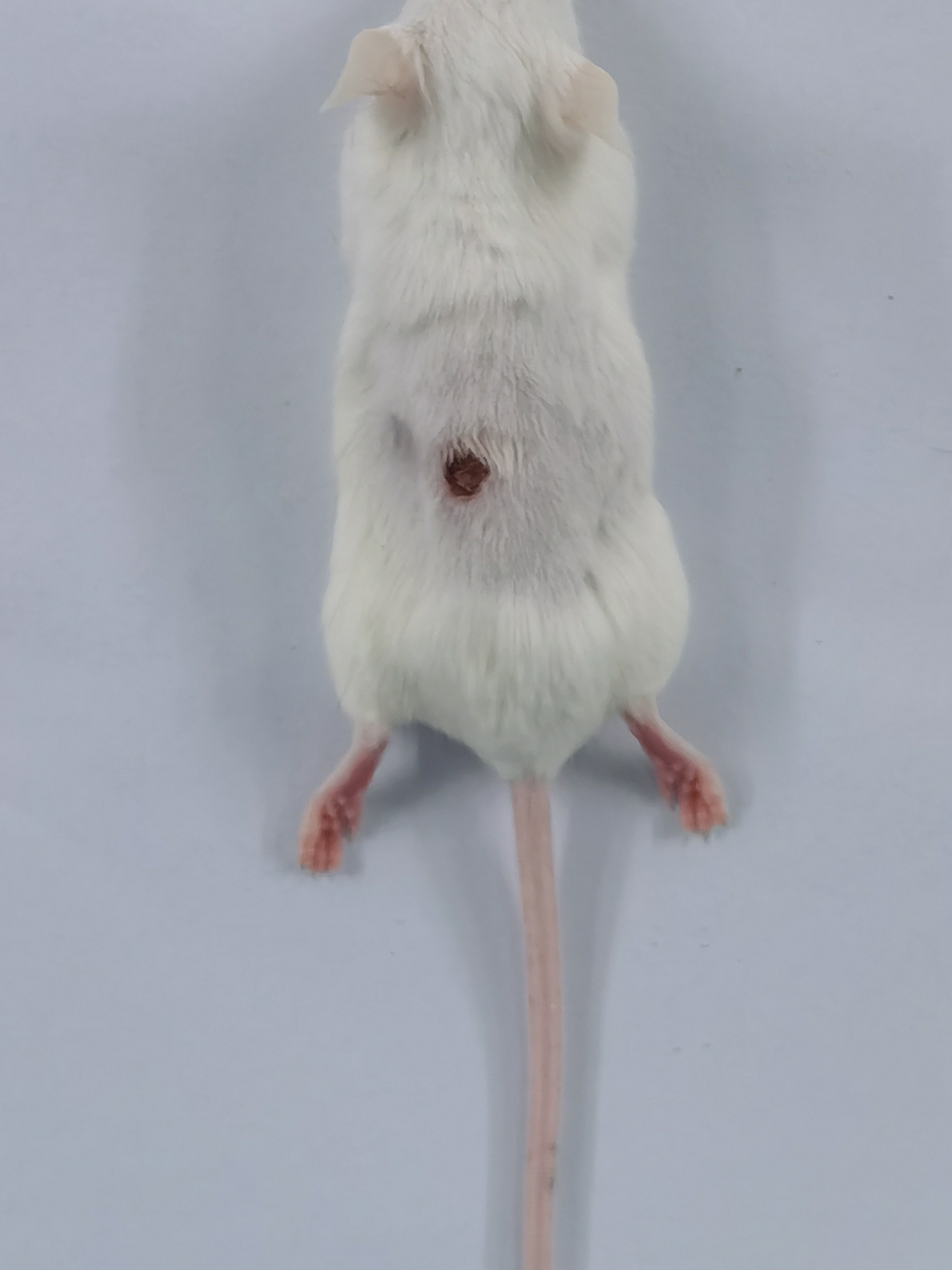

Supplement: Supplementary file 11 — Source data Fig. 6 [file 44321_2026_418_MOESM11_ESM.zip › Figure 6/Data-Figure 6B/Day 3/1-2.jpg]

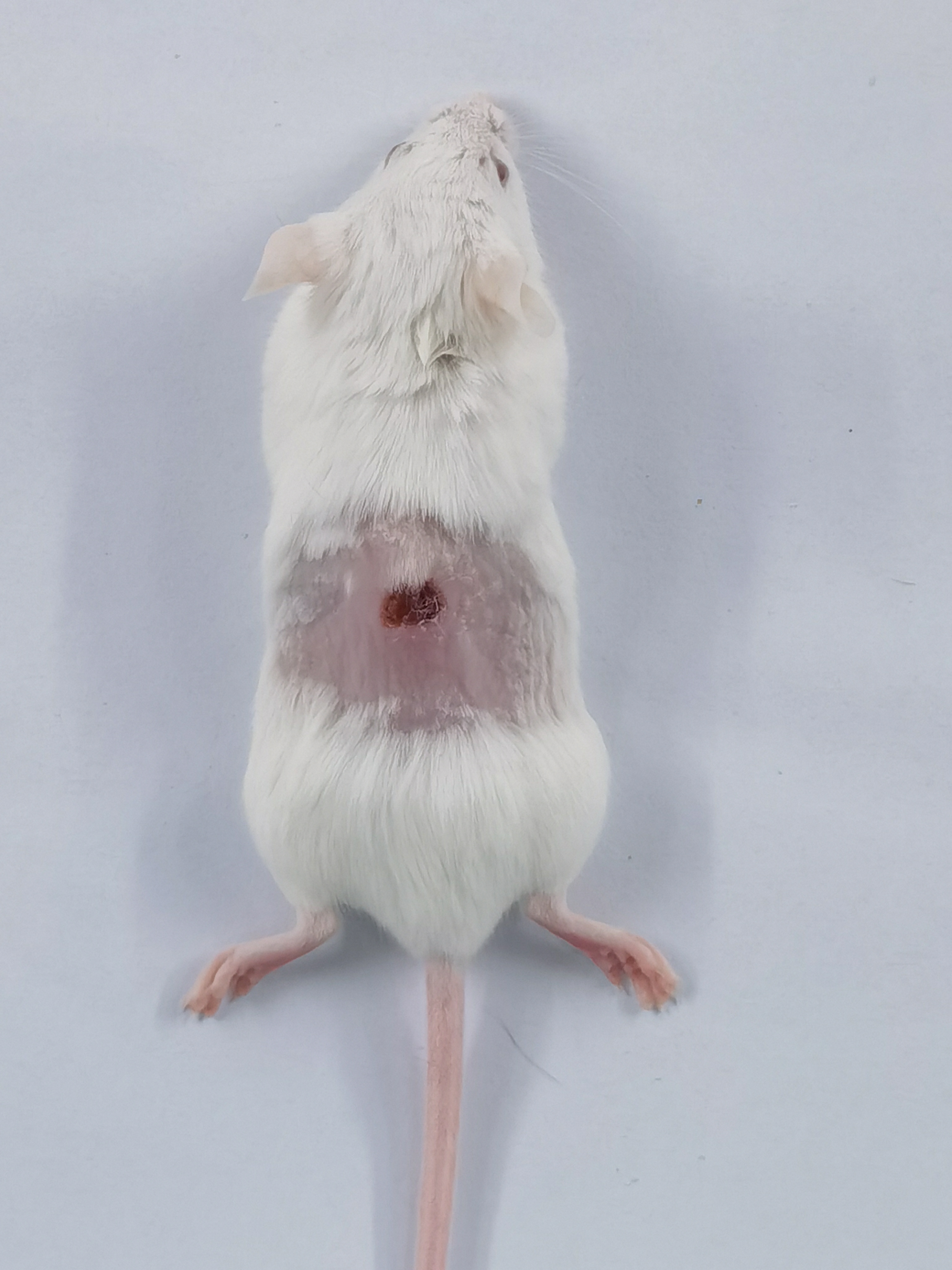

Supplement: Supplementary file 11 — Source data Fig. 6 [file 44321_2026_418_MOESM11_ESM.zip › Figure 6/Data-Figure 6B/Day 3/1-3.jpg]

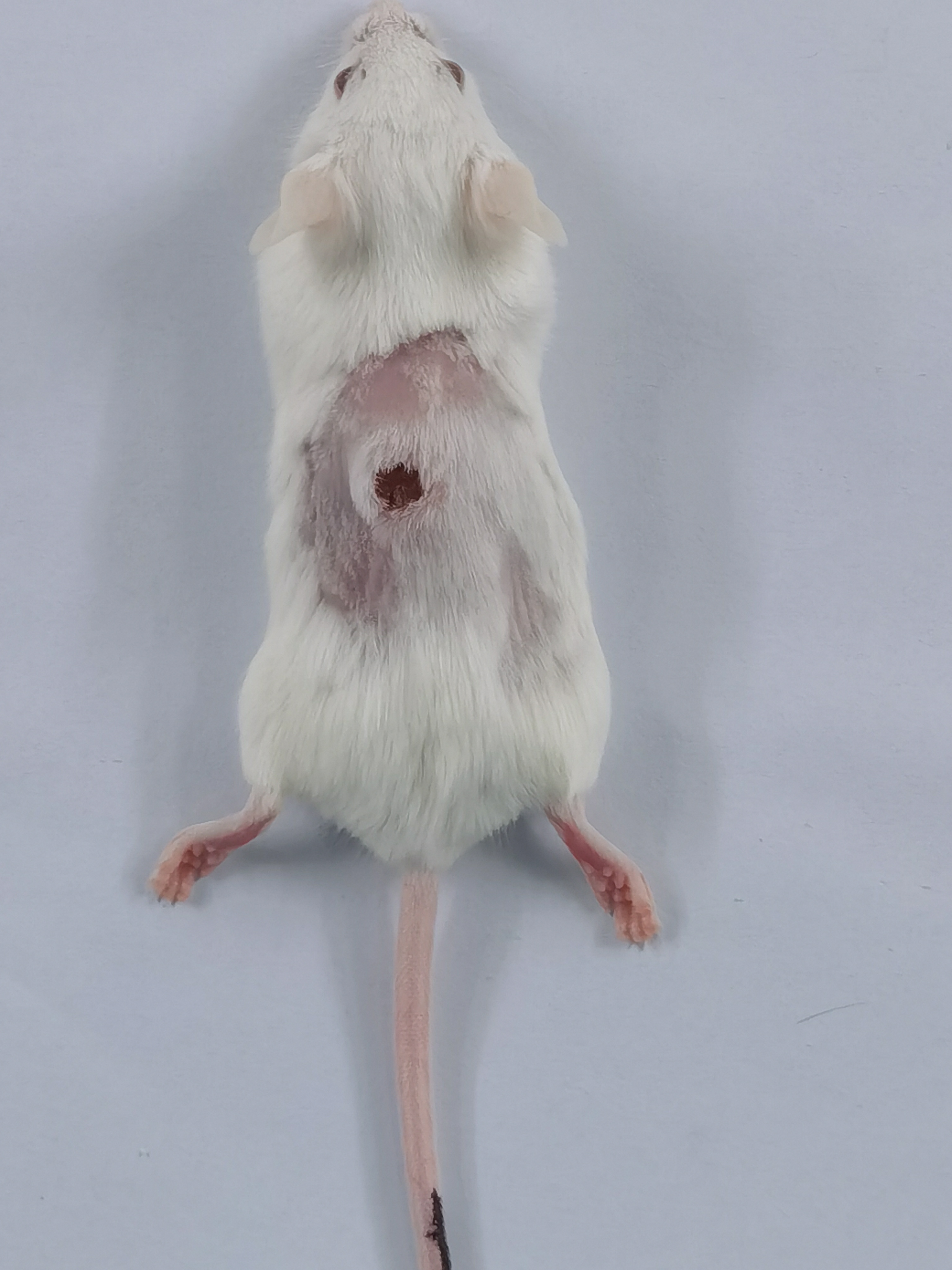

Supplement: Supplementary file 11 — Source data Fig. 6 [file 44321_2026_418_MOESM11_ESM.zip › Figure 6/Data-Figure 6B/Day 3/3-1.jpg]

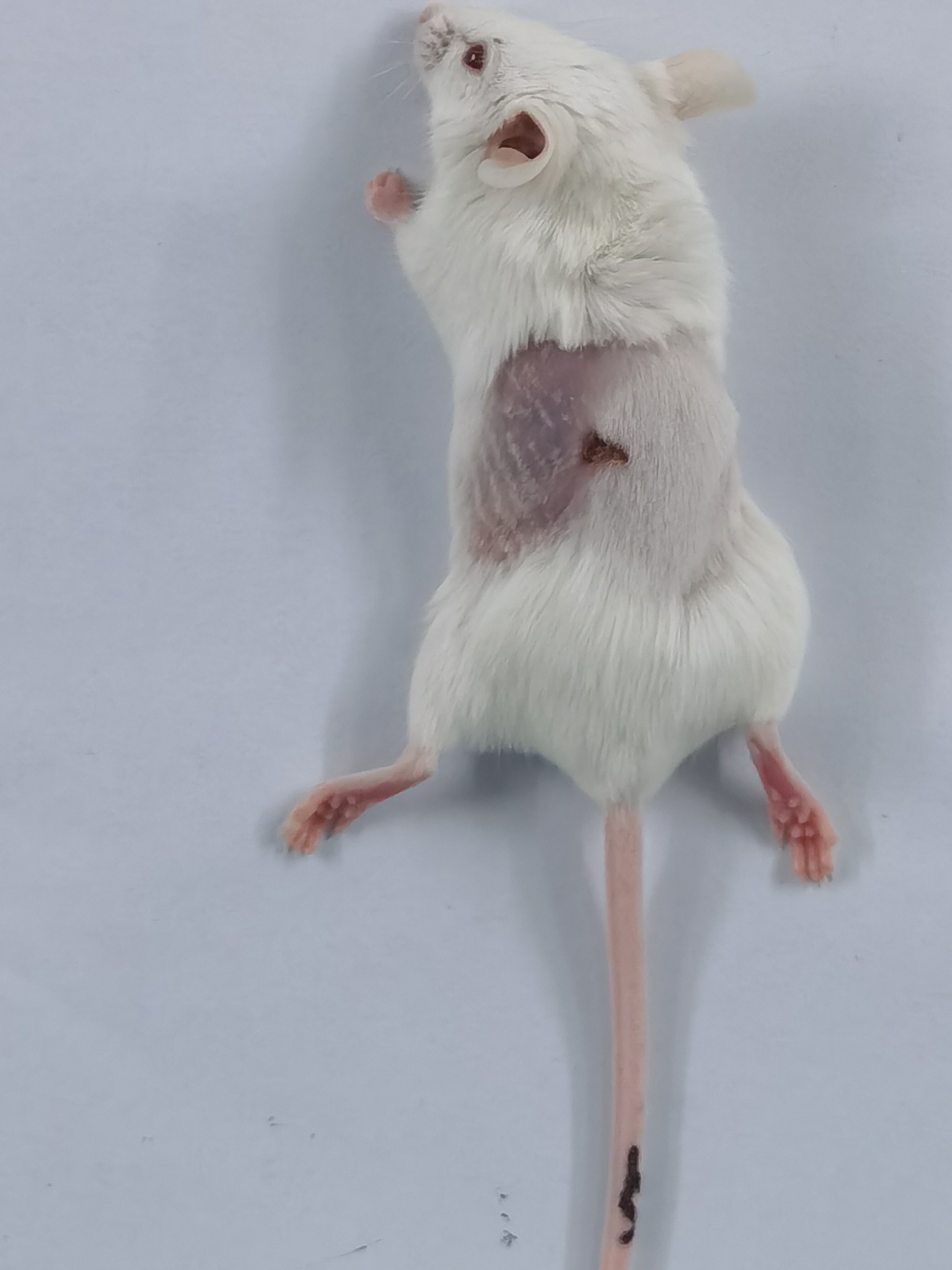

Supplement: Supplementary file 11 — Source data Fig. 6 [file 44321_2026_418_MOESM11_ESM.zip › Figure 6/Data-Figure 6B/Day 3/3-5.jpg]

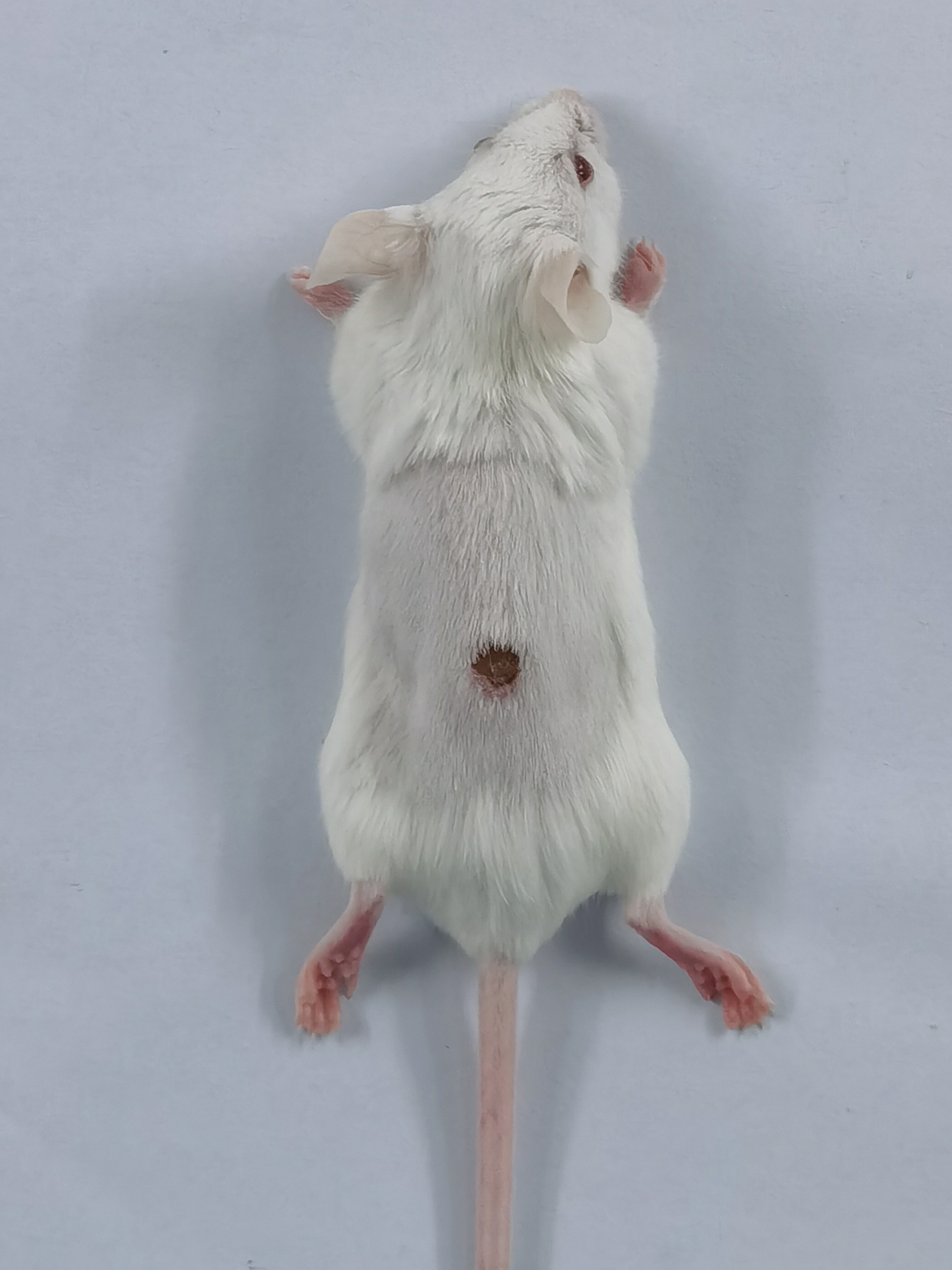

Supplement: Supplementary file 11 — Source data Fig. 6 [file 44321_2026_418_MOESM11_ESM.zip › Figure 6/Data-Figure 6B/Day 3/3-4.jpg]

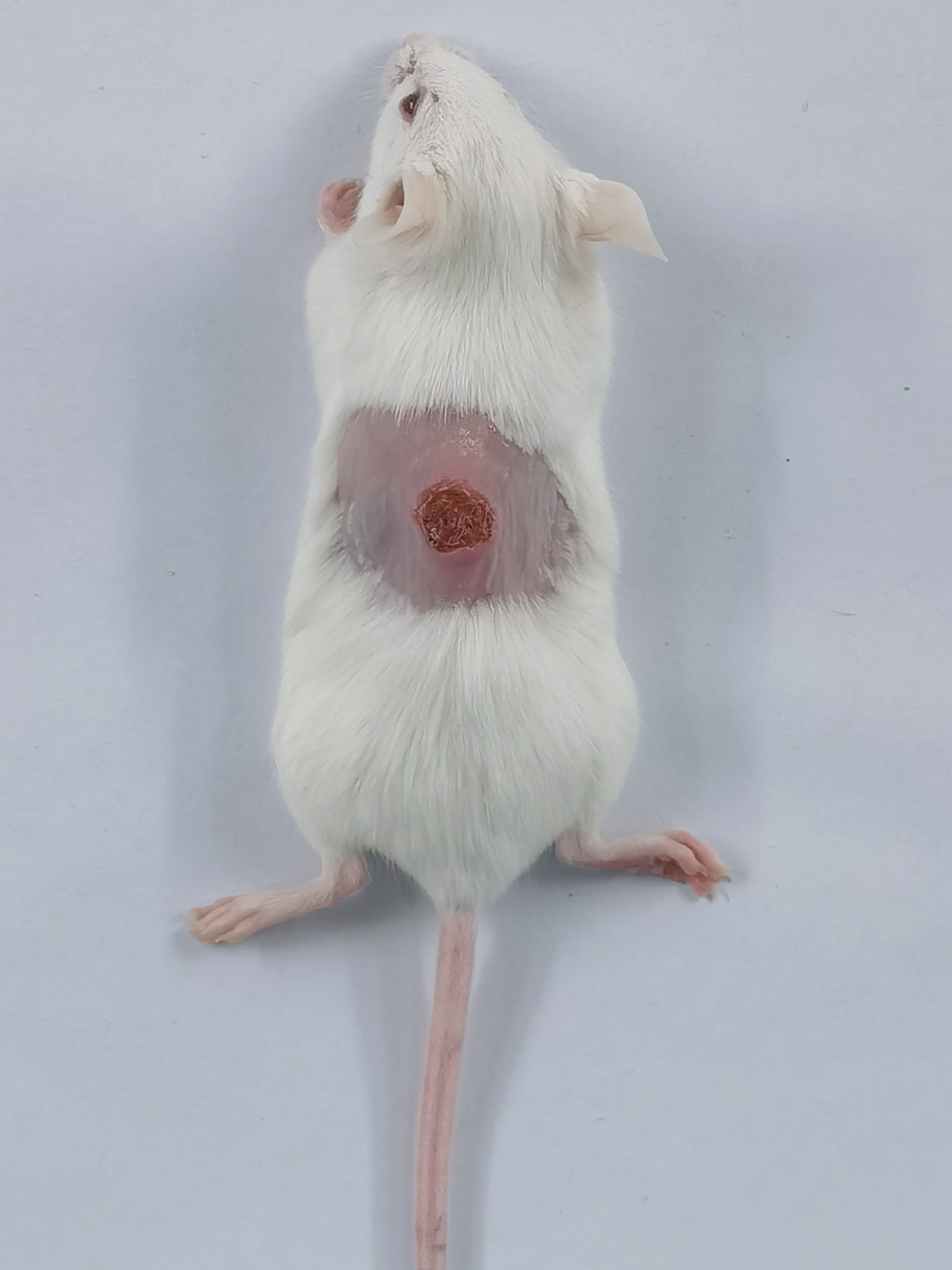

Supplement: Supplementary file 11 — Source data Fig. 6 [file 44321_2026_418_MOESM11_ESM.zip › Figure 6/Data-Figure 6B/Day 3/1-4.jpg]

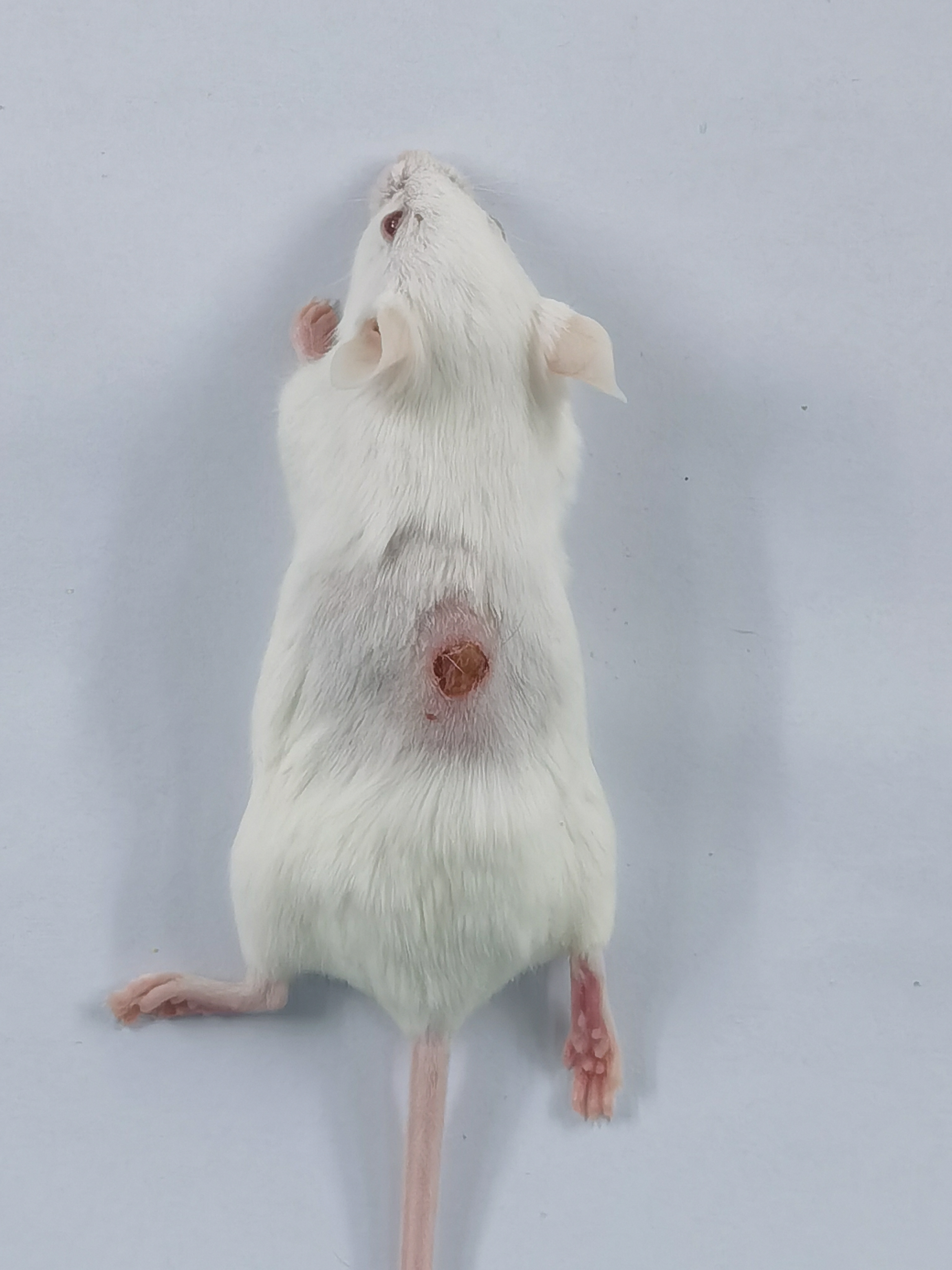

Supplement: Supplementary file 11 — Source data Fig. 6 [file 44321_2026_418_MOESM11_ESM.zip › Figure 6/Data-Figure 6B/Day 3/1-5.jpg]

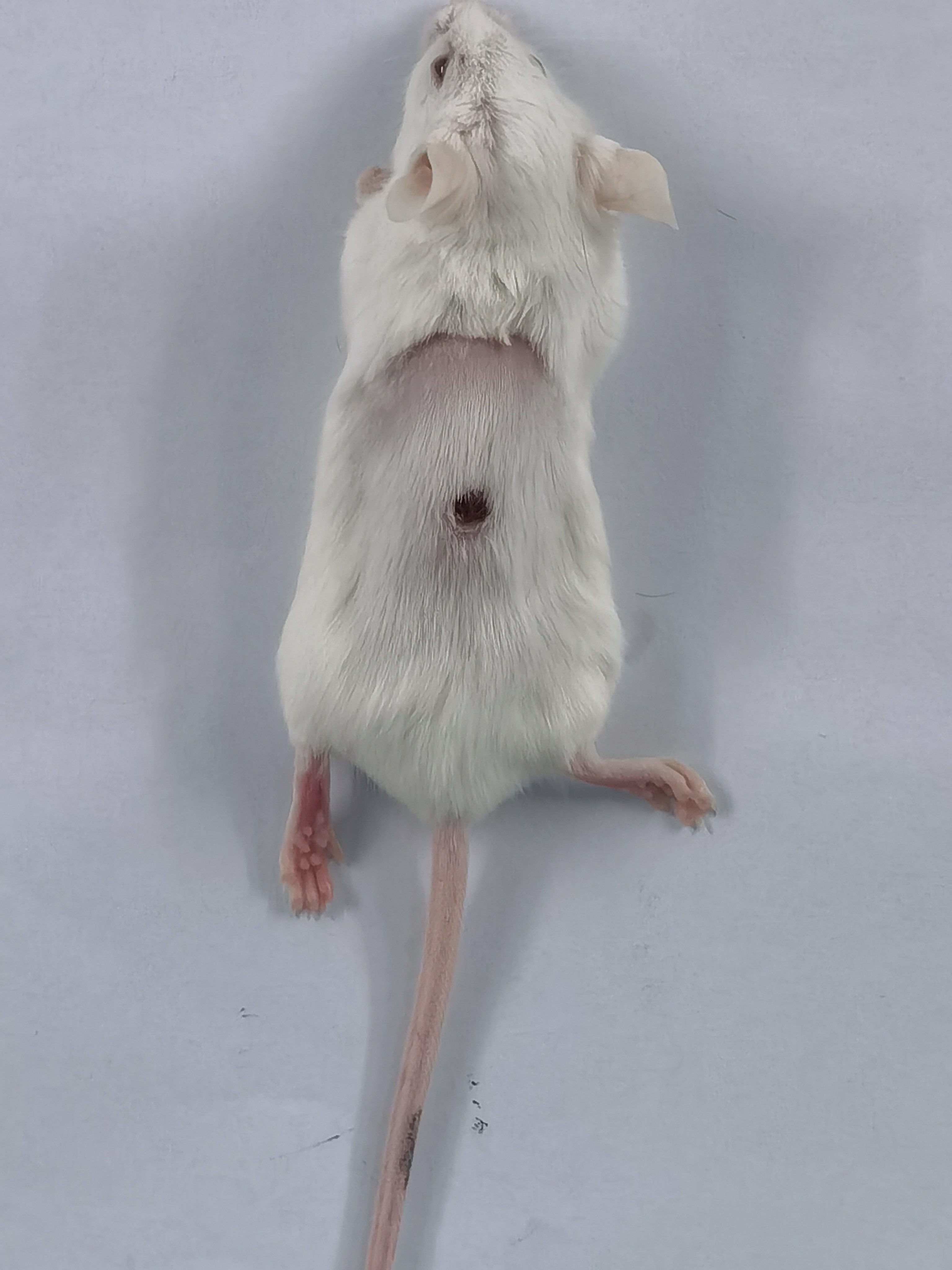

Supplement: Supplementary file 11 — Source data Fig. 6 [file 44321_2026_418_MOESM11_ESM.zip › Figure 6/Data-Figure 6B/Day 6/2-1.jpg]

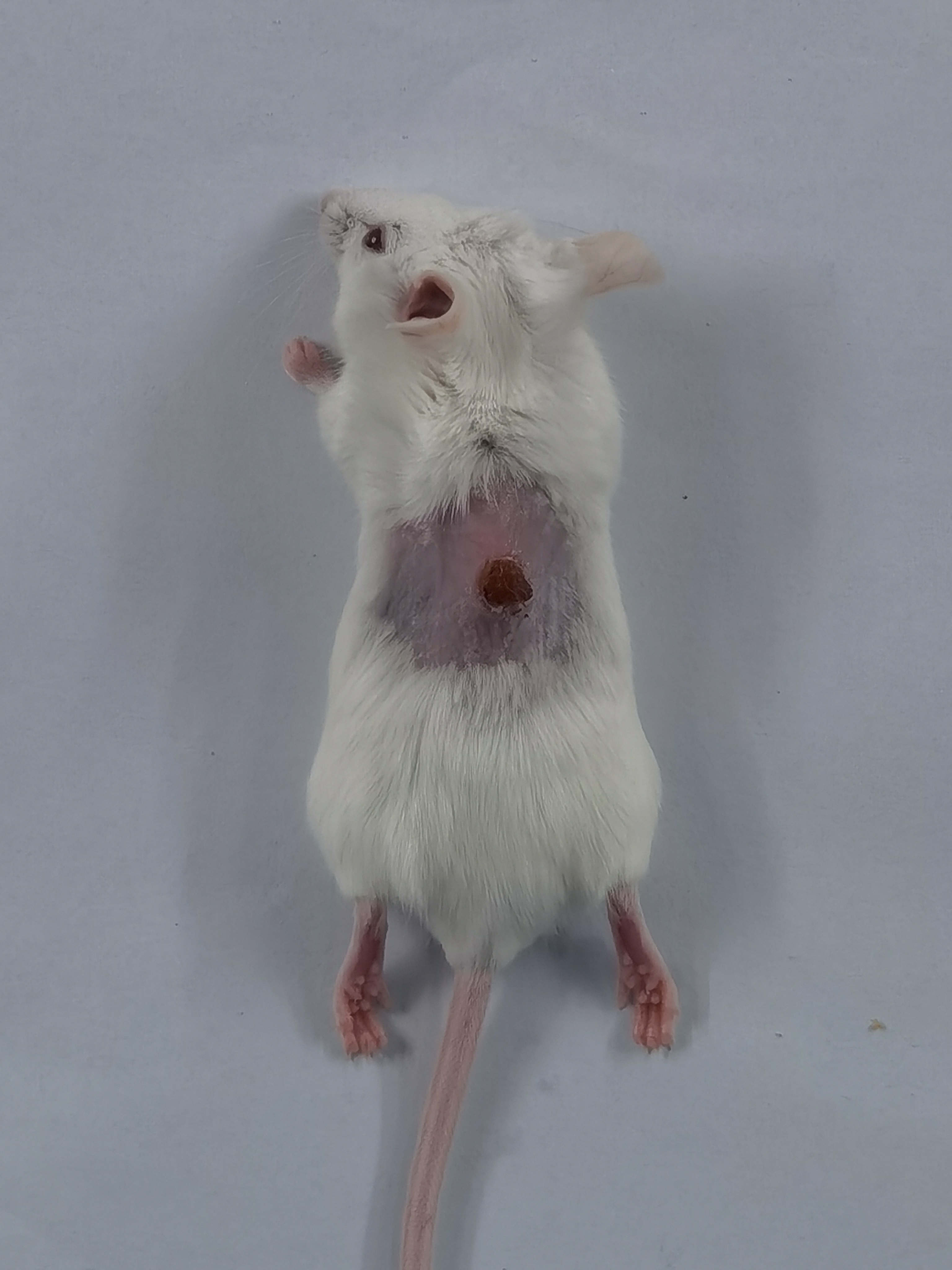

Supplement: Supplementary file 11 — Source data Fig. 6 [file 44321_2026_418_MOESM11_ESM.zip › Figure 6/Data-Figure 6B/Day 6/4-5.jpg]

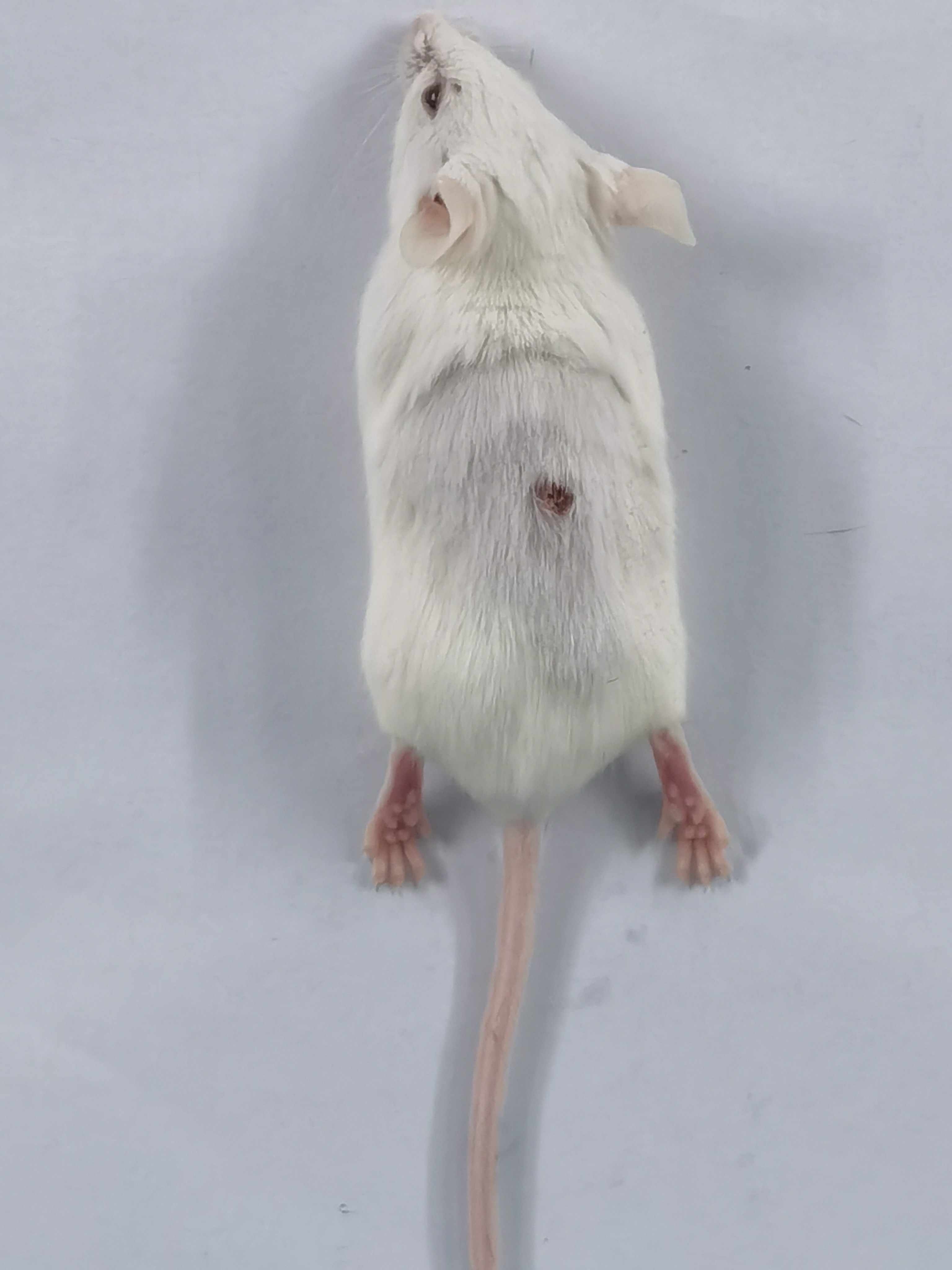

Supplement: Supplementary file 11 — Source data Fig. 6 [file 44321_2026_418_MOESM11_ESM.zip › Figure 6/Data-Figure 6B/Day 6/2-3.jpg]

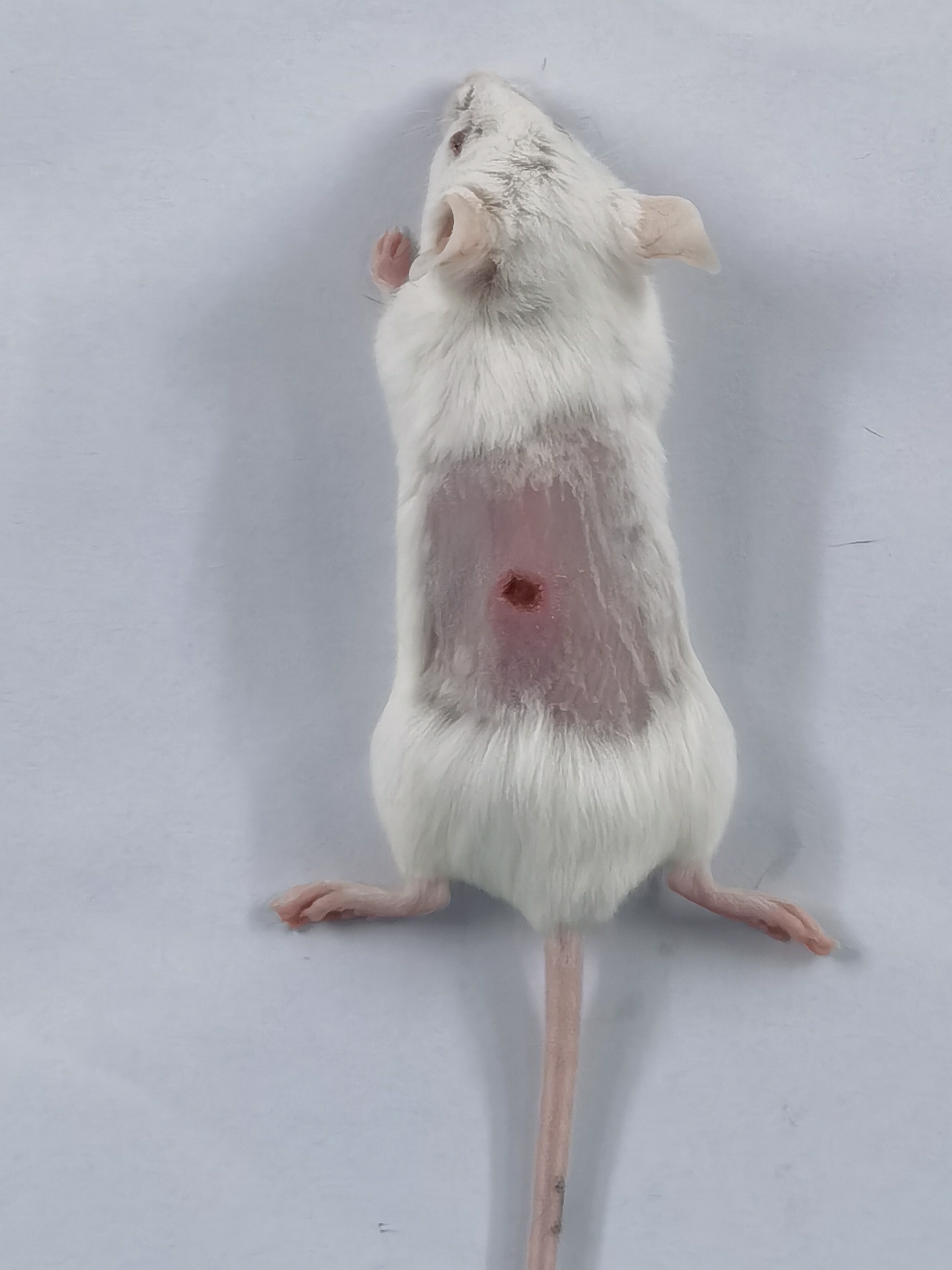

Supplement: Supplementary file 11 — Source data Fig. 6 [file 44321_2026_418_MOESM11_ESM.zip › Figure 6/Data-Figure 6B/Day 6/2-2.jpg]

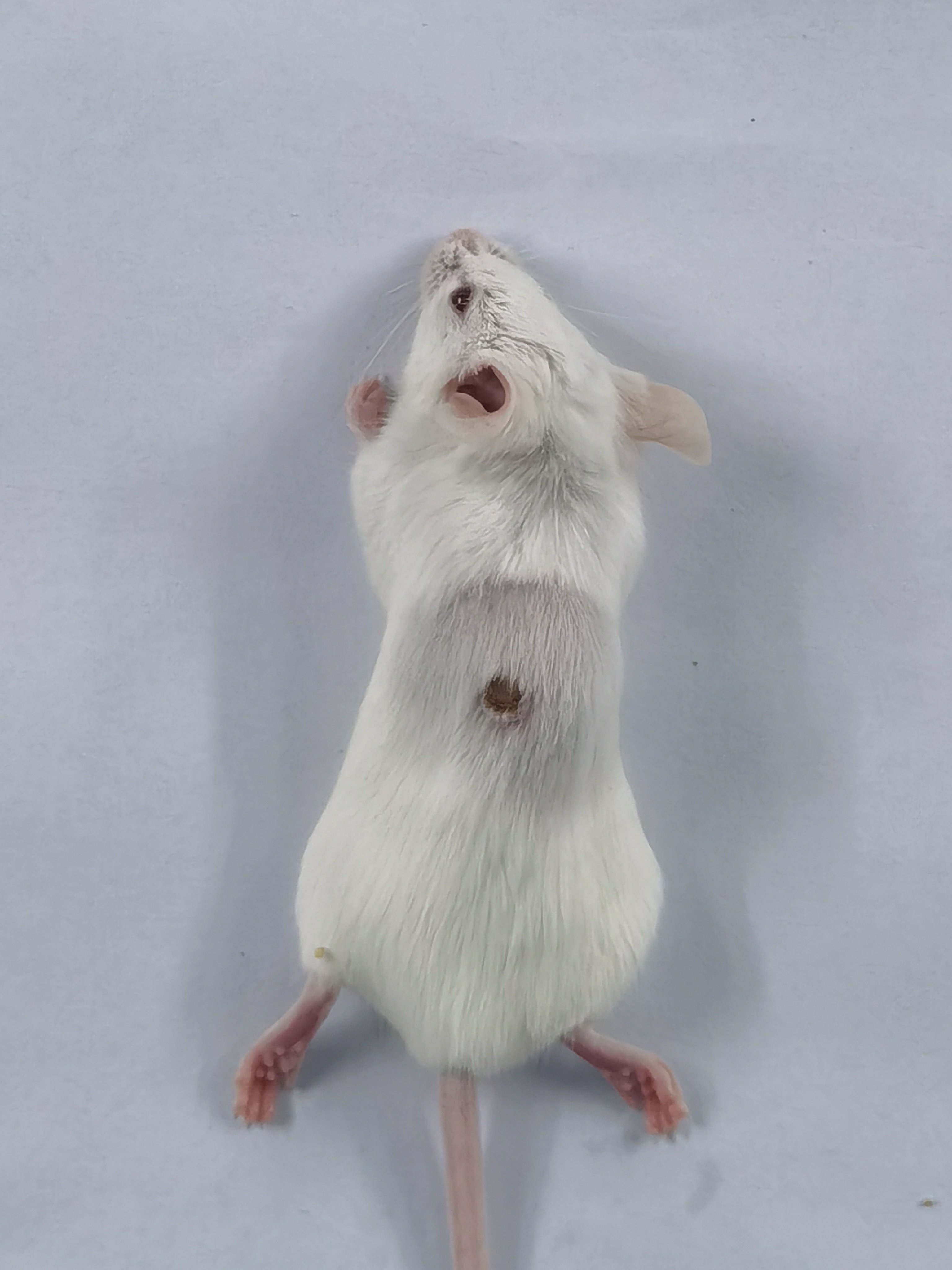

Supplement: Supplementary file 11 — Source data Fig. 6 [file 44321_2026_418_MOESM11_ESM.zip › Figure 6/Data-Figure 6B/Day 6/4-4.jpg]

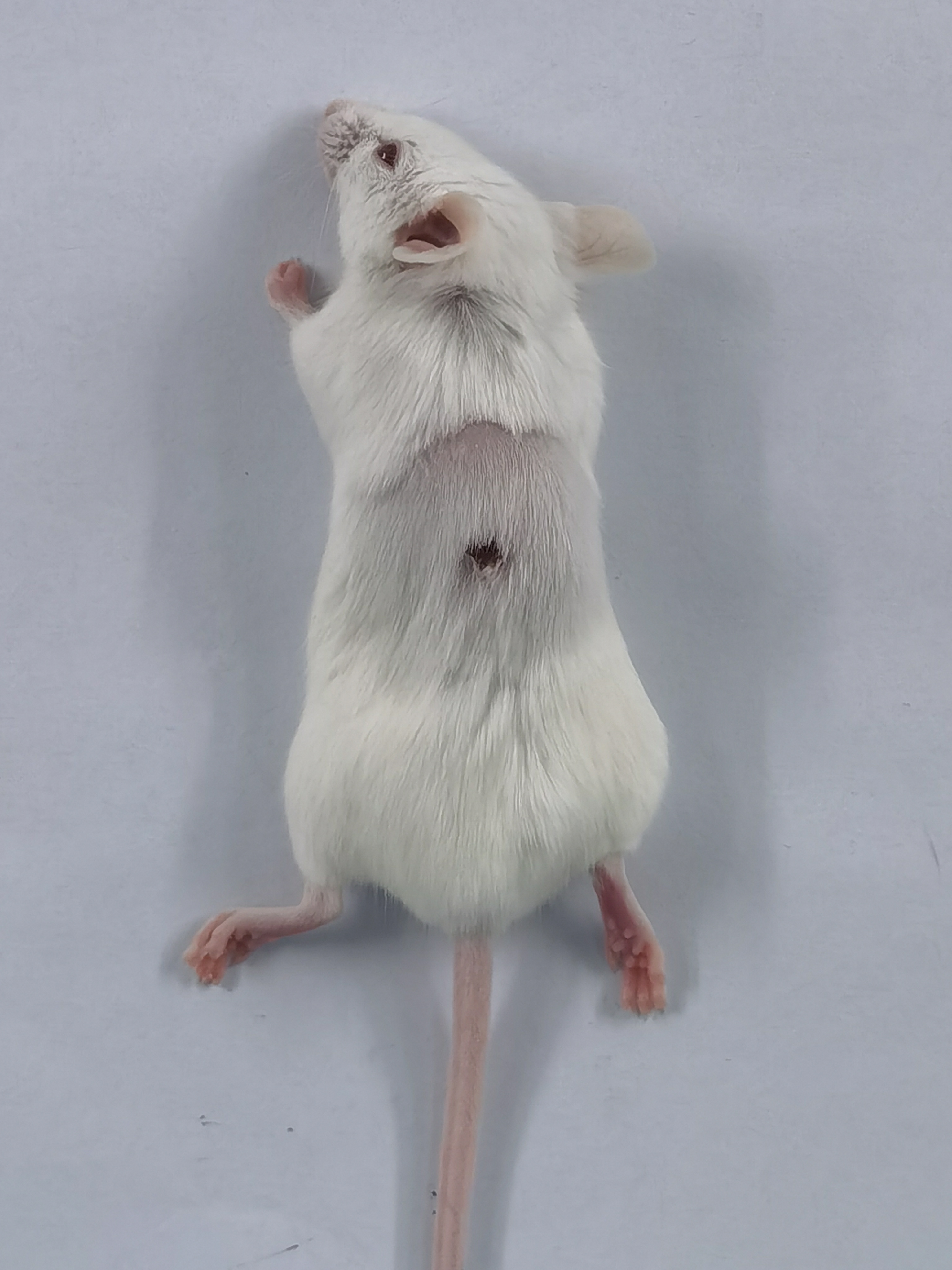

Supplement: Supplementary file 11 — Source data Fig. 6 [file 44321_2026_418_MOESM11_ESM.zip › Figure 6/Data-Figure 6B/Day 6/4-1.jpg]

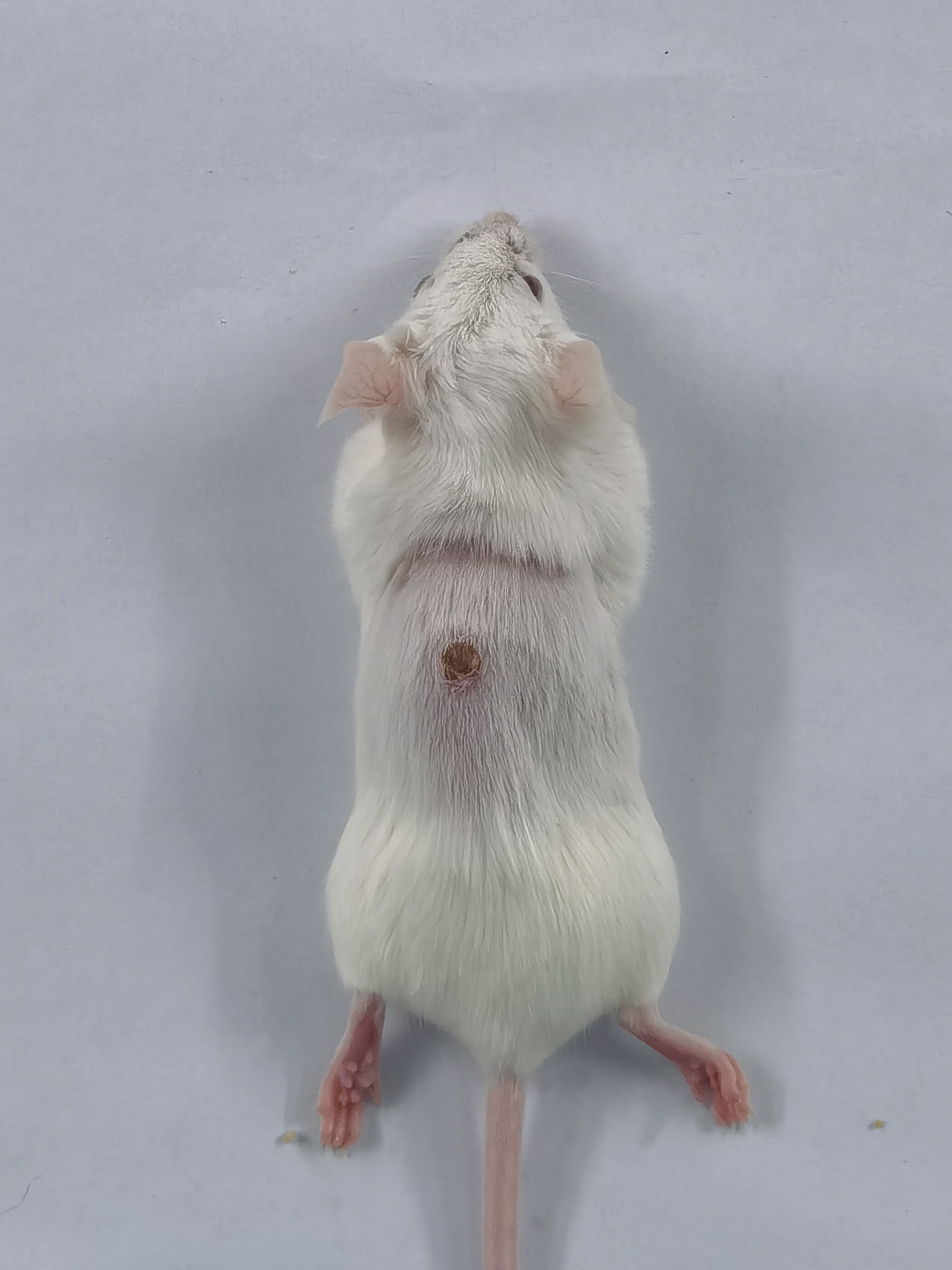

Supplement: Supplementary file 11 — Source data Fig. 6 [file 44321_2026_418_MOESM11_ESM.zip › Figure 6/Data-Figure 6B/Day 6/4-3.jpg]

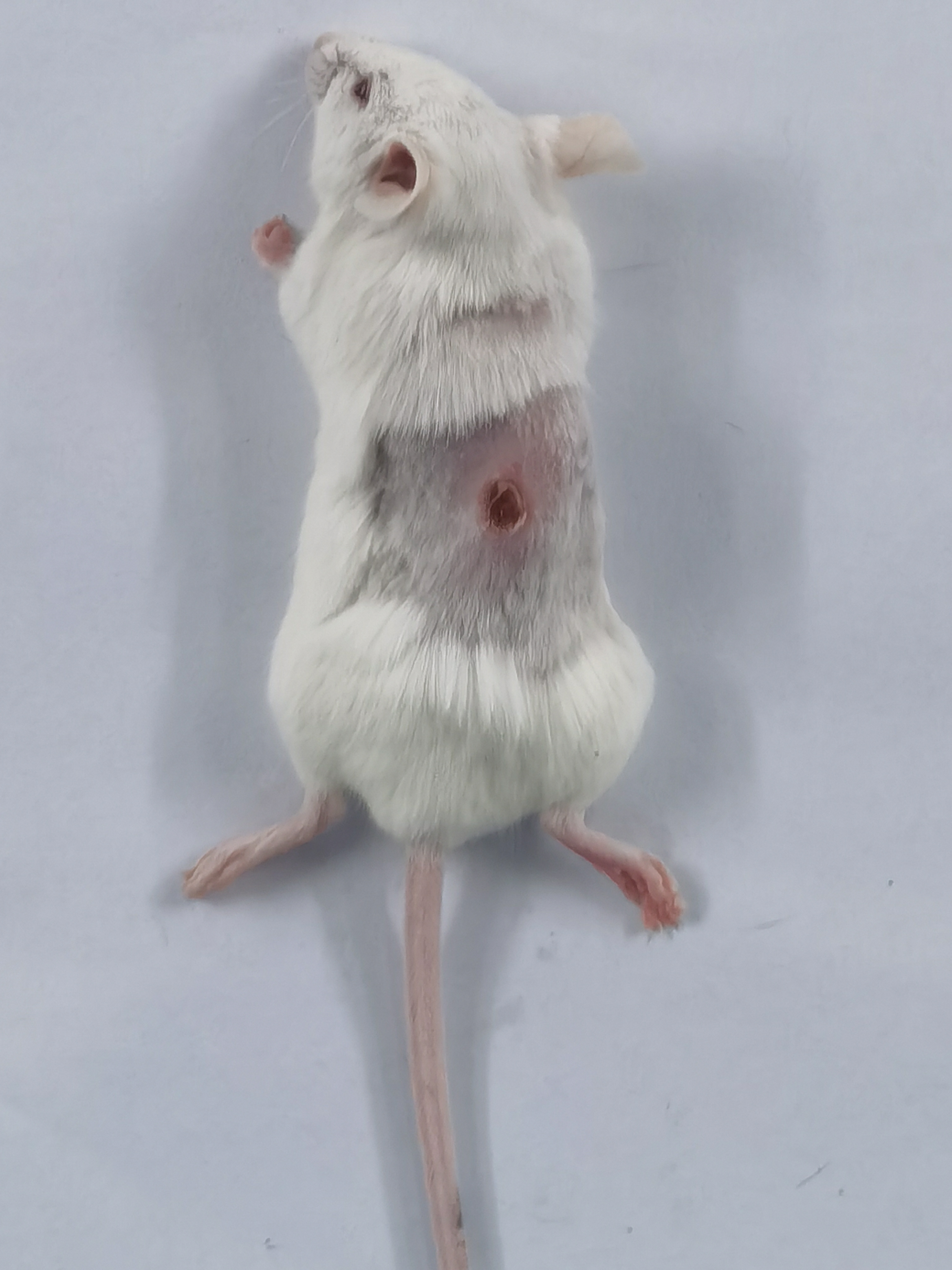

Supplement: Supplementary file 11 — Source data Fig. 6 [file 44321_2026_418_MOESM11_ESM.zip › Figure 6/Data-Figure 6B/Day 6/2-5.jpg]

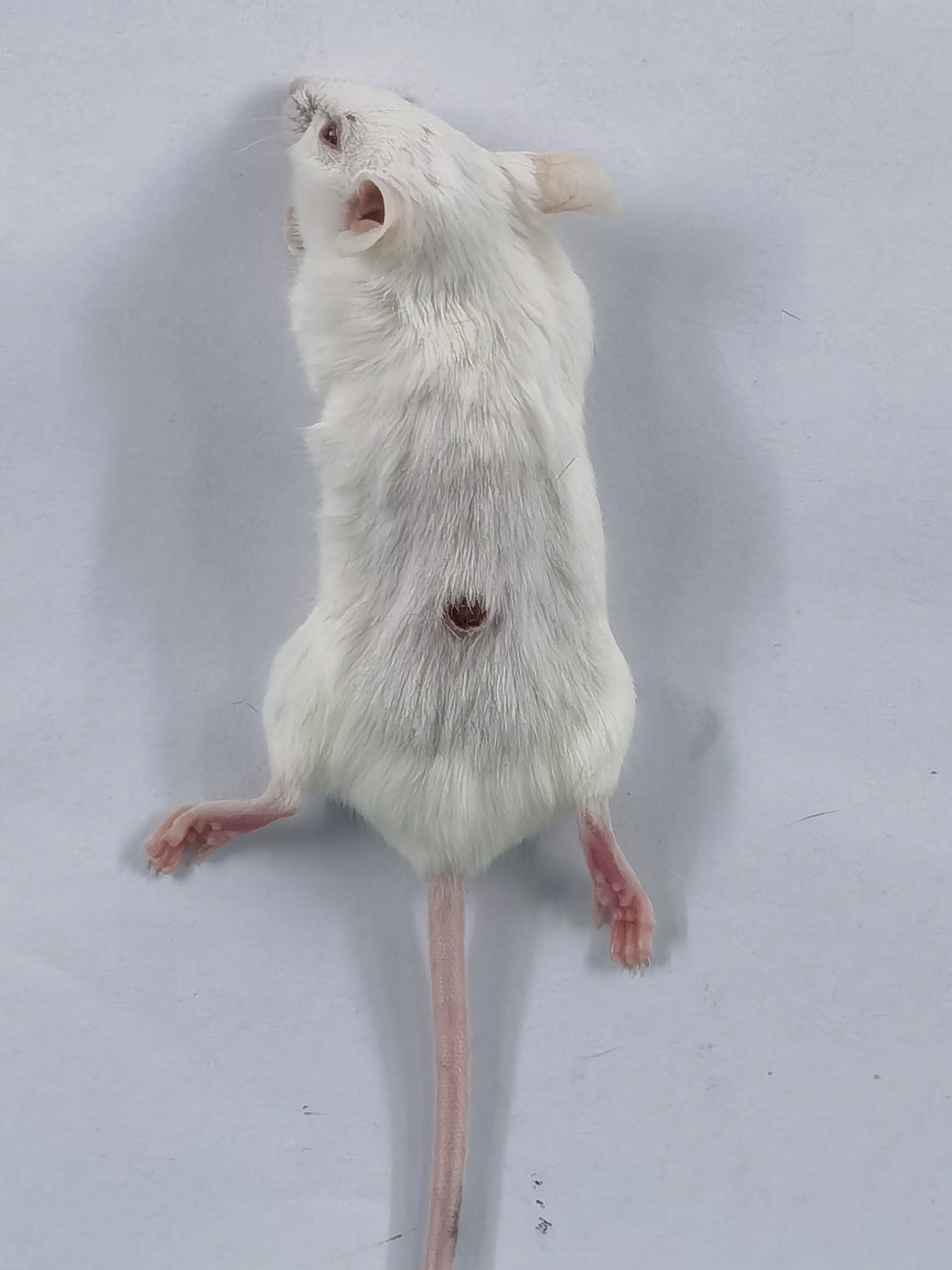

Supplement: Supplementary file 11 — Source data Fig. 6 [file 44321_2026_418_MOESM11_ESM.zip › Figure 6/Data-Figure 6B/Day 6/2-4.jpg]

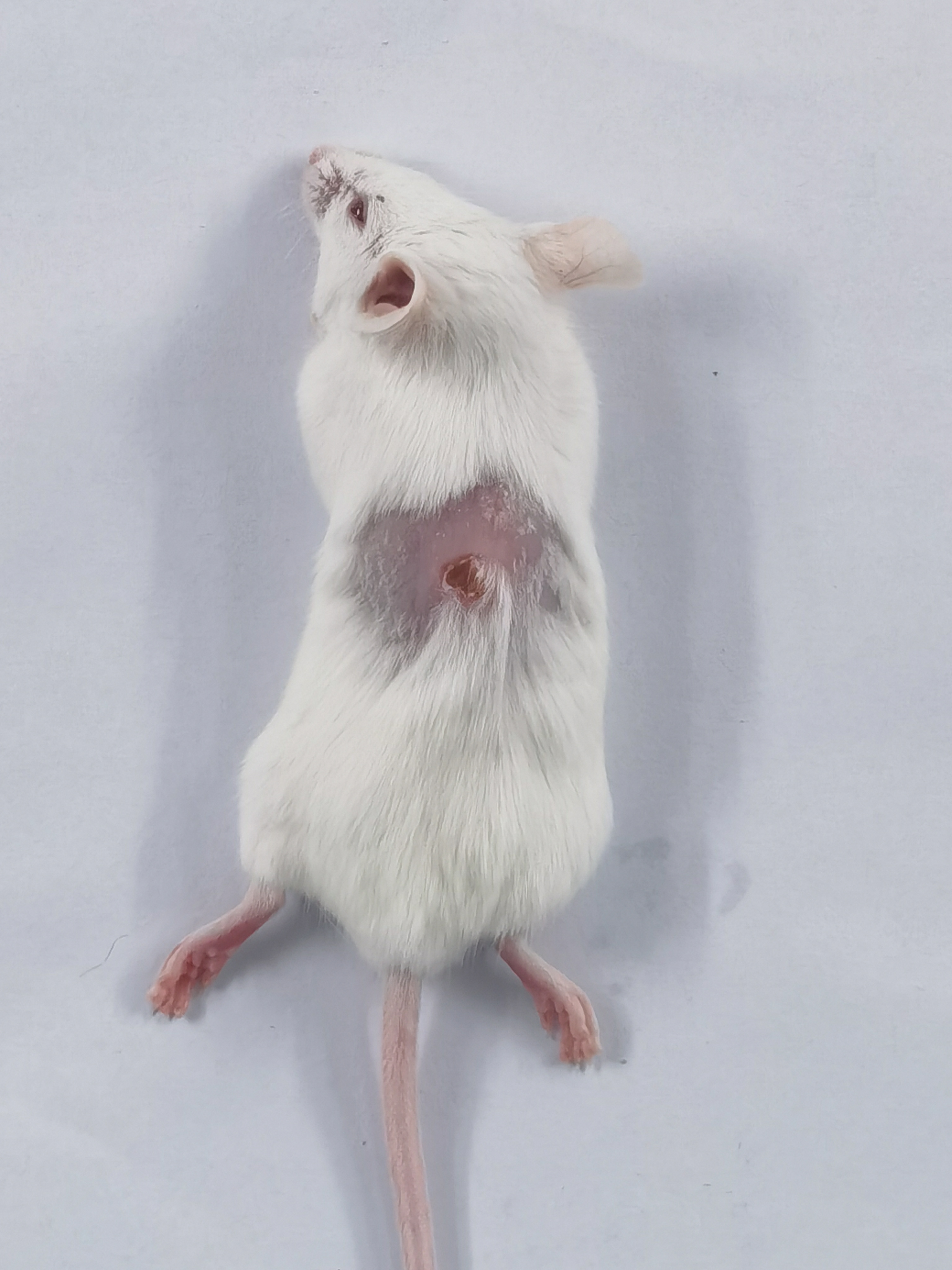

Supplement: Supplementary file 11 — Source data Fig. 6 [file 44321_2026_418_MOESM11_ESM.zip › Figure 6/Data-Figure 6B/Day 6/4-2.jpg]

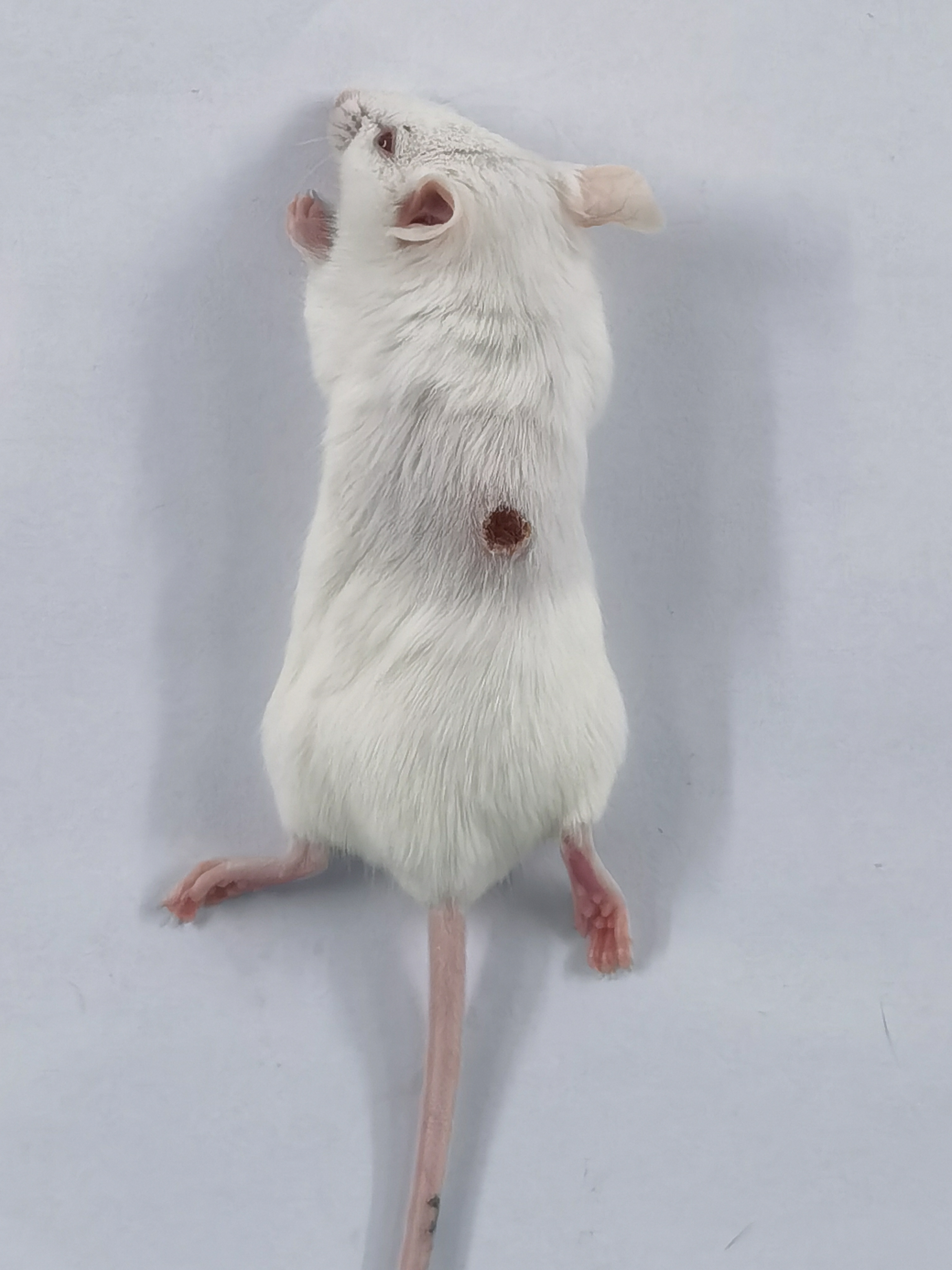

Supplement: Supplementary file 11 — Source data Fig. 6 [file 44321_2026_418_MOESM11_ESM.zip › Figure 6/Data-Figure 6B/Day 6/3-3.jpg]

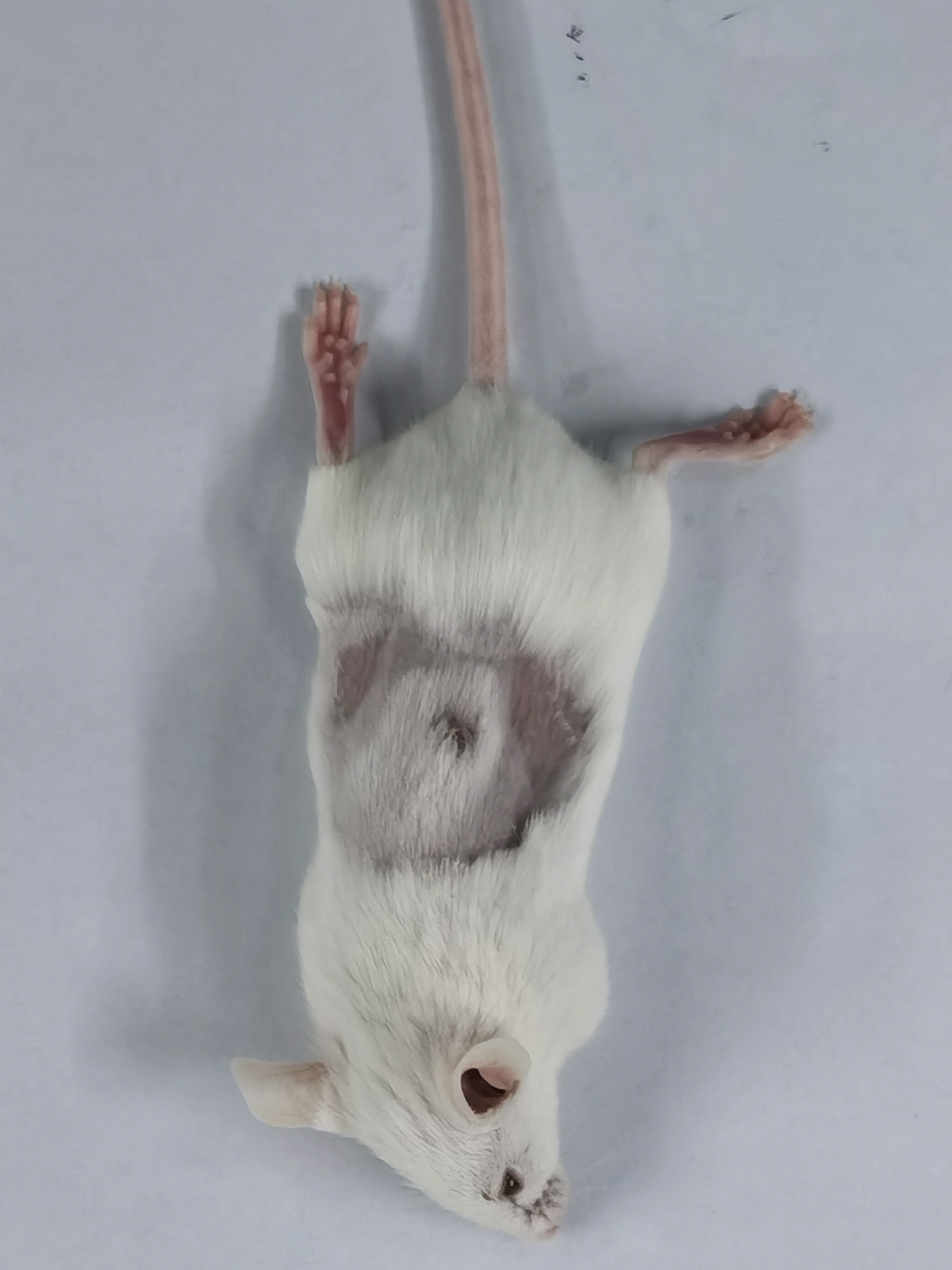

Supplement: Supplementary file 11 — Source data Fig. 6 [file 44321_2026_418_MOESM11_ESM.zip › Figure 6/Data-Figure 6B/Day 6/1-1.jpg]

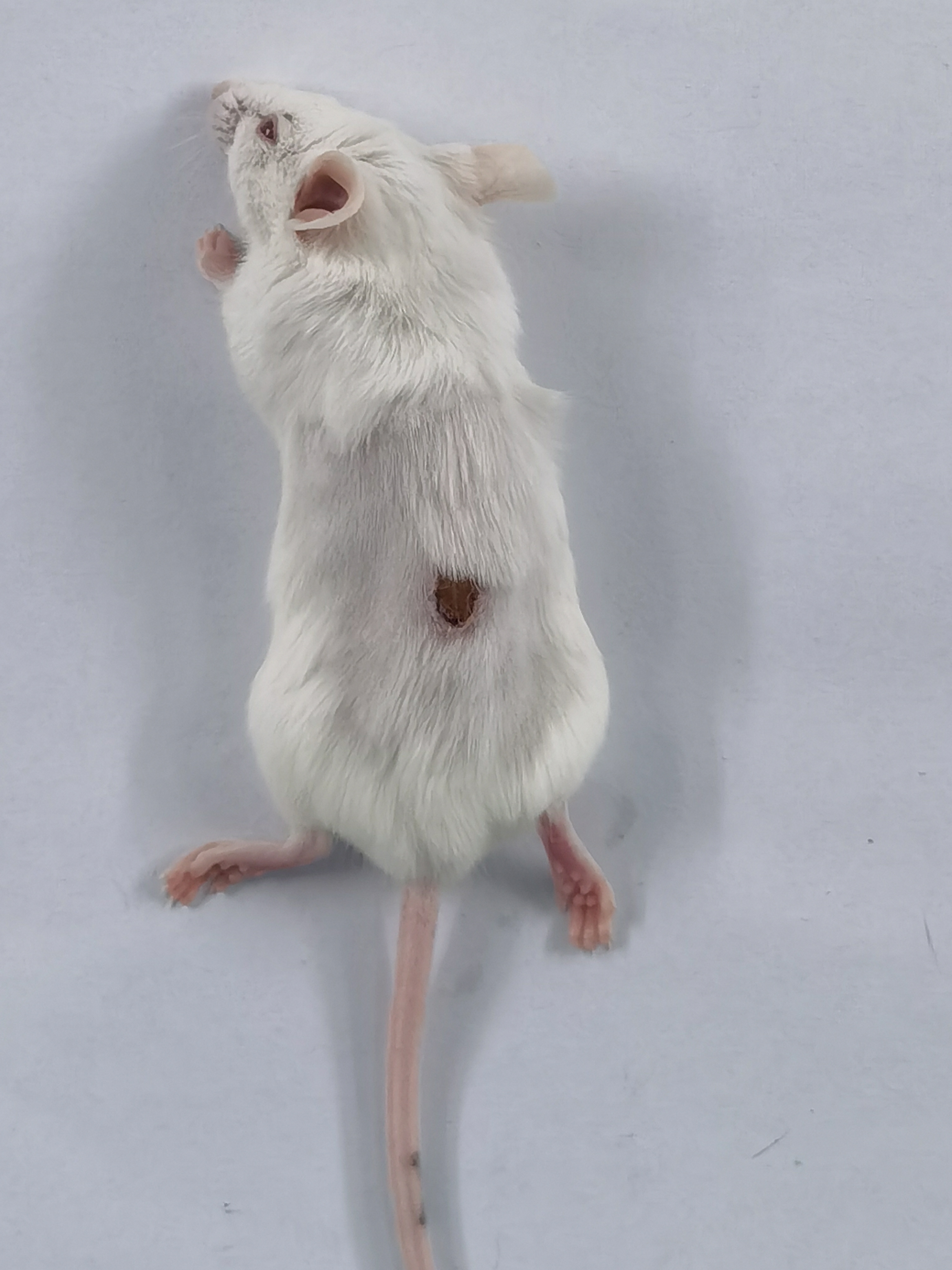

Supplement: Supplementary file 11 — Source data Fig. 6 [file 44321_2026_418_MOESM11_ESM.zip › Figure 6/Data-Figure 6B/Day 6/3-2.jpg]

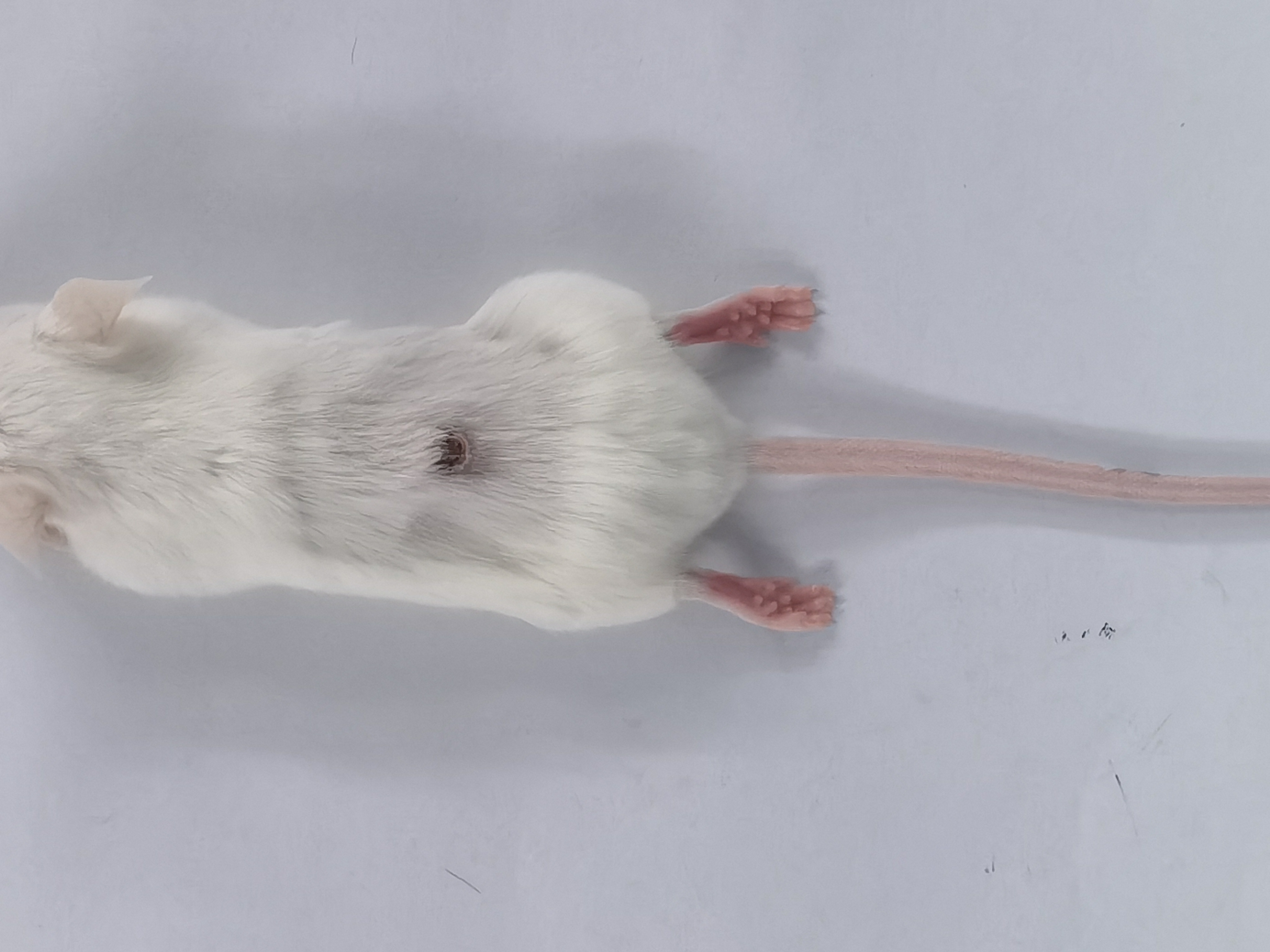

Supplement: Supplementary file 11 — Source data Fig. 6 [file 44321_2026_418_MOESM11_ESM.zip › Figure 6/Data-Figure 6B/Day 6/1-2.jpg]

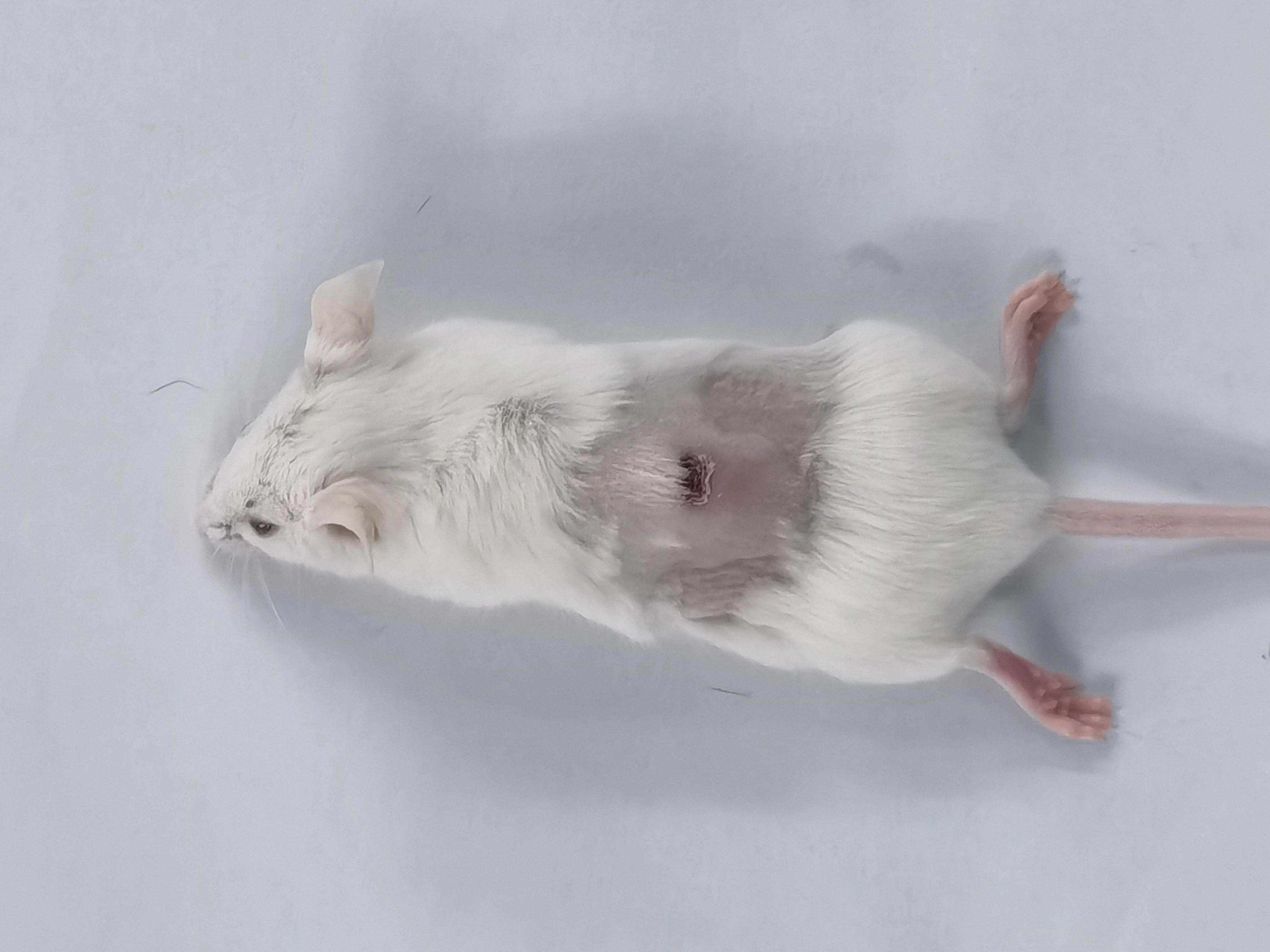

Supplement: Supplementary file 11 — Source data Fig. 6 [file 44321_2026_418_MOESM11_ESM.zip › Figure 6/Data-Figure 6B/Day 6/1-3.jpg]

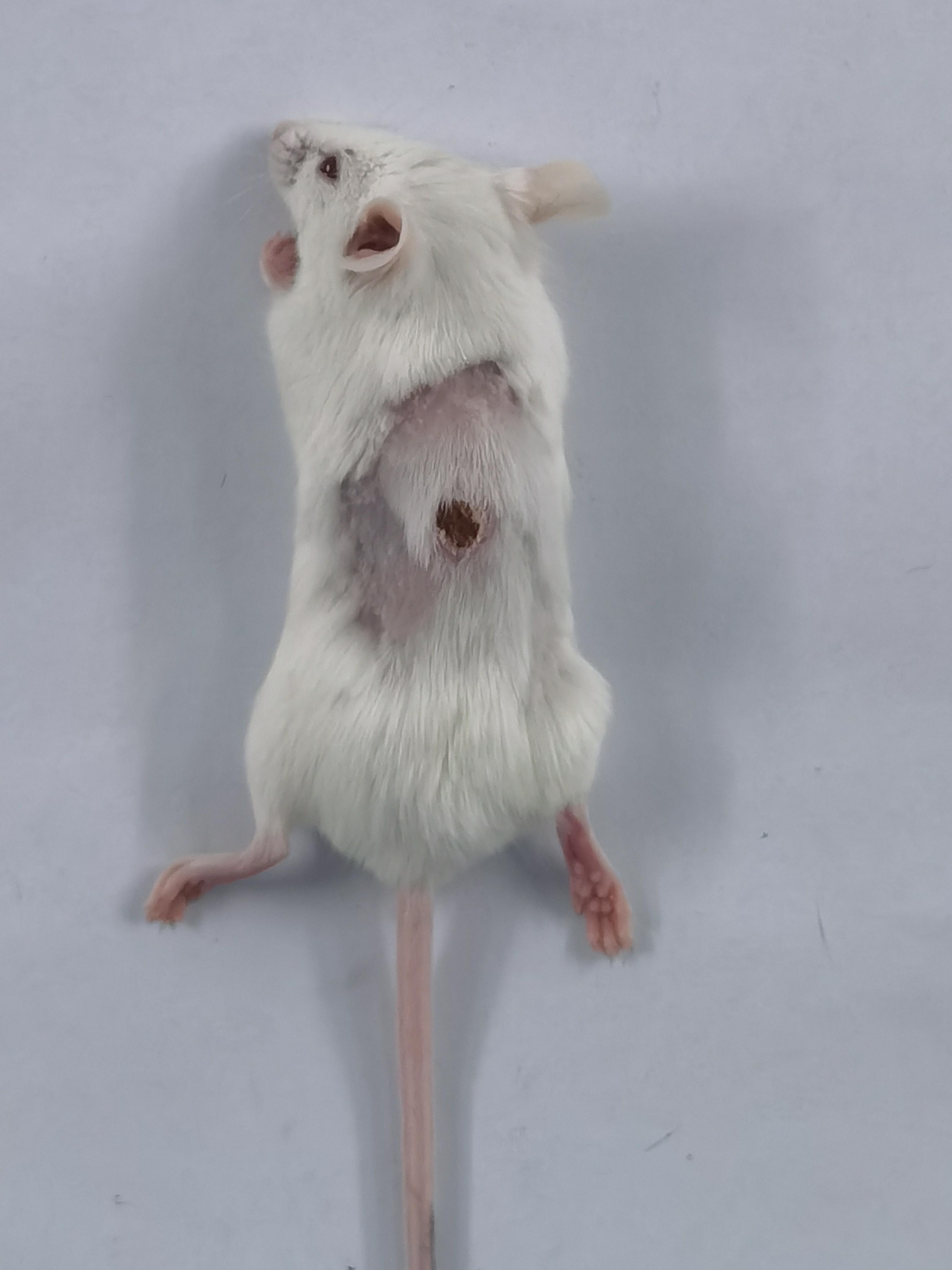

Supplement: Supplementary file 11 — Source data Fig. 6 [file 44321_2026_418_MOESM11_ESM.zip › Figure 6/Data-Figure 6B/Day 6/3-1.jpg]

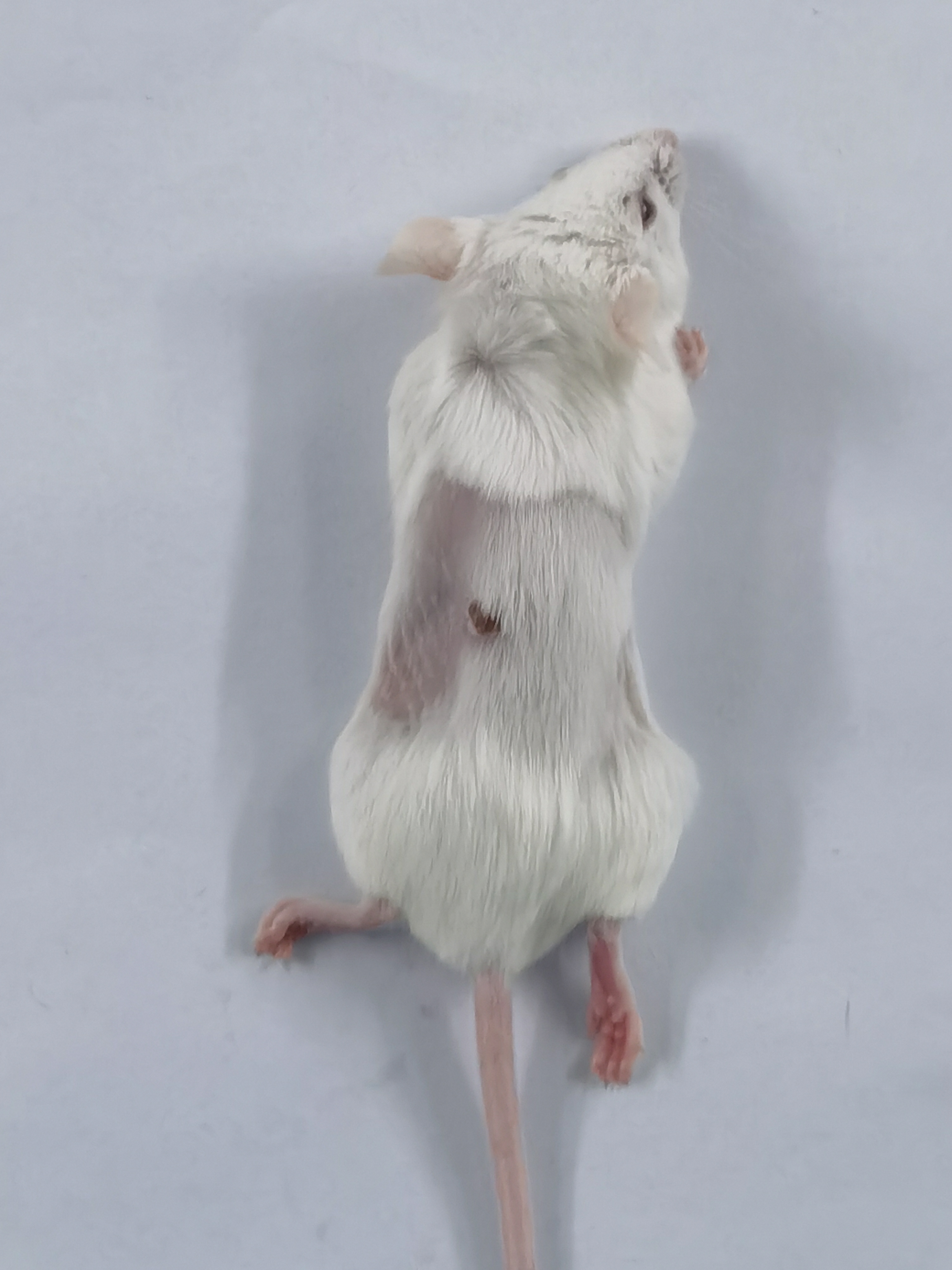

Supplement: Supplementary file 11 — Source data Fig. 6 [file 44321_2026_418_MOESM11_ESM.zip › Figure 6/Data-Figure 6B/Day 6/3-5.jpg]

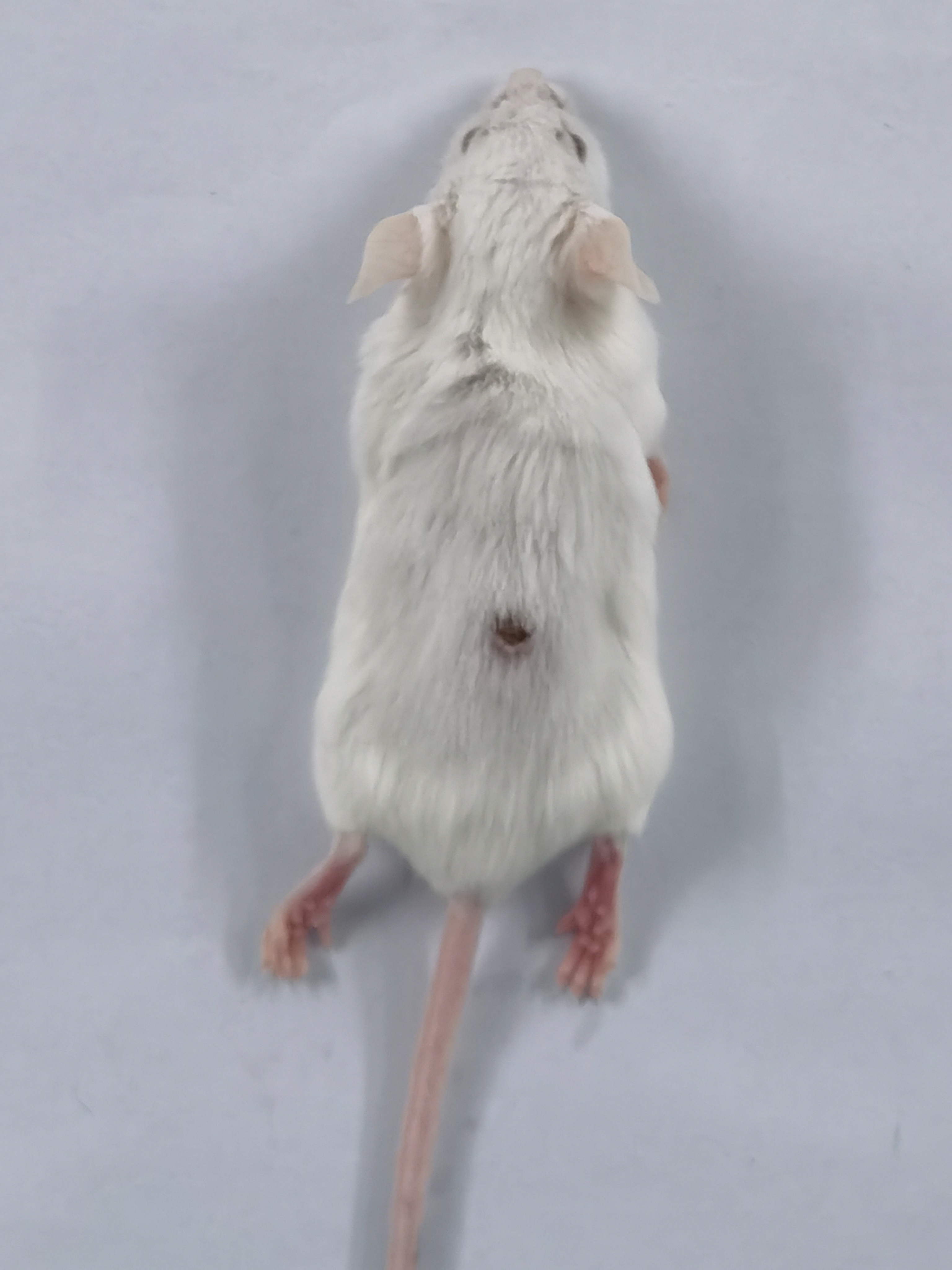

Supplement: Supplementary file 11 — Source data Fig. 6 [file 44321_2026_418_MOESM11_ESM.zip › Figure 6/Data-Figure 6B/Day 6/3-4.jpg]

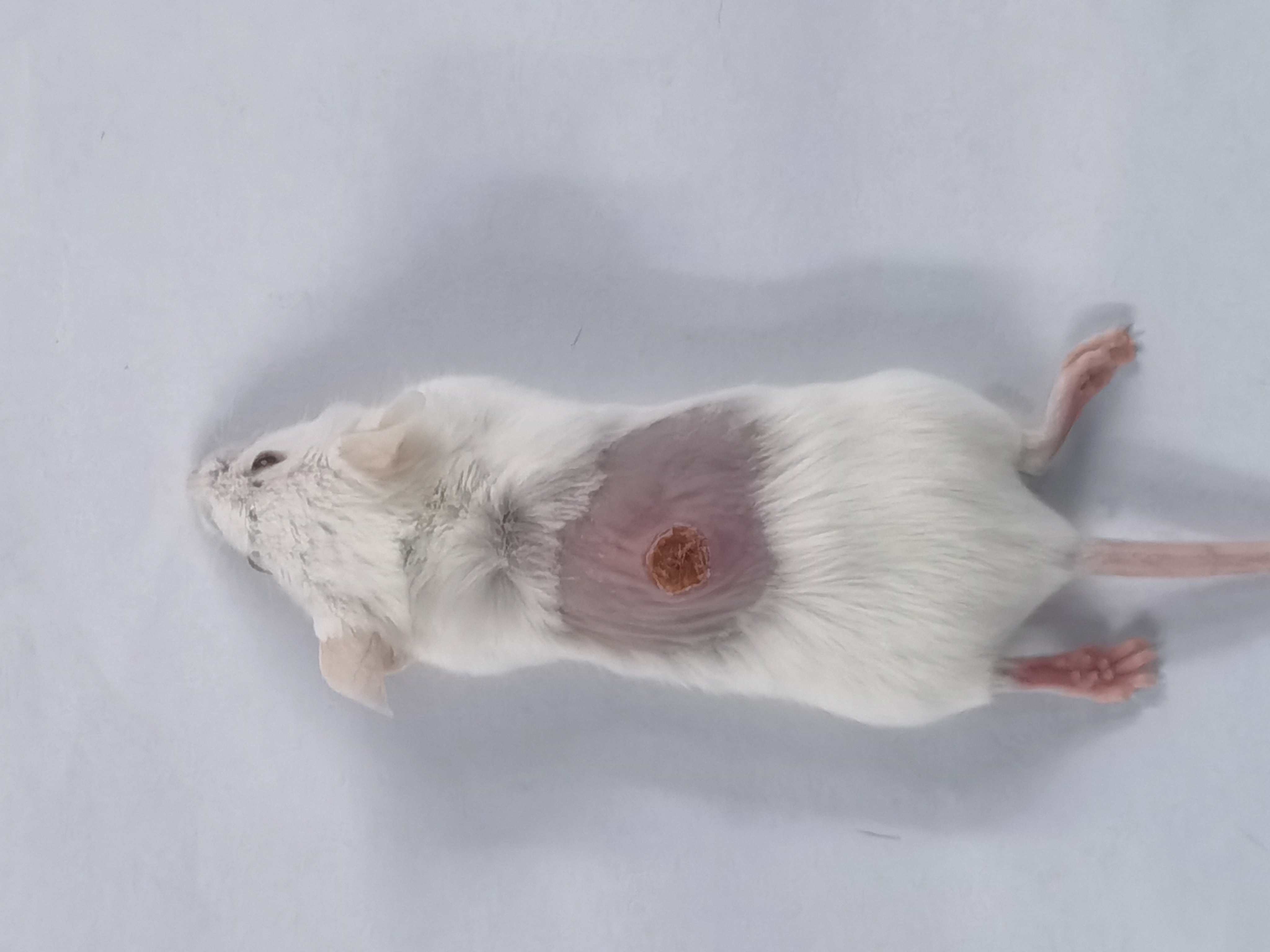

Supplement: Supplementary file 11 — Source data Fig. 6 [file 44321_2026_418_MOESM11_ESM.zip › Figure 6/Data-Figure 6B/Day 6/1-4.jpg]

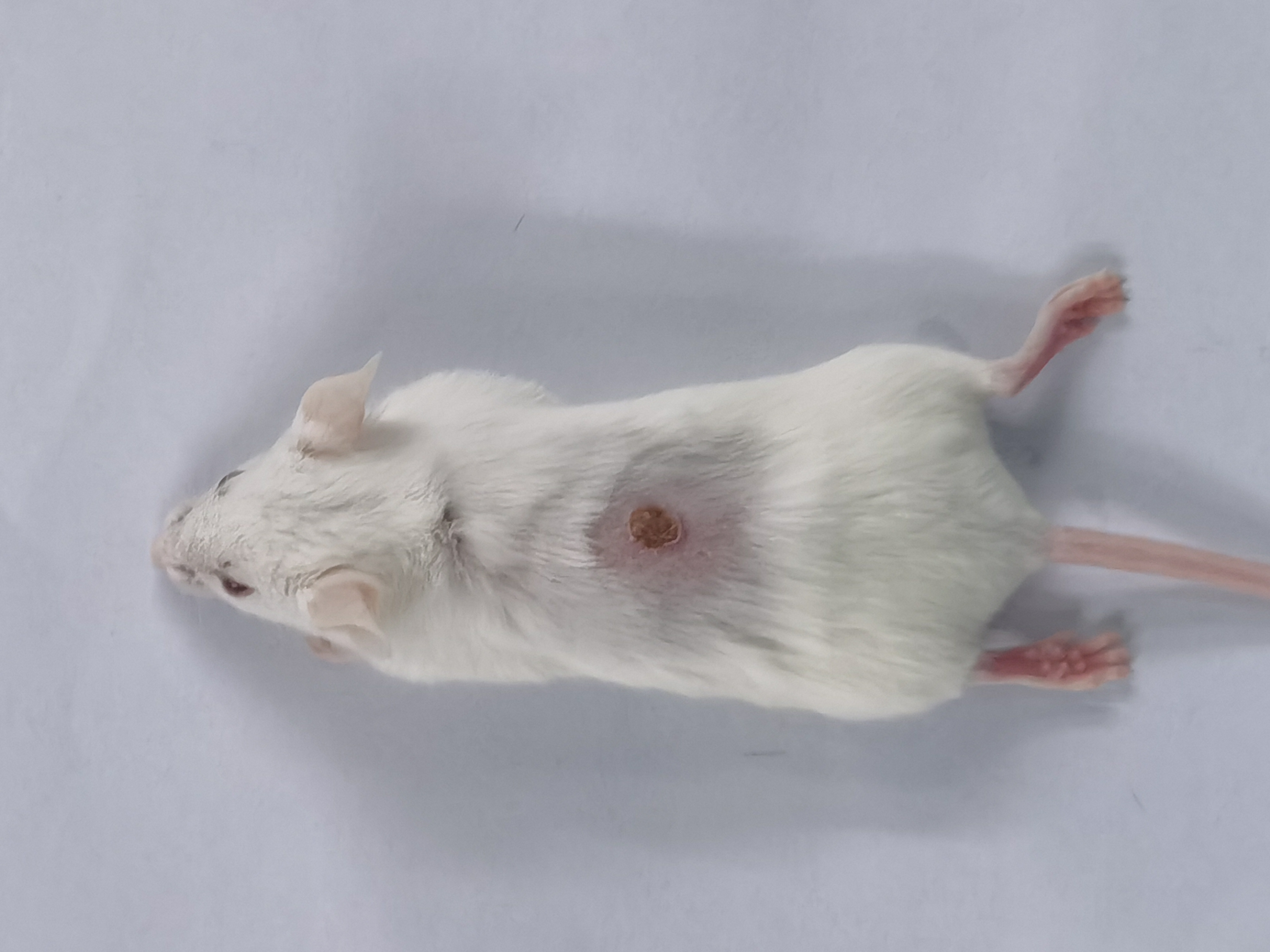

Supplement: Supplementary file 11 — Source data Fig. 6 [file 44321_2026_418_MOESM11_ESM.zip › Figure 6/Data-Figure 6B/Day 6/1-5.jpg]
